# A Genome-Wide Identification Study Reveals That *HmoCYP76AD1*, *HmoDODAα1* and *HmocDOPA5GT* Involved in Betalain Biosynthesis in *Hylocereus*

**DOI:** 10.3390/genes12121858

**Published:** 2021-11-23

**Authors:** Qingzhu Hua, Canbin Chen, Fangfang Xie, Zhike Zhang, Rong Zhang, Jietang Zhao, Guibing Hu, Yonghua Qin

**Affiliations:** Guangdong Provincial Key Laboratory of Postharvest Science of Fruits and Vegetables/Key Laboratory of Biology and Genetic Improvement of Horticultural Crops (South China), Ministry of Agriculture and Rural Affairs, College of Horticulture, South China Agricultural University, Guangzhou 510642, China; huaqingzhu@stu.scau.edu.cn (Q.H.); Chenjiayi98@stu.scau.edu.cn (C.C.); xiefangfang202012@163.com (F.X.); poloky2@163.com (Z.Z.); r-zhang@scau.edu.cn (R.Z.); zhaojietang@gmail.com (J.Z.); guibing@scau.edu.cn (G.H.)

**Keywords:** pitaya, betalain biosynthesis, genome-wide identification, *HmoCYP76AD1*, *HmoDODAα1*, *HmocDOPA5GT*

## Abstract

Betalains are water-soluble nitrogen-containing pigments with multiple bioactivities. Pitayas are the only at large-scale commercially grown fruit containing abundant betalains for consumers. Currently, the key genes involved in betalain biosynthesis remain to be fully elucidated. Moreover, genome-wide analyses of these genes in betalain biosynthesis are not available in betalain-producing plant species. In this study, totally 53 genes related to betalain biosynthesis were identified from the genome data of *Hylocereus undatus*. Four candidate genes i.e., one cytochrome P-450 R gene (*HmoCYP76AD1*), two L-DOPA 4,5-dioxygenase genes (*HmoDODAα1* and *HmoDODAα2*), and one cyclo-DOPA 5-O glucosyltransferase gene (*HmocDOPA5GT*) were initially screened according to bioinformatics and qRT-PCR analyses. Silencing *HmoCYP76AD1*, *HmoDODAα1*, *HmoDODAα2* or *HmocDOPA5GT* resulted in loss of red pigment. *HmoDODAα1* displayed a high level of L-DOPA 4,5-dioxygenase activity to produce betalamic acid and formed yellow betaxanthin. Co-expression of *HmoCYP76AD1*, *HmoDODAα1* and *HmocDOPA5GT* in *Nicotiana benthamiana* and yeast resulted in high abundance of betalain pigments with a red color. These results suggested that *HmoCYP76AD1*, *HmoDODAα1*, and *HmocDOPA5GT* play key roles in betalain biosynthesis in *Hylocereus*. The results of the present study provide novel genes for molecular breeding programs of pitaya.

## 1. Introduction

Betalains are a group of hydrophilic nitrogen-containing pigments with multiple functions [1,2,3]. Betalains are usually used as functional additives in pharmaceuticals, cosmetic, and food products due to their antimicrobial effects and natural colorant [4]. In addition, betalains are involved in plant protection against abiotic and biotic stresses [5,6,7,8,9,10]. Therefore, it is of great significance to enhance the betalain contents in plants through genetic transformation.

Betalains are secondary metabolites derived from L-tyrosine. The betalain biosynthetic pathway can be divided into three stages [11,12]. At present, cytochrome P450 (CYP), 4,5-dihydroxy-phenylalanine (DOPA)-dioxygenase (DOD), and glucosyltransferase (GT) have been characterized as key players in betalain biosynthesis. CYP enzyme can catalyze the hydroxylation of tyrosine to L-3,4-dihydroxyphenylalanine (L-DOPA) and the subsequent oxidation of DOPA to 5,6-dihydroxyindoline-2-carboxylic acid (cyclo-DOPA) [12,13]. Based on phylogenetic analyses of 100 Caryophyllales plants, *CYP76AD1-like* genes are classified into CYP76AD1-α, CYP76AD1-β and CYP76AD1-γ [14]. The functions of *CYP76AD1-α* genes (*BvCYP76AD1*, *MjCYP76AD3*, *CqCYP76AD1* and *AtriCYP76AD1*) encoding betalain biosynthesis enzymes have been elucidated in *Beta vulgaris* [13], *Mirabilis jalapa* [15], *Chenopodium quinoa* [16] and *Amaranthus tricolor* [17]. The *CYP76AD1-β* genes (*BvCYP76AD5*, *BvCYP76AD6* and *MjCYP76AD15*) encoding the enzyme convert tyrosine into L-DOPA which have been confirmed in *M. jalapa* and *B. vulgaris* [18,19]. However, the function of *CYP76AD1-γ* genes remains unknown.

The key enzymatic step in betalain biosynthesis is the formation of betalamic acid [11,12]. 4,5-DOPA dioxygenase extradiol (DODA) catalyzes the L-DOPA to seco-DOPA and then convert to betalamic acid spontaneously [20]. L-DOPA 4,5-dioxygenase is a rate-limiting enzyme encoded by the *DODA* gene [21]. Within Caryophyllales, gene duplication in the *LigB*/*DODA* gene lineage gives rise to the DODAα and DODAβ clades, with at least one homologue from the DODAβ lineage and two paralogues from the DODAα lineage [14,22]. Repeated convergent acquisition of L-DOPA 4,5-dioxygenase activity is consistent with the gene duplication events within the DODAα clade in Caryophyllales [22]. In *B. vulgaris*, *BvDODAα1* exhibits high levels of L-DOPA 4,5-dioxygenase activity, compared with low L-DOPA 4,5-dioxygenase activity for *BvDODAα2* [12,13,23,24]. Gene duplications within the DODAα lineage related to L-DOPA 4,5-dioxygenase activity are found in *Amaranthus hypochondriacus* [25], *Parakeelya mirabilis* [24], *Stegnosperma halimifolium*, *Carnegiea gigantea*, *Mesembryanthemum crystallinum*, *Macarthuria australis*, *Telephium imperati*, *Polycarpon tetraphyllum*, *Cardionema ramosissimum*, *Spergularia marina*, *Limeum aethiopicum* and *Kewa caespitosa* [22]. Betalains are divided into betaxanthins (yellow-orange pigments) and betacyanins (red-violet pigments). Betaxanthins are from spontaneous formation of betalamic acid and amino acids or amines. The formation of betacyanins can be carried out through glycosylation by GT [11,12]. One of the glycosylation routes is performed directly on betanidin by UDPG-dependent betanidin 5-*O*-glucosyltransferase (B5GT)/betanidin 6-*O*-glucosyltransferase (B6GT) [26,27]. *B5GT* and *B6GT* have been isolated from *Dorotheanthus bellidiformis* [28,29]. Another glycosylation occurs on glucosylated cDOPA by the action of a cDOPA 5-*O*-glucosyltransferase (cDOPA-5GT), which was found in *M. jalapa*, *A. hypochondriacus* and *C. quinoa* [3,15,25,30].

Pitaya is a climbing vine cactus species in the family of Cactaceae. It is becoming increasingly fashionable worldwide due to its adaption to arid conditions, attractive appearance, delicious taste and rich nutrients [3,31,32]. Pitaya is the edible fruit containing high levels of betalains at large-scale commercial cultivation. Currently, there are mainly four types of pitayas, namely red peel with white pulp pitaya (*Hylocereus undatus*), red peel with red pulp pitaya (*Hylocereus monacanthus*), yellow peel with green scales and white pulp pitaya (*H. undatus*) and yellow peel with thorny and white pulp pitaya (*Hylocereus megalanthus/Selenicereus megalanthus*), which are commercially produced as fruit crops [33,34]. Recently, the coding genes of key enzymes involved in pitaya have been obtained from transcriptome database. Compared to *H. undatus*, *CYP76ADs*, *DODA* and *GTs* in red pulp were upregulated in *H. monacanthus* [35,36,37,38,39]. In addition, *HpNAC*, *HpGSTs* and *HpWRKY* may play roles in the regulation of betalain biosynthesis in coordination with MYB transcription factor in pitaya [37,40,41]. Recently, candidate genes CYP76AD-α, CYP76AD-β, and CYP76AD-γ involved in betalain biosynthesis were isolated from pitaya genome [42,43]. The DODA lineage experienced gene duplication resulting in two main clades: DODA-α and DODA-β. Betalain-related GT enzymes (B5GT, B6GT, and cDOPA5GT) from flavonoid-related GTs showed higher expression levels in *H. monacanthus* than that of *H. undatus*. However, genome-wide identification and function of these genes involved in betalain biosynthesis in pitaya are still not available. In this study, the genome-wide identification and characterization of genes related to betalain biosynthesis were performed using available *H. undatus* genome data [43]. The functions of candidate genes were analyzed through virus-induced gene silencing (VIGS), transient expression, yeast recombinant expression, and genetic transformation. The results of the present study will provide valuable information for a better understanding of betalain biosynthesis in pitaya.

## 2. Materials and Methods

### 2.1. Plant Materials

**Figure 1:**
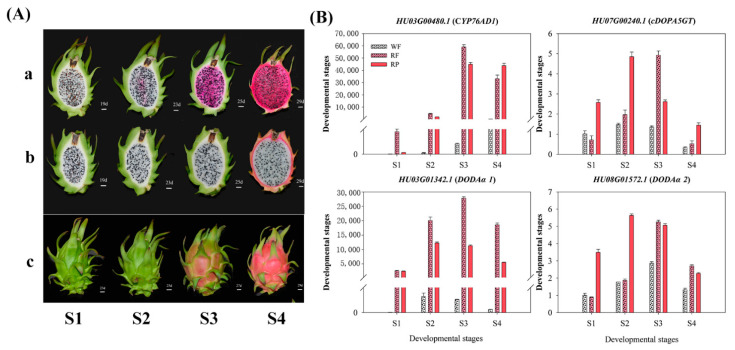
Identification of *H. undatus CYP76AD*, *DODA* and *GTs* as candidate betalain biosynthesis-related genes. (**A**) Pitaya peels and pulps from four developmental stages used for gene expression. (**a**) pulps of ‘Guanhuahong’ pitaya. (**b**) pulps of ‘Guanhuabai’ pitaya. (**c**) peels of ‘Guanhuahong’ pitaya. (**B**) Expression patterns of betalain biosynthesis-related genes of *HmoCYP76AD1*, *HmoDODAα1*, *HmoDODAα2* and *HmocDOPA5GT*. WF, pulps of ‘Guanhuabai’ pitaya; RF, pulps of ‘Guanhuahong’ pitaya; RP, peels of ‘Guanhuahong’ pitaya.

‘Guanhuahong’ (red peel and green scales with red pulp, *H.* *monacanthus*), ‘Hongguan No. 1′ (red peel and red scales with red pulp, *H. monacanthus*) and ‘Guanhuabai’ (red peel and green scales with white pulp, *H. undatus*) pitayas, *Arabidopsis thaliana* (Columbia, wild type) and *N. benthamiana* were used as materials. ‘Guanhuahong’ and ‘Guanhuabai’ pitayas were used to select the candidate genes due to the higher production of betalain in ‘Guanhuahong’ pitaya than that of ‘Guanhuabai’ pitaya [35]. Pulps of ‘Guanhuahong’ and ‘Guanhuabai’ pitayas were collected at four developmental stages (Figure 1A(a,b)): S1, 19 days after artificial pollination (DAAP); S2, 23 DAAP; S3, 25 DAAP and S4, 29 DAAP. Peels of ‘Guanhuahong’ pitaya were collected at four developmental stages (Figure 1A(c)): S1, 23 DAAP; S2, 25 DAAP; S3, 27 DAAP and S4, 29 DAAP. Three fruits were sampled at each developmental stage. Fruits with 40–50 cm stem of ‘Hongguan No. 1′ were collected on the 23 DAAP and cultivated in a climate room (26 °C; 16:8 h, light:dark) for VIGS. *A. thaliana* and *N. benthamiana* plants were cultivated in a climate room (22 °C; 70% humidity; 16:8 h, light:dark; 2500 Lux light intensity). All samples were immediately frozen in liquid nitrogen and stored at −80 °C until further use. All experiments were repeated in triplicate.

### 2.2. Gene Identification and Phylogenetic Analyses

Genes associated with betalain biosynthesis were downloaded from NCBI database according to the publications. The GenBank Accession No. are listed in Supplementary Table S1. Gene sequences were identified by BLAST search of *H. undatus* genomes [43] using TBtools software [44]. Annotated gene sequences were manually checked and adjusted to remove the repeated sequences and retain the genes with identity equal to or greater than 40%. Each potential gene was further manually examined to ensure the complete conserved domain using the Conserved Domain Database (CDD) (http://www.ncbi.nlm.nih.gov/cdd (accessed on 1 September 2019)).

Alignment of multiple sequences was performed with CLUSTAL (Bootstrap value = 1000). The phylogenetic tree was constructed using a neighbors-joining (NJ) method with MEGA7.0 software. The Newick tree was exported from MEGA7.0 and improved with iTOL (https://itol.embl.de/gallery.cgi (accessed on 20 July 2021)).

### 2.3. Chromosomal Distribution Analysis

The location information of genes related to betalain biosynthesis was obtained from the pitaya genome database [43]. The gene location map was constructed using TBtools software [44].

### 2.4. Relative Expression Analyses by qRT-PCR

qRT-PCR was performed with SYBR^®^ Premix Ex *Taq*™ II (Tli RNase H Plus) (TaKaRa, Shiga, Japan) in an ABI 7500 Real-Time PCR System (Applied Biosystems, New York, NY, USA) using the following process: initial denaturation step at 95 °C for 1 min, followed by 40 cycles of 15 s at 95 °C, 30 s at 58 °C and 40 s at 72 °C. The melting curve analyses were included in every reaction to confirm primer specificity and stability. A total of 10 μL reaction mixture included 1 μL of cDNA (Ct = 21), 0.5 μL of each primer (2 mM), 5 μL of 2× SYBR^®^ Premix Ex *Taq*™ II and 3 μL of double distilled water (ddH_2_O). Relative expression levels were calculated by the 2^−ΔΔC^^T^ method [45]. The data were normalized using the *Actin (1)* for pitaya [46], *AtActin2* (AT3G18780) for *A. thaliana* [47], and *NtActin1* for *N. benthamiana* [48], respectively. Three biological replicates for each reaction were performed. All qRT-PCR primers were designed by BatchPrimer3v1.0 (http://batchprimer3.bioinformatics.ucdavis.edu/index.html (accessed on 3 October 2019)) (Supplementary Table S2).

### 2.5. Full-Length cDNA Amplification and Plasmid Construction

Results from qRT-PCR indicated that all four candidate genes were preferentially expressed in pigmented tissues. Differences in nucleotide sequence and amino acids were detected between *H. monacanthus* and *H. undatus*. Therefore, the sequences from *H. monacanthus* were used in the subsequent experiments. Total RNA was extracted using EASYspin Plus Complex Plant RNA Kit (RN53) (Aidlab Biotechnology, Beijing, China) according to the manufacturer’s protocol. The cDNAs were synthesized using a PrimeScript^TM^ RT reagent Kit with gDNA Eraser (TaKaRa, Shiga, Japan). The full-length coding sequences of *HmoCYP76AD1* (MZ541999), *HmoDODAα1* (MZ542000), *HmoDODAα2* (MZ542001), and *HmocDOPA-5GT* (MZ542002) were cloned using Phanta^®^ Max Super-Fidelity DNA Polymerase (Vazyme, Nanjing, China). Vectors were constructed using ClonExpress^®^II One Step Cloning Kit (Vazyme, Nanjing, China) according to the manufacturer’s manual. Gene coding sequences were constructed into pEAQ-GFP and pC18 vectors for transient expression. pPZP6k90 vector harboring gene coding sequences was used for genetic transformation. All gene fragments were recombined into a pTRV2 vector for VIGS assay. Single-gene expression vectors (YES2-Ura, pYES3-Trp) and/or double-gene expression vector (pESC-Ura) were used for recombinant expression in yeast. The primers used for gene cloning and vector construction are listed in Supplementary Table S2. The information of all vectors is described in Supplementary Text S3.

### 2.6. Subcellular Localization Assay

The pEAQ-GFP fusion vectors (pEAQ-HmoCYP76AD1, pEAQ-HmoDODAα1, pEAQ-HmoDODAα2, and pEAQ-HmocDOPA-5GT), positive control GFP vector, and the nuclear and plasma membrane RFP marker [49] were transformed into *Agrobacterium tumefaciens* strain GV3101 (Coolaber, Beijing, China) using the freeze-thaw method. The bacterial suspensions harboring the RFP marker and pEAQ-GFP fusion vectors were mixed with 1:1 ratio and co-infiltrated into the abaxial side of 4- to 6-week-old *N. benthamiana* plants according to the method of Cheng et al. (2017) [40]. Two to three days after infiltration, the abaxial epidermis of the leaves was subjected to confocal microscopy (Zeiss LSM 7 DUO, Oberkochen, Germany). The fluorescence signals were detected using an emission bandwidth of 488/505-525 nm for GFP and 543/585-615 nm for RFP.

### 2.7. VIGS of Candidate Genes in H. monacanthus

Fragments of *HmoCYP76AD1* (421 bp), *HmoDODAα1* (407 bp), *HmoDODAα2* (365 bp), and *HmocDOPA-5GT* (491 bp) genes were cloned into the pTRV2 vector, transformed into *A. tumefaciens* strain GV3101 (Coolaber, Beijing, China) and infiltrated into the scales of ‘Hongguan No. 1’ pitaya on the 23 DAAP following the method of Xie et al. (2020) [39]. The *A. tumefaciens* was suspended to an OD_600_ of 0.4 with VIGS infiltration buffer (10 mM MES, 10 mM MgCl_2_ and 200 μM acetosyringone). Bacterial suspension harboring pTRV2-HmoCYP76AD1, pTRV2-HmoDODAα1, pTRV2-HmoDODAα2 or pTRV2-HmocDOPA-5GT vector and pTRV1 plasmid were mixed in a 1:1 ratio and used for co-infiltration. *A. tumefaciens* strains harboring pTRV2-HmB5GT1 and pTRV2 were used as positive and negative controls, respectively. Fifteen fruits were used for VIGS. Phenotypes were observed after 14 d of cultivation at 25 °C with 75% relative humidity. Samples were collected for betalain content and gene expression analyses. Betalains were extracted and measured following the method of Hua et al. (2016a) [35].

### 2.8. Genetic Transformation of A. thaliana

The constructed plasmids (pPZP6k90-HmoDODAα1 and pPZP6k90-HmoDODAα2) were separately introduced into the *A. tumefaciens* strain GV3101 (Coolaber, Beijing, China) and transferred to *A. thaliana* using the floral dipping transformation method [50]. T_0_ seeds were harvested, and transgenic lines were selected on MS medium supplemented with 100 mg/L kanamycin and confirmed by PCR analyses. T_3_ transgenic homozygous seedlings were used for phenotypic characterization and gene expression analyses.

Ten-day-old *A. thaliana* seedlings were transferred onto plates containing absorbent paper with 10 mM L-DOPA solution. Sterile water was substituted for the L-DOPA as a negative control. Inflorescences from transformed *A. thaliana* seedlings were collected and immersed in 10 mM L-DOPA solution or water for 24 h. Betalain pigment production in *A. thaliana* seedlings and inflorescences were photographed after 24 h treatment.

### 2.9. Transient Expression Assay in N. benthamiana

Transient expression vectors pC18 containing candidate genes (*HmoCYP76AD1*, *HmoDODAα1*, *HmoDODAα2*, *HmoB5GT1* and *HmocDOPA-5GT*) were introduced into *A. tumefaciens* strain GV3101 (Coolaber, Beijing, China), respectively. Transient expression in *N. benthamiana* was performed as described previously [22,51]. *A. tumefaciens* strains were grown overnight in YEP media and resuspended in infiltration buffer (10 mM MES, 10 mM MgCl_2_ and 100 μM acetosyringone) to a final OD_600_ of 0.2–0.3. *Agrobacteria* harboring different plasmids were mixed in a 1:1 ratio before infiltration. Pigment deposition in the infected *N. benthamiana* leaves was analyzed within 2–3 d after co-infiltration. Betalains were extracted and measured as described by Hua et al. (2016a) [35]. 0.1 g fresh leaves were ground and mixed with 1 mL 80% (*v*/*v*) aqueous methanol solution. After sonicating for 10 min, samples were centrifuged and kept at room temperature for 20 min in darkness. Then the supernatants were measured through spectrophotometry (Infinite M200, Shanghai, China) at 478 nm for betaxanthins and 538 nm for betacyanins. Three biological replicates were performed for each experiment.

### 2.10. Recombinant Expression in Yeast

Coding sequences of candidate genes related to betalain biosynthesis were recombined into pYES2-Ura (for *HmoCYP76AD1* and *HmocDOPA-5GT*), pYES3-Trp (for *HmoDODAα1* and *HmoDODAα2*) and pESC-Ura (for tandem *HmoCYP76AD1* and *HmocDOPA-5GT*) vectors, respectively. The constructed plasmids were transformed into *Saccharomyces cerevisiae* strain INVSc1 (Coolaber, Beijing, China) using the polyethylene glycol/lithium acetate (PEG/LiAc) method (Coolaber, Beijing, China). A single gene or different combinations of *CYP76AD1-DODA* and *CYP76AD1-DODA-cDOPA5GT* were transferred into yeast competent cells. Yeast strains were cultured overnight in SD medium lacking Ura and Trp (SD/SD- Ura -Trp). Single colonies were picked and transferred into liquid SC/SC- Ura -Trp with 2 mM ascorbate and 10 mM L-DOPA, and grown overnight at 200 rpm in a shaking water bath at 30 °C. An aliquot of 1 mL supernatant was transferred to a rotary evaporator to remove water, re-suspended in 100 μL of ddH_2_O, and then detected by spectral analysis (Infinite M200, Shanghai, China).

### 2.11. Statistical Analysis

The experiments were carried out at least three individual biological replicates. Data in figures are the mean ± standard errors (S.E.). Statistical analysis of one-way ANOVA was used to determine the significant differences using SPSS 17.0 software at *p* < 0.05 and *p* < 0.01.

## 3. Results

### 3.1. Identification and Classification of Candidate Genes Involved in Betalain Biosynthesis

Potential encoding genes involved in pitaya betalain biosynthesis were comprehensively screened by alignment using TBtools (Table S1). These candidate genes were further manually screened according to the completeness of conserved domains. A total of 53 candidate genes related to betalain biosynthesis were identified in the pitaya genome (Table S3), including 15 *CYP76AD*, 3 *DODA* and 35 *GTs* genes.

**Figure 2:**
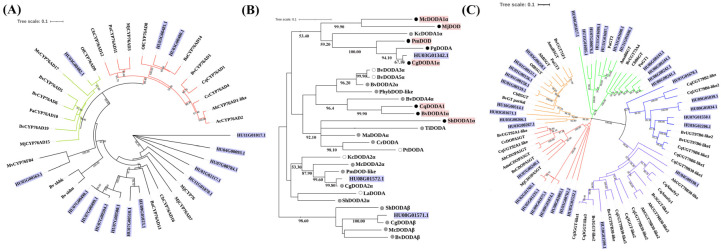
Neighbor-Joining phylogeny of *CYP76AD* (**A**), *DODA* (**B**) and *GTs* (**C**) with the other plant species. (**A**) The red lines indicate α evolution branch, green lines indicate β evolution branch and the other belong to γ branch; (**B**) Red background labeled tips show functionally characterized DODAs. Purple rhombus indicates candidate genes from *H. undatus*. Labeled tips shaded squares correspond to DODA activity (white, no activity; grey, marginal activity; black, high activity); (**C**) Branches are colored according to the putative characterized GTs (red, CDOPA5GT; orange, B5GT; green, B6GT; blue, UGT79B6; purple, UGT79B30). Purple label backgrounds indicate candidate genes from *H. undatus*.

Three phylogenetic trees were generated to analyze the phylogenetic relationships of the 53 candidate genes (Figure 2). The 15 candidate *CYP76AD* proteins were classified into CYP76AD-α, CYP76AD-β and CYP76AD1-γ clades (Figure 2A). *HU03G00480.1* and *HU03G00481.1* belonged to the CYP76ADα clade, which synthesize both L-DOPA and cyclo-DOPA. *HU03G00482.1* belonged to the CYP76ADβ clade, exhibiting tyrosine hydroxylase activity. The other 12 genes belonged to unknown CYP76AD1-γ clade (Figure 2A). Three DODAs (*HU03G01342.1*, *HU08G01571.1* and *HU08G01572.1*) and 29 homologous proteins from betalain-producing plants were further analyzed. *HU03G01342.1* and *HU08G01572.1* belonged to the DODAα clade, while *HU08G01571.1* was classified into the DODAβ clade (Figure 2B). GTs were divided into five groups: B5GT, B6GT, cDOPA5GT, UGT79B6 and UGT7B30. Among the 35 GTs proteins, nine belonged to B5GT, 11 to B6GT, eight to cDOPA5GT, five to UGT79B6, and two to UGT7B30 (Figure 2C).

### 3.2. Genomic Distribution among Pitaya Chromosomes

**Figure 3:**
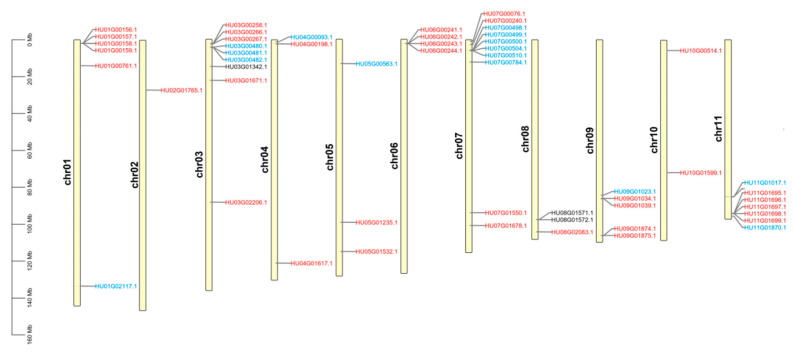
Chromosomal localization of *CYP76AD*, *DODA* and *GTs* genes in the *H. undatus* genome. *CYP76AD* (Blue): HU01G02117.1, HU03G00480.1, HU03G00481.1, HU03G00482.1, HU04G00093.1, HU05G00563.1, HU07G00498.1, HU07G00499.1, HU07G00500.1, HU07G00504.1, HU07G00510.1, HU07G00784.1, HU09G01023.1, HU11G01017.1 and HU11G01870.1; *DODA* (Black): HU03G01342.1, HU08G01571.1 and HU08G01572.1; *GTs* (Red): HU01G00156.1, HU01G00157.1, HU01G00158.1, HU01G00159.1, HU01G00761.1, HU02G01765.1, HU03G00258.1, HU03G00266.1, HU03G00267.1, HU03G01671.1, HU03G02206.1, HU04G00198.1, HU04G01617.1, HU05G01235.1, HU05G01532.1, HU06G00241.1, HU06G00242.1, HU06G00243.1, HU06G00244.1, HU07G00076.1, HU07G00240.1, HU07G01550.1, HU07G01678.1, HU08G02083.1, HU09G01034.1, HU09G01039.1, HU09G01874.1, HU09G01875.1, HU10G00514.1, HU10G01599.1, HU11G01695.1, HU11G01696.1, HU11G01697.1, HU11G01698.1 and HU11G01699.1.

The 53 candidate genes were mapped on 11 pitaya chromosomes (Figure 3). *CYP76AD*, *DODA*, and *GTs* genes were distributed on different chromosomes. Most of these candidate genes were mapped on Chr 3 (9 genes; 17.0%) and Chr 7 (10 genes; 18.7%). All *DODA* genes were distributed on Chr 3 and Chr 8. *GTs* were uniformly distributed on Chr 4 to Chr 11, while no *CYP76AD* was detected on Chr 2, Chr 6 and Chr 8.

### 3.3. Gene Characteristics and Structures

The lengths of the genomic and protein sequences, isoelectric point (pI), molecular weight (MW), grand average of hydropathicity (GRAVY), number of exons and instability index of the 53 candidate genes were comprehensively analyzed to obtain more information about the nature of the proteins (Table S3 and Figure S1). The genomic sequence lengths of *CYP76ADs* ranged from 1796 bp (*HU09G01023.1*) to 71,367 bp (*HU11G01017.1*). The protein lengths ranged from 438 amino acids (HU07G00784.1) to 1835 amino acids (HU11G01017.1) with MW 49.88 to 208.48 kDa. The pI ranged from 5.98 (HU07G00784.1) to 9.39 (HU03G00481.1). Twelve proteins had a negative GRAVY score, indicating that they were hydrophilic in nature. Moreover, eight proteins were unstable (Table S3). Most of the full-length *CYP76AD* genes had two exons, except that *HU01G02117.1*, *HU07G00500.1*, *HU07G00841.1*, *HU04G00093.1*, and *HU11G01017.1* had 3, 4, 4, 5 and 8 exons, respectively (Figure S1).

The genomic full-length sequences of *DODAs* were 9485 bp (*HU03G01342.1*), 3921 bp (*HU08G01571.1*) and 3882 bp (*HU08G01572.1*), respectively. The protein lengths ranged from 271 amino acids (*HU07G00784.1*) to 375 amino acids (*HU11G01017.1*); the MW were from 29.94 kDa to 42.17 kDa. The pI of DODAα (HU03G01342.1 and HU08G01572.1) were lower than that of DODAβ (HU08G01571.1). HU03G01342.1, HU08G01571.1 and HU08G01572.1 were hydrophilic stable proteins (Table S3). *DODA* genes had the same exon/intron structure: three exons and two introns (Figure S1).

The genomic full-length sequences of *GTs* ranged from 1392 bp (*HU03G02206.1* and *HU07G01550.1*) to 21,223 bp (*HU04G00198.1*). The proteins were mainly distributed between 400 bp and 500 bp in length. The average molecular mass of GTs was ~52.70 kDa except for HU04G00198.1 (96.81 kDa). The encoded proteins were weak acidic protein as supported by their pI. Only five GT proteins had hydrophilicity greater than 0 indicating that most of these GT proteins were hydrophilic (Table S3). The 35 *GTs* genes had an average of 1.3 exons. Among these GTs proteins, 66% proteins were unstable and 77% contained one exon (Figure S1).

### 3.4. Expression Patterns of Candidate Genes Involved in Betalain Biosynthesis

The expression patterns of 36 candidate genes (nine *CYP76ADs*, three *DODAs* and 24 *GTs*) involved in pitaya betalain biosynthesis were analyzed during pitaya fruit development by qRT-PCR (Figure S2). cDNA and amino acid sequences of *H. monacanthus* and *H. undatus* were provided in the Supplementary Text S1. Amino acid sequence similarities between *H. monacanthus* and *H. undatus* were shown in Supplementary Table S4. As shown in Figure 1A, the pulp and peel color of ‘Guanhuahong’ pitaya began to turn red at S2 stage and continuously accumulated towards fully red, while the pulp color of ‘Guanhuabai’ pitaya did not change during fruit maturation. The expression levels of *CYP76AD1* (*HU03G00480.1*, belonging to CYP76AD1-α) in pigmented tissues were higher than those of the white ones throughout fruit developmental stages (Figure 1B). Compared with ‘Guanhuabai’ pitaya, the expression of *CYP76AD1* in ‘Guanhuahong’ pitaya displayed a sharp upward trend with pigmentation (Figure 1B). However, no close correlation was observed between the changes in genes belonging to the CYP76AD1-β or CYP76AD1-γ clade and the alterations of pigmentation. L-DOPA 4,5-dioxygenase in betalain-producing plants is encoded by *DODA* gene. The expression patterns of two *DODAα* genes (*HU03G01342.1* and *HU08G01572.1*) were correlated with pigment accumulation: increasing during red pigmentation, while constantly low in white pulps. Expression levels of *HU03G01342.1* were higher than *HU08G01572.1* (Figure 1B). According to the reported betalain-related genes, *GTs* genes were categorized into B5GT, B6GT, cDOPA5GT, UGT79B6 and UGT7B30 (Figure 2C). The expression levels of *cDOPA5GT* (*HU07G00240.1*) increased gradually with betalain accumulation (Figure 1B). The expression patterns of *CYP76AD1* (*HU03G00480.1*), *DODAα* (*HU03G01342.1* and *HU08G01572.1*) and *cDOPA5GT* (*HU07G00240.1*) were consistent with betalain accumulation during pitaya fruit development (Figure 1B), suggesting that they are possibly involved in betalain biosynthesis in pitaya.

### 3.5. Subcellular Localizations

**Figure 4:**
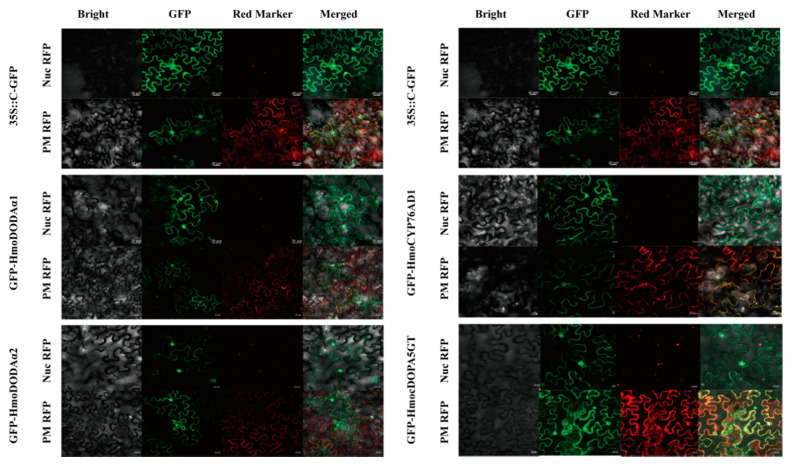
Subcellular localization of *HmoDODAα1*, *HmoDODAα2*, *HmoCYP76AD1* and *HmocDOPA5GT*. Every GFP construct and nucleolus-localized RFP marker or dual nucleus and cytoplasm RFP marker were co-transformed to leaf of *N. benthamiana*. Nuc RFP, Nucleolus-localized RFP marker (pAN95-At1g22590); PM RFP, Plasma membrane RFP marker (pAN-At5g19750); GFP-Gene, GFP tagged at the C-terminus of the gene; Bar = 20 µm.

Subcellular localizations of HmoCYP76AD1, HmoDODAα1, HmoDODAα2 and HmocDOPA5GT were validated using the GFP tag. As shown in Figure 4, the HmoCYP76AD1-GFP, HmoDODAα1-GFP, HmoDODAα2-GFP and HmocDOPA5GT-GFP were localized to both the cytoplasm and nucleus in terms of stronger green fluorescent signals. These results are in accordance with previously reported studies [52]. The dual localization signals were coincided with those of the PM marker RFP and the nuclear signal matched with that of the nucleolar marker RFP when the fusion protein was co-expressed with the markers.

### 3.6. VIGS Analyses of Candidate Genes

**Figure 5:**
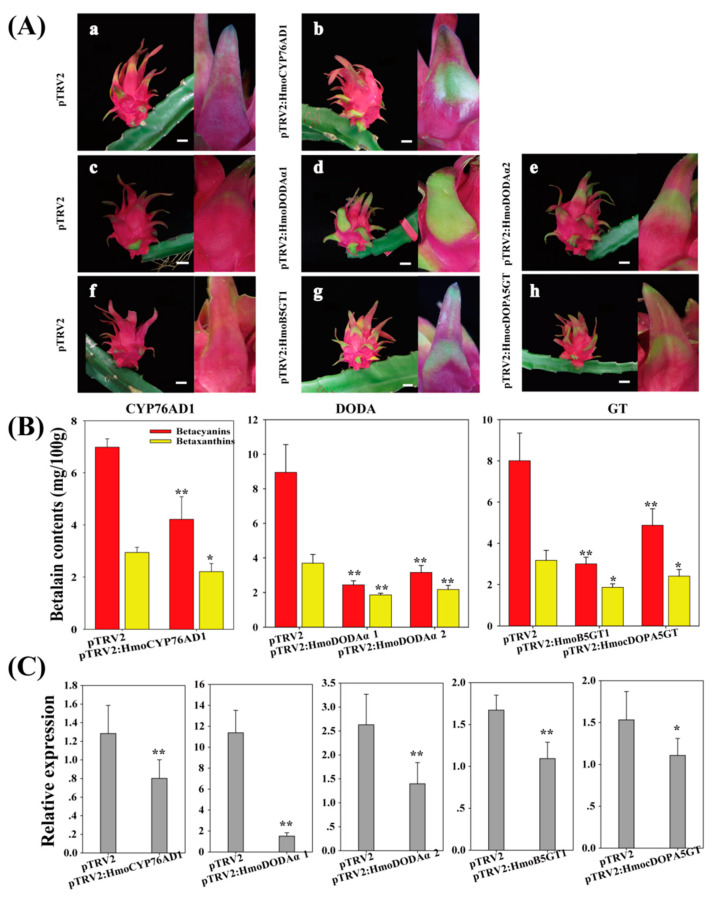
Virus-induced gene silencing (VIGS) phenotypes in pitaya. (**A**) Pitaya fruit (**left**) and ‘scale’ (**right**) at 14 d after inoculation. (**a**,**c**,**f**) TRV1/TRV2 empty vector–inoculated; (**b**) TRV1/TRV2-HmoCYP76AD1–inoculated; (**d**) TRV1/TRV2-HmoDODAα1–inoculated; (**e**) TRV1/TRV2-HmoDODAα2–inoculated; (**g**) TRV1/TRV2-HmoB5GT1–inoculated; (**h**) TRV1/TRV2-HmocDOPA5GT–inoculated. (**B**) Betalain contents of inoculation pitaya scales; (**C**) Gene expression of inoculation pitaya scale. **: *p* < 0.01, *: *p* < 0.05, Scale bars = 2 cm.

Gene silencing assay was performed to further elucidate the functions of *HmoCYP76AD1*, *HmoDODAα1*, *HmoDODAα2* and *HmocDOPA5GT* genes. Silencing *HmoB5GT1* resulted in the loss of betalain accumulation in red scales of pitaya [39]. Therefore, pTRV2 and pTRV2-HmoB5GT1 were separately infiltrated pitaya scales and served as the negative and positive controls. Silencing *HmoCYP76AD1*, *HmoDODAα1*, *HmoDODAα2* or *HmocDOPA5GT* resulted in the loss of betalain accumulation, which was evidenced by green scales (Figure 5A). Significant differences in contents of betacyanins and betaxanthins were detected in the gene-silenced tissues (Figure 5B). Results from qRT-PCR confirmed that *HmoCYP76AD1*, *HmoDODAα1*, *HmoDODAα2* and *HmocDOPA5GT* were silenced (Figure 5C).

### 3.7. Overexpression of HmoDODAα1 and HmoDODAα2 in Arabidopsis and Yeast

**Figure 6:**
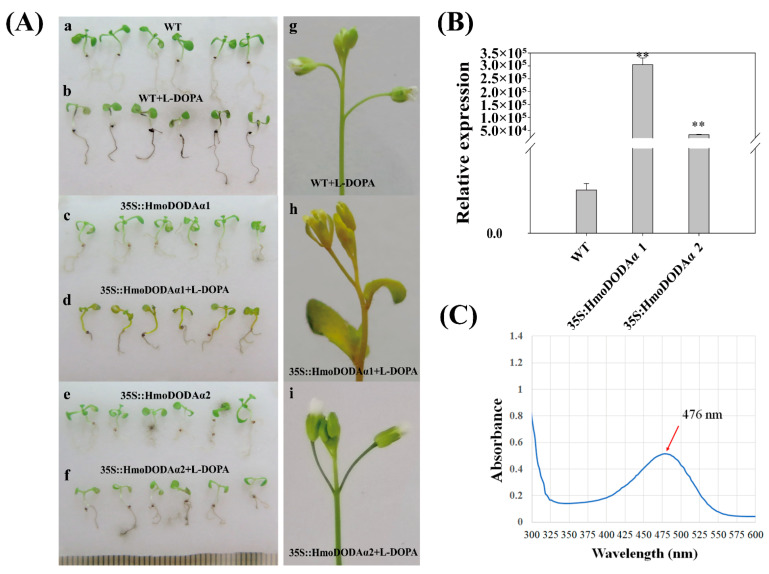
Genotypes of transgenic *A. thaliana* with *HmoDODAα1* and *HmoDODAα2*. (**A**) 35S::HmoDODAα1 and 35S::HmoDODAα2 seedlings were grown on moistened filter disks either without (**a**,**c**,**e**) or with (**b**,**d**,**f**) 10 mM L-DOPA. (**g**–**i**) The flowers of wild type and transformed with 35S::HmoDODAα1 and 35S::HmoDODAα2 feeding with L-DOPA. (**B**) Expression levels of *HmoDODAs* in transgenic *A. thaliana* seedlings. **: *p* < 0.01. (**C**) Maximum absorption of the pigment.

**Figure 7:**
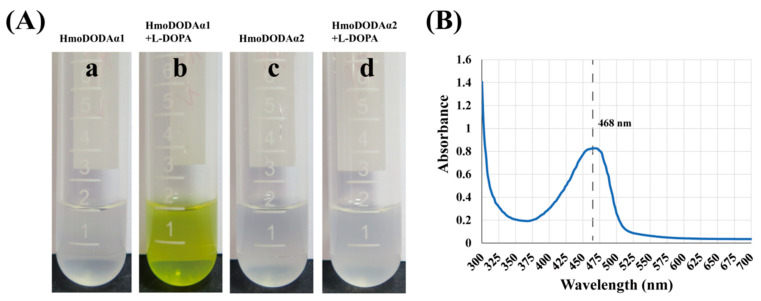
Expression of *HmoDODAα1* and *HmoDODAα2* in yeast. (**A**) Visual appearance of yeast strains after expression of *HmoDODAα1* and *HmoDODAα2*. The yeast strains only harboring *HmoDODAα1* or *HmoDODAα2*. (**a**) Yeast expressing *HmoDODAα1* without L-DOPA. (**b**) Yeast expressing *HpDODAα1* with L-DOPA. (**c**) Yeast expressing *HmoDODAα2* without L-DOPA. (**d**) Yeast expressing *HmoDODAα2* with L-DOPA. (**B**) Maximum absorption of the pigment.

To further confirm the role of *HmoDODAα1* and *HmoDODAα2*, we generated transgenic *A. thalianas* plants constitutively expressing *HmoDODAα1* and *HmoDODAα2* driven by the 35S promoter (Figure 6). No pigmentation was observed in the wild-type and transgenic lines harboring HmoDODAα1/2 without L-DOPA (Figure 6A(a,c,e)). Yellow pigmentation was produced in the 35S::HmoDODAα1 transgenic T_3_ generation seedlings and inflorescences with the addition of L-DOPA (Figure 6A(d,h)). No yellow pigment was detected in the 35S::moDODAα2 transgenic lines after feeding L-DOPA (Figure 6A(f,i)). The expression levels of *HmoDODAα1* and *HmoDODAα2* in the transgenic lines were significantly higher than those of wild types (Figure 6B). Similar results were obtained in yeast (Figure 7). The expression of *HmoDODAα1* in yeast supplemented with L-DOPA resulted in yellow cultures (Figure 7A(b)). The yellow pigments had a peak absorption at about 468 nm wavelength (Figure 6C and Figure 7B) which was the typical characteristic of betaxanthins [53]. Compared to *HmoDODAα1*, no visible pigment production in yeast was observed by expressing of *HmoDODAα2* with the presence of L-DOPA (Figure 7A(d)).

### 3.8. Co-Expression of HmoCYP76AD1, HmoDODAα1/α2 and HmoB5GT1/HmocDOPA5GT in N. benthamiana

**Figure 8:**
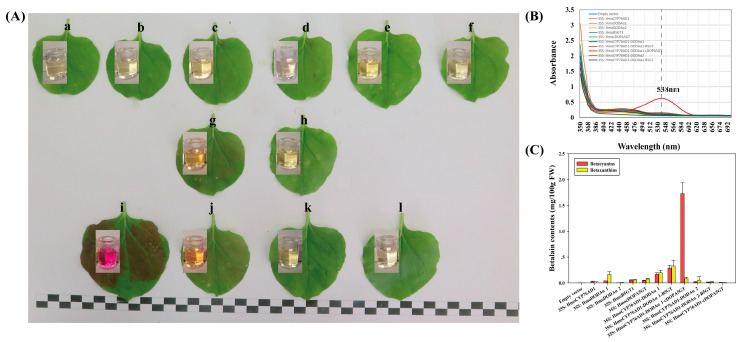
Transient expression of candidate genes in *N. benthamiana*. (**A**) Phenotype and extract of *N. benthamiana* leaves after infected. (**a**–**f**) Empty vector, *HmoCYP76AD1*, *HmoDODAα1*, *HmoDODAα2*, *HmoB5GT1*, *HmocDOPA5GT*; (**g**,**h**) *HmoCYP76AD1*-*HmoDODAα1*, *HmoCYP76AD1*-*HmoDODAα2*; (**i**–**l**) *HmoCYP76AD1*-*HmoDODAα1*-*HmocDOPA5GT*, *HmoCYP76AD1*-*HmoDODAα1*-*HmoB5GT1*, *HmoCYP76AD1*-*HmoDODAα2*-*HmoB5GT1*, *HmoCYP76AD1*-*HmoDODAα2*-*HmocDOPA5GT*; (**B**) Maximum absorption of the pigment. (**C**) Betalain contents in *N. benthamiana* leaves. Scale bars = 1 cm.

*HmoCYP76AD1*, *HmoDODAα1/α2*, and *HmoB5GT1*/*HmocDOPA5GT* were transiently overexpressed in *N. benthamiana* leaves. No visible pigmentation was detected in the infiltrated area of *N. benthamiana* leaves when single *HmoCYP76AD1*, *HmoDODAα1*, *HmoDODAα2*, *HmoB5GT1* or *HmocDOPA5GT* was overexpressed (Figure 8A(a–f)). Co-infiltration of *A. tumefaciens* containing *HmoCYP76AD1* and *HmoDODAα1* resulted in light red pigmentation (Figure 8A(g)). Light red pigment was also observed when *HmoCYP76AD1, HmoDODAα1* and *HmoB5GT* were co-expressed in *N. benthamiana* (Figure 8A(j)). Dark red pigmentation was only observed in the infiltrated areas after co-expressing *HmoCYP76AD1, HmoDODAα1* and *HmocDOPA5GT* (Figure 8A(i)). However, no pigmentation was detected in the infiltrated areas when *HmoCYP76AD1*-*DODAα2*-*cDOPA5GT* or *HmoCYP76AD1*-*DODAα2*-*B5GT1* were co-expressed (Figure 8A(k,l)). Moreover, there was no pigment accumulation in *N. benthamiana* leaves when *HmoDODAα1* or *HmoDODAα2* was co-expressed with *HmocDOPA5GT* or *HmoB5GT1* without *HmoCYP76AD1* (Figure S3). The red pigments had a peak absorption at 538 nm wavelength (Figure 8B) which was the typical characteristic of betacyanins. Co-expression of *HmoCYP76AD1*-*DODAα1*-*cDOPA5GT* resulted in the highest abundance of betacyanins in the infiltrated areas compared to very low levels of betacyanin after co-expressions of the other combinations such as *HmoCYP76AD1*-*DODAα1* and *HmoCYP76AD1*-*DODAα2*-*B5GT1* (Figure 8C). In addition, the expression levels of these genes significantly increased in the infiltrated areas (Figure S4).

### 3.9. Recombinant Expression of HmoCYP76AD1, HmoDODAα1 and HmocDOPA5GT in Yeast

**Figure 9:**
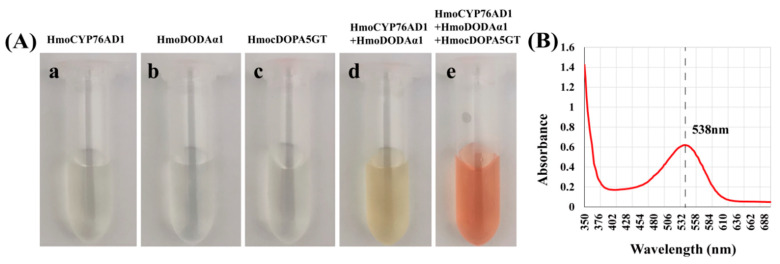
Recombinant expression of *HmoCYP76AD1*, *HmoDODAα1* and *HmocDOPA5GT* in yeast. (**A**) Visual appearance of yeast medium from yeast strains. (**a**–**c**) The yeast strains only harboring *HmoCYP76AD1*, *HmoDODAα1* or *HmocDOPA5GT*. (**d**) Co-expressing *HmoCYP76AD1* and *HmoDODAα1*. (**e**) Co-expressing *HmoCYP76AD1*, *HmoDODAα1* and *HmocDOPA5GT.* (**B**) Maximum absorption of the pigment.

Recombinant expression assay was performed to further confirm the function of *HmoCYP76AD1*, *HmoDODAα1* and *HmocDOPA5GT* genes. No pigment was detected when *HmoCYP76AD1*, *HmoDODAα1* or *HmocDOPA5GT* was separately expressed in yeast (Figure 9A(a–c)). Yellow pigments were detected in the yeast cultures when *HmoCYP76AD1* was co-expressed with *HmoDODAα1* (Figure 9A(d)). Red pigments were observed in the yeast cultures containing pYES2-HmoDODAα1 and pESC-HmoCYP76AD1-HmocDOPA5GT vectors (Figure 9A(e)). The peak absorption of red pigments was detected at 538 nm wavelength (Figure 9B) suggesting that betacyanins are produced with co-expression of *HmoCYP76AD1*, *HmoDODAα1* and *HmocDOPA5GT* in yeast.

## 4. Discussion

### 4.1. Identification of Genes Involved in Betalain Biosynthesis

To date, key genes involved in betalain biosynthesis remain to be fully elucidated. It has been reported that CYP76AD, DODA and GT were responsible for betalain biosynthesis [12,13]. However, genome-wide analyses of these genes in betalain biosynthesis are not available in betalain-producing plant species. In the present study, candidate pitaya betalain biosynthesis-related genes were identified and their distribution, structure, conservation, clustering and expression were analyzed. Based on phylogenetic analysis, 15 CYP76AD-encoding genes from pitaya were classified into three categories: CYP76AD-α, CYP76AD-β and CYP76AD1-γ (Figure 2A), which is consistent with the results from Brockington et al. (2015) and Chen et al. (2021) [14,43]. Three *DODA* genes including one homologue from DODAβ lineage and two paralogues from DODAα lineage have been isolated from betalain-pigmented lineages of Caryophyllales [14,22,43]. In the pitaya genome, three *DODA* genes assigned to DODAα and DODAβ were obtained from pitaya (Figure 2B) [43]. *DODAα1* (*HU03G01342.1*) and *DODAα2* (*HU08G01571.1*) were clustered in the DODAα clade while *DODAβ* (*HU08G01572.1*) belonged to the DODAβ clade. *DODAα1* and *CYP76AD1* are involved in betalain biosynthesis which have been identified in *B. vulgaris* [13], *C. quinoa* and *A. hypochondriacus* [22]. In this study, *CYP76AD1* (*HU03G00480.1*) and *DODAα1* (*HU03G01342.1*) were localized in Chr 3. These results suggested that *H. undatus*, *B. vulgaris*, *C. quinoa* and *A. hypochondriacus* may share one putative origin of betalain pigmentation. The diversity of betacyanins in species depends on different GTs [16,25,28,29,30]. Chen et al. (2021) found that GTs involved in betalain biosynthesis were divided into three classes: cDOPA5GT, B5GT and B6GT [43]. In this study, GTs were divided into five groups: B5GT, B6GT, cDOPA5GT, UGT79B6 and UGT7B30 (Figure 2C).

The expression levels of betalain biosynthesis-related genes were increasing with betalain accumulation [13,15,16,18,19,22,43]. Candidate genes such as CYP76AD, DODA and GTs involved in betalain biosynthesis were isolated from pitaya [35,36,39,40,43,54]. In this study, *CYP76AD1* (*HU03G00480.1*), *DODAα1* (*HU03G01342.1*), *DODAα2* (*HU08G01571.1*) and *cDOPA5GT* (*HU07G00240.1*) were preferentially expressed in pigmented tissues (Figure 1B). This result was consistent with the results from Chen et al. (2021) [43]. Compared with *B. vulgaris* [18], no candidate gene that can catalyze the first hydroxylation step in redundancy with *CYP76AD1* was obtained in pitaya (Figure 1B). Therefore, we supposed that *CYP76AD1* (*HU03G00480.1*) from pitaya belonging to the CYP76ADα clade maybe a unique gene with both tyrosine hydroxylase and oxidase activities responsible for L-DOPA and cyclo-DOPA formation. The expression pattern of *cDOPA5GT* was in accordance with the accumulation of betalains (Figure 1B) suggesting that *cDOPA5GT* maybe a potential gene encoding GT in pitaya.

### 4.2. HmoCYP76AD1 Catalyzes Tyrosine to L-DOPA and to Cyclo-DOPA

The functions of CYP76ADα and CYP76ADβ clade homologues have been previously reported in betalain-producing plant species [13,15,16,17,18,19]. In *B. vulgaris*, silencing *CYP76AD1* alone blocked the production of betacyanins but not betaxanthins, suggesting that *CYP76AD1* and *CYP76AD6* catalyze the formation of L-DOPA from tyrosine. However, only *CYP76AD1* can catalyze the conversion of L-DOPA to cyclo-DOPA [18]. In this study, *HmoCYP76AD1* belonging to the CYP76ADα clade was identified in pitaya. Silencing *HmoCYP76AD1* resulted in the reduced accumulation of betacyanins and betaxanthins (Figure 5), suggesting that *HmoCYP76AD1* was the enzyme that contributes to L-DOPA formation and the subsequent conversion to cyclo-DOPA in pitaya. Betaxanthins were detected in yeast cultures after co-expression of *HmoCYP76AD1* and *HmoDODAα1* genes without feeding L-DOPA (Figure 9), suggesting that *HmoCYP76AD1* has tyrosine hydroxylase activity for converting tyrosine to L-DOPA. Similar findings were also reported in other studies [17,18,19]. Red pigmentation was detected after expression of *HmoCYP76AD1*, *HmoDODAα1* and *HmocDOPA5GT* in *N. benthamiana* and yeast culture (Figure 8 and Figure 9), indicating that *HmoCYP76AD1* catalyzes two succeeding steps in the pitaya betalain biosynthesis pathway to produce cyclo-DOPA. Similar results were also found in other betalain-producing plant species such as *B. vulgaris*, *C. quinoa*, *A. tricolor* and *M. jalapa* [13,16,17,18,22,23].

### 4.3. HmoDODAα1 Exhibits a High Degree of L-DOPA 4,5-Dioxygenase Activity

L-DOPA 4,5-dioxgenase responsible for synthesis of betalamic acid is considered to be an important betalain-producing enzyme. Betalain-pigmented species have multiple paralogues of *DODAα* genes, but only one of them in each species encodes the protein with a high degree of L-DOPA 4,5-dioxygenase activity [22,24]. Loss of betalains was observed when the gene encoding DODA was silenced in *B. vulgaris* [13]. In this study, two candidate genes (*HmoDODAα1* and *HmoDODAα2*) were obtained from *H.* *monacanthus*. Silencing *HmoDODAα1* and *HmoDODAα2* in *H.* *monacanthus* resulted in betalain reduction (Figure 5A,C), suggesting that *HmoDODAα1* and *HmoDODAα2* encode proteins with L-DOPA 4,5-dioxygenase activity. Overexpression of *HmoDODAα1* and *HmoDODAα2* in *A. thaliana*, *N. benthamiana* and yeast revealed that only *HmoDODAα1* could synthesize betalamic acid (Figure 6, Figure 7, Figure 8 and Figure 9). Similar findings were also reported in *B. vulgaris*, *C. quinoa* and *A. hypochondriacus* [16,22]. Silencing *DODAα2* caused slight decolouration, but *DODAα2* is not active in betalain accumulation when *DODAα2* was overexpressed in *N. benthamiana* and yeast. The reason is that the *DODAα2* may not have sufficient levels to influence pigment accumulation [22]. Further experiments such as co-silencing *DODAα1* and *DODAα2* are necessary to elucidate its role in betalain biosynthesis. These results suggested that *HmoDODAα1* encodes a protein with a high degree of L-DOPA 4,5-dioxygenase activity involved in pitaya betalain biosynthesis.

### 4.4. Reconstruction of Heterogenous Production of Betalain Biosynthesis Pathway

Betacyanins are formed through glycosylation by GT [11,12]. In *D. bellidiformis*, glycosylation on betanidin is directly catalyzed by B5GT and B6GT [12,28,29]. In *M. jalapa*, *A. hypochondriacus* and *C. quinoa*, glycosylation by cDOPA 5-O-GT (cDOPA-5GT) is involved in the betalain biosynthesis [16,25,30]. In our previous study, we found that *HmoB5GT1* was involved in the betalain biosynthesis in pitaya [39]. In the present study, low production of betacyanins was observed when *HmoCYP76AD1*, *HmoDODAα1* and *HmoB5GT1* were co-expressed in *N. benthamiana* (Figure 8). In contrast, the co-expression of *HmoCYP76AD1*, *HmoDODAα1* and *HmocDOPA5GT* resulted in high levels of betalain pigments (Figure 8 and Figure 9). These results suggested that *HmocDOPA5GT* plays an important role in glycosylation during betalain biosynthesis in *H.* *monacanthus*.

## 5. Conclusions

In summary, this study was the first attempt to identify and validate the key genes involved in pitaya betalain biosynthesis based on the whole genome sequence of *H. undatus*. Fifteen CYP76AD-encoding genes, three DODA-encoding genes and 35 GTs-encoding genes were obtained from the *H. undatus* genome. Four candidate genes i.e., *HmoCYP76AD1*, *HmoDODAα1*, *HmoDODAα2* and *HmocDOPA5GT* showed upward expression trends, which was consistent with betalain accumulation during pitaya fruit maturation. Furthermore, *HmoDODAα1* displayed a high degree of L-DOPA 4,5-dioxygenase activity to produce betalamic acid. Co-expression of *HmoCYP76AD1*, *HmoDODAα1* and *HmocDOPA5GT* in *N. benthamiana* and yeast resulted in high abundances of betalain pigments. These results suggested that *HmoCYP76AD1*, *HmoDODAα1*, and *HmocDOPA5GT* play key roles in pitaya betalain biosynthesis. The results of the present study not only shed light on the evolution of betalain biosynthesis pathway in Caryophyllales, but also provide novel genes to genetically improve the fruit quality of pitaya.

## Data Availability

Data is contained within the article and supplementary materials.

## Supplementary Materials

The following are available online at https://www.mdpi.com/article/10.3390/genes12121858/s1, Figure S1: Gene structures of 53 genes. Figure S2: Expression analyses of candidate genes in different fruit developmental stages of pitayas by RT-qPCR. Figure S3: Penotype of *N. benthamiana* leaves after infected. Figure S4: Gene expression of *N. benthamiana* leaves after infected. Table S1: Th e accession numbers of genes in this study. Table S2: All primers used in this study. Table S3: 53 candidate genes in pitaya. Table S4: Amino acid sequence similarities between *H. monacanthus* and *H. undatus*. Text S1: cDNA and amino acid sequences of genes for qPCR in pitaya. Text S2: cDNA sequences of genes for VIGS in red pitaya. Text S3: Vectors used in this study.
