# Supplementary material for: A Genome-Wide Identification Study Reveals That HmoCYP76AD1, HmoDODAα1 and HmocDOPA5GT Involved in Betalain Biosynthesis in Hylocereus
Source: Genes (Basel). 2021 Nov 23;12(12):1858. doi: 10.3390/genes12121858 (PMC8702118; doi:10.3390/genes12121858)
Supplement: Supplementary file 1 [file genes-12-01858-s001.zip › Supplementary Figure S2. Expression analyses of candidate genes in different fruit developmental stages of pitayas by RT-qPCR.pdf]

CYP76ADs

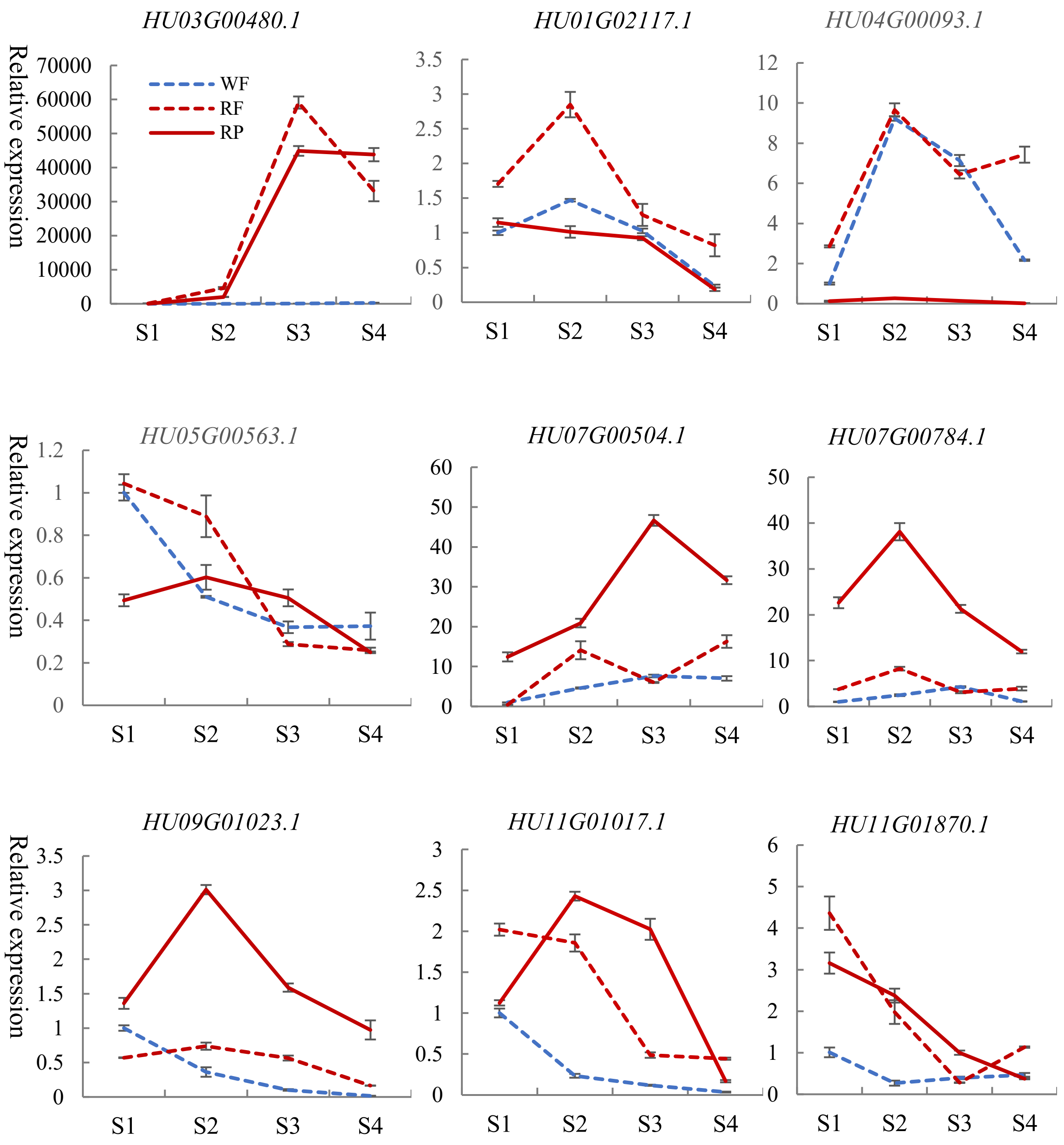

Figure S2-1. Expression patterns of betalain biosynthesis-related *CYP76ADs* during four fruit developmental stages of pitaya.

(RF) pulps of 'Guanhuahong' pitaya, (WF) pulps of 'Guanhuabai' pitaya, (RP) peels of 'Guanhuahong' pitaya.

DODAs

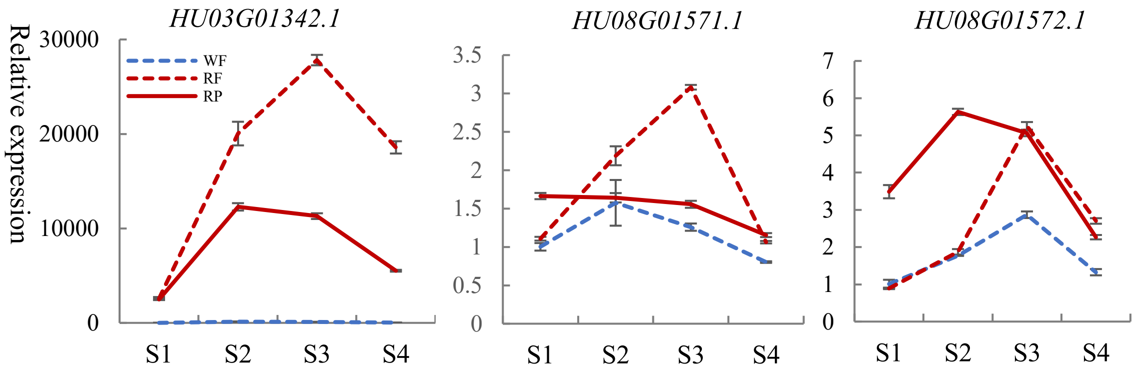

Figure S2-2. Expression patterns of betalain biosynthesis-related *DODAs* during four fruit developmental stages of pitaya.

(RF) pulps of ‘Guanhuahong’ pitaya, (WF) pulps of ‘Guanhuabai’ pitaya, (RP) peels of ‘Guanhuahong’ pitaya.

GTs

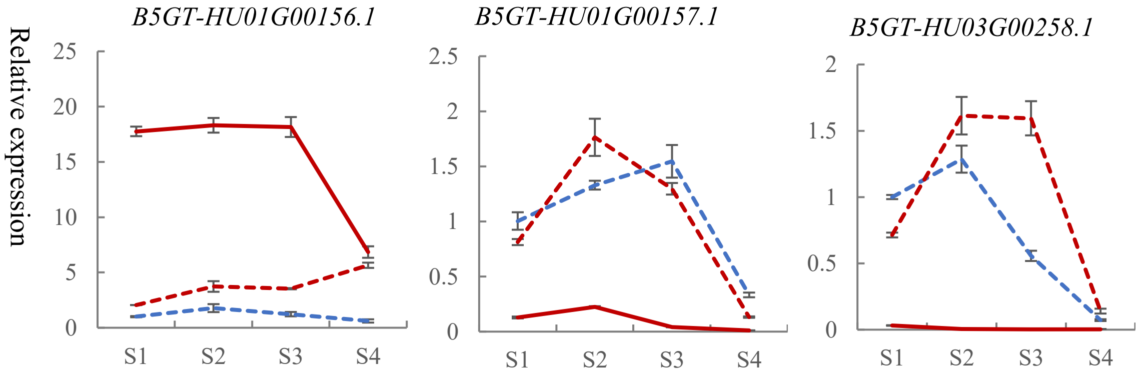

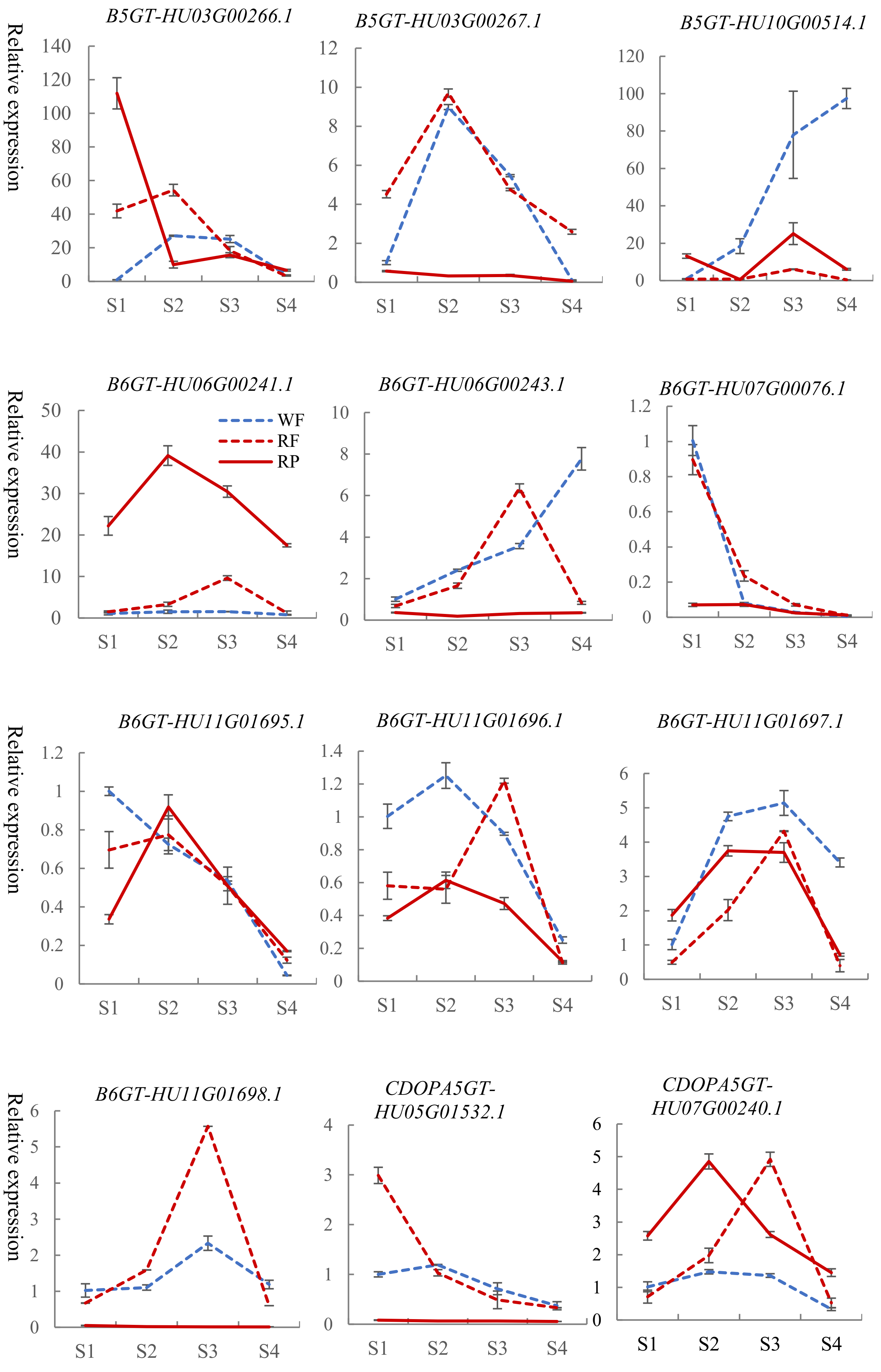

B5GT-HU03G00266.1
B5GT-HU03G00267.1
B5GT-HU10G00514.1
B6GT-HU06G00241.1
B6GT-HU06G00243.1
B6GT-HU07G00076.1
B6GT-HU11G01695.1
B6GT-HU11G01696.1
B6GT-HU11G01697.1
B6GT-HU11G01698.1
CDOPA5GT-HU05G01532.1
CDOPA5GT-HU07G00240.1
Relative expression
WF
RF
RP
S1
S2
S3
S4

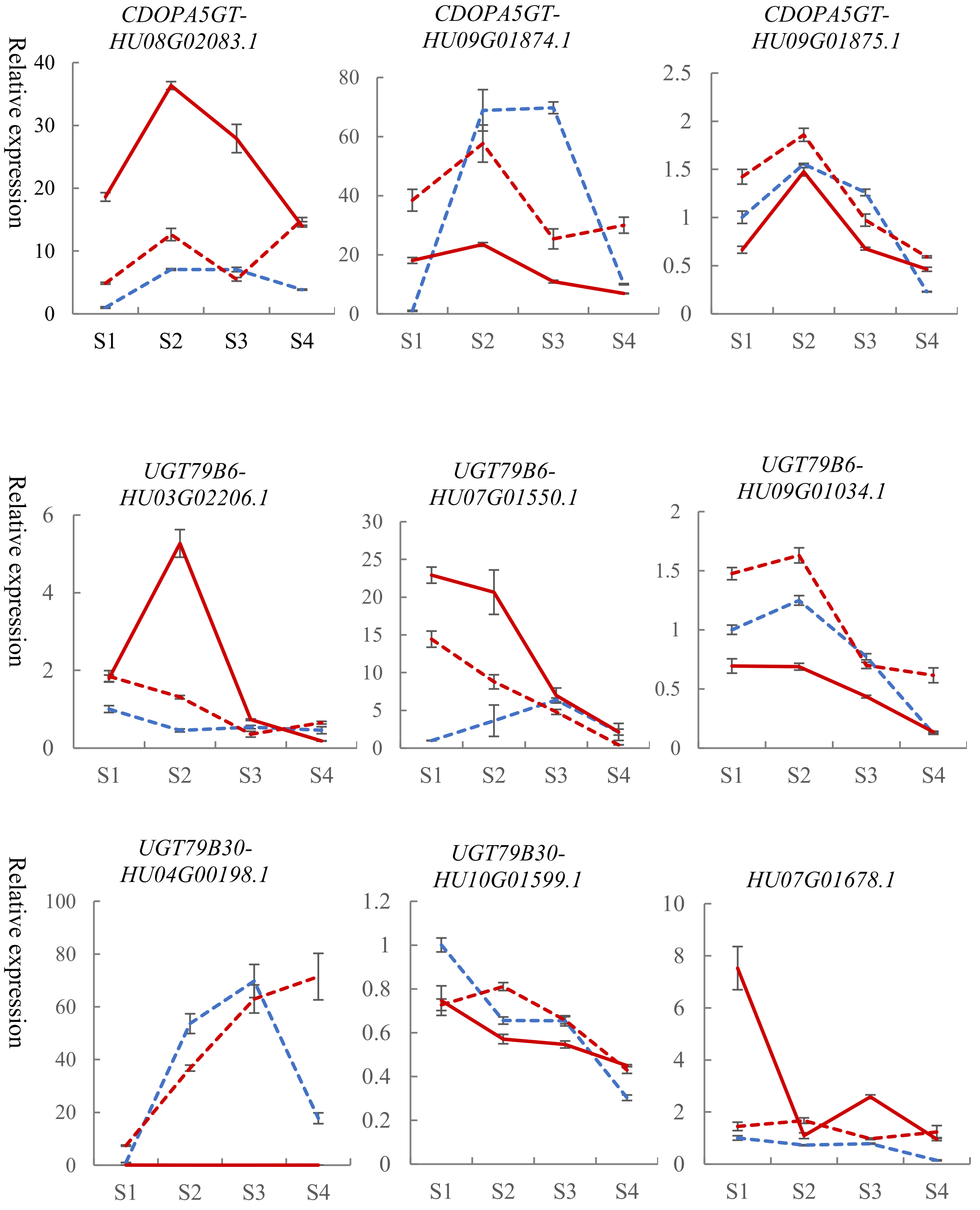

Figure S2-3. Expression patterns of betalain biosynthesis-related *GTs* during four fruit developmental stages of pitaya.

(RF) pulps of 'Guanhuahong' pitaya, (WF) pulps of 'Guanhuabai' pitaya, (RP) peels of 'Guanhuahong' pitaya.
