# Supplementary material for: A Genome-Wide Identification Study Reveals That HmoCYP76AD1, HmoDODAα1 and HmocDOPA5GT Involved in Betalain Biosynthesis in Hylocereus
Source: Genes (Basel). 2021 Nov 23;12(12):1858. doi: 10.3390/genes12121858 (PMC8702118; doi:10.3390/genes12121858)
Supplement: Supplementary file 1 [file genes-12-01858-s001.zip › Supplementary Figure S3. Phenotype of N. benthamiana leaves after infected.pdf]

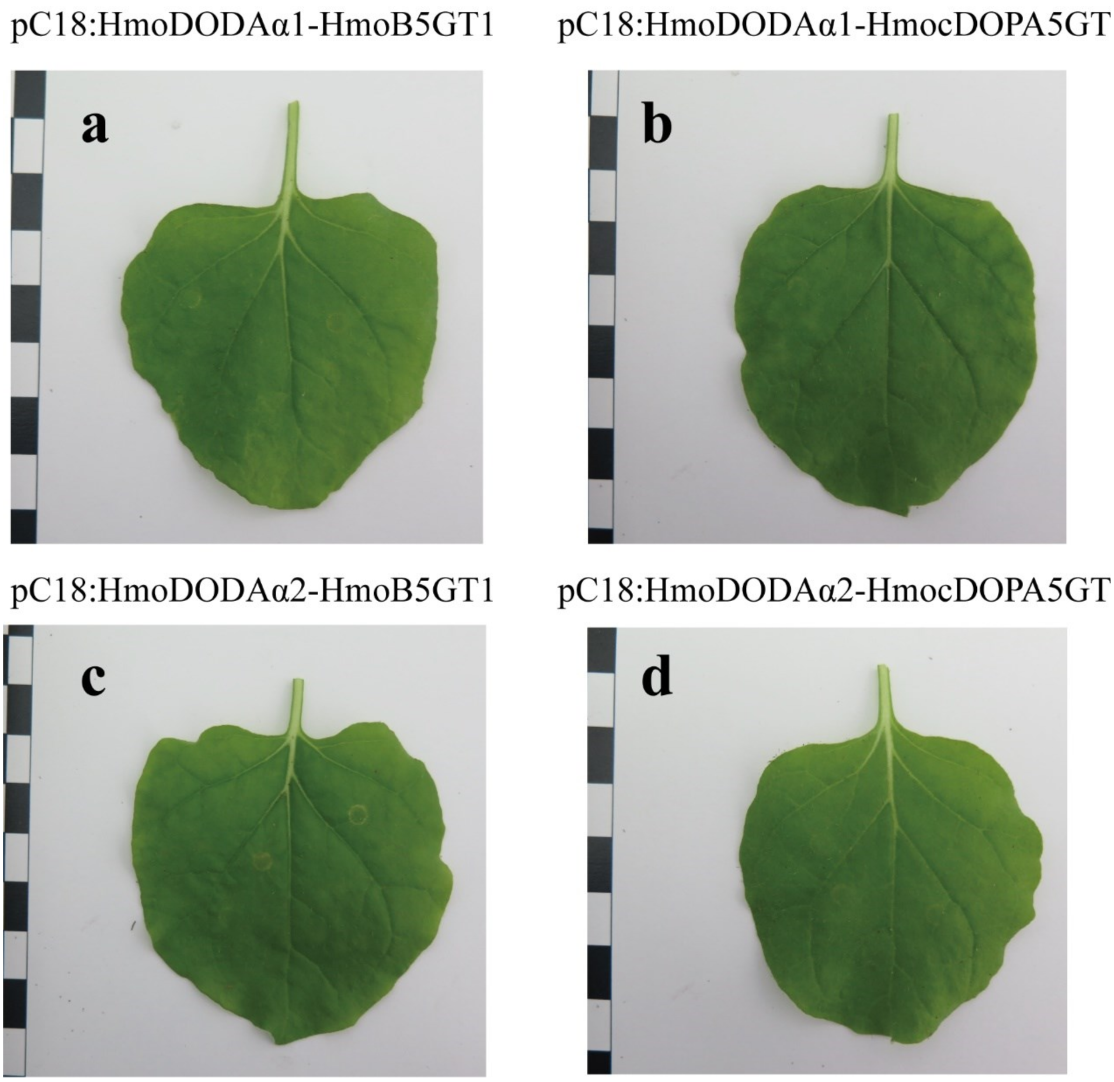

Figure S3. Phenotype of *N. benthamiana* leaves after infected.

(a), Co-expression of *HmoDODAα1*/*HmoB5GT1*; (b), Co-expression of *HmoDODAα1*/*HmocDOPA5GT*; (c), Co-expression of *HmoDODAα2*/*HmoB5GT1*; (D), Co-expression of *HmoDODAα1*/*HmocDOPA5GT*.
