# Supplementary material for: A Genome-Wide Identification Study Reveals That HmoCYP76AD1, HmoDODAα1 and HmocDOPA5GT Involved in Betalain Biosynthesis in Hylocereus
Source: Genes (Basel). 2021 Nov 23;12(12):1858. doi: 10.3390/genes12121858 (PMC8702118; doi:10.3390/genes12121858)
Supplement: Supplementary file 1 [file genes-12-01858-s001.zip › Supplementary Figure S4. Gene expression of N. benthamiana leaves after infected.pdf]

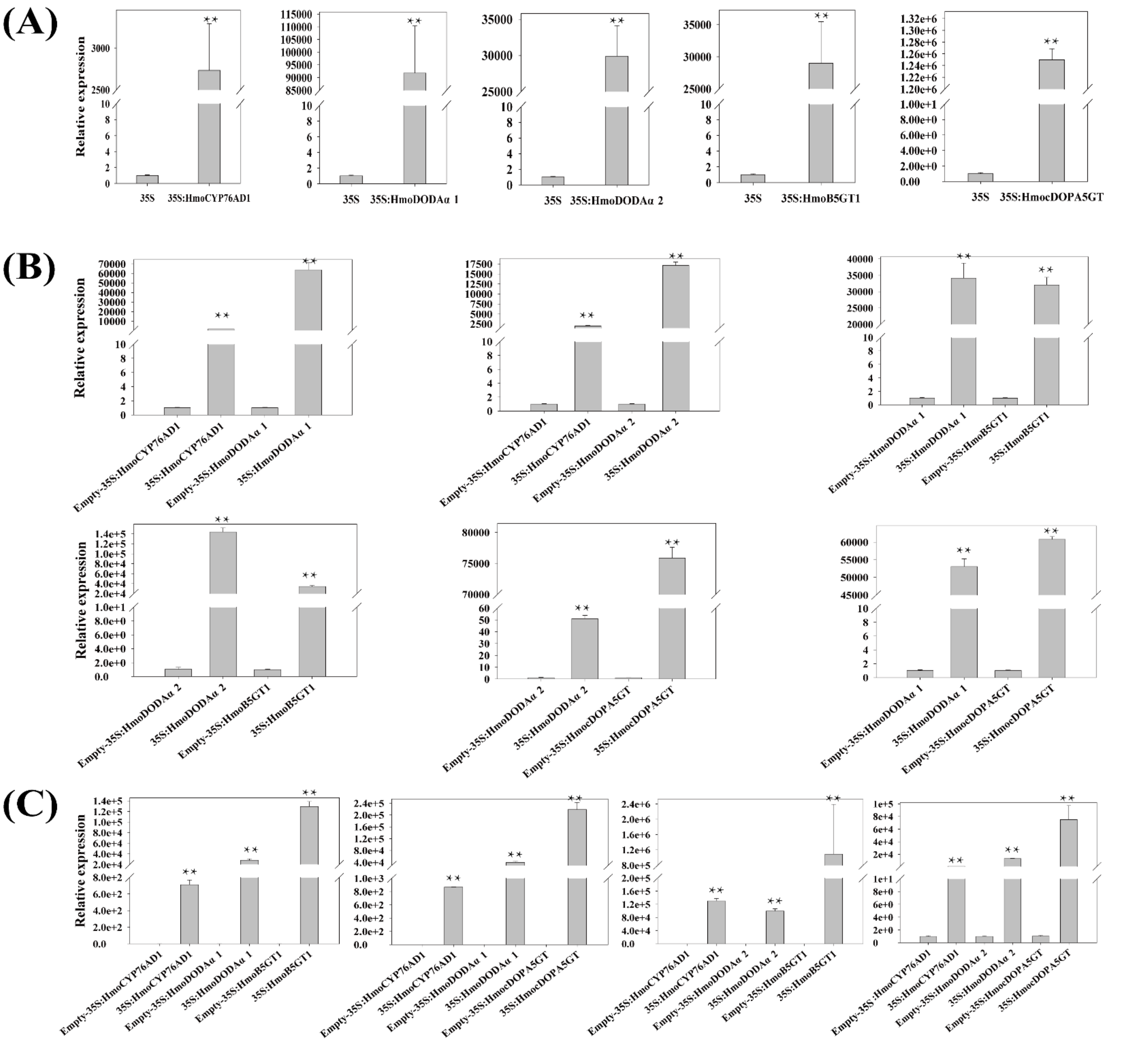

Figure S3. Gene expression of *N. benthamiana* leaves after infected.

(A), Single-gene expression of *HmoCYP76AD1*, *HmoDODAα1*, *HmoDODAα2*, *HmoB5GT1* or *HmocDOPA5GT*; (B), Co-expression of *HmoCYP76AD1/HmoDODAα1*, Co-expression of *HmoCYP76AD1/HmoDODAα2*, Co-expression of *HmoDODAα1/HmoB5GT1*, Co-expression of *HmoDODAα2/HmoB5GT1*, Co-expression of *HmoDODAα2/ HmocDOPA5GT*, Co-expression of *HmoDODAα1/HmocDOPA5GT*; (C), Co-expression of *HmoCYP76AD1/ HmoDODAα1/HmoB5GT1*, Co-expression of *HmoCYP76AD1/HmoDODAα2/HmoB5GT1*, Co-expression of *HmoCYP76AD1/HmoDODAα1/HmocDOPA5GT*, Co-expression of *HmoCYP76AD1/HmoDODAα2/HmocDOPA5GT*.
