# Supplementary material for: A Genome-Wide Identification Study Reveals That HmoCYP76AD1, HmoDODAα1 and HmocDOPA5GT Involved in Betalain Biosynthesis in Hylocereus
Source: Genes (Basel). 2021 Nov 23;12(12):1858. doi: 10.3390/genes12121858 (PMC8702118; doi:10.3390/genes12121858)
Supplement: Supplementary file 1 [file genes-12-01858-s001.zip › Supplementary Table S1. The accession numbers of genes in this study.pdf]

Table S1. The accession numbers of genes in the paper.

Species	Genes	GenBank accession No.
Beta vulgaris	*BvADHα*	AST12931.1
Beta vulgaris	*BvADHβ*	AST12926.1
Mirabilis jalapa	*MjADHα*	AMR60658.1
Nepenthes ventricosa×*Nepenthes alata*	*NaADHβ*	AST12937.1
Portulaca oleracea	*PoADHα*	AST12940.1
Rivina humilis	*RhADHα*	AST12939.1
Spinacia oleracea	*SoADHα*	AST12938.1
Spinacia oleracea	*SoADHβ*	AST12936.1
Amaranthus cruentus	*AcCYP76AD2*	AET43291.1
Amaranthus hybridus	*AhCYP76AD1-like*	AKI33932.1
Basella alba	*BaCYP76AD13*	AJD87469.1
Basella alba	*BaCYP76AD14*	AJD87470.1
Beta vulgaris	*BvCYP76AD5*	AJD87473.1
Beta vulgaris	*BvCYP76AD6*	ALS88208.1
Beta vulgaris	*BvCYP76AD1*	AQS22859.1
Beta vulgaris	*Bv_xkhk*	
Beta vulgaris	*Bv_sidm*	
Cleretum bellidiforme	*CbCYP76AD12*	AJD87468.1
Cleretum bellidiforme	*CbCYP76AD16*	AJD87472.1
Celosia cristata	*CcCYP76AD4*	AGI78466.1
Chenopodium quinoa	*CqCYP76AD1*	XP_021769302.1
Dianthus caryophyllus	*DcCYP76AD19*	AMA07825.1
Mirabilis jalapa	*MjCYP76*	ALT04745.1
Mirabilis jalapa	*MjCYP76AD3*	AET43292.1

Species	Genes	GenBank accession No.
Mirabilis jalapa	*MjCYP76AD7*	AJD87463.1
Mirabilis jalapa	*MjCYP76AD15*	AJD87471.1
Opuntia ficus	*OfCYP76AD8*	AJD87464.1
Opuntia ficus	*OfCYP76AD9*	AJD87465.1
Phytolacca americana	*PaCYP76AD10*	AJD87466.1
Phytolacca americana	*PaCYP76AD11*	AJD87467.1
Mollugo verticillata	*MvCYP76AD17*	AMA07822.1
Mollugo verticillata	*MvCYP76F84*	AMA07823.1
Carnegiea gigantea	*CgDODAα1*	MN136222
Carnegiea gigantea	*CgDODAα2*	MN136223
Carnegiea gigantea	*CgDODAβ*	MN136224
Chenopodium quinoa	*CqDODA1*	XM_021913611.1
Beta vulgaris	*BvDODAα1*	HQ656027.1
Beta vulgaris	*BvDODAα2*	MN153195
Beta vulgaris	*BvDODAα3*	MN153196
Beta vulgaris	*BvDODAα4*	MN136228
Beta vulgaris	*BvDODAα5*	MN153197
Beta vulgaris	*BvDODAβ*	MN153198
Mesembryanthemum crystallinum	*McDODAα1*	MN136225
Mesembryanthemum crystallinum	*McDODAα2*	MN136226
Mesembryanthemum crystallinum	*McDODAβ*	MN136227
Stegnosperma halimifolium	*ShDODAα1*	MN136219
Stegnosperma halimifolium	*ShDODAα2*	MN136220
Stegnosperma halimifolium	*ShDODAβ*	MN136221
Macarthuria australis	*MaDODAα*	MN153205

Species	Genes	GenBank accession No.
Limeum aethiopicum	*LaDODA*	MN153203
Kewa caespitosa	*KcDODAα1*	MN153209
Kewa caespitosa	*KcDODAα2*	MN153210
Polycarpon tetraphyllum	*PtDODA*	MN153211
Cardionema ramosissimum	*CrDODA*	MN153200
Spergularia marina	*SmDODAα*	MN153204
Telephium imperati	*TiDODA*	MN153212
Parakeelya mirabilis	*PmDOD*	KF747352.1
Parakeelya mirabilis	*PmDOD-like*	KF747353.1
Ptilotus hybrid	*PhybDOD-like*	KF747354.1
Mirabilis jalapa	*MjDOD*	AB435372.1
Portulaca grandiflora	*PgDODA*	AJ580598.1
Amaranthus hypochondriacus	*AhUGT79B30-like2*	AH018627-RA
Amaranthus hypochondriacus	*AhUGT79B30-like3*	AH018628-RA
Amaranthus hypochondriacus	*AhUGT79B30-like*	AH018629-RA
Amaranthus hypochondriacus	*AhB5GT*	AHV78225.1
Amaranthus hypochondriacus	*AhCDOPA5GT*	AHV78226.1
Amaranthus tricolor	*AmaB5GT*	AJY59053.1
Amaranthus tricolor	*AmaB6GT*	AJY59055.1
Amaranthus tricolor	*AmaCDOPA5GT*	AJY59054.1
Beta vulgaris	*Bv3GGT-like1*	XP_010695817
Beta vulgaris	*Bv3GGT-like2*	XP_010686377
Beta vulgaris	*BvUGT97B30-like*	XP_010666234
Beta vulgaris	*BvUGT97B6-like1*	XP_010674067
Beta vulgaris	*BvUGT97B6-like2*	XP_010675464

Species	Genes	GenBank accession No.
Beta vulgaris	*BvGT partial*	AAO88911.1
Beta vulgaris	*BvUGT71F1*	AAS94329.1
Beta vulgaris	*BvUGT73A4*	AAS94330.1
Beta vulgaris	*BvUGT92A1-like*	XP_10681653.1
Bougainvillea spectabilis	*BsCDOPA5GT*	AIS20905.1
Cleretum bellidiforme	*CbB5GT*	CAB56231.1
Cleretum bellidiforme	*CbB6GT*	AF374004.1
Celosia cristata	*CcDOPA5GT*	BAD91804.1
Chenopodium quinoa	*CqUGT79B6-like1*	XP_021719267
Chenopodium quinoa	*Cq3GGT-like1*	XP_021726554
Chenopodium quinoa	*CqUGT79B2-like*	XP_021731181
Chenopodium quinoa	*CqUGT79B30-like1*	XP_021735671
Chenopodium quinoa	*Cq3GGT-like2*	XP_021735839
Chenopodium quinoa	*CqUGT79B30-like2*	XP_021735840
Chenopodium quinoa	*CqAmaSy2*	XP_021735841
Chenopodium quinoa	*CqUGT79B6-like2*	XP_021747968
Chenopodium quinoa	*CqAmaSy1*	XP_021754077
Chenopodium quinoa	*CqUGT79B30-like5*	XP_021758620
Chenopodium quinoa	*Cq3GGT-like3*	XP_021766006
Chenopodium quinoa	*CqUGT79B6-like3*	XP_021773738
Chenopodium quinoa	*CqUGT92A1-like*	XP_021748306.1
Mirabilis jalapa	*MjCDOPA5GT*	BAD91803.1
Opuntia ficus-indica	*OfB5GT putative*	ABW79879.1
Phytolacca americana	*PaGT3*	BAG71127.1
Phytolacca americana	*PaGT2*	BAG71125.1
