# Supplementary material for: A Genome-Wide Identification Study Reveals That HmoCYP76AD1, HmoDODAα1 and HmocDOPA5GT Involved in Betalain Biosynthesis in Hylocereus
Source: Genes (Basel). 2021 Nov 23;12(12):1858. doi: 10.3390/genes12121858 (PMC8702118; doi:10.3390/genes12121858)
Supplement: Supplementary file 1 [file genes-12-01858-s001.zip › Supplementary Table S2. All primers used in this study.pdf]

Table S2. All primers used in this study.

Primer names	Primer sequences (5'-3')	Assays
Actin(1) F	AAAGGCTAACAGGGAGAAAA	RT-qPCR
Actin(1) R	GACCACTGGCGTAAAGAGAA	RT-qPCR
AtActin2 F	ATTCAGATGCCCAGAAGTCTTGTTCC	RT-qPCR
AtActin2 R	ACCACCGATCCAGACACTGTACTTCC	RT-qPCR
NtActin1 F	GGTATTGTGTTGGACTCGGG	RT-qPCR
NtActin1 R	GCTGTGGTAGTGGATGAGTAAC	RT-qPCR
qHU03G02979.1 F	TGATGATGGTGAGAAGACGA	RT-qPCR
qHU03G02979.1 R	GTAGTCGGAGCGAGAGTAGG	RT-qPCR
qHU03G02980.1 F	GATTCTATCCACCGAACCTG	RT-qPCR
qHU03G02980.1 R	TATCAAAGTCACGAGGCAAA	RT-qPCR
qHU03G00480.1 F	GCTCCAGCCGAACCATACCC	RT-qPCR
qHU03G00480.1 R	TCTTCCTAAAACTCCGCCAT	RT-qPCR
qHU03G00481.1 F	AACCGAACCATACCAAACTC	RT-qPCR
qHU03G00481.1 R	AGGAGAGAGAAGGTGGAAGG	RT-qPCR
qHU05G00563.1 F	CTTTCTTCTCAGTGGATTTGG	RT-qPCR
qHU05G00563.1 R	TTGGGTTTCCCTATTTCATC	RT-qPCR
qHU11G01870.1 F	GGAAATCGCCAAACAAGTC	RT-qPCR
qHU11G01870.1 R	GTGGTGGTCGGAAACCTT	RT-qPCR
qHU07G00504.1 F	GCAACCACCTCACTCAACTT	RT-qPCR
qHU07G00504.1 R	TCTTCCAACAGATGCCAAG	RT-qPCR
qHU09G01023.1 F	ATTGGTTCAAAGGACGAT	RT-qPCR
qHU09G01023.1 R	ATTGTTGCTAAGCAGTTG	RT-qPCR
qHU11G01017.1 F	TGGAGTATCTTTTGCCCTTC	RT-qPCR
qHU11G01017.1 R	AGGCTTTGAGGTGGGTTAGT	RT-qPCR
qHU01G02117.1 F	GAGAGAGTGGGGGAGTTGA	RT-qPCR
qHU01G02117.1 R	GCAAGCCAAGGAATAAAATC	RT-qPCR
qHU07G00784.1 F	TTCGGAGCAGGTAGAAGAAT	RT-qPCR
qHU07G00784.1 R	TTCCAGGCAATACATCAAAG	RT-qPCR
qHU04G00093.1 F	TAGCGATGAACCAAACGAA	RT-qPCR
qHU04G00093.1 R	CAGAACCCAAGAAAGGAAGA	RT-qPCR
qHU03G01342.1 F	TGTGCCTTCTGTATCTGCCG	RT-qPCR
qHU03G01342.1 R	TGTCTTGAACCCTCCTGCTA	RT-qPCR
qHU08G01571.1 F	GGTCGCCGATGCTTTCTGTT	RT-qPCR
qHU08G01571.1 R	TTCTTGTGCTCGCTTTGCCA	RT-qPCR
qHU08G01572.1 F	GACTGATGTGCCTGCTGTTA	RT-qPCR
qHU08G01572.1 R	AGTTCCTCTACCCTCCTTGC	RT-qPCR
qHU01G00156.1 F	GGAGAAGACACGACCGAAC	RT-qPCR
qHU01G00156.1 R	CCGAAGCACACAAGTAAAAG	RT-qPCR
qHU01G00157.1 F	GTTCACTCTTCCTTCGCTTC	RT-qPCR
qHU01G00157.1 R	CATCCAATCACTTTCCTCGT	RT-qPCR
qHU03G00258.1 F	TTGAAAACTGGGGTCTCC	RT-qPCR
qHU03G00258.1 R	ACTTCTTGGCTCTGCTTCTC	RT-qPCR

Primer names	Primer sequences (5'-3')	Assays
qHU03G00266.1 F	CTGAAGCAACCAAAACTGAC	RT-qPCR
qHU03G00266.1 R	AGCCCAAGGAAACTGAAAAT	RT-qPCR
qHU03G00267.1 F	ACTTTGCGACCTGATTGTCT	RT-qPCR
qHU03G00267.1 R	ATGTTCTTGAGTGGTTGGTG	RT-qPCR
qHU10G00514.1 F	ATGCTTTCTTTCCCTTCG	RT-qPCR
qHU10G00514.1 R	ACCTGCTTCCACAATCCTTA	RT-qPCR
qHU06G00241.1 F	TACATTGATTCCCAGACACG	RT-qPCR
qHU06G00241.1 R	GGACAGCCTCCTTTACGAAG	RT-qPCR
qHU06G00243.1 F	GCATTTCAGTCGTCATCCTA	RT-qPCR
qHU06G00243.1 R	AAGGGGCTTGTGGTCCTC	RT-qPCR
qHU07G00076.1 F	GCCCTACATCTTCTACCCTACA	RT-qPCR
qHU07G00076.1 R	TCACCCAACTCCCTAAACTC	RT-qPCR
qHU11G01695.1 F	TCTCACTCTTCCTCCACCTC	RT-qPCR
qHU11G01695.1 R	TCAGCCAACCGATACCTC	RT-qPCR
qHU11G01696.1 F	GGGTCGGTTCTATTTGTGTC	RT-qPCR
qHU11G01696.1 R	GAGTGTTCGTTTGGGCTTC	RT-qPCR
qHU11G01697.1 F	AAACGGTGTCGTGTGACTAC	RT-qPCR
qHU11G01697.1 R	AAATACCCTCAGCCAATACG	RT-qPCR
qHU11G01698.1 F	GGGTCGGTTTTATTTGTGTC	RT-qPCR
qHU11G01698.1 R	GACTTTGGGCAGTGAAGAAC	RT-qPCR
qHU05G01532.1 F	TCCCTCACTTCACCTTTCAT	RT-qPCR
qHU05G01532.1 R	ACCACCATTCATAACCCAAG	RT-qPCR
qHU07G00240.1 F	CGGCGTATTTGTCACTGT	RT-qPCR
qHU07G00240.1 R	CGACCCATCAGCGTATCTTA	RT-qPCR
qHU08G02083.1 F	CCGCAAGGACACAATAGAC	RT-qPCR
qHU08G02083.1 R	ACTGGAAAATGAACTGATGG	RT-qPCR
qHU09G01874.1 F	AACAAACATTCCCTCCAGTG	RT-qPCR
qHU09G01874.1 R	TTGGTAGCCTTTCCAGTAGC	RT-qPCR
qHU09G01875.1 F	TCATCATCTCGTAGCCTCCT	RT-qPCR
qHU09G01875.1 R	CTGTAGGTCGTGGAACTCAA	RT-qPCR
qHU03G02206.1 F	GCAAGCCAATACAACAAGC	RT-qPCR
qHU03G02206.1 R	GCAAAACACCACCGAACG	RT-qPCR
qHU07G01550.1 F	GTGGCAGAACCTCCTCTTTA	RT-qPCR
qHU07G01550.1 R	GGCTTGTTGTATTGGCTTG	RT-qPCR
qHU09G01034.1 F	GTTGCTTCGTGAGCCATT	RT-qPCR
qHU09G01034.1 R	CCCTCTTCTCTACCTCCACA	RT-qPCR
qHU04G00198.1 F	CTGTTTACGGCTGCTTACCT	RT-qPCR
qHU04G00198.1 R	ATTCATCCCTCCACCAAAT	RT-qPCR
qHU10G01599.1 F	AAGAAACCTCCCTGTCAATG	RT-qPCR
qHU10G01599.1 R	GCATCACACTCTCGGAAA	RT-qPCR
qHU07G01678.1 F	TCTATGAAGGTCCCCCTGT	RT-qPCR
qHU07G01678.1 R	ACCAGCGAAAAGAAGAGGTT	RT-qPCR
CYP76AD1 F	ATGGATAGCCCAACCCTCT	Gene cloning

Primer names	Primer sequences (5'-3')	Assays
CYP76AD1 R	TCAATCCTTGCAAACGGGAA	Gene cloning
DODAα1 F	ATGGGTGTTGGCAAAGAAGTGTC	Gene cloning
DODAα1 R	TCAGATGGAAGTGAACTTGTAGG	Gene cloning
DODAα2 F	ATGCTTCAGCAACTAATTAGTGGATGGC	Gene cloning
DODAα2 R	TCAGCAGGAAGTGAATATGTAGGAGG	Gene cloning
B5GT1 F	ATGAACATGGCTGCTGAAGATCAA	Gene cloning
B5GT1 R	TTAAGTGCGATAAACCCTAAGCTCA	Gene cloning
B5GT2 F	ATGGGTGCTCAACCTCAACAGCT	Gene cloning
B5GT2 R	TCAAGCAGCTGGTCGATCCCT	Gene cloning
CDOPA5GT F	ATGGACTCTGCAACAGAGCATA	Gene cloning
CDOPA5GT R	TCAAGCAGCTGGTCGATCCCT	Gene cloning
QCYP76AD1-TRV2 F	AGCCGAACCATACCCAAC	RT-qPCR for VIGS
QCYP76AD1-TRV2 R	GGGAGAGAAGGTGGAAGGT	RT-qPCR for VIGS
QDODAα1-TRV2 F	AGCAGGAGGGTTCAAGACAG	RT-qPCR for VIGS
QDODAα1-TRV2 R	GCTTAGGTGAGGCTGGACTG	RT-qPCR for VIGS
QDODAα2-TRV2 F	GAGACTGATGTGCCTGCTGT	RT-qPCR for VIGS
QDODAα2-TRV2 R	GCTGAACCCTGATGCTGTC	RT-qPCR for VIGS
QB5GT1-TRV2 F	CGGGTCGTTGATGTTGAG	RT-qPCR for VIGS
QB5GT1-TRV2 R	GCAGTTCGGTCGTGTCTTCT	RT-qPCR for VIGS
QB5GT2-TRV2 F	CGAGCAGTATTTGGATAAGACG	RT-qPCR for VIGS
QB5GT2-TRV2 R	AGCGAAGGAAGAGTGAACAG	RT-qPCR for VIGS
pC18-CYP76AD1 F	GGTATCGATAAGCTTATGGATAGCCCAACCCTCTC	transient expression
pC18-CYP76AD1 R	CATACTAGTGGATCCATCCTTGCAAACGGGAATAA	transient expression
pC18-DODAα1 F	GGTATCGATAAGCTTATGGGTGTTGGCAAAGAAGT	transient expression
pC18-DODAα1 R	CATACTAGTGGATCCGATGGAAGTGAACTTGTAGG	transient expression
pC18-DODAα2 F	GGTATCGATAAGCTT ATGCTTCAGCAACTAATTAG	transient expression
pC18-DODAα2 R	CATACTAGTGGATCC GCAGGAAGTGAATATGTAGG	transient expression
pC18-B5GT1 F	GGTATCGATAAGCTTATGAACATGGCTGCTGAAGA	transient expression
pC18-B5GT1 R	CATACTAGTGGATCCAGTGCGATAAACCCTAAGCT	transient expression
pC18-B5GT2 F	GGTATCGATAAGCTTATGGGTGCTGAACCTCAACA	transient expression
pC18-B5GT2 R	CATACTAGTGGATCCAGTGCGATAAACCCTCAACT	transient expression
pC18-CDOPA5GT F	GGTATCGATAAGCTTATGGACTCTGCAACAGAGCA	transient expression
pC18-CDOPA5GT R	CATACTAGTGGATCCAGCAGCTGGTCGATCCCTGC	transient expression
pEAQ-CYP76AD1 F	CAAATTCGCGACCGGTATGGATAGCCCAACCCTCTC	subcellular localization
pEAQ-CYP76AD1 R	TGCTAGCCATACCGGTATCCTTGCAAACGGGAATAA	subcellular localization
pEAQ-DODAα1 F	CAAATTCGCGACCGGTATGGGTGTTGGCAAAGAAGT	subcellular localization
pEAQ-DODAα1 R	TGCTAGCCATACCGGTGATGGAAGTGAACTTGTAGG	subcellular localization
pEAQ-DODAα2 F	CAAATTCGCGACCGGTATGCTTCAGCAACTAATTAGTGGATGGC	subcellular localization
pEAQ-DODAα2 R	TGCTAGCCATACCGGTGCAGGAAGTGAATATGTAGGAGG	subcellular localization
pEAQ-B5GT1 F	CAAATTCGCGACCGGTATGAACATGGCTGCTGAAGATCAA	subcellular localization
pEAQ-B5GT1 R	TGCTAGCCATACCGGTAGTGCGATAAACCCTAAGCTCA	subcellular localization
pEAQ-B5GT2 F	CAAATTCGCGACCGGTATGGGTGCTCAACCTCAACAGCT	subcellular localization
pEAQ-B5GT2 R	TGCTAGCCATACCGGTCGAGTTCCACCCACAGTGAGTCA	subcellular localization

Primer names	Primer sequences (5'-3')	Assays
pEAQ-CDOPA5GT F	CAAATTCGCGACCGGTATGGACTCTGCAACAGAGCATA	subcellular localization
pEAQ-CDOPA5GT R	TGCTAGCCATACCGGTAGCAGCTGGTCGATCCCTGCCG	subcellular localization
pPZP6k90-CYP76AD1 F	CATTCTACAACTACATCTAGAATGGATAGCCCAACCCTCTC	Overexpression
pPZP6k90-CYP76AD1 R	AGCTTGCATGCCAATTCTAGATCAATCCTTGCAAACGGGAATAA	Overexpression
pPZP6k90-DODAα1 F	CATTCTACAACTACATCTAGAATGGGTGTTGGCAAAGAAGT	Overexpression
pPZP6k90-DODAα1 R	AGCTTGCATGCCAATTCTAGATCAGATGGAAGTGAACTTGTAGG	Overexpression
pPZP6k90-DODAα2 F	CATTCTACAACTACATCTAGAATGCTTCAGCAACTAATTAGTGG	Overexpression
pPZP6k90-DODAα2 R	AGCTTGCATGCCAATTCTAGATCAGCAGGAAGTGAATATGTAG	Overexpression
pPZP6k90-B5GT1 F	CATTCTACAACTACATCTAGAATGAACATGGCTGCTGAAGA	Overexpression
pPZP6k90-B5GT1 R	AGCTTGCATGCCAATTCTAGATTAAGTGCGATAAACCCTAAGCT	Overexpression
pPZP6k90-B5GT2 F	CATTCTACAACTACATCTAGAATGGGTGCTGAACCTCAACA	Overexpression
pPZP6k90-B5GT2 R	AGCTTGCATGCCAATTCTAGATTAAGTGCGATAAACCCTCAACT	Overexpression
pPZP6k90-CDOPA5GT F	CATTCTACAACTACATCTAGAATGGACTCTGCAACAGAGCA	Overexpression
pPZP6k90-CDOPA5GT R	AGCTTGCATGCCAATTCTAGATCAAGCAGCTGGTCGATCCCT	Overexpression
TRV2-CYP76AD1 F	GCCTCCATGGGGATCCATGGATAGCCCAACCCTCTC	VIGS
TRV2-CYP76AD1 R	CTTCGGGACATGCCCGGGTCCTAAAACTCCGCCATTTGG	VIGS
TRV2-DODAα1 F	GCCTCCATGGGGATCCATGGGTGTTGGCAAAGAAGTGTC	VIGS
TRV2-DODAα1 R	CTTCGGGACATGCCCGGGATGTCAGCCTCAGGGTACATGAGG	VIGS
TRV2-DODAα2 F	GCCTCCATGGGGATCCACAGCATCAGGGTTCAGC	VIGS
TRV2-DODAα2 R	CTTCGGGACATGCCCGGGTGTGCCAGTTCCCAGTTT	VIGS
TRV2-B5GT1 F	GCCTCCATGGGGATCCAGGTTGAAACAACCCGATCT	VIGS
TRV2-B5GT1 R	CTTCGGGACATGCCCGGGTCTTAGATCCAATGGGCACC	VIGS
TRV2-B5GT2 F	GCCTCCATGGGGATCCACACATGATCCCAACCCT	VIGS
TRV2-B5GT2 R	CTTCGGGACATGCCCGGGTCCAGCACAAAGCGCAAA	VIGS
TRV2-CDOPA5GT F	GCCTCCATGGGGATCCACCAAGCCCGAACTGAAA	VIGS
TRV2-CDOPA5GT R	CTTCGGGACATGCCCGGGATACGCCGCCGTCCCATA	VIGS
pYES3-DODAα1 F	GGGAATATTAAGCTTATGGGTGTTGGCAAAGAAGT	expression in yeast
pYES3-DODAα1 R	ATATCTGCAGAATTCTCAGATGGAAGTGAACTTGTAGG	expression in yeast
pYES3-DODAα2 F	GGGAATATTAAGCTTATGCTTCAGCAACTAATTAGTGG	expression in yeast
pYES3-DODAα2 R	ATATCTGCAGAATTCTCAGCAGGAAGTGAATATGTAG	expression in yeast
pCYP76AD1-F	GACATAAGACTCAATTTACTTGGAA	promoter cloning
pCYP76AD1-R	AGTTATGAAGTAAAAGATTGAGGCA	promoter cloning
pDODAα1-F	CTATGTCTTTTGGTGAGCTGTGTTA	promoter cloning
pDODAα1-R	CCCAGTGAGCAGAGACAACCAGGAT	promoter cloning
pCDOPA5GT-F	TGACTTGGTCAAAACGCTAA	promoter cloning
pCDOPA5GT-R	CTGCGCCATGTATGGGAGCA	promoter cloning
