# Supplementary material for: A Genome-Wide Identification Study Reveals That HmoCYP76AD1, HmoDODAα1 and HmocDOPA5GT Involved in Betalain Biosynthesis in Hylocereus
Source: Genes (Basel). 2021 Nov 23;12(12):1858. doi: 10.3390/genes12121858 (PMC8702118; doi:10.3390/genes12121858)
Supplement: Supplementary file 1 [file genes-12-01858-s001.zip › Supplementary Table S3. 53 candicate genes in pitaya.pdf]

Table S3A. The Basic information of *CYP76AD* in pitaya.

Gene Name	Chr.	Gene Length (bp)	Amino Acids	pI	MW (kDa)	GRAVY [1]	Instability Index
HU01G02117.1	1	24714	733	6.21	82.72	0.032	38.49
HU03G00480.1	3	3338	506	8.91	57.67	-0.117	44.73
HU03G00481.1	3	2837	442	9.39	49.89	-0.111	38.84
HU03G00482.1	3	1684	498	6.51	56.55	-0.095	35.02
HU04G00093.1	4	11846	1025	6.88	114.44	0.082	30.31
HU05G00563.1	5	1978	496	6.82	55.36	-0.093	45.22
HU07G00498.1	7	2814	511	7.00	58.14	-0.135	47.24
HU07G00499.1	7	2403	504	7.73	57.37	-0.145	47.05
HU07G00500.1	7	27114	711	5.99	80.85	-0.110	45.57
HU07G00504.1	7	2282	511	6.58	58.15	-0.123	48.29
HU07G00510.1	7	2515	491	8.95	55.86	-0.178	39.52
HU07G00784.1	7	18290	438	5.98	49.88	0.082	39.83
HU09G01023.1	9	1796	446	6.68	50.46	-0.080	40.71
HU11G01017.1	11	71367	1835	9.22	208.48	-0.275	37.36
HU11G01870.1	11	2639	489	6.90	54.92	-0.076	33.14

[1]: If GRAVY score of a protein was less than zero, it was considered to be hydrophilic.

Table S3B. Basic information of *DODA* genes in pitaya.

Gene Name	Chr.	Gene Length (bp)	Amino Acids	pI	MW (kDa)	GRAVY [1]	Instability Index
HU03G01342.1	3	9485	271	5.66	29.94	-0.163	27.31

HU08G01571.1	8	3921	375	9.25	42.17	-0.421	35.57
HU08G01572.1	8	3882	318	6.22	35.50	-0.207	37.48

[1]: If GRAVY score of a protein was less than zero, it was considered to be hydrophilic.

Table S3C. Basic information of *GT* genes in pitaya.

Gene Name	Chr.	Gene Length (bp)	Amino Acids	pI	MW (kDa)	GRAVY [1]	Instability Index
HU01G00156.1	1	1686	484	5.35	55.40	-0.133	48.52
HU01G00157.1	1	1449	482	5.60	54.91	-0.278	50.02
HU01G00158.1	1	2127	486	6.01	54.69	-0.256	44.93
HU01G00159.1	1	1623	486	6.01	54.70	-0.273	45.83
HU01G00761.1	1	4727	444	5.63	49.89	-0.121	48.64
HU02G01765.1	2	1823	475	5.81	53.33	-0.271	50.74
HU03G00258.1	3	1999	481	5.97	54.31	-0.182	41.87
HU03G00266.1	3	1598	477	5.36	54.01	-0.173	46.80
HU03G00267.1	3	1708	472	5.66	52.84	-0.047	41.00
HU03G01671.1	3	11126	466	5.44	52.27	-0.264	48.56
HU03G02206.1	3	1392	463	6.32	51.33	0.086	35.69
HU04G00198.1	4	21223	869	6.01	96.81	-0.129	43.65
HU04G01617.1	4	2715	464	6.18	51.01	0.103	35.87
HU05G01235.1	5	1805	446	5.70	50.26	-0.209	48.71
HU05G01532.1	5	1445	381	5.33	42.56	-0.036	42.66

HU06G00241.1	6	1490	489	5.47	54.74	-0.116	43.34
HU06G00242.1	6	1470	489	5.34	54.72	-0.108	40.86
HU06G00243.1	6	1628	486	5.30	54.45	-0.236	48.12
HU06G00244.1	6	12389	493	5.63	54.77	-0.151	42.40
HU07G00076.1	7	2061	468	6.00	51.37	-0.082	42.73
HU07G00240.1	7	1520	493	5.73	55.13	-0.211	49.79
HU07G01550.1	7	1392	463	5.73	51.36	0.079	37.37
HU07G01678.1	7	1917	475	5.68	53.40	-0.111	36.95
HU08G02083.1	8	3292	484	5.91	54.19	-0.180	38.07
HU09G01034.1	9	1612	458	5.95	51.02	-0.049	47.49
HU09G01039.1	9	1733	458	5.87	50.79	-0.005	47.44
HU09G01874.1	9	1969	475	5.77	53.74	-0.300	52.89
HU09G01875.1	9	1702	475	5.67	53.52	-0.297	50.08
HU10G00514.1	10	1624	484	5.74	54.75	-0.191	46.15
HU10G01599.1	10	1922	459	5.67	50.94	0.029	44.48
HU11G01695.1	11	2049	474	6.21	52.15	-0.141	44.13
HU11G01696.1	11	1859	480	5.71	52.91	-0.033	42.62
HU11G01697.1	11	1904	479	6.35	52.84	0.032	39.97
HU11G01698.1	11	1413	470	5.93	51.66	-0.028	37.82
HU11G01699.1	11	1716	470	5.65	51.96	-0.090	42.50

[1]: If GRAVY score of a protein was less than zero, it was considered to be hydrophilic.
