# Supplementary material for: A Genome-Wide Identification Study Reveals That HmoCYP76AD1, HmoDODAα1 and HmocDOPA5GT Involved in Betalain Biosynthesis in Hylocereus
Source: Genes (Basel). 2021 Nov 23;12(12):1858. doi: 10.3390/genes12121858 (PMC8702118; doi:10.3390/genes12121858)
Supplement: Supplementary file 1 [file genes-12-01858-s001.zip › Supplementary Table S4. Amino acid sequence similarities between H. monacanthus and H. undatus .pdf]

Table S4. Amino acid sequence similarities between *H. monacanthus* and *H. undatus.*

Gene name	Identity (%)
HU03G00480.1	100.00
HU01G02117.1	62.76
HU04G00093.1	99.51
HU05G00563.1	98.99
HU07G00504.1	99.41
HU07G00784.1	100.00
HU09G01023.1	100.00
HU11G01017.1	97.25
HU11G01870.1	100.00
HU03G01342.1	98.52
HU08G01572.1	99.69
HU08G01571.1	99.20
HU01G00156.1	100.00
HU01G00157.1	100.00
HU03G00258.1	98.75
HU03G00266.1	99.58
HU03G00267.1	100.00
HU10G00514.1	98.35
HU06G00241.1	99.39
HU06G00243.1	96.30
HU07G00076.1	99.79
HU11G01695.1	98.95
HU11G01696.1	99.13
HU11G01697.1	100.00
HU11G01698.1	98.30

Gene name	Identity (%)
HU05G01532.1	100.00
HU07G00240.1	100.00
HU08G02083.1	100.00
HU09G01874.1	99.02
HU09G01875.1	100.00
HU03G02206.1	95.7
HU07G01550.1	100.00
HU09G01034.1	96.72
HU04G00198.1	52.24
HU10G01599.1	100.00
HU07G01678.1	98.74
