# Supplementary material for: A Genome-Wide Identification Study Reveals That HmoCYP76AD1, HmoDODAα1 and HmocDOPA5GT Involved in Betalain Biosynthesis in Hylocereus
Source: Genes (Basel). 2021 Nov 23;12(12):1858. doi: 10.3390/genes12121858 (PMC8702118; doi:10.3390/genes12121858)
Supplement: Supplementary file 1 [file genes-12-01858-s001.zip › Supplementary Text S1. cDNA and amino acid sequences of genes for qPCR in pitaya .pdf]

Text S1. cDNA and amino acid sequences of genes for qPCR. PCR products were marked red color. The sequences from *H. monacanthus* and *H. undatus* were named 'Gene ID-R', and 'Gene ID-W', respectively.

CYP76ADα

> HU03G00480.1-R

ATGGATAGCCCAACCCTCTCGCTTTTCATCTTTGCCTCAATCTTTTACTTCATAACTTTTCAAATTGTGA
AGCTAGGGTTTAATGTGGTCATGACCTCTAAAAAAACCAAAAGAAGAAGACCCCCCTTACCTCCGGG
CCCCAAGCCATTGCCCATCATAGGCAATGTGCTCGAGCTCGGGCCGAAGCCACACCGCTCGTTCGCCG
ACCTAGCCAAGGTCCATGGTCCACTCATGTCCCTCCGGCTAGGTAGTGTGACCACGATTATCGTGTCAT
CCTCTGATGTTGCCAAAGAAATGTTCCTTAAAAATGACCAACCCTTGAGCTCCAGCCGAACCATACCC
AACTCAGTCACGGCTGGGGATCACCACATGCTGACCATGTCTTGGCTCCCAGTCTCCCCCAAATGGCG
GAGTTTTAGGAAGATCACCACCTTCCACCTTCTCTCCCCCCAGCGCCTCGATGCTTGCTCTAGCCTTAG
GCAAGCCAAGGTGCAGCAGCTATTCGAGTACGTTCTGGAATGTTCTAGAACCGGCCAGGCCGTCGAT
ATAGGCAAGGCTGCTTTCACGACGTCCCTTAACTTGTTGTCCAAGCTGTTTTTTCTTTAGAGTTGGCT
CACCATAGATCTAGCAAGTCTCAAGAGTTTAAGGACTTAATTTGGGATATTATGGAGGATATTGGGAAG
CCTAATTACGCGGATTATTTCCCATGCTTAAAGTACTTTGACCCATGTGGAATACGACGTCGTTTGGCA
AATAGTTTTGAGAAATTAATTGAGGTCTTTCAAGGTATTATTCGTCAAAGGCTATCCCTGTCATCTGGCT
CTCATACTCATAATGATGTGTTAGATGTTCTTCTTCAATTGTACAACCAAGAGGAACTCACCATGGACG
AGATAAACCATCTGCTCGTGGATATATTTGATGCCGGAACAGACACCACTTCCAGTACATTTGAATGGG
CCATGGCTGAGTTAATTAAAAATCCGACGATGATGGAGAAAGCTCAAGCTGAAATCAAAGTGGTTCTT
GGGAAACAGTCGCATATTCAAGAGTCCGATATCCCAAAATTGCCTTATTTGCGGGCAATTATCAAAGA
AACATTGCGTCTACACCCTCCTACTGTATTCCTCCTACCTCGTAAGGCTGAGACCGATGTGGAACTCTA
TGGCTACACCGTACCAAAAAATGCACAAATACTGGTGAACTTGTGGGCCTTAGGTCGAGACCCCAAA
GTTTGGGAAAACCCAGAGGTGTTCTTACCTGAAAGGTTCCTGACTTGCGACATCGATGTTAAAGGAA
GAGATTTTGGACTACTGCCTTTTGGGGCAGGAAGGCGAATATGTCCTGGGATGAATTTGGCGTACAGA
ATGCTGACCTTAATGCTCGCTACGCTTCTACAATCGTTTGATTGGAAACTCCCAAATGAGATGAACTCC
AAGAATTTGGACATGGATGAAAAGTTTGGAATAGCATTGCAAAAGACTAAACCCCTTGAAATTATTCC
CGTTTGCAAGGATTGA

MDSPTLSLFIFASIFYFITFQIVKLGFNVVMTSKKTKRRRPPLPPGPKPLPIIGNVLELGPKPHRSFADLAKV
HGPLMSLRLGSVTTIIVSSSDVAKEMFLKNDQPLSSSRTIPNSVTAGDHHMLTMSWLPVSPKWRSFRKITT
FHLLSPQRLDACSSLRQAKVQQLFEYVLECSRTGQAVDIGKAAFTTSLNLLSKLFFSLELAHHRSSKSQEF
KDLIWDIMEDIGKPNYADYFPCLKYFDPCGIRRRLANSFEKLIEVFQGIIRQRLSLSSGSHTHNDVLDVLLQ
LYNQEELTMDEINHLLVDIFDAGTDTTSSTFEWAMAELIKNPTMMEKAQAEIKVVLGKQSHIQESDIPKLP
YLRAIIKETLRLHPPTVFLLPRKAETDVELYGYTVPKNAQILVNLWALGRDPKVWENPEVFLPERFLTCDI
DVKGRDFGLLPFGAGRRICPGMNLAYRMLTLMLATLLQSFDWKLPNEMNSKNLDMDEKFGIALQKTKP

LEIIPVCKD

> HU03G00480.1-W

ATGGATAGCCCAACCCTCTCGCTTTTCATCTTTGCCTCAATCTTTTACTTCATAACTTTTCAAATTGTGA
AGCTAGGGTTTAATGTGGTCATGACCTCTAAAAAAACCAAAAGAAGAAGACCCCCCTTACCTCCGGG
CCCCAAGCCATTGCCCATCATAGGCAATGTGCTCGAGCTCGGGCCGAAGCCACACCGCTCGTTCGCCG
ACCTAGCCAAGGTCCATGGTCCACTCATGTCCCTCCGGCTAGGTAGTGTGACCACGATTATCGTGTCAT
CCTCTGATGTTGCCAAAGAAATGTTCCTTAAAAATGACCAACCCTTGAGCTCCAGCCGAACCATACCC
AACTCAGTCACGGCTGGGGATCACCACATGCTGACCATGTCTTGGCTCCCAGTCTCCCCCAAATGGCG
GAGTTTTAGGAAGATCACCACCTTCCACCTTCTCTCCCCCCAGCGCCTCGATGCTTGCTCTAGCCTTAG
GCAAGCCAAGGTGCAGCAGCTATTCGAGTACGTTCTGGAATGTTCTAGAACCGGCCAGGCCGTCGAT
ATAGGCAAGGCTGCTTTCACGACGTCCCTTAACTTGTTGTCCAAGCTGTTTTTTTCTTTAGAGTTGGCT
CACCATAGATCTAGCAAGTCTCAAGAGTTTAAGGACTTAATTTGGGATATTATGGAGGATATTGGGAAG
CCTAATTACGCGGATTATTTCCCATGCTTAAAGTACTTTGACCCATGTGGAATACGACGTCGTTTGGCA
AATAGTTTTGAGAAATTAATTGAGGTCTTTCAAGGTATTATTCGTCAAAGGCTATCCCTGTCATCTGGCT
CTCATACTCATAATGATGTGTTAGATGTTCTTCTTCAATTGTACAACCAAGAGGAACTCACCATGGACG
AGATAAACCATCTGCTCGTGGATATATTTGATGCCGGAACAGACACCACTTCCAGTACATTTGAATGGG
CCATGGCTGAGTTAATTAAAAATCCGACGATGATGGAGAAAGCTCAAGCTGAAATCAAAGTGGTTCTT
GGGAAACAGTCGCATATTCAAGAGTCCGATATCCCAAAATTGCCTTATTTGCGGGCAATTATCAAAGA
AACATTGCGTCTACACCCTCCTACTGTATTCCTCCTACCTCGTAAGGCTGAGACCGATGTGGAACTCTA
TGGCTACACCGTACCAAAAAATGCACAAATACTGGTGAACTTGTGGGCCTTAGGTCGAGACCCCAAA
GTTTGGGAAAACCCAGAGGTGTTCTTACCTGAAAGGTTCCTGACTTGCGACATCGATGTTAAAGGAA
GAGATTTTGGACTACTGCCTTTTGGGGCAGGAAGGCGAATATGTCCTGGGATGAATTTGGCGTACAGA
ATGCTGACCTTAATGCTCGCTACGCTTCTACAATCGTTTGATTGGAAACTCCCAAATGAGATGAACTCC
AAGAATTTGGACATGGATGAAAAGTTTGGAATAGCATTGCAAAAGACTAAACCCCTTGAAATTATTCC
CGTTTGCAAGGATTGA

MDSPTLSLFIFASIFYFITFQIVKLGFNVVMTSKKTKRRRPPLPPGPKPLPIIGNVLELGPKPHRSFADLAKV
HGPLMSLRLGSVTTIIVSSSDVAKEMFLKNDQPLSSSRTIPNSVTAGDHHMLTMSWLPVSPKWRSFRKITT
FHLLSPQRLDACSSLRQAKVQQLFEYVLECSRTGQAVDIGKAAFTTSLNLLSKLFFSLELAHHRSSKSQEF
KDLIWDIMEDIGKPNYADYFPCLKYFDPCGIRRRLANSFEKLIEVFQGIIRQRLSLSSGSHTHNDVLDVLLQ
LYNQEELTMDEINHLLVDIFDAGTDTTSSTFEWAMAELIKNPTMMEKAQAEIKVVLGKQSHIQESDIPKLP
YLRAIIKETLRLHPPTVFLLPRKAETDVELYGYTVPKNAQILVNLWALGRDPKVWENPEVFLPERFLTCDI
DVKGRDFGLLPFGAGRRICPGMNLAYRMLTLMLATLLQSFDWKLPNEMNSKNLDMDEKFGIALQKTKP
LEIIPVCKD

CYP76ADγ

>HU01G02117.1-R

ATGCCTTCCTTTGAGGCTGCTGTGCCCCTGATCTTACTCCTTCCGCTCCTATGGGTATTTTTCAAGGCA
AAGACCCGAAAACCCGGCCCAAACTTGCCACCAGGCCCCCGGCCGCTCCCGATAATCGGAAACCTGC
ACATGTTGGGCCAGAACCCGCATCGAGCTTTAGCAGAATTGGCCCAAAAGTACGGCCCAATCATGTC
GCTCCGCCTGGGTCACGTGCCTGCCATAGTTGTATCGTCAGCTCAGGCGGCCGAGCTCTTCCTTAAGC
AACATGATGCCCCTTTTGCCTACCGGCCCGTGCTACTAGTTTGGGATCACATGGTCTACGGAGCCAAG
GACGTGGCATTCACGCCTTATGGGGATTACTGGCGGCGTGTGAGGAAGATGTGCGTCATCCACCTCCT
TAGCGCCGCCAAGGTGGCCGGGTTCGAGGGGCTGAGGAGGGCGGAGATCGAGGCAGCCGTGGGGCG
GCTGGCAGAGGCTGCGGTGGCGCGTGAGGTGGTGGATGTGGGGGAGAGAGTGGGGGAGTTGATTGA
AGACATAGTGTTTAAGATGTTGATTGGAAAGGGGAAGGAGGATAAGAGGTATGATTTCAGGGGAGTG
ATTCAGGAGGCTTTGGCTCTTGCTGGGGCATTTAACTTGGCTGATTTTATTCCTTGGCTTGCCCCGCTT
GACTTGCAGGGACTAATGCGAAGGACAAAAGCTGCAAGGAAGGCCACAGATGAAATATTTGATAACA
TTATTGAGGACCATCTCAAAGAAGAAGATTTTGGACAAAATAGGGACATTATTGGTTTGTTGCTATCTC
TTATGCATAACCCTACAAACGAGTTGCACTCCTCCTCATTTGGCCTAGACAATGTCAAAGCTCTTATGT
TCGACTTGTTTGCTGGTGGCATCGACACCACATACGTCGCCATAACATGGGTCCTCTCAGCACTCATTA
AGCACCCACGGGTGATGAGACTCGTCCAAGATGAGCTTGACTCCATAGTTGGACGGGAGAGAATGCT
AGTGGAATCAGACTTGTCGAAATTAACCTATTTAGACATGGTGATAAAAGAGACTTTTAGGCTTTACCC
TGCAGGGCCACTATCGGTGCCTCATGAGTCTATGGAGGATGTTATTGTAAATGGGTTTATGATACTCAA
AAAGTCAAGGGTTATTATAAATGTTTGGGCAATTGGTCGAGACCCAAATGTATGGTCAGAAAATGCTG
AGGAGTTTTACCCAGAGAGATTTATAGGTAGGAATATCGATGTTCGAGGACTGCATTTTGAGTTGATCC
CATTTGGAGCTGGGCGAAGAGAGTGTCCTGGGATGCATTTGGCTTTGATCCATGTCAAGCTAGTTGTT
GCTCACCTGGTACATTGTTTTGATTGGGAGCTCCCCAATGGAATGCATCCCGATGATGTGGACATGATG
GAGAAATTCGGGCTGTCAATGCCAAGAGCTAATAAGTTGTCAGCGATACCTCGTTATCGCTTA

MPSFEAAVPLILLLPLLWVFFKAKTRKPGPNLPPGPRPLPIIGNLHMLGQNPHRALAELAQKYGPIMSLRL
GHVPAIVVSSAQAAELFLKQHDAPFAYRPVLLVWDHMVYGAKDVAFTPYGDYWRRVRKMCVIHLLSAA
KVAGFEGLRRAEIEAAVGRLAEAAVAREVVDVGERVGELIEDIVFKMLIGKGKEDKRYDFRGVIQEALAL
AGAFNLADFIPWLAPLDLQGLMRRTKAARKATDEIFDNIIEDHLKEEDFGQNRDIIGLLLSLMHNPTNELH
SSSFGLDNVKALMFDLFAGGIDTTYVAITWVLSALIKHPRVMRLVQDELDSIVGRERMLVESDLSKLTYLD
MVIKETFRLYPAGPLSVPHESMEDVIVNGFMILKKSRVIINVWAIGRDPNVWSENAEEFYPERFIGRNIDVR
GLHFELIPFGAGRRECPGMHLALIHVKLVVAHLVHCFDWELPNGMHPDDVDMMEKFGLSMPRANKLSAI
PRYRL

>HU01G02117.1-W

ATGCCTTCCTTTGACTTTGAGGCTGCTGCGCCCCTGATCTTACTCCTTCCGCTCCTATGGGTATTTTTCA
AGGCAAAGACCCGAAAACCCGGCCCAAACTTGCCACCAGGCCCCCGGCCGCTCCCGATAATCGGAA
ACCTGCACATGTTGGGCCAGAACCCGCATCGGGCTTTAGCAGAATTGGCCCAAAAGTACGGCCCAAT
CATGTCGCTCCGCCTGGGTCACGTGCCTGCCATAGTTGTATCGTCAGCTCAGGCGGCCGAGCTCTTCC

TTAAGCAACATGACGCCCCTTTTGCCTACCGGCCCGTGCTACTAGTTTGGGATCACATGGTCTACGGA
GCCAAGGACGTGGCATTCACGCCTTATGGGGATTACTGGCGGCGTGTGAGGAAGATGTGCGTCATCC
ACCTCCTTAGTGCCGCCAAGGTGGCCGGGTTCGAGGGGCTGAGGAGGGCGGAGATCGAGGCAGCCG
TGGGGCGGCTGGCAGAGGCTGCGGTGGCGCGTGAGGTGGTGGATGTAGGGGAGAGAGTGGGGGAGT
TGATTGAAGACATAGTGTTTAAGATGTTGATTGGAAAGGGGAAGGAGGATAAGAGGTATGATTTCAGG
GGAGTGATTCAGGAGGCTTTGGCTCTTGCTGGGGCATTTAACTTGGCTGATTTTATTCCTTGGCTTGCC
CCGCTTGACTTGCAGGGACTAATGCGACGGACAAAAGCTGCAAGGAAGGCCACAGATGAAATATTTG
ATAACATTATTGAGGACCATCTCAAAGAAGAAGATTTTGGACAAAATAGGGACATTATTGGTTTGTTGC
TATCTCTTATGCATAACCCTACAAACGAGTTGCACTCCTCCTCATTTGGCCTAGACAATGTCAAAGCTC
TTATGTTCGACTTGTTTGCTGGTGGCATCGACACCACATACGTCGCCATAACATGGGTCCTCTCAGCAC
TCATTAAGCACCCACGGGTGATGAGACTCGTCCAAGATGAGCTTGACTCCATAGTTGGACGGGAGAG
AATGCTAGTGGAATCAGACCTGTCGAAATTAACCTATTTAGACATGGTGATAAAAGAGACTTTTAGGC
TTTACCCTGCAGGGCCACTATCGGTGCCTCATGAGTCTATGGAGGATGTTATTGTAAATGGGTTTATGAT
ACTCAAAAAGTCAAGGGTTATTATAAATGTTTGGGCAATTGGTCGAGACCCAAATGTATGGTCAGAAA
ATGCTGAGGAGTTTTACCCAGAGAGATTTATAGGTAGGAATATCGATGTTCGAGGACTGCATTTTGAGT
TGATCCCATTTGGAGCTGGGCGAAGAGAGTGTCCTGGGATGCATTTGGCTTTGATACATGTCAAGCTA
GTTGTTGCTCACCTGGGACTAACTCGAAAGATTAAAGCTGTAAGTAAGGTTGCAGATGAAATGGCTG
AGAATATAATTGAGGATCATCTCAAAGGAGAAGATTTTGGACAACAAAGGGACATTGTAGGTGCTATG
CTAGCCACAATGGCGAACCCCAACAGCGAGTTCTTTTCTACTTTTGATCGAGAAAATGTTAAAGGCAT
CATGCTTGACTTATTTGTTGGTGGGATTGACTCATCTCTCCAAACCATTATGTGGGCACTCTCGGCACT
AACCAAGCATCCAAGAGTGATGAAGCTCCTGCAAGAAGAGCTAGATGCAAAAATCGGGAGACATAG
AATGGTGTTGGAATCAGACTTGCGGAAATTAACTTATTTAGACATGGTGGTGAAGGAGACTTTTAGGC
TTCACCCTGTGGGACCACTGTTAGTTCCCCATGAGTCTATGGAGGATGTTATCGTAGATGGGTATATGA
TTCCGAAGAAATCAAGGGTTATCATCAACTTTTATGCTATTGGGCACGATCCCAATGTATGGTCGGAAA
ACGTAGAGGACTTTTATCCAGAGAGATTTATGGGTAGAGAGACTGACGTCCGAGGGCAAGATTTTGA
ATTAATCCCATTTGGCGCAGGACGAAGAGGGTGCCCGGGAATACATTTGGGTTTGATCACAGTGAAAA
TAGTGTTAGCTCAACTGGTACATTGTTTTAATTGGGAGCTCCCAAATGGTATGCGGCCTGAAGAAGTT
GACATGACTGAGGAGTTCGGGTTAGCAATGTCTAGAGTTAATAAGTTGTTAGCTATACCATCTTATCGC
TTATTACTCATTCTTAAGTACCATTGA

MPSFDFEAAAPLILLLPLLWVFFKAKTRKPGPNLPPGPRPLPIIGNLHMLGQNPHRALAELAQKYGPIMSL
RLGHVPAIVVSSAQAAELFLKQHDAPFAYRPVLLVWDHMVYGAKDVAFTPYGDYWRRVRKMCVIHLLS
AAKVAGFEGLRRAEIEAAVGRLAEAAVAREVVDVGERVGELIEDIVFKMLIGKGKEDKRYDFRGVIQEAL
ALAGAFNLADFIPWLAPLDLQGLMRRTKAARKATDEIFDNIIEDHLKEEDFGQNRDIIGLLLSLMHNPTNE
LHSSSFGLDNVKALMFDLFAGGIDTTYVAITWVLSALIKHPRVMRLVQDELDSIVGRERMLVESDLSKLTY
LDMVIKETFRLYPAGPLSVPHESMEDVIVNGFMILKKSRVIINVWAIGRDPNVWSENAEEFYPERFIGRNID
VRGLHFELIPFGAGRRECPGMHLALIHVKLVVAHLGLTRKIKAVSKVADEMAENIIEDHLKGEDFGQQRDI
VGAMLATMANPNSEFFSTFDRENVKGIMLDLFVGGIDSSLQTIMWALSALTKHPRVMKLLQEELDAKIGR
HRMVLESDLRKLTYLDMVVKETFRLHPVGPLLVPHESMEDVIVDGYMIPKKSRVIINFYAIGHDPNVWSE

NVEDFYPERFMGRETDVRGQDFELIPFGAGRRGCPGIHLGLITVKIVLAQLVHCFNWELPNGMRPEEVDM
TEEFGLAMSRVNKLLAIPSYRLLLILKYH

>HU04G00093.1-R

ATGGATACATCAGTTACTATAGTTGAGTGGGCCATGGCAGAGATGCTGCGACATCCTAAGGTAATGGAGGA
AGTCCAAGAAGAATTGGCCAAGATTGTGGGATTAAATAGTACTGTCGAAGAGGCTCATTTGTCCGAGTTGA
AGTACCTGAAAGCCGTTGTGAAGGAGACACTTCGTTTGCACTCGCCTCTACCTCTCCTTGTTCCCCATTGTT
CGAGCAAGCCTACCACTGTAGGAGGATATATGATACCAAAGGGCTCTCAGGTCTTCCTTAATGTTCGAGCA
ATTCAAACAGACCCGCAGCTTTGGGAAGCTCCATTAGAGTTTCAACCTGGGCGGTTTATGAGGGGCTCCTC
TGCTGAGGCAGTTGACTACTGGGGTAACCACCTTCAGTTTCTTCCATTCGGCTCCGGGAGGAGAATGTGTC
CTGGTATTCCTCTAGCAGAGAGAACAGTCATGCATGTCTTAGCTTCACTGTTGCATGCTTTTGAGTGGTCAT
TACCAAACGGTGTTGAAGATGTGAATCTCTCTGAGAAATTTGGAATCGTTGTGAAAAAAGAAAAACCATT
ACTGGCCGTCCCAAAACTGGCGAGGACCTATGGTCCCATCTTTAAGGTCCGGCTAGGAACCAAGACTTGC
ATTGTGATCAGCTCACCCTACTTTCTCAAAGAGGTGGTGCGAGACCAAGATGTCACTTTTGCTAACCGTGA
TCCTACGGCTGTTGCTATTACATGCTTTTATGGGGGATCCGATATTACTTTCACCAATTACGGCGCTGAATGG
CGAAAGATGAGGAAAATATTGGCCAGTGAAATGCTAAGTAATTCTAGCCTCGATTCTACTTATCATCTCAGG
AAACAACAGGTGAAGAAAATGATAGCAGATACATACGAAAAGGCCGGTGAAGTAGTTGATATAGGTGAAT
TGGCTTTTCTCACCATTATGAATGCTACGATGAGCATGGTATGGGGACAAACACTTAAAGGCAACCAGGGT
TATGCTGTGAAGTCTGAGTTTCGAGATGTGATAAGAGAATTCATGGAGCTGTTAGGGGCACCAAATATTTC
GGACCTTTTCCCTTTGCTTGCTAGGTTTGATTTGCAAGGAATAAAGTGCAAAGCGGAGATTATCTCCCGCC
AATTTAATGAGATCCTGGATTCTGCCATAAATATTCACAACTGTGGAGAAAAAAGTGGGCAGAAAGATTTT
CTGGGATACCTCTTACCACTCACCAAGTGCGAAGATCCTACAACATCGCTCACCTTGGTTCAAGCAAAGGC
CTTGCTCATTGACATTATCATTGGGGGAATGGATACAACAGTTACTGCAGTCGAGTGGGCTATGGTGCTGAT
GCTGAAACATCCGAAGGTAATGAGGAAAGTCCAGCTCTACCACGTTTCGATCTTAGACACATTCTTAATCC
TTTCTCTTATAAGTACTGCTAGATTTCACTGGCTTAGCTGCATTACTTTGTTGATCCCCCTCTCATGCACGTTA
ACTCACATATTGTATGAAATGGCGTCATGGTGGTGGGATACTAGCGATGAACCAAACGAAGCCTCTCTTAA
AATCTTCATTGCCTTGTTCTTCTTGATCATTCTTTGGTTCATTTCCCTGCTCTGTAAACAAAACAAGGCGACA
CCTCCCCTGCCTCCTGGACCCAATGGCTTGCCTTTCGTTGGATATCTTCCTTTCTTGGGTTCTGATCTTCTAT
GCTCTTTTACACAACTGGCGAGGACCTATGGTCCCATCTTTAAGGTCCGGCTAGGAACCAAGACTTGCATT
GTGATCAGCTCGCCCTCCTTTCTCAAAGAGGTGGTGCGAGACCAAGATGTCACTTTTGCCAACCGTGATCC
TACTGTTGTTGCTGTTACATGCATATATGGGGGATCCGATATTGCTTTCACCAATTACGGCCCTGAATGGCGA
AAGCTGAGGAAAATCTTGGCCAGTGAAATGCTAAGTAATTCTAACCTCGATTCTGCTTATCATTTCAGGAA
ACAACAGGTGAAGAAAATGATAGCAGATACATATGACAAGGCCGGTGGAGCAGTTGATGTAGGTGAATTG
GCTTTTCTCACCATTATGAATTCTATGATGAGCATGGTATTGGGACAAACACTTGAAGGCAACCAGGGTTAT
GCTGTAAAGTCTGAGTTTCGAGACGTGATAAGAGAATTCATGGAGCTGTTAGGGGCAGCAAATATTTCGGA
CCTGTTCCCTTTGCTTGCTAGGTTTGATTTGCAAGGAATAAAGTGCAAAGCAGAGATGATCTCCCACCAATT
TGATGAGATCCTGGATTCTGCCATAAATATTCACAACTGTGGAGAAAAAAGTGGGCAGAAAGATTTTCTGG
GATGCCTCTTAGCACTTACCAAGCGCGAAGATCCTACAACATCGCTCACCTTGGTTCAAGCAAAGGCCACG

ATCATTGACATTATTATTGGGGGAGTGGAGACAACAGTTAGTACAGTCGAGTGGGCTACGGTGCTGATGCT
GAAACATCCAAAGGTAATGAGGAAAGTCCAAGAAGAATTAGCTGAGATTGTGGGACTAAATACTTCTGTT
GAAGAGGTTCATTTTCCAAAGTTGAAGTACCTGAAAGCTGTTGTGAAGGAGACCTTACGTTTGCACCCGC
CAATACCTTTCCTCGTTCCCCATTGTCCAAGCAAATCTACCACTGTAGGAGGATATACCATACCAAAGGGTG
CTCAGATCTACCTCAATTTGTTTGCTATGCAAAAGGACGCGCAACTTTGGGGAGATCCTCTAGAGTTTAAA
CCCGAGAGGTTCTTAAAGGGCTCATCAGCGGGGGGCTTGGACTACTCAGGCAACCATCTTCAGTATCTTCC
ATTTGGGTCAGGGCGAAGAGCATGCCCTGGCGTGCCTTTGGCAGATAGGATGTTGATGCTTGTCTTGGCTT
CACTTTTGCATGCTTTTGAGTGGTCATTACCTAATGGAGTTGAGGATGTCGACCTCTCTGAGAGATTTGGAA
TCGTCGTGAAAAAATCAAAACCTTTACTCGCTATCCCTTGTCCCAGACTCTCCAGCTTGGAGCTCTATTCAT
ACTCAGAATAG

MDTSVTIVEWAMAEMLRHPKVMEEVQEELAKIVGLNSTVEEAHLSELKYLKAVVKETLRLHSPLPLLVP
HCSSKPTTVGGYMIPKGSQVFLNVRAIQTDPQLWEAPLEFQPGRFMRGSSAEAVDYWGNHLQFLPFGSG
RRMCPGIPLAERTVMHVLASLLHAFEWSLPNGVEDVNLSEKFGIVVKKEKPLLAVPKLARTYGPIFKVRL
GTKTCIVISSPYFLKEVVRDQDVTFANRDPTAVAITCFYGGSDITFTNYGAEWRKMRKILASEMLSNSSLD
STYHLRKQQVKKMIADTYEKAGEVVDIGELAFLTIMNATMSMVWGQTLKGNQGYAVKSEFRDVIREFM
ELLGAPNISDLFPLLARFDLQGIKCKAEIISRQFNEILDSAINIHNCGEKSGQKDFLGYLLPLTKCEDPTTSLT
LVQAKALLIDIIIGGMDTTVTAVEWAMVLMLKHPKVMRKVQLYHVSILDTFLILSLISTARFHWLSCITLLI
PLSCTLTHILYEMASWWWDTSDEPNEASLKIFIALFFLIILWFISLLCKQNKATPPLPPGPNGLPFVGYLPFL
GSDLLCSFTQLARTYGPIFKVRLGTKTCIVISSPSFLKEVVRDQDVTFANRDPTVVAVTCIYGGSDIAFTNY
GPEWRKLRKILASEMLSNSNLDSAYHFRKQQVKKMIADTYDKAGGAVDVGELAFLTIMNSMMSMVLGQ
TLEGNQGYAVKSEFRDVIREFMELLGAANISDLFPLLARFDLQGIKCKAEMISHQFDEILDSAINIHNCGEK
SGQKDFLGCLLALTKREDPTTSLTLVQAKATIIDIIIGGVETTVSTVEWATVLMLKHPKVMRKVQEELAEIV
GLNTSVEEVHFPKLKYLKAVVKETLRLHPPIPFLVPHCPSKSTTVGGYTIPKGAQIYLNLFAMQKDAQLW
GDPLEFKPERFLKGSSAGGLDYSGNHLQYLPFGSGRRACPGVPLADRMLMLVLASLLHAFEWSLPNGVE
DVDLSERFGIVVKKSKPLLAIPCPRLSSLELYSYSE

>HU04G00093.1-W

ATGGATACATCAGTTACTATAGTTGAGTGGGCCATGGCAGAGATGCTGCGACATCCTAAGGTAATGGAGGA
AGTCCAAGAAGAATTGGCCAAGATTGTGGGATTAAATAGTACTGTCGAAGAGGCTCATTTGTCCGAGTTGA
AGTACCTGAAAGCCGTTGTGAAGGAGACACTTCGTTTGCACTCGCCTCTACCTCTCCTTGTTCCCCATTGTT
CGAGCAAGCCTACCACTGTAGGAGGATATATGATACCAAAGGGCTCTCAGGTCTTCCTTAATGTTCGAGCA
ATTCAAACAGACCCGCAGCTTTGGGAAGCTCCATTAGAGTTTCAACCTGGGCGGTTTATGAGGGGCTCCTC
TGCTGAGGCAGTTGACTACTGGGGTAACCACCTTCAGTTTCTTCCATTCGGCTCCGGGAGGAGAATGTGTC
CTGGTATTCCTCTAGCAGAGAGAACAGTCATGCATGTCTTAGCTTCACTGTTGCATGCTTTTGAGTGGTCAT
TACCAAACGGTGTTGAAGATGTGAATCTCTCTGAGAAATTTGGAATCGTTGTGAAAAAAGAAAAACCATT
ACTGGCCGTCCCAAAACTGGCGAGGACCTATGGTCCCATCTTTAAGGTCCGGCTAGGAACCAAGACTTGC
ATTGTGATCAGCTCACCCTACTTTCTCAAAGAGGTGGTGCGAGACCAAGATGTCACTTTTGCTAACCGTGA
TCCTACGGCTGTTGCTATTACATGCTTTTATGGGGGATCCGATATTACTTTCACCAATTACGGCGCTGAATGG

CGAAAGATGAGGAAAATATTGGCCAGTGAAATGCTAAGTAATTCTAGCCTCGATTCTACTTATCATCTCAGG
AAACAACAGGTGAAGAAAATGATAGCAGATACATACGAAAAGGCCGGTGAAGTAGTTGATATAGGTGAAT
TGGCTTTTCTCACCATTATGAATGCTACGATGAGCATGGTATGGGGACAAACACTTAAAGGCAACCAGGGT
TATGCTGTGAAGTCTGAGTTTCGAGATGTGATAAGAGAATTCATGGAGCTGTTAGGGGCACCAAATATTTC
GGACCTTTTCCCTTTGCTTGCTAGGTTTGATTTGCAAGGAATAAAGTGCAAAGCGGAGATTATCTCCCGCC
AATTTAATGAGATCCTGGATTCTGCCATAAATATTCACAACTGTGGAGAAAAAAGTGGGCAGAAAGATTTT
CTGGGATACCTCTTACCACTCACCAAGTGCGAAGATCCTACAACATCGCTCACCTTGGTTCAAGCAAAGGC
CTTGCTCATTGACATTATCATTGGGGGAATGGATACAACAGTTACTGCAGTCGAGTGGGCTATGGTGCTGAT
GCTGAAACATCCGAAGGTAATGAGGAAAGTCCAGCTCTACCACGTTTCGATCTTAGACACATTCTTAATCC
TTTCTCTTATAAGTACTGCTAGATTTCACTGGCTTAGCTGCATTACTTTGTTGATCCCCCTCTCATGCACGTTA
ACTCACATATTGTATGAAATGGCGTCATGGTGGTGGGATACTAGCGATGAACCAAACGAAGCCTCTCTTAA
AATCTTCATTGCCTTGTTCTTCTTGATCATTCTTTGGTTCATTTCCCTGCTCTGTAAACAAAACAAGGCGACA
CCTCCCCTGCCTCCTGGACCCAATGGCTTGCCTTTCGTTGGATATCTTCCTTTCTTGGGTTCTGATCTTCTAT
GCTCTTTTACACAACTGGCGAGGACCTATGGTCCCATCTTTAAGGTCCGGCTAGGAACCAAGACTTGCATT
GTGATCAGCTCGCCCTCCTTTCTCAAAGAGGTGGTGCGAGACCAAGATGTCACTTTTGCCAACCGTGATCC
TACTGTTGTTGCTGTTACATGCATATATGGGGGATCCGATATTGCTTTCACCAATTACGGCCCTGAATGGCGA
AAGCTGAGGAAAATCTTGGCCAGTGAAATGCTAAGTAATTCTAACCTCGACTCTGCTTATCATTTCAGAAA
ACAACAGGTGAAGAAAATGATAGCAGATACATATGACAAGGCCGGTGGAGCAGTTGATGTAGGTGAATTG
GCTTTTCTCACCATTATGAATTCTATGATGAGCATGGTATTGGGACAAACACTTGAAGGCAACCAGGGTTAT
GCTGTAAAGTCTGAGTTTCGAGATGTGATAAGAGAATTCATGGAGCTGTTAGGGGCACCAAATATTTCGGA
CCTTTTCCCTTTGCTTGCTAGGTTTGATTTGCAAGGAATAAAGTGCAAAGCAGAGATGATCTCCCGCCAATT
TGATGAGATCCTGGATTCTGCCATAAATATTCACAACTGTGGAGAAAAAAGTGGGCAGAAAGATTTTCTGG
GATGCCTCTTAGCACTTACCAAGCGCGAAGATCCTACAACATCGCTCACCTTGGTTCAAGCAAAGGCCACG
ATCATTGACATTATTATTGGGGGAGTGGAGACAACAGTTAGTACAGTCGAGTGGGCTACGGTGCTGATGCT
GAAACATCCAAAGGTAATGAGGAAAGTCCAAGAAGAATTAACTGAGATTGTGGGACTAAATACTTCTGTT
GAAGATGTTCATTTTCCAAAGTTGGAGTACCTGAAAGCTGTTGTGAAGGAGACCTTACGTTTGCACCCGCC
TATACCTTTCCTCGTTCCCCATTGTCCAAGCAAATCTACCACTGTAGGAGGATATACCATACCAAAGGGTGC
TCAGATCTACCTCAATTTGTTTGCTATGCAAAAGGACGCGCAACTTTGGGGAGATCCTCTAGAGTTTAAAC
CCGAGAGGTTCTTAAAGGGCTCATCAGCGGGGGGCTTGGACTACTCGGGCAACCATCTTCAGTATCTTCCA
TTTGGGTCAGGGCGAAGAGCATGCCCTGGCGTGCCTTTGGCAGATAGGATGTTGATGCTTGTCTTGGCTTC
ACTTTTGCATGCTTTTGAGTGGTCATTACCTAATGGAGTTGAGGATGTCGACCTCTCTGAGAGATTTGGAAT
CGTCGTGAAAAAATCAAAACCTTTACTCGCTATCCCTTGTCCCAGACTCTCCAGCTTGGAGCTCTATTCATA
CTCAGAATAG

MDTSVTIVEWAMAEMLRHPKVMEEVQEELAKIVGLNSTVEEAHLSELKYLKAVVKETLRLHSPLPLLVP
HCSSKPTTVGGYMIPKGSQVFLNVRAIQTDPQLWEAPLEFQPGRFMRGSSAEAVDYWGNHLQFLPFGSG
RRMCPGIPLAERTVMHVLASLLHAFEWSLPNGVEDVNLSEKFGIVVKKEKPLLAVPKLARTYGPIFKVRL
GTKTCIVISSPYFLKEVVRDQDVTFANRDPTAVAITCFYGGSDITFTNYGAEWRKMRKILASEMLSNSSLD
STYHLRKQQVKKMIADTYEKAGEVVDIGELAFLTIMNATMSMVWGQTLKGNQGYAVKSEFRDVIREFM

ELLGAPNISDLFPLLARFDLQGIKCKAEIISRQFNEILDSAINIHNCGEKSGQKDFLGYLLPLTKCEDPTTSLT
LVQAKALLIDIIIGGMDTTVTAVEWAMVLMLKHPKVMRKVQLYHVSILDTFLILSLISTARFHWLSCITLLI
PLSCTLTHILYEMASWWWDTSDEPNEASLKIFIALFFLIILWFISLLCKQNKATPPLPPGPNGLPFVGYLPFL
GSDLLCSFTQLARTYGPIFKVRLGTKTCIVISSPSFLKEVVRDQDVTFANRDPTVVAVTCIYGGSDIAFTNY
GPEWRKLRKILASEMLSNSNLDSAYHFRKQQVKKMIADTYDKAGGAVDVGELAFLTIMNSMMSMVLGQ
TLEGNQGYAVKSEFRDVIREFMELLGAPNISDLFPLLARFDLQGIKCKAEMISRQFDEILDSAINIHNCGEK
SGQKDFLGCLLALTKREDPTTSLTLVQAKATIIDIIIGGVETTVSTVEWATVLMLKHPKVMRKVQEELTEIV
GLNTSVEDVHFPKLEYLKAVVKETLRLHPPIPFLVPHCPSKSTTVGGYTIPKGAQIYLNLFAMQKDAQLW
GDPLEFKPERFLKGSSAGGLDYSGNHLQYLPFGSGRRACPGVPLADRMLMLVLASLLHAFEWSLPNGVE
DVDLSERFGIVVKKSKPLLAIPCPRLSSLELYSYSE

>HU05G00563.1-R

ATGGATTACCTGAACTTCCTGTTGTGTCTAGTACTAGCATGGGTTGCAATCTACCTCCTTCTCTCAACT
GCAAAGAAAGGCAAAGCAGCAAATCCTAGAATCCTACCACCAGGTCCTTCCCCTCTACCCATCTTTGG
AAACCTCTTCATTCTGGGCACCAAGCCCCATATCTCCTTGACTGAACTCGCCAGAACCTATGGTCCCC
TCATGAGTCTCCAGCTCGGCCAAGTGCCCACAGTAGTCATTTCTTCAGCTGTTGTGGCCAAGGAAGCC
CTTCAAAAGAATGATATTTCCTTCTCCAGTCGAACAGTTGGAGATGCTGTTTGTGCACTCAGCCATCA
CCAAAGTTCAATAATCTGGTTGCCGGCAGCAGCTCAATGGAGAAATCTGCGCAAAATCTGCAACTCCC
ATGTCTTTTCCAACAGCAGGCTAGAAAGCAGCCAAAATCTCCGAAGAGATAAGGTGAAAGATCTCAT
TTCCCATGTCCAAAAATGCAGTGAATCTGGCATGACAGTGGATATTGGCCAGGCTGCATTTACTACTAC
CTCAAATCTGCTATCAAACACTTTCTTCTCAGTGGATTTGGGAGACCCAACTTCTGAATTTGCTCGCGA
ATTTAAAGGGGTTGTACGAGGTGTTGTGGATGAAATAGGGAAACCCAACTTTGCAGATTACTTCCCTC
TCCTGAGAAACATGGATCCACAAGGCATCAGGCGGCGCATGAGCATCCACTTTGGGAAGATGTTGGG
CCTATTCAATGACATGATTAACCAAAGGTTGCAGGGCAAGAGGCCATCAGAAGGTAATGATGTCTTGG
ATGCATTGATGGGCTTCAATCAAGAGAAATCGGAAGAAATCGAGCAATCAAACATTCCTCATCTATTA
GTGGATTTATTCGCAGCCGGAACTGATACAACATCAAGCACCCTTGAATGGGCAATGACAGAAATTCT
CCGCAACCCAGAGATACTCAAGAAAGCACAAGCCGAGCTCGAAGAAATCATCGGAAAAGGAAACCC
AGTCGAAGAACCAGACATTTCCCGGCTGCCATACTTGCAGGCCATCGTCAATGAAACCTTCCGATTGC
ATCCCGCAGTGGCGCTAGTGGTGCGAAAAGTTGATTCAGATGTCAAGCTCTTCAACTACACAGTGCCC
AAAAACGCACAAGTGTTGGTCAATTTGTGGGCAATTGGGAGAGATCCAGAGATTTGGGAGGAACCCA
ACACATTTGAACCAGAAAGATTTCTGGGATCAGAAGTTGATGTCAAAGGTCGCGATTTTAAGCTGATT
CCCTTTGGAGCTGGCCGTCGGATATGCCCTGGGCTGCCATTGGCAACCAGGATGATCCATTTAATGTTG
GCTTCGCTCATTCATGGGTTTGATTGGAAGCTTGAAGCTGGGGTTTCACCTGAGAACACGGACATGGA
GGAGAGATTTGGGCTCACTCTGGAGAAAGCGCAGCGGCTTCGTGCCATTCCTGTACGTGTATGA

MDYLNFLLCLVLAWVAIYLLLSTAKKGKAANPRILPPGPSPLPIFGNLFILGTKPHISLTELARTYGPLMSLQ
LGQVPTVVISSAVVAKEALQKNDISFSSRTVGDAVCALSHHQSSIIWLPAAAQWRNLRKICNSHVFSNSRL
ESSQNLRRDKVKDLISHVQKCSESGMTVDIGQAAFTTTSNLLSNTFFSVDLGDPTSEFAREFKGVVRGVV
DEIGKPNFADYFPLLRNMDPQGIRRRMSIHFGKMLGLFNDMINQRLQGKRPSEGNDVLDALMGFNQEKS

EEIEQSNIPHLLVDLFAAGTDTTSSTLEWAMTEILRNPEILKKAQAELEEIIGKGNPVEEPDISRLPYLQAIVN
ETFRLHPAVALVVRKVDSDVKLFNYTVPKNAQVLVNLWAIGRDPEIWEEPNTFEPERFLGSEVDVKGRDF
KLIPFGAGRRICPGLPLATRMIHLMLASLIHGFDWKLEAGVSPENTDMEERFGLTLEKAQRLRAIPVRV

>HU05G00563.1-W

ATGGATTACCTGAACTTCCTGTTGTGTCTAGTACTAGCATGGGTTGCAATCTACCTCCTTCTCTCAACT
GCAAAGAAAGGCAAAGCAGCAAATCCTAGAATCCTACCACCAGGTCCTTCCCCTCTACCCATCTTTGG
AAACCTCTTCATTCTGGGCACCAAGCCCCATATCTCCTTGACTGAACTCGCCAGAACCTATGGTCCCC
TCATGAGTCTCCAGCTCGGCCAAGTGCCCACAGTAGTCATTTCTTCAGCTGTTGTGGCCAAGGAAGCC
CTTCAAAAGAATGATATTTCCTTCTCCAGTCGAACAGTTGGAGATGCTGTTTGTGCACTCAGCCATCA
CCAAAGTTCAATAATCTGGTTGCCGGCAGCAGCTCAATGGAGAAATCTGCGCAAAATCTGCAACTCCC
ATGTCTTTTCCAACAGCAGGCTAGAAAGCAGCCAAAATCTCCGAAGAGATAAGGTGAAAGATCTCAT
TTCCCATGTCCAAAAATGCAGTGAATCTGGCATGACAGTGGATATTGGCCAGGCTGCATTTACTACTAC
CTCAAATCTGCTATCAAACACTTTCTTCTCAGTGGATTTGGGAGACCCAACTTCTGAATTTGCTCGCGA
ATTTAAAGGGGTTGTACGAGGTGTTGTGGATGAAATAGGGAAACCCAACTTTGCAGATTACTTCCCTC
TCCTGAGAAACATGGATCCACAAGGCATCAGGCGGCGCATGAGCATCCACTTTGGGAAGATGTTGGG
CCTATTCAATGACATGATTAACCAAAGGTTGCAGGGCAAGAGGCCATCAGAAGGTAATGATGTCTTGG
ATGCATTGATGGCCATCAATCAAGAGAAATCGGAAGAAATCGAGCAATCAAACATTCCTCATCTATTA
GTGGATTTATTCGCAGCCGGAACTGATACGACATCAAGCACCCTTGAATGGGCAATGACAGAAATTCT
CCGCAACCCAGAGATACTCAAGAAAGCACAAGCCGAGCTCGAAGAAATCATCGGAAAAGGAAACCC
AGTCGAAGAACCAGACATTTCCTGGCTCCCATACTTGCAGGCCATCGTCAAGGAAACCTTCCGATTGC
ATCCCGCAGTGCCGCTCGTGGTGCGAAAAGTTGATTCAGATGTCAAGCTCTTCAACTACACAGTGCCC
AAAAACGCACAAGTGTTAGTCAATTTGTGGGCAATTGGGAGAGATCCAGAGATTTGGGAGGAACCCA
ATACATTTGAACCAGAAAGATTTCTGGGATCAGAAGTTGATGTCAAAGGTCGCGATTTTAAGCTGATT
CCCTTTGGAGCTGGCCGTCGGATATGCCCTGGGCTGCCATTGGCAACCAGGATGATCCATTTAATGTTG
GCTTCGCTCATTCATGGGTTTGATTGGAAGCTTGAAGCTGGGGTTTCACCTGAGAACACGGACATGGA
GGAGAGATTTGGGCTCACTCTGGAGAAAGCGCAGCGGCTTCGTGCCATTCCTGTACGTGTATGA

MDYLNFLLCLVLAWVAIYLLLSTAKKGKAANPRILPPGPSPLPIFGNLFILGTKPHISLTELARTYGPLMSLQ
LGQVPTVVISSAVVAKEALQKNDISFSSRTVGDAVCALSHHQSSIIWLPAAAQWRNLRKICNSHVFSNSRL
ESSQNLRRDKVKDLISHVQKCSESGMTVDIGQAAFTTTSNLLSNTFFSVDLGDPTSEFAREFKGVVRGVV
DEIGKPNFADYFPLLRNMDPQGIRRRMSIHFGKMLGLFNDMINQRLQGKRPSEGNDVLDALMAINQEKS
EEIEQSNIPHLLVDLFAAGTDTTSSTLEWAMTEILRNPEILKKAQAELEEIIGKGNPVEEPDISWLPYLQAIV
KETFRLHPAVPLVVRKVDSDVKLFNYTVPKNAQVLVNLWAIGRDPEIWEEPNTFEPERFLGSEVDVKGRD
FKLIPFGAGRRICPGLPLATRMIHLMLASLIHGFDWKLEAGVSPENTDMEERFGLTLEKAQRLRAIPVRV

>HU07G00504.1-R

ATGGGCATCTCCTCCCAAGAAATGGCAAAAAAACCAAAAACTCATCTTACATCAATGGATTACTACAC
TGTATCGTTGGTAGTCCTCCCCATTATTATCTATGTCCTCCATAACTTCCTCAAAAAGTCACCTAAAACC

AAGCTTCCTCCAGGACCAACACCATGGCCAATCATAGGCAACATCCACCTCCTTGGCGCCAAGCCTCA
CCGCTCTGTCGCAGGACTCTCCAAGATTTATGGACCCTTAATGAGCCTCAGGCTCGGGAGCATCACCA
CAATAGTGATATCATCCCCCGAAGTAGCCAAAGAAATGTTCCTAAAACATGACTTAGCCTTTTCTTCTA
GACAAATCCCAGATGCTTCCCGGATAGCAGGCCATGACAAGCTCTCCATTGCGTGGCTTCCCGTATGC
CCCAAATGGCGGGATCTCAGGAAAGTCTTGGCCATCCAGTTGTTCACCAACCAACAGCTCCATGCGA
GTCAGGGCCTCAGGAAGAAGAAAGTGGATGACCTTGTTCAGTTTGTAAAGGGGCGCTCTGAAAAGG
GTCTAGCCATTGATATTGGCAAGGCCGTGGCAACCACCTCACTCAACTTATTATCAAATACATTTTTTTC
AATGGACTTTTCTAGCTATGATTCTTCAGTTTCAGAGGAGTTTAAGGATCTTGCTTGGCATCTGTTGGA
AGAGGCCGCGAGGCCAAATGTGTCGGATTTTTTCCCATTGCTTAGGCCTTTGGATTTGCAAGGGGTGA
GAAGAAGGCAGACTATGTACTTCCACAAAATGATGGGATTTTTTGAGAGAATTGTTGATGAAAGATTG
AGGGATCAAACAGGTGCAAATGAAGATTTTTTAGGCACTCTACTCAAGCTTGTCAAGGAAGATGAGC
TCACCCTTGACGATATCAAACATCTGCTGATTGACTTGTTTATAGCGGGAATAGATACAACTTCCAATG
TATTGCAATGGGCAATGACTGAATTACTACGCCATCCAGAAAAAATGACAAAAGCACAAGCTGAAATC
GATCAAGTCCTAGGCGAAGGTCAATCAAGGTCAATTGAAGAATCTGACATCTCAAATTTGCCTTACAT
ACAAGCAATACTGAAGGAAACATTGAGGTTGCACCCACCAGCCCCTTTTTTGGTACCCCATAAGGCTA
ACTGTGAGGTACAATTATGTGGCTACTATGTGCCAAAAAATGCACAAGTGTGGGTGAATCTATGGTGC
CTCAGCCATGATCCAAATGTTTGGGTAAACCCCGAATCATTTATGCCAGAAAGGTTCTTGGAGAGTGA
AATTGATTTCAAAGGCCGAAATTTTGAGCTCATACCCTTCGGAACAGGAAGAAGGATGTGTCCTGGA
ATGCCGCTAGCTCATAGGATGGCACATCTAATGTTGGCAACTCTTCTTCATTCATTCAATTGGAAGCTT
GATGGTCAGTTAAATTCAGAAGACATAGATATCGAAGAAATGTTTGGGATTACGTTGGCAAAAGCTCA
GCCACTCGAAGCTATCCCGCTCTCAAGATGGCTTTGA

MGISSQEMAKKPKTHLTSMDYYTVSLVVLPIIIYVLHNFLKKSPKTKLPPGPTPWPIIGNIHLLGAKPHRSV
AGLSKIYGPLMSLRLGSITTIVISSPEVAKEMFLKHDLAFSSRQIPDASRIAGHDKLSIAWLPVCPKWRDLR
KVLAIQLFTNQQLHASQGLRKKKVDDLVQFVKGRSEKGLAIDIGKAVATTSLNLLSNTFFSMDFSSYDSSV
SEEFKDLAWHLLEEAARPNVSDFFPLLRPLDLQGVRRRQTMYFHKMMGFFERIVDERLRDQTGANEDFL
GTLLKLVKEDELTLDDIKHLLIDLFIAGIDTTSNVLQWAMTELLRHPEKMTKAQAEIDQVLGEGQSRSIEE
SDISNLPYIQAILKETLRLHPPAPFLVPHKANCEVQLCGYYVPKNAQVWVNLWCLSHDPNVWVNPESFM
PERFLESEIDFKGRNFELIPFGTGRRMCPGMPLAHRMAHLMLATLLHSFNWKLDGQLNSEDIDIEEMFGIT
LAKAQPLEAIPLSRWL

>HU07G00504.1-W

ATGGGCATCTCCTCCCAAGAAATGGCAAAAAAACCAAAAACTCATCTTACATCAATGGATTACTACAC
TGTATCGTTGGTAGTCCTCCCCATTATTATCTATGTCCTCCATAACTTCCTCAAAAAGTCACCTAAAACC
AAGCTTCCTCCAGGACCAACACCATGGCCAATCATAGGCAACATCCACCTCCTTGGCGCCAAGCCTCA
CCGCTCTGTCGCAGGACTCTCCAAGATTTATGGACCCTTAATGAGCCTCAGGCTCGGGAGCATCACCA
CAATAGTGATATCATCCCCCGATGTAGCCAAAGAAATGTTCCTAAAACATGACTTAGCCTTTTCTTCTA
GACAAATCCCAGATGCTTCCCGGATAGCAGGCCATGACAAGCTCTCCATTGCGTGGCTTCCCGTATGC
CCCAAATGGCGGGATCTCAGGAAAGTCTTGGCCATCCGGTTGTTCACCAACCAACAGCTCCATGCGA

GTCAGGGCCTCAGGAAGAAGAAAGTGGATGACCTTGTTCAGTTTGTAAAGGGGCGCTCTGAAAAGG
GTCTAGCCATTGATATTGGCAAGGCCGTGGCAACCACCTCACTCAACTTATTATCAAATACATTTTTTTC
AATGGACTTTTCTAGCTATGATTCTTCAGTTTCAGAGGAGTTTAAGGATCTTGCTTGGCATCTGTTGGA
AGAGGCCGCGAGGCCAAATGTGTCGGATTTTTTCCCATTGCTTAGGCCTTTGGATTTGCAAGGGGTGA
GAAGAAGGCAGACTATGTACTTCCACAAAATGATGGGATTTTTTGAGAGAATTGTTGATGAAAGATTG
AGGGATCAAACAGGTGCAAATGAAGATTTTTTAGGCACTCTACTCAAGCTTGTCAAGGAAGATGAGC
TCACCCTTGACGATATCAAACATCTGCTGATTGACTTGTTTATAGCGGGAATAGATACAACTTCCAATG
TATTGCAATGGGCAATGACTGAATTACTACGCCATCCAGAAAAAATGACAAAAGCACAAGCTGAAATC
GATCAAGTCCTAGGCGAAGGTCAATCAAGGTCAATTGAAGAATCTGACATCTCAAATTTGCCTTACAT
ACAAGCAATACTGAAGGAAACATTGAGGTTGCACCCACCAGCCCCTTTTTTGGTACCCCATAAGGCTG
ACTGTGAGGTACAATTATGTGGCTACTATGTGCCAAAAAATGCACAAGTGTGGGTGAATCTATGGTGC
CTCAGCCATGATCCAAATGTTTGGGTAAACCCCGAATCATTTATGCCAGAAAGGTTCTTGGAGAGTGA
AATTGATTTCAAAGGCCGAAATTTTGAGCTCATACCCTTCGGAACAGGAAGAAGGATGTGTCCTGGA
ATGCCGCTAGCTCATAGGATGGCACATCTAATGTTGGCAACTCTTCTTCATTCATTCAATTGGAAGCTT
GATGGTCAGTTAAATTCAGAAGACATAGATATCGAAGAAATGTTTGGGATTACGTTGGCAAAAGCTCA
GCCACTCGAAGCTATCCCGCTCTCAAGATGGCTTTGA

MGISSQEMAKKPKTHLTSMDYYTVSLVVLPIIIYVLHNFLKKSPKTKLPPGPTPWPIIGNIHLLGAKPHRSV
AGLSKIYGPLMSLRLGSITTIVISSPDVAKEMFLKHDLAFSSRQIPDASRIAGHDKLSIAWLPVCPKWRDLR
KVLAIRLFTNQQLHASQGLRKKKVDDLVQFVKGRSEKGLAIDIGKAVATTSLNLLSNTFFSMDFSSYDSSV
SEEFKDLAWHLLEEAARPNVSDFFPLLRPLDLQGVRRRQTMYFHKMMGFFERIVDERLRDQTGANEDFL
GTLLKLVKEDELTLDDIKHLLIDLFIAGIDTTSNVLQWAMTELLRHPEKMTKAQAEIDQVLGEGQSRSIEE
SDISNLPYIQAILKETLRLHPPAPFLVPHKADCEVQLCGYYVPKNAQVWVNLWCLSHDPNVWVNPESFM
PERFLESEIDFKGRNFELIPFGTGRRMCPGMPLAHRMAHLMLATLLHSFNWKLDGQLNSEDIDIEEMFGIT
LAKAQPLEAIPLSRWL

>HU07G00784.1-R

ATGGTGCTGGTGCTCCTCCTGGTTGTAGTTTGCTCTTTCATCTTCTTCCTTGTAATTCTCCCACATCGCG
AAAAACATGGAAAATTCAATCTCCCTCCTGGACCCATAGGCCTCCCCCTCATCGGAAACCTCCACCAG
TTCGATTCCTCGGCCCCTCACCTCTACTTCTCCAAGCTTGCCAAGATCTACGGCCCGATCTTGTCCTTG
CGATTCGGGTGTAGATCAGCGGTGGTGATTCAATCAGCTGCTCTGGCGAAGGAGGTCCTGAAAACAC
AGGATCACAACTTCTGTACGAGGCCAACTCTGGTGGCTCAGCAGAGACTGTCTTACAATGGTTCGGA
CCTTGTTTTGCGCCCTACAATGACTGTTTCAGAGAGCTTAGGAAATTAAGTGTTGTTCATTTGTTTAGC
TCCAAGAAGGTTCAGTCTTCCGCTCCCATTCGACAAGAAGAAGTTTCTAGAATGATCCAGAAGATCAC
TTCTCTGTATTCTGCTTCGAAAGTCGTTAACTTGAGCAACTTGCTTATGGCTCTTACCAGCTCCATTATT
TGCAGGGTTGCTTTTGGCAAGAGATATGAGGATGAAGGTGTTGAGAGAAGCAGATTTCATGAGCTCC
TGAATGAAGTTCAAGCTATGGCGGCAGCTCCTTTCTTCACAGATTATTTCCCTTTCATAGGAAGCTGGC
TTGATAAGCTAACTGGACTGTCATCCAGGCTTGAGAAGGTGTTTGAGAATATGGATGAATTTTATGATG
AAATCATTAATGATCACCTTGATCCCAATAGGCCTAAAGATGATAGTCGTGAGGATATCATTGATGTTCT

TCTGCAGCTCCGGAAGGAACGTTGCTTCTCTTTTGAGCTCACCTTGGACCACATCAAAGCGCTATTAC
TGGATATCATTGTAGCAGGAACTGATACAAGTGCAGCAATTGTAGTTTGGGCAATGACGGAGTTAATG
AAAAACCCAACTCTGATGAGGAAAGTTCAAGAAGAGCTTAGAACCTTGATTCAAGACAAGACATTTA
TCGACGAGAGTGATCTCTCAAGGTTAACTTACCTCAAAGCTATAGTGAAGGAAACACTTCGACTGCA
CAATGCAGGTCCATTATTGGTTGTTCGGGAAGTCATCCAAAAATGTAGTGTACAAGGGTATGATATTCT
CCCAAAAAGCTTGGTATTTGTTAGCATATGGGAGATCGGTAGAGACCCCAACTTTTGGACTGATCCTG
AAATATTCATGCCTGAAAGATTTCTCAGGAGCTCAATTGATTTCAGAGGGCAAGATTTTGAATTGATAC
CTTTCGGAGCAGGTAGAAGAATATGTCCTGGACTGCTTCTCGGTTTAGCTAATGTGGAGCTTGCACTT
GCCAATTTATTATATACTTTCGACTGGAAATTGCCAGTTGGAATGACGGAGGAAGATATTGACTTTGAT
GTATTGCCTGGAATCACAATGCACAAAAAGAATCCACTACGCCTCGTAGCAAAGAAGTTCTCTCATGT
AAACTAA

MVLVLLLVVVYSFIFFLVILPHREKHGKFNLPPGPIGLPLLGNLHQFDSSAPHLYFSKLAKIYGPILSLRFGS
KKVQSSAPIRQEEVSRMIQKITSLSSASKVVNLSNLLMALTSSIICRVAFGKRYEDEGVERSRFHELLNEVQ
AMAATPFFTDYFPFIGSWLDKLTGLSSRLEKVFENMDEFYDEIINDHLDPNRPKDDSREDIIDVLLQLRKE
RCFSFELTLDHIKAVLLDIIVAGTDTSATIVVWAMTELMKNPTMMRKVQEELRTLIQDKTFIDESDLSRLTY
LKAIVKETLRLHNAGPLLVVREVIQKCSVQGYDILPKSLVFVSIWEIGRDPNFWTDPEIFMPERFLGSSIDF
RGQDFELIPFGAGRRICPGLLLGLANVELALANLLYTFDWKLPVGMTEEDIDFDVLPGITMHKKNPLCLV
AKKFSHVN

>HU07G00784.1-W

ATGGTGCTGGTGCTCCTCCTGGTTGTAGTTTGCTCTTTCATCTTCTTCCTTGTAATTCTCCCACATCGCG
AAAAACATGGAAAATTCAATCTCCCTCCTGGACCCATAGGCCTCCCCCTCATCGGAAACCTCCACCAG
TTCGATTCCTCGGCCCCTCACCTCTACTTCTCCAAGCTTGCCAAGATCTACGGCCCGATCTTGTCCTTG
CGATTCGGGTGTAGATCAGCGGTGGTGATTCAATCAGCTGCTCTGGCGAAGGAGGTCCTGAAAACAC
AGGATCACAACTTCTGTACGAGGCCAACTCTGGTGGCTCAGCAGAGACTGTCTTACAATGGTTCGGA
CCTTGTTTTGCGCCCTACAATGACTGTTTCAGAGAGCTTAGGAAATTAAGTGTTGTTCATTTGTTTAGC
TCCAAGAAGGTTCAGTCTTCCGCTCCCATTCGACAAGAAGAAGTTTCTAGAATGATCCAGAAGATCAC
TTCTCTGTATTCTGCTTCGAAAGTCGTTAACTTGAGCAACTTGCTTATGGCTCTTACCAGCTCCATTATT
TGCAGGGTTGCTTTTGGCAAGAGATATGAGGATGAAGGTGTTGAGAGAAGCAGATTTCATGAGCTCC
TGAATGAAGTTCAAGCTATGGCGGCAGCTCCTTTCTTCACAGATTATTTCCCTTTCATAGGAAGCTGGC
TTGATAAGCTAACTGGACTGTCATCCAGGCTTGAGAAGGTGTTTGAGAATATGGATGAATTTTATGATG
AAATCATTAATGATCACCTTGATCCCAATAGGCCTAAAGATGATAGTCGTGAGGATATCATTGATGTTCT
TCTGCAGCTCCGGAAGGAACGTTGCTTCTCTTTTGAGCTCACCTTGGACCACATCAAAGCGCTATTAC
TGGATATCATTGTAGCAGGAACTGATACAAGTGCAGCAATTGTAGTTTGGGCAATGACGGAGTTAATG
AAAAACCCAACTCTGATGAGGAAAGTTCAAGAAGAGCTTAGAACCTTGATTCAAGACAAGACATTTA
TCGACGAGAGTGATCTCTCAAGGTTAACTTACCTCAAAGCTATAGTGAAGGAAACACTTCGACTGCA
CAATGCAGGTCCATTATTGGTTGTTCGGGAAGTCATCCAAAAATGTAGTGTACAAGGGTATGATATTCT
CCCAAAAAGCTTGGTATTTGTTAGCATATGGGAGATCGGTAGAGACCCCAACTTTTGGACTGATCCTG

AAATATTCATGCCTGAAAGATTTCTCAGGAGCTCAATTGATTTCAGAGGGCAAGATTTTGAATTGATAC
CTTTCGGAGCAGGTAGAAGAATATGTCCTGGACTGCTTCTCGGTTTAGCTAATGTGGAGCTTGCACTT
GCCAATTTATTATATACTTTCGACTGGAAATTGCCAGTTGGAATGACGGAGGAAGATATTGACTTTGAT
GTATTGCCTGGAATCACAATGCACAAAAAGAATCCACTACGCCTCGTAGCAAAGAAGTTCTCTCATGT
AAACTAA

MVLVLLLVVVYSFIFFLVILPHREKHGKFNLPPGPIGLPLLGNLHQFDSSAPHLYFSKLAKIYGPILSLRFGS
KKVQSSAPIRQEEVSRMIQKITSLSSASKVVNLSNLLMALTSSIICRVAFGKRYEDEGVERSRFHELLNEVQ
AMAATPFFTDYFPFIGSWLDKLTGLSSRLEKVFENMDEFYDEIINDHLDPNRPKDDSREDIIDVLLQLRKE
RCFSFELTLDHIKAVLLDIIVAGTDTSATIVVWAMTELMKNPTMMRKVQEELRTLIQDKTFIDESDLSRLTY
LKAIVKETLRLHNAGPLLVVREVIQKCSVQGYDILPKSLVFVSIWEIGRDPNFWTDPEIFMPERFLGSSIDF
RGQDFELIPFGAGRRICPGLLLGLANVELALANLLYTFDWKLPVGMTEEDIDFDVLPGITMHKKNPLCLV
AKKFSHVN

>HU09G01023.1-R

ATGGAATATTACTCCATATCAATTTTGCTCATCCCCGTAATCCTCATTTTCACCCTTCATCACTTGCTCAA
AAACTCCCCTAAAACCAAGCTTCCTCCAGGCCCAAAACCATGGCCAATCATAGGCAACATCCACCTC
ATCGGCTCCAAGCCACACCGCTCTGTGGCAGACCTCTCCAAGACTTATGGACCCTTGATGACCCTCAG
GCTTGGGAGCATCACCACGATAGTCATATCATCTCCAGATGTAGCCAAGGAAATGTTCTTAAAACACG
ACGTAGACTTTGCCTCTAGGCAAGTCCCGGACTCTGCCTGGGCCGTAGACCATGCAAAGTTCTCAATG
GTGTGGCTCCCTGTATGCCCCAAATGGCGGGAGATCAGGAAGATCTCAGCCATCCAGCTGTTCACCAA
CCAACGGTTGGATGCAAGCCAGGGCCTTAGGAAGGAGAAAGTCAATGAGCTGGTAAAGTTTGTAAA
AGGTTGTTGTGAAAAGGGTCTGGCCGTTGACATTGGCAAGGCCGCGTTCACAACCGCGCTTAACTTG
TTATCAAACACATTTTTTTCAATGAATTTGTCTGCATATGATTCTTCAGGCTCGCAGGAGTTCAAGGATC
TTGCTTGGCATATAATGGAAGAGATTGGACGGCCAAACATATCGGATTTCTTCCCATTGGTTAAGGCCT
TGGACTTGCAAGGTGTGAAAAGAAGCTTAAGTACTTCTTTTAGCAAAATGCTCGAAGTTTTCGACAA
GATCATCGATGAAAGATTAAGCGATCAGATTGGTTCAAAGGACGATGTTCTGGACACCCTGCTCAGTC
TTGTGAAGCAAAATGAGTTGACCCTTGACGATGTCAGACACATGCTCATAGATTTATTTGTTGCGGGG
ACGGATACAACCTCCAACACAATAGAATGGGCTTTGACAGAACTACTACGCCATCCAGAAAAAATGG
CAAAGGCACAAGCCGAAATGGAACAACTTCTAGGCAAGGGTCAATCAAGATCAATACAAGAATCAG
ATATCTCAAAACTACCTTACATTCAAGCGATAGTGAAAGAGACAATGAGATTGCATCCTACTGTCCCTT
TTTTGGTACCCCATAAGGCTACGACAAATGTAGAATTATGTGGCTATTGGGTGCCCAAAAATGCACAA
GTATGGGTAAACGTATGGTCTATGGGGCGAGATCCAAGTGTTTGGCCAGATTCTGAATCATTTACACCC
GAAAGATTTTTGGAAAGCGAAATTGATATTAAAGGCCAAGATTTCGAGCTAATACCCTTTGGAGCAGG
AAGAAGGATATGTCCCGGAATGCCACTGGCTTATAGAATGCTACATCTAA

MEYYSISILLIPVILIFTLHHLLKNSPKTKLPPGPKPWPIIGNIHLIGSKPHRSVADLSKTYGPLMTLRLGSITT
IVISSPDVAKEMFLKHDVDFASRQVPDSAWAVDHAKFSMVWLPVCPKWREIRKISAIQLFTNQRLDASQG
LRKEKVNELVKFVKGCCEKGLAVDIGKAAFTTALNLLSNTFFSMNLSAYDSSGSQEFKDLAWHIMEEIGR

PNISDFFPLVKALDLQGVKRSLSTSFSKMLEVFDKIIDERLSDQIGSKDDVLDTLLSLVKQNELTLDDVRH
MLIDLFVAGTDTTSNTIEWALTELLRHPEKMAKAQAEMEQLLGKGQSRSIQESDISKLPYIQAIVKETMRL
HPTVPFLVPHKATTNVELCGYWVPKNAQVWVNVWSMGRDPSVWPDSESFTPERFLESEIDIKGQDFELIP
FGAGRRICPGMPLAYRMLHL

>HU09G01023.1-W

ATGGAATATTACTCCATATCAATTTTGCTCATCCCCGTAATCCTCATTTTCACCCTTCATCACTTGCTCAA
AAACTCCCCTAAAACCAAGCTTCCTCCAGGCCCAAAACCATGGCCAATCATAGGCAACATCCACCTC
ATCGGCTCCAAGCCACACCGCTCTGTGGCAGACCTCTCCAAGACTTATGGACCCTTGATGACCCTCAG
GCTTGGGAGCATCACCACGATAGTCATATCATCTCCAGATGTAGCCAAGGAAATGTTCTTAAAACACG
ACGTAGACTTTGCCTCTAGGCAAGTCCCGGACTCTGCCTGGGCCGTAGACCATGCAAAGTTCTCAATG
GTGTGGCTCCCTGTATGCCCCAAATGGCGGGAGATCAGGAAGATCTCAGCCATCCAGCTGTTCACCAA
CCAACGGTTGGATGCAAGCCAGGGCCTTAGGAAGGAGAAAGTCAATGAGCTGGTAAAGTTTGTAAA
AGGTTGTTGTGAAAAGGGTCTGGCCGTTGACATTGGCAAGGCCGCGTTCACAACCGCGCTTAACTTG
TTATCAAACACATTTTTTTCAATGAATTTGTCTGCATATGATTCTTCAGGCTCGCAGGAGTTCAAGGATC
TTGCTTGGCATATAATGGAAGAGATTGGACGGCCAAACATATCGGATTTCTTCCCATTGGTTAAGGCCT
TGGACTTGCAAGGTGTGAAAAGAAGCTTAAGTACTTCTTTTAGCAAAATGCTCGAAGTTTTCGACAA
GATCATCGATGAAAGATTAAGCGATCAGATTGGTTCAAAGGACGATGTTCTGGACACCCTGCTCAGTC
TTGTGAAGCAAAATGAGTTGACCCTTGACGATGTCAGACACATGCTCATAGATTTATTTGTTGCGGGG
ACGGATACAACCTCCAACACAATAGAATGGGCTTTGACAGAACTACTACGCCATCCAGAAAAAATGG
CAAAGGCACAAGCCGAAATGGAACAACTTCTAGGCAAGGGTCAATCAAGATCAATACAAGAATCAG
ATATCTCAAAACTACCTTACATTCAAGCGATAGTGAAAGAGACAATGAGATTGCATCCTACTGTCCCTT
TTTTGGTACCCCATAAGGCTACGACAAATGTAGAATTATGTGGCTATTGGGTGCCCAAAAATGCACAA
GTATGGGTAAACGTATGGTCTATGGGGCGAGATCCAAGTGTTTGGCCAGATTCTGAATCATTTACACCC
GAAAGATTTTTGGAAAGCGAAATTGATATTAAAGGCCAAGATTTCGAGCTAATACCCTTTGGAGCAGG
AAGAAGGATATGTCCCGGAATGCCACTGGCTTATAGAATGCTACATCTAA

MEYYSISILLIPVILIFTLHHLLKNSPKTKLPPGPKPWPIIGNIHLIGSKPHRSVADLSKTYGPLMTLRLGSITT
IVISSPDVAKEMFLKHDVDFASRQVPDSAWAVDHAKFSMVWLPVCPKWREIRKISAIQLFTNQRLDASQG
LRKEKVNELVKFVKGCCEKGLAVDIGKAAFTTALNLLSNTFFSMNLSAYDSSGSQEFKDLAWHIMEEIGR
PNISDFFPLVKALDLQGVKRSLSTSFSKMLEVFDKIIDERLSDQIGSKDDVLDTLLSLVKQNELTLDDVRH
MLIDLFVAGTDTTSNTIEWALTELLRHPEKMAKAQAEMEQLLGKGQSRSIQESDISKLPYIQAIVKETMRL
HPTVPFLVPHKATTNVELCGYWVPKNAQVWVNVWSMGRDPSVWPDSESFTPERFLESEIDIKGQDFELIP
FGAGRRICPGMPLAYRMLHL

>HU11G01017.1-R

ATGGTTGGTGGTTATCACATCCCAGCAGGCACTTGGCTCTTCGTTAATCTTTACAAGTTACACAGAGAC
CCTCAAGTGTGGCCAGAACCCCTGGAATTCCGTCCTGAACGGTTTCTCACAACTCACAAGGATTACG
ATGTCAGAGGGCAAAATTTTGCACTCATACCTTTTGGAGTTGGTAGAAGAATGTGTCCTGGAGTATCT

TTTGCCCTTCAGGCCATGCGATTCATAATTGCTAACCTACTTCATGGTTTTGAGATCTCAACTCCATATG
GTGAAAAAGTTGACATGACAGAGGGGTTTGGACTAACCCACCTCAAAGCCTCC

MVGGYHIPAGTWLFVNLYKLHRDPQVWPEPLEFRPERFLTTHKDYDVRGQNFALIPFGVGRRMCPGVSF
ALQAMRFIIANLLHGFEISTPYGEKVDMTEGFGLTHLKAS

>HU11G01017.1-W

ATGGTTGGTGGTTATCACATCCCAGCAGGCACTTGGCTCTTCGTTAATCTTTACAAGTTACACAGAGAC
CCTCAAGTGTGGCCAGAACCCCTGGAATTCCGTCCTGAACGGTTTCTCACAACTCACAAGGATTACG
ATGTCAGAGGGCAAAAGTTTGCACTCATACCTTTTGGAGTTGGTAGAAGAATGTGTCCTGGAGTATCT
TTTGCCCTTCAGGCCATGCGATTCATAGTTGCTAACCTACTTCATGGTTTTGAGATCTCAACTCCATCTG
GTGAAAAAGTTGACATGACAGAGGGGTTTGGACTAACCCACCTCAAAGCCTCC

MVGGYHIPAGTWLFVNLYKLHRDPQVWPEPLEFRPERFLTTHKDYDVRGQKFALIPFGVGRRMCPGVSF
ALQAMRFIVANLLHGFEISTPSGEKVDMTEGFGLTHLKAS

>HU11G01870.1-R

ATGGACACATACACAGTTGTACTTGCACAACTACTACTGGCCTTCACAATCTTCGTCCTCTTCTTCAAA
CCCAGAAACTCCAACCTCCCTCCCGGCCCGAAACCATGGCCGATCATCGGCAACCTGCTCCACCTCG
GCGACAAGCCCCACCGCCGCCTCACCGAACTTTCCAGAACCTACGGCCCCATAATGTTCATCAAGCTA
GGGTTCGTACCCACCATCGTCATCTCCTCCCCGGAAATCGCCAAACAAGTCTTCCTCAACCATGATCT
AGACTTTTCCGGCAGGAATGTCCCCGCCGGCGGCAAGGTTTCCGACCACCACAAACTCTCAATCGGG
TGGTTACCCGTTTGCCCCAAATGGCGGACCCTCCGGAAAATCGCGACGATTCATTTGTTCACCGGAAA
AGCCCTGGACGCCGGGGAGAGTCTCCGGCGAACGGTGGTGGATAAGCTAATTGGGTTCGTCGGAGAC
TGTTGTAAAACGGGTCGACCCGTTGATATTGGCAAGGCTGTGTTTGTTACGTCGATTAACTTATTGTCG
AACACTCTTTTTTCGATCGATTTGGCTAGTTATGGTGATTGGAAATCGGATGATTTTAAGAAGATTTTGT
GGGAGTTTTGGGAGGAAGCTGGGAAGCCCAATTTGACTGATGCTTTTCCGGTGCTGGGGTGGTTGGA
TTTGCAGAGAGCATCAAAGAGAGTTGGAAGGTGTTTTGATAGGGTTTTGGGGG.TTTTTGAGGAGATT
ATCGAGCGAAGAATTAGGGATTCCAAGGGTTTTGGGGATGATGGTTTGGGGAATTTTCTGAAGCTTGT
TGAAGAAAATGAATGGAGCCTTGATGATGTCAAACATTTTCTCTTCGATTTGTTTATAGCAGGAACAG
ACACTACCTCAACTACAGTAGAGTGGGCGATGACAGAATTACTACGACACCCAGAAAAGATGGCGAA
GGCCCAAAGTGAAATCGTTCAAGTAATTGGCGATAATGGTAGGCCAGTTCAAGAACTTGACATCCCAA
AACTTCCCTATATACAAGCAATAGCGAAAGAGACATTGAGAATGCACCCGCCAGCCCCTTTCCTCTTA
CCCCACAAGGCCGAAAAAAATGTACAATTAGACGGCTACCTAGTGCCTAAGAATGCGCAAGTATGGG
TCAATGTATGGGGAATTGGTCACGACCCTAGTGTGTGGCCAAACCCAGATTCATTTACACCCGAAAGA
TTTTTGAACGGGGGTGTTGATGCCAAAGGTAGAGATTTCGAGTTCATACCATTCGGTGAAGGAAGAA
GGATATGTCTTGGGATGCCATTGGCATATCGGATGTTGCATCTAGTGTTGGCTAATTTGCTTTGTTCGTT
CGATTGGAAGCCTAGCGATGGAGTCAGCCCCCATGATGTAGGCGTGGATGATAAGTTTGGAATTGTGG
TACAAAGAAACCCTCCATTAGAAGCTATTCCTTGTGCCATATGA

MDTYTVVLAQLLLAFTIFVLFFKPRNSNLPPGPKPWPIIGNLLHLGDKPHRRLTELSRTYGPIMFIKLGFVP
TIVISSPEIAKQVFLNHDLDFSGRNVPAGGKVSDHHKLSIGWLPVCPKWRTLRKIATIHLFTGKALDAGES
LRRTVVDKLIGFVGDCCKTGRPVDIGKAVFVTSINLLSNTLFSIDLASYGDWKSDDFKKILWEFWEEAGK
PNLTDAFPVLGWLDLQRASKRVGRCFDRVLGVFEEIIERRIRDSKGFGDDGLGNFLKLVEENEWSLDDVK
HFLFDLFIAGTDTTSTTVEWAMTELLRHPEKMAKAQSEIVQVIGDNGRPVQELDIPKLPYIQAIAKETLRM
HPPAPFLLPHKAEKNVQLDGYLVPKNAQVWVNVWGIGHDPSVWPNPDSFTPERFLNGGVDAKGRDFEFI
PFGEGRRICLGMPLAYRMLHLVLANLLCSFDWKPSDGVSPHDVGVDDKFGIVVQRNPPLEAIPCAI

>HU11G01870.1-W

ATGGACACATACACAGTTGTACTTGCACAACTACTACTGGCCTTCACAATCTTCGTCCTCTTCTTCAAA
CCCAGAAACTCCAACCTCCCTCCCGGCCCGAAACCATGGCCGATCATCGGCAACCTGCTCCACCTCG
GCGACAAGCCCCACCGCCGCCTCACCGAACTTTCCAGAACCTACGGCCCCATAATGTTCATCAAGCTA
GGGTTCGTACCCACCATCGTCATCTCCTCCCCGGAAATCGCCAAACAAGTCTTCCTCAACCATGATCT
AGACTTTTCCGGCAGGAATGTCCCCGCCGGCGGCAAGGTTTCCGACCACCACAAACTCTCAATCGGG
TGGTTACCCGTTTGCCCCAAATGGCGGACCCTCCGGAAAATCGCGACGATTCATTTGTTCACCGGAAA
AGCCCTGGACGCCGGGGAGAGTCTCCGGCGAACGGTGGTGGATAAGCTAATTGGGTTCGTCGGAGAC
TGTTGTAAAACGGGTCGACCCGTTGATATTGGCAAGGCTGTGTTTGTTACGTCGATTAACTTATTGTCG
AACACTCTTTTTTCGATCGATTTGGCTAGTTATGGTGATTGGAAATCGGATGATTTTAAGAAGATTTTGT
GGGAGTTTTGGGAGGAAGCTGGGAAGCCCAATTTGACTGATGCTTTTCCGGTGCTGGGGTGGTTGGA
TTTGCAGAGAGCATCAAAGAGAGTTGGAAGGTGTTTTGATAGGGTTTTGGGGG.TTTTTGAGGAGATT
ATCGAGCGAAGAATTAGGGATTCCAAGGGTTTTGGGGATGATGGTTTGGGGAATTTTCTGAAGCTTGT
TGAAGAAAATGAATGGAGCCTTGATGATGTCAAACATTTTCTCTTCGATTTGTTTATAGCAGGAACAG
ACACTACCTCAACTACAGTAGAGTGGGCGATGACAGAATTACTACGACACCCAGAAAAGATGGCGAA
GGCCCAAAGTGAAATCGTTCAAGTAATTGGCGATAATGGTAGGCCAGTTCAAGAACTTGACATCCCAA
AACTTCCCTATATACAAGCAATAGCGAAAGAGACATTGAGAATGCACCCGCCAGCCCCTTTCCTCTTA
CCCCACAAGGCCGAAAAAAATGTACAATTAGACGGCTACCTAGTGCCTAAGAATGCGCAAGTATGGG
TCAATGTATGGGGAATTGGTCACGACCCTAGTGTGTGGCCAAACCCAGATTCATTTACACCCGAAAGA
TTTTTGAACGGGGGTGTTGATGCCAAAGGTAGAGATTTCGAGTTCATACCATTCGGTGAAGGAAGAA
GGATATGTCTTGGGATGCCATTGGCATATCGGATGTTGCATCTAGTGTTGGCTAATTTGCTTTGTTCGTT
CGATTGGAAGCCTAGCGATGGAGTCAGCCCCCATGATGTAGGCGTGGATGATAAGTTTGGAATTGTGG
TACAAAGAAACCCTCCATTAGAAGCTATTCCTTGTGCCATATGA

MDTYTVVLAQLLLAFTIFVLFFKPRNSNLPPGPKPWPIIGNLLHLGDKPHRRLTELSRTYGPIMFIKLGFVP
TIVISSPEIAKQVFLNHDLDFSGRNVPAGGKVSDHHKLSIGWLPVCPKWRTLRKIATIHLFTGKALDAGES
LRRTVVDKLIGFVGDCCKTGRPVDIGKAVFVTSINLLSNTLFSIDLASYGDWKSDDFKKILWEFWEEAGK
PNLTDAFPVLGWLDLQRASKRVGRCFDRVLGVFEEIIERRIRDSKGFGDDGLGNFLKLVEENEWSLDDVK
HFLFDLFIAGTDTTSTTVEWAMTELLRHPEKMAKAQSEIVQVIGDNGRPVQELDIPKLPYIQAIAKETLRM
HPPAPFLLPHKAEKNVQLDGYLVPKNAQVWVNVWGIGHDPSVWPNPDSFTPERFLNGGVDAKGRDFEFI
PFGEGRRICLGMPLAYRMLHLVLANLLCSFDWKPSDGVSPHDVGVDDKFGIVVQRNPPLEAIPCAI

DODAα

> HU03G01342.1-R

ATGGGTGTTGGCAAAGAAGTGTCGTTCAAGGAGACTTTCTATGTGTCTCATGGGAATCCGGCCATGTTGGC
CGATGTGTCGTTCATAGCACGGAACTTCCTGCTGGGGTGGAAGACGAATGTCTTCCCCATCAAACCCAAGT
CGATCCTGGTTGTCTCTGCTCACTGGGAGACTGATGTGCCTTCTGTATCTGCCGGTGAACATCCTGATGTCA
TTTACGATTTCAGCGATGTTCCTGACTGTATGTTCCAGATGAAGTACCCAGCTCTAGGGTCACCAAAACTG
GCCAAAAGGGTGCAGGAGCTACTGATAGCAGGAGGGTTCAAGACAGCGAGCCTAGACGAGAGTCGTGGG
TTCGACCACAGCTCATGGGTGCCCCTGAGCCTCATGTACCCTGAGGCTGACATCCCGGTGTGCCAGCTCTC
AGTCCAGCCTCACCTAAGCGCGAGCCACCACTTCGACATAGGGAGGGCTTTGGCTCCTCTCAAGGAGGAA
GGGGTCCTGTTCATTGGGTCTGGGGGTGCAGTGCACCCTTCTGATGACACCCCACACTGGTCTGATGGGGT
TGCCCCTTGGGCTGCTGAGTTTGATCAATGGCTTGAGGATGCTCTCATTAATGGAAGGTACGATGATGTGAA
TAATTATCAAACAAAAGCACCTTCTGGGTGGAAAATAGCACATCCAATTCCAGAACACTTTTTACCGTTGC
ATGTAGCCATGGGTGCAGCTGGTGAAAAATCAAAGGCAGAGCTCATTTATCGTACGTGGGATCATGGTACT
CTTGGCTATGCCTCCTACAAGTTCACTTCCATCTGA

MGVGKEVSFKETFYVSHGNPAMLADVSFIARNFLLGWKTNVFPIKPKSILVVSAHWETDVPSVSAGEHPD
VIYDFSDVPDCMFQMKYPALGSPKLAKRVQELLIAGGFKTASLDESRGFDHSSWVPLSLMYPEADIPVCQ
LSVQPHLSASHHFDIGRALAPLKEEGVLFIGSGGAVHPSDDTPHWSDGVAPWAAEFDQWLEDALINGRYD
DVNNYQTKAPSGWKIAHPIPEHFLPLHVAMGAAGEKSKAELIYRTWDHGTLGYASYKFTSI

> HU03G01342.1-W

ATGGGTGTTGGCAAAGAAGTGTCGTTCAAGGAGACTTTCTATGTGTCTCATGGGAATCCGGCCATGTTGGC
CGATGTGTCGTTCATAGCGCGGAACTTCCTGCTGGGGTGGAAGAAGAATGTCTTCCCCATCAAACCCAAGT
CGATCCTGGTTGTCTCTGCTCACTGGGAGACTGATGTGCCTTCTGTATCTGCCGGTGAACATCCTGATGTCA
TTTACGATTTCAGCGATGTTCCTGACTGCATGTTCCAGATGAAGTACCCAGCTCCAGGGTCACCAAAACTG
GCCAAAAGAGTGCAGGAGCTACTGATAGCAGGAGGGTTCAAGACAGCGAGCCTAGACGAGAGTCGTGGG
TTCGACCACAGCTCATGGGTGCCCCTGAGCCTCATGTACCCTGAGGCTGACATCCCGGTGTGCCAGCTCTC
AGTCCAGCCTCACCTAAGCGCGAGCCACCACTTCGACGTAGGGAGGGCTTTGGCTCCTCTCAAGGAGGAA
GGGGTCCTGTTCATTGGGTCTGGGGGTGCAGTGCACCCTTCTGATGACACCCCACACTGGTTTGATGGGGT
CGCCCCTTGGGCTGCTGAGTTTGATCAATGGCTTGAGGATGCTCTCATTAATGGAAGGTACGATGATGTGA
ATAATTATCAAACAAAAGCACCTTCTGGGTGGAAAATAGCACATCCAATTCCAGAACACTTTTTACCGTTG
CATGTAGCCATGGGTGCAGCTGGTGAAAAATCAAAGGCAGAGCTCATTTATCGTACGTGGGATCATGGTAC
TCTTGGCTATGCCTCCTACAAGTTCACTTCCATCTGA

MGVGKEVSFKETFYVSHGNPAMLADVSFIARNFLLGWKKNVFPIKPKSILVVSAHWETDVPSVSAGEHP
DVIYDFSDVPDCMFQMKYPAPGSPKLAKRVQELLIAGGFKTASLDESRGFDHSSWVPLSLMYPEADIPVC
QLSVQPHLSASHHFDVGRALAPLKEEGVLFIGSGGAVHPSDDTPHWFDGVAPWAAEFDQWLEDALINGR

YDDVNNYQTKAPSGWKIAHPIPEHFLPLHVAMGAAGEKSKAELIYRTWDHGTLGYASYKFTSI

>HU08G01572.1-R

ATGCTTCAGCAACTAATTAGTGGATGGCAGAGATTGGGCATTGGCTGTCCTTATCTTGATTATAAGTATCTCG
TGACAGGCAGAGAAGTGGGTACACTTCTAATAGCTTCTAAGATCAAAACAAGAGAAAAACTAAATTTCAT
TTGCACCAGCAAAATGGGTGAGCAGGAAGCAATTAGGGAGACATTCTACATATCTCATGGGACACCAAAG
ATGTCGATCGATGATTCTATAGCAGCAAAGAAGTTCTTTCAGGAATGGGAGAAAGTCTACTCCAAGAGGCC
AAAGTCTATGCTTGTGATCTCTGCTCACTGGGAGACTGATGTGCCTGCTGTTAATGCTGTTACTCACTCTGA
TCTCATCTATGATTTCAGGGGCTTTCCTGCCATCATGTACCAGCTCAAGTACCCAGCTCCTGGTGCACCAGA
TTTGGCAAGGAGGGTAGAGGAACTTCTGACAGCATCAGGGTTCAGCTGTGTTGTAGACAAGAAGCGCGGT
CTTGATCATGGCTCATGGGTGCCTCTCATGCTCATGTACCCGGAGGCTGATATCCCGGTTTGCCAGCTCTCA
GTGCAATCACACCTAGATGGAACGCACCACTATAACTTGGGAAAGGCTTTGGCTCCTCTTAAGGAGGAAG
GTGTCCTTATCATTGGTTCTGGGAGTGCTGTTCACCCTTCAAATAGTACTCCTATTTCTCTTGATGGAGTTGC
TCCTTGGGCTGCAGCGTTCGATTCCTGGCTAGAAGAAGCTCTCAAAAGTGGAAGGTATGAAGATGTGAAC
AATTATCAAGCAAAGGCACCAAACTGGGAACTGGCaCACCCATGGCCAGAGCACTTTTACCCATTACATGT
AGCCATGGGTGCAGCTGGTGAAAACTCGAAGGCCGAGCTTATTCATCGCAGtTGGGACCATGGCACCCTTG
GCTATGCCTCCTACATATTCACTTCCTGCTGA

MLQQLISGWQRLGIGCPYLDYKYLVTGREVGTLLIASKIKTREKLNFICTSKMGEQEAIRETFYISHGTPK
MSIDDSIAAKKFFQEWEKVYSKRPKSMLVISAHWETDVPAVNAVTHSDLIYDFRGFPAIMYQLKYPAPGA
PDLARRVEELLTASGFSCVVDKKRGLDHGSWVPLMLMYPEADIPVCQLSVQSHLDGTHHYNLGKALAPL
KEEGVLIIGSGSAVHPSNSTPISLDGVAPWAAAFDSWLEEALKSGRYEDVNNYQAKAPNWELAHPWPEHF
YPLHVAMGAAGENSKAELIHRSWDHGTLGYASYIFTSC

> HU08G01572.1-W

ATGCTTCAGCAACTAATTAGTGGATGGCAGAGATTGGGCATTGGCTGTCCTTATCTTGATTATAAATATCTCG
TGACAGGCAGAGAAGTGGGTACACTTGTAATAGCTTCTAAGATCAAAACAAGAGAAAAACTAAATTTCAT
TTGCACCAGCAAAATGGGTGAGCAGGAAGCAATTAGGGAGACATTCTACATATCTCATGGGACACCAAAG
ATGTCGATCGATGATTCTATAGCAGCAAAGAAGTTCTTTCAGGAATGGGAGAAAGTCTACTCCAAGAGGCC
AAAGTCTATGCTTGTGATCTCTGCTCACTGGGAGACTGATGTGCCTGCTGTTAATGCTGTTACTCACTCTGA
TCTCATCTATGATTTCAGGGGCTTTCCTGCCATCATGTACCAGCTCAAGTACCCAGCTCCTGGTGCACCGGA
TTTGGCAAGGAGGGTAGAGGAACTTCTGACAGCATCAGGGTTCAGCTGTGTTGTAGACAAGAAGCGCGGT
CTTGATCATGGCTCATGGGTGCCTCTCATGCTCATGTACCCGGAGGCTGATATCCCGGTTTGCCAGCTCTCA
GTGCAATCACACCTAGATGGAACGCACCACTATAACTTGGGAAAGGCTTTGGCTCCTCTTAAGGAGGAAG
GTGTCCTTATCATTGGTTCTGGGAGTGCTGTTCACCCTTCAAATAGTACTCCTATTTCTCTTGATGGAGTTGC
TCCTTGGGCTGCAGCGTTCGATTCCTGGCTAGAAGAAGCTCTCAAAAGTGGAAGGTATGAAGATGTGAAC
AATTATCAAGCAAAGGCACCAAACTGGGAACTGGCACACCCATGGCCAGAGCACTTTTACCCATTACATGT
AGCCATGGGTGCAGCTGGTGAAAACTCGAAGGCCGAGCTCATTCATCGCAGTTGGGACCATGGCACCCTT
GGCTATGCCTCCTACATATTCACTTCCTGCTGA

MLQQLISGWQRLGIGCPYLDYKYLVTGREVGTLVIASKIKTREKLNFICTSKMGEQEAIRETFYISHGTPK
MSIDDSIAAKKFFQEWEKVYSKRPKSMLVISAHWETDVPAVNAVTHSDLIYDFRGFPAIMYQLKYPAPGA
PDLARRVEELLTASGFSCVVDKKRGLDHGSWVPLMLMYPEADIPVCQLSVQSHLDGTHHYNLGKALAPL
KEEGVLIIGSGSAVHPSNSTPISLDGVAPWAAAFDSWLEEALKSGRYEDVNNYQAKAPNWELAHPWPEHF
YPLHVAMGAAGENSKAELIHRSWDHGTLGYASYIFTSC

DODAβ

> HU08G01571.1-R

ATGTCATCAGCATCGCATAACTTGCCTTATGATTTTAAATACGCATACCCAATGATGTCGAGAAAAAAA
CCCAAGTTGCGAAATTCAAAATTATTCTCAAAACCCATTTCCCCTCTGTCTCTGGGCATCATCTTCATC
CTCTCCTTCTTCGGTCTGGCTTTCGCCTTCAAATTCAATTGTTGTTTTAGCAACGGTTTTCTGCAGTCA
AAGAAGGCGAACAACAGTTTTCCGCAATCAAATAAGGCCCCTGTTGTCGGTTTTCGGAAAACCCAGA
TCAAATCTAGGGCAACAAATTCAACGAGTATCGCAACAAATACGATGGCTGGTGCAACGAAGGTGAA
GGAGACGCTGTACATCTCTCATGGGTCGCCGATGCTTTCTGTTGATGATTCGTTGCCCGCGAGGCATTT
TTTGAAGAGTTGGAAGGAGAAGATCTACTCAATGAAACCCAATTCGATTCTTGTTATTTCTGGGCATTG
GGAAACCGATGAACCCTCGATTACTGCCTCTGATCGTTGCGATACTATCCATGATTTCTATGGGTTTCCT
GATCGATTGTATAAGCTCAAGTACCCAGCTCCAGGGGCTCCAGATTTGGCAAAGCGAGCACAAGAAC
TTCTCCTGAACTCAGGATTCAAGCATGTCAACTTGGACAAAAAGCGTGGGCTTGATCATGGTGCATGG
GTGCCTCTCATGCTTATGTATCCCGAGGCCGATATCCCTGTCTGTCAGCTCTCCGTTCAACTGCACAAG
GATGCCAAATATCACTATAACATCGGACAAGCATTGGCCCCCCTTAAGGAGGATGGTGTTCTCATCATG
GGTTCTGGAAGCGCAACCCACAATTTGAGGGACCTTGACTTTGATGCTGAAGGGGCTACCCCGTGGG
CTGCAGAGTTTGATAAATGGCTAGAAGAATCTCTGACAAATGGAAGGTATGAAGATGTGAATAACTAT
GAGAAGAAAGCTCCACATGCGAAAAGAGCTCACCCATGGCCAGATCACTTCTACCCATTGCATGTAG
CCATGGGTGCGGCTGGGGAGACCTCAAAGGCGGAGCTCATTCATCGCAGCTGGGGCCTTGGTACTAT
ATCTTATGCATCCTATAAATTCACCTCCACTGATTAA

MSSASHNLPYDFKYAYPMMSRKKPKLRNSKLFSKPISPLSLGIIFILSFFGLAFAFKFNCCFSNGFLQSKKA
NNSFPQSNKAPVVGFRKTQIKSRATNSTSIATNTMAGATKVKETLYISHGSPMLSVDDSLPARHFLKSWKE
KIYSMKPNSILVISGHWETDEPSITASDRCDTIHDFYGFPDRLYKLKYPAPGAPDLAKRAQELLLNSGFKH
VNLDKKRGLDHGAWVPLMLMYPEADIPVCQLSVQLHKDAKYHYNIGQALAPLKEDGVLIMGSGSATHN
LRDLDFDAEGATPWAAEFDKWLEESLTNGRYEDVNNYEKKAPHAKRAHPWPDHFYPLHVAMGAAGET
SKAELIHRSWGLGTISYASYKFTSTD

>HU08G01571.1-W

ATGTCATCAGCATCGCATAACTTGCCTTATGATTTTAAATACACATACCCAATGATGTCGAGAAAAAAA
CCCAAGTTGCGAAATTCAAAATTATTCTCAAAACCCATTTCCCCTCTGTCTCTGGGCATCATCTTCATC
CTCTCCTTCTTCGGTCTGGCTTTCGCCTTCAAATTCAACTGTTGTTTTAGAAACGGTTTTCTGCAGTCA

AAGAAGGCGAACAACAGTTTTCCGCAATCAAATAAGGCCCCTGTTGTCGGTTTTCGGAAAACCCAGA
TCAAATCTAGGGCAACAAATTCAACGAGTATCGCAACAAATACGATGGCTGGTGCAACGAAGGTGAA
GGAGACGCTGTACATCTCTCATGGGTCGCCGATGCTTTCTGTTGATGATTCGTTGCCCGCGAGGCATTT
TTTGAAGAGTTGGAAGGAGAAGATCTACTCAATGAAACCCAATTCGATTCTTGTTATTTCTGGGCATTG
GGAAACCGATGAACCCTCGATTACTGCCTCTGATCGTTGCGATACTATCCATGATTTCTATGGGTTTCCT
GATCGATTGTATAAGCTCAAGTACCCAGCTCCAGGGGCTCCGGATTTGGCAAAGCGAGCACAAGAAC
TTCTCCTGAACTCAGGATTCAAGCATGTCAACTTGGACAAAAAGCGTGGGCTTGATCATGGTGCATGG
GTGCCTCTCATGCTTATGTATCCCGAGGCCGATATCCCTGTCTGTCAGCTCTCCGTTCAACTGCACAAG
GATGCCAAATATCACTATAACATCGGACAAGCATTGGCCCCCCTTAAGGAGGATGGTGTTCTCATCATG
GGTTCTGGAAGCGCAACCCACAATTTGAGGGACCTTGACTTTGATGCTGAAGGGGTTACCCCGTGGG
CTGCAGAGTTTGATAAATGGCTAGAAGAATCTCTGACAAATGGAAGGTATGAAGATGTGAATAACTAT
GAGAAGAAAGCTCCACATGCGAAAAGAGCTCACCCATGGCCAGATCACTTCTACCCATTGCATGTAG
CCATGGGTGCAGCTGGGGAGACCTCAAAGGCGGAGCTCATTCATCGCAGCTGGGGCCTTGGTACTAT
ATCTTATGCATCCTATAAATTCACCTCCACTGATTAA

MSSASHNLPYDFKYTYPMMSRKKPKLRNSKLFSKPISPLSLGIIFILSFFGLAFAFKFNCCFRNGFLQSKKA
NNSFPQSNKAPVVGFRKTQIKSRATNSTSIATNTMAGATKVKETLYISHGSPMLSVDDSLPARHFLKSWKE
KIYSMKPNSILVISGHWETDEPSITASDRCDTIHDFYGFPDRLYKLKYPAPGAPDLAKRAQELLLNSGFKH
VNLDKKRGLDHGAWVPLMLMYPEADIPVCQLSVQLHKDAKYHYNIGQALAPLKEDGVLIMGSGSATHN
LRDLDFDAEGVTPWAAEFDKWLEESLTNGRYEDVNNYEKKAPHAKRAHPWPDHFYPLHVAMGAAGET
SKAELIHRSWGLGTISYASYKFTSTD

B5GT

> HU01G00156.1-R

ATGAACATGGCTGCTGAAGATCAACAGCTTCATGTAGTGTTCTTCCCCTTCTTGGCCCATGGCCATATG
ATTCCGACCCTCGATGTTGTAAGGCTCTTTGCATGTCATGGGGTCAGGGTCACCATCATCACCACCCCT
CTGAACGCCCACATCTTCAATAAGGCTGTTGAGACGGCTACGAAAATGGGTGCCCGGGTCGTTGATGT
TGAGCTATTCAGGTTCCCTGCCGAAGAAGCAGGCCTGCCAGAAGGATTTGAGAACTTAGAGCTAGTT
AAGGAGGATGACATGATTGTTAAGTTCATCGCTGCCAGTGAGCTGCTCAGAGAGCAGCTTGAGCCATA
CTTGGAGAAGACACGACCGAACTGCCTCGTTGCTGACATGTTTTTCCATGGGCAACGGAGTGTGCA
GCCAAGTTCGACATCCCTAGGCTAGTCTTCCATGGCACTAACTTCTTTTACTTGTGTGCTTCGGAGATT
ATCAGGCTATATGAGCCTTATAAGAATGTCTCTTCGGATGAGGAGCCATTCGCTCTTCCGTCCCTTCCA
CATGAGATACAGATGACAAGACTGCAGCTACCAGAGGATCTATGGAAGTACGAGGAATCCGATTTCAA
AAGGAGGATAGCACTAATCCACGAATCGGAGGTTGCAAGCTATGGGGTGATCGTCAACAGCTTTTATG
AGTTGGAACCGGATTATGCAGATTTCTTCGGGAAGGAATTAGGAAGGAGGGCATGGCATATAGGCCCA
GCTTTACTCTGCAATAGGAGTACCAAGAACAAAGCTCAGAGGTTGAAACAACCCGATCTAGATGAAC
ACGAGTGCCTGAAGTGGCTTAACTCAAGGAAGCCCAACTCAGTCATTTACATCTGTTTCGGAAGCACT

GCACATGTGATTGGCCCTCAGCTACATGAGATAGCAGTGGCTTTGGAAGCGTCAGAACAAGCGTTCAT
TTGGGTAGTGAAGAATGAGGATTACGAAAAGAGTGCAGAGTGGTTGCCCACAGTGTTTGAGGAGCG
GTTGGAACAACGAGGTCTAATTATAAAAGGGTGGGCTCCTCAGGTGCTTATTTTAGAGCATGAAGCTA
TAGGAGCATTTGTGACCCACTGTGGATGGAACTCGATCTTAGAAGGGATCTCGGCTGGGGTGCCAGTG
GTTACATGGCCTGTTTTTGCTGAGCAGTTCTATAACGAAAGGTTCGTGACTCAGATTTTGAAGATTGG
GGTGCCCATTGGATCTAAGAAGTGGGGTATTGTGACTACTACAGAGTACATGGTGAAACGGGACTCTA
TAGAGAAGGCATTGAGAGAAATCATGGTAGGAGAGGAAGCTGAGGAGAGGAGAAACAGAGCACAG
AAGTTGAAGGCGATGGCATGGAAGGCTGTCGAAGAAGGGGGTTCATCTTATTCTGATCTAAGTGCACT
AATAAATGAGCTTAGGGTTTATCGCACTTAA

MNMAAEDQQLHVVFFPFLAHGHMIPTLDVVRLFACHGVRVTIITTPLNAHIFNKAVETATKMGARVVDV
ELFRFPAEEAGLPEGFENLELVKEDDMIVKFIAASELLREQLEPYLEKTRPNCLVADMFFPWATECAAKFDI
PRLVFHGTNFFYLCASEIIRLYEPYKNVSSDEEPFALPSLPHEIQMTRLQLPEDLWKYEESDFKRRIALIHES
EVASYGVIVNSFYELEPDYADFFGKELGRRAWHIGPALLCNRSTKNKAQRLKQPDLDEHECLKWLNSRK
PNSVIYICFGSTAHVIGPQLHEIAVALEASEQAFIWVVKNEDYEKSAEWLPTVFEERLEQRGLIIKGWAPQV
LILEHEAIGAFVTHCGWNSILEGISAGVPVVTWPVFAEQFYNERFVTQILKIGVPIGSKKWGIVTTTEYMV
KRDSIEKALREIMVGEEAEERRNRAQKLKAMAWKAVEEGGSSYSDLSALINELRVYRT

>HU01G00156.1-W

ATGAACATGGCTGCTGAAGATCAACAGCTTCATGTAGTGTTCTTCCCCTTCTTGGCCCATGGCCATATG
ATTCCGACCCTCGATGTTGTAAGGCTCTTTGCATGTCATGGGGTCAGGGTCACCATCATCACCACCCCT
CTGAACGCCCACATCTTCAATAAGGCTGTTGAGACGGCTACGAAAATGGGTGCCCGGGTCGTTGATGT
TGAGCTATTCAGGTTCCCTGCCGAAGAAGCAGGCCTGCCAGAAGGATTTGAGAACTTAGAGCTAGTT
AAGGAGGATGACATGATTGTTAAGTTCATCGCTGCCAGTGAGCTGCTCAGAGAGCAGCTTGAGCCATA
CTTGGAGAAGACACGACCGAACTGCCTCGTTGCTGACATGTTTTTCCATGGGCAACGGAGTGTGCA
GCCAAGTTCGACATCCCTAGGCTAGTCTTCCATGGCACTAACTTCTTTTACTTGTGTGCTTCGGAGATT
ATCAGGCTATATGAGCCTTATAAGAATGTCTCTTCGGATGAGGAGCCATTCGCTCTTCCGTCCCTTCCA
CATGAGATACAGATGACAAGACTGCAGCTACCAGAGGATCTATGGAAGTACGAGGAATCCGATTTCAA
AAGGAGGATAGCACTAATCCACGAATCGGAGGTTGCAAGCTATGGGGTGATCGTCAACAGCTTTTATG
AGTTGGAACCGGATTATGCAGATTTCTTCGGGAAGGAATTAGGAAGGAGGGCATGGCATATAGGCCCA
GCTTTACTCTGCAATAGGAGTACCAAGAACAAAGCTCAGAGGTTGAAACAACCCGATCTAGATGAAC
ACGAGTGCCTGAAGTGGCTTAACTCAAGGAAGCCCAACTCAGTCATTTACATCTGTTTCGGAAGCACT
GCACATGTGATTGGCCCTCAGCTACATGAGATAGCAGTGGCTTTGGAAGCGTCAGAACAAGCGTTCAT
TTGGGTAGTGAAGAATGAGGATTACGAAAAGAGTGCAGAGTGGTTGCCCACAGTGTTTGAGGAGCG
GTTGGAACAACGAGGTCTAATTATAAAAGGGTGGGCTCCTCAGGTGCTTATTTTAGAGCATGAAGCTA
TAGGAGCATTTGTGACCCACTGTGGATGGAACTCGATCTTAGAAGGGATCTCGGCTGGGGTGCCAGTG
GTTACATGGCCTGTTTTTGCTGAGCAGTTCTATAACGAAAGGTTCGTGACTCAGATTTTGAAGATTGG
GGTGCCCATTGGATCTAAGAAGTGGGGTATTGTGACTACTACAGAGTACATGGTGAAACGGGACTCTA
TAGAGAAGGCATTGAGAGAAATCATGGTAGGAGAGGAAGCTGAGGAGAGGAGAAACAGAGCACAG

AAGTTGAAGGCGATGGCATGGAAGGCTGTCGAAGAAGGGGGTTCATCTTATTCTGATCTAAGTGCACT
AATAAATGAGCTTAGGGTTTATCGCACTTAA

MNMAAEDQQLHVVFFPFLAHGHMIPTLDVVRLFACHGVRVTIITTPLNAHIFNKAVETATKMGARVVDV
ELFRFPAEEAGLPEGFENLELVKEDDMIVKFIAASELLREQLEPYLEKTRPNCLVADMFFPWATECAAKFDI
PRLVFHGTNFFYLCASEIIRLYEPYKNVSSDEEPFALPSLPHEIQMTRLQLPEDLWKYEESDFKRRIALIHES
EVASYGVIVNSFYELEPDYADFFGKELGRRAWHIGPALLCNRSTKNKAQRLKQPDLDEHECLKWLNSRK
PNSVIYICFGSTAHVIGPQLHEIAVALEASEQAFIWVVKNEDYEKSAEWLPTVFEERLEQRGLIIKGWAPQV
LILEHEAIGAFVTHCGWNSILEGISAGVPVVTWPVFAEQFYNERFVTQILKIGVPIGSKKWGIVTTTEYMV
KRDSIEKALREIMVGEEAEERRNRAQKLKAMAWKAVEEGGSSYSDLSALINELRVYRT

>HU01G00157.1-R

ATGGGTGCTGAACCTCAACAACTCCATGCAGTGTTCTTCCCCTTCATGGCCCATGGCCACATGATCCC
AACCCTGGACATCGCGAGGCTCTTTGCATCTCGCGGTGTCAGATCAACCATCATCACCACCCCTCTCA
ACGCGCCCATCTTCACCAAGGCCATCGACAAGGCCAAGAGGACGGGAACCCCTGTGATTGACATTGA
GGTTTTCAGGTTCCCAGCCAAAGAAGCCGGCCTACCTGAAGGAAGTGAAAACCTTGATGAAGCATTG
GGTCATGAGTTTCTAAGGAAATTCATAAATGCTTCGGAGTTGCTGCGTGAGCAGCTCGAGCAGTATTT
GGATAAGACGCGGCCCAATTGCCTTGTGGCCGATGTGTTCTTCCCATGGGCAACTGAGTCTGCATCCA
AGTTCAACATCCCTAGGCTAGTGTTCCATGGGACTAGCTTCTTTGCGCTTTGTGCTGGAGAAATTATTA
ACCGGTATGAGCCTTATAAGAATGTATCCTCAGATGAGGAGCTGTTCACTCTTCCTTCGCTTCCCCATG
AGATACAGCTGACAAGGCTGCAACTTCCGGAGGATCTATGGAAGTACGAGGAAAGTGATTGGATGAA
GAGATCAAGACTAATCAAGGAATCAGAAGTTAAGAGCTATGGAGTCCTTGTGAACAGCTTTTATGAGT
TGGAACCAGATTATGCAGATTTCTACAGGAATGAATTGGGTAGGAAAGCATGGCATATTGGCCCTGTA
GTACTCTGTAATCGAAGCATCGAGGACAAGGCTCAGCGAGGAAAACCACCAGCTGTAGATGAGCACG
AATGCCGAAAATGGCTTGACTCGAGGAAGCCCAACTCAGTCATTTACGTCTGTTTTGGAAGCACCGC
GCATGTCATAGGCCCTCAGCTGCATGAGATTGCAGTGGCATTGGAATCCTCAGAACTAGCATTCATATG
GGTTGTGAAGAACGAAGACTATGAAAAGAGTGCAGAGTGGCTGCCCCCAGGACTCGAAGAGCAGGT
CCAACGAAGAGGTCTGATAATAAAAGGGTGGGCTCCTCAGGTGCTTATTTTGGAGCATGAAGCCATAG
GAGCATTCGTGACTCACTGTGGGTGGAACTCGACACTAGAAGGGATCTCAGCTGGTGTCCCAATGGT
TACATGGCCTGTTTTTGCAGAGCAATTCTACAACGAAAAGCTGGTGACTCAGATTTTGAAGATTGGAG
TGCCTGTTGGATCTAAGAAGTGGAGTATTGTGGCTTCGACAGAGTACATGGTGAAGCAGGATGCCATC
GAGAAGGCATTAAGAGAAGTTATGGAGGGCGAGGAAGCTGAAGAGAGGAGAAACAGGGCAAACAA
GTTGAAGGAAATGGCCTGGAAGGCTGTTGAAGAAGGTGGTTCATCATATTCAGACTTAACAGCATTGA
TCCATGAGTTGAGGGTTTATCGCACTTAA

MGAEPQQLHAVFFPFMAHGHMIPTLDIARLFASRGVRSTIITTPLNAPIFTKAIDKAKRTGTPVIDIEVFRFP
AKEAGLPEGSENLDEALGHEFLRKFINASELLREQLEQYLDKTRPNCLVADVFFPWATESASKFNIPRLVF
HGTSFFALCAGEIINRYEPYKNVSSDEELFTLPSLPHEIQLTRLQLPEDLWKYEESDWMKRSRLIKESEVKS
YGVLVNSFYELEPDYADFYRNELGRKAWHIGPVVLCNRSIEDKAQRGKPPAVDEHECRKWLDSRKPNSVI

YVCFGSTAHVIGPQLHEIAVALESSELAFIWVVKNEDYEKSAEWLPPGLEEQVQRRGLIIKGWAPQVLILE
HEAIGAFVTHCGWNSTLEGISAGVPMVTWPVFAEQFYNEKLVTQILKIGVPVGSKKWSIVASTEYMVKQ
DAIEKALREVMEGEEAEERRNRANKLKEMAWKAVEEGGSSYSDLTALIHELRVYRT

>HU01G00157.1-W

ATGGGTGCTGAACCTCAACAACTCCATGCAGTGTTCTTCCCCTTCATGGCCCATGGCCACATGATCCC
AACCCTGGACATCGCGAGGCTCTTTGCATCTCGCGGTGTCAGATCAACCATCATCACCACCCCTCTCA
ACGCGCCCATCTTCACCAAGGCCATCGACAAGGCCAAGAGGACGGGAACCCCTGTGATTGACATTGA
GGTTTTCAGGTTCCCAGCCAAAGAAGCCGGCCTACCTGAAGGAAGTGAAAACCTTGATGAAGCATTG
GGTCATGAGTTTCTAAGGAAATTCATAAATGCTTCGGAGTTGCTGCGTGAGCAGCTCGAGCAGTATTT
GGATAAGACGCGGCCCAATTGCCTTGTGGCCGATGTGTTCTTCCCATGGGCAACTGAGTCTGCATCCA
AGTTCAACATCCCTAGGCTAGTGTTCCATGGGACTAGCTTCTTTGCGCTTTGTGCTGGAGAAATTATTA
ACCGGTATGAGCCTTATAAGAATGTATCCTCAGATGAGGAGCTGTTCACTCTTCCTTCGCTTCCCCATG
AGATACAGCTGACAAGGCTGCAACTTCCGGAGGATCTATGGAAGTACGAGGAAAGTGATTGGATGAA
GAGATCAAGACTAATCAAGGAATCAGAAGTTAAGAGCTATGGAGTCCTTGTGAACAGCTTTTATGAGT
TGGAACCAGATTATGCAGATTTCTACAGGAATGAATTGGGTAGGAAAGCATGGCATATTGGCCCTGTA
GTACTCTGTAATCGAAGCATCGAGGACAAGGCTCAGCGAGGAAAACCACCAGCTGTAGATGAGCACG
AATGCCGAAAATGGCTTGACTCGAGGAAGCCCAACTCAGTCATTTACGTCTGTTTTGGAAGCACCGC
GCATGTCATAGGCCCTCAGCTGCATGAGATTGCAGTGGCGTTGGAATCCTCAGAACTAGCATTCATATG
GGTTGTGAAGAACGAAGACTATGAAAAGAGTGCAGAGTGGCTGCCCCCAGGACTCGAAGAGCAGGT
CCAACGAAGAGGTCTGATAATAAAAGGGTGGGCTCCTCAGGTGCTTATTTTGGAGCATGAAGCCATAG
GAGCATTCGTGACTCACTGTGGGTGGAACTCGACACTAGAAGGGATCTCAGCTGGTGTCCCAATGGT
TACATGGCCTGTTTTTGCAGAGCAATTCTACAACGAAAAGCTGGTGACTCAGATTTTGAAGATTGGAG
TGCCTGTTGGATCTAAGAAGTGGAGTATTGTGGCTTCGACAGAGTACATGGTGAAGCAGGATGCCATC
GAGAAGGCATTAAGAGAAGTTATGGAGGGCGAGGAAGCTGAAGAGAGGAGAAACAGGGCAAACAA
GTTGAAGGAAATGGCCTGGAAGGCTGTTGAAGAAGGTGGTTCATCATATTCAGACTTAACAGCATTGA
TCCATGAGTTGAGGGTTTATCGCACTTAA

MGAEPQQLHAVFFPFMAHGHMIPTLDIARLFASRGVRSTIITTPLNAPIFTKAIDKAKRTGTPVIDIEVFRFP
AKEAGLPEGSENLDEALGHEFLRKFINASELLREQLEQYLDKTRPNCLVADVFFPWATESASKFNIPRLVF
HGTSFFALCAGEIINRYEPYKNVSSDEELFTLPSLPHEIQLTRLQLPEDLWKYEESDWMKRSRLIKESEVKS
YGVLVNSFYELEPDYADFYRNELGRKAWHIGPVVLCNRSIEDKAQRGKPPAVDEHECRKWLDSRKPNSVI
YVCFGSTAHVIGPQLHEIAVALESSELAFIWVVKNEDYEKSAEWLPPGLEEQVQRRGLIIKGWAPQVLILE
HEAIGAFVTHCGWNSTLEGISAGVPMVTWPVFAEQFYNEKLVTQILKIGVPVGSKKWSIVASTEYMVKQ
DAIEKALREVMEGEEAEERRNRANKLKEMAWKAVEEGGSSYSDLTALIHELRVYRT

>>HU03G00258.1-R

ATGGGTGCTGAGCCTCAACAACTCCATGTAGCTTTCTTCCCTTTCATGGCTCATGGCCACATGATCCCC
ATCCTCGACATAGCGAGGCTCTTTGTGGCTCGTGGGGTCAAGGCAACCATCATAACCACCCCGCTTAA

TGTGCCTGTCTTCACAAGGGCCATTGACAAAGGAAGAAAGAAACCAGGGAGCACAGGGATCAACAT
TGAGGTTTTCACACTGCCAGCACGAGAAGCTGGCTTACCAGAAGGGTGTGAGAATCTGGATCAAGCA
ATAGCCATGGGTATAGCCCCGGTGTTATTTAAGGCTTCGGAGATGCTCCAGGACCAGCTAGAGCGGTT
CTTGGAGCAGACCCGCCCCAACTGCATTGTGGCCGATATGTTCTTCGCATGGGTAACTGACTCCGCAG
ACAAGTTCAATGTCCCCCGGCTAGTTTTCCATGGGATCAGCTTTTTTTCCCTTTGTGCAGAAGAGATGG
TCGTGCTACATGAGCCTCACAAGAATGTTTCTTCTGATGAGGAGCCCTTGGTTCTTCCTTACCTCCCAC
ACGAGGTGAAGTTGACAAGGTTGCAGCTCCCAGATTGTGTCATGAACCAGGAGAGCGAATTTGGGAA
CAGATTAAAAAAGATCAGGGAATCTGAGCTCAAGAGTTATGGGGTGCTTGTTAACAGTTTCTATGAGT
TAGAACCAGACTATGCAGAGCTCTACAGGAAAGAGTTGGGAAGGAGAGCATGGCACATAGGCCCTGT
TTCACTCTGCAATAGGAGCATTGAGGATAAGGCTTGGAGGGGGAAGCAAGCTTCCTTAGACAAACAC
GAGTGGTTGAATTGGCTCAACTTGAAGAAACCCAACTCCGTCATTTATATCAGTTTCGGGAGTATGGC
AAACGTGATAGCTCCTCAGTTGCACGAGATTGCTCTGGCTCTAGAAGCTGCTGGCCAAGACTTCATCT
GGGTCGTGAGGAACAGCGATGGGCAGGAAGAAGAGTGGTTGCCACAAGGGTTCGAACAGAGGATG
GAAGGAAAAGGTCTGATAATAAGAGGGTGGGCCCCACAGGTGTTGATTTTGGAGCATGAGGCTGTGG
GAGCCTTTGTGACTCACTGTGGGTGGAACTCGACAATTGAGAGTATAAGCAGTGGGAAGCCCATGGT
TACATGGCCTTTGTTCGCCGAGCAATTCTATAATGAGAAATTAGTGACTCACATTTTGAAAACTGGGGT
CTCCGTTGGAGTTAAGAAGTGGAGCAAAGAGCCTTCCCTAGAGAATGTGGTGAAGCGCGATGCCATA
GAGAAGGCACTGAGAGAGATAATGGCAGGAGAGGAGGCAGAAGAGAGGAGAAGCAGAGCCAAGA
AGTTGAAGGAGATGGCATGGAAGGCAGTTGAAGAAGGTGGCTCATCATACTGTGATTTAACTGCTTTG
ATAAATGGATTGCAGAATTATCGTGCTTAA

MGAEPQQLHVAFFPFMAHGHMIPILDIARLFVARGVKATIITTPLNVPVFTRAIDKGRKKPGSTGINIEVFT
LPAREAGLPEGCENLDQAIAMGIAPVLFKASEMLQDQLERFLEQTRPNCIVADMFFAWVTDSADKFNVPR
LVFHGISFFSLCAEEMVVLHEPHKNVSSDEEPLVLPYLPHEVKLTRLQLPDCVMNQESEFGNRLKKIRESE
LKSYGVLVNSFYELEPDYAELYRKELGRRAWHIGPVSLCNRSIEDKAWRGKQASLDKHEWLNWLNLKKP
NSVIYISFGSMANVIAPQLHEIALALEAAGQDFIWVVRNSDGQEEEWLPQGFEQRMEGKGLIIRGWAPQV
LILEHEAVGAFVTHCGWNSTIESISSGKPMVTWPLFAEQFYNEKLVTHILKTGVSVGVKKWSKEPSLENV
VKRDAIEKALREIMAGEEAEERRSRAKKLKEMAWKAVEEGGSSYCDLTALINGLQNYRA

>HU03G00258.1-W

ATGGGTGCTGAGCCTCAACAACTCCATGTAGCTTTCTTCCCTTTCATGGCTCATGGCCACATGATCCCC
ATCCTCGACATAGCGAGGCTCTTTGTGGCTCGTGGGGTCAAGGCAACCATCATAACCACCCCGCTTAA
TGTGCCTGTCTTCACAAGGGCCATTGACAAAGGAAGAAAGAAACCAGGGAGCACAGGGATCAACAT
TGAGGTTTTCACACTGCCAGCACGAGAAGCTGGCTTACCAGAAGGGTGTGAGAATCTGGATCAAGCA
ATAGCCATGGGTATAGCCCCGGTGTTATTTAAGGCTTCGGAGATGCTCCAGGACCAGCTAGAGCGGTT
CTTGGAGCAGACCCGCCCCAACTGCATTGTGGCCGATATGTTCTTCGCATGGGTAACTGACTCCGCAG
ACAAGTTCAATGTCCCCCGGCTAGTTTTCCATGGGATCAGCTTTTTTTCCCTTTGTGCAGAAGAGATGG
TCGTGCTACATGAGCCTCACAAGAATGTTTCTTCTGATGAGGAGCCCTTGGTTCTTCCTTACCTCCCAC
ACGAGGTGAAGTTGACAAGGTTGCAGCTCCCAGATTGTGTCATGAACCAGGAGAGCGAATTTGGGAA

CAGATTTAAAAAGATCAGGGAATCTGAGCTCAAGAGTTATGGGGTGCTTGTTAACAGTTTCTATGAGT
TAGAACCAGACTATGCAGAGCTCTACAGGAAAGAGTTGGGAAGGAGAGCATGGCACATAGGCCCTGT
TTCACTCTGCAATAGGAGCATTGAGGATAAGGCTTGGAGGGGGAAGCAAGCTTCCTTAGACAAACAC
GAGTGGTTGAATTGGCTCAACTTGAAGAAACCCAACTCCGTCATTTATATCAGTTTCGGGAGTATGGC
AAACGTGATAGCTCCTCAGTTGCACGAGATTGCTCTGGCTCTAGAAGCTGCTGGCCAAGACTTCATCT
GGGTCGTGAGGAACAGCAATGGGCAGGAAGAAGAGTGGTTGCCACAAGGGTTCGAACAGAGGATG
GAAGGAAAAGGTCTGATAATAAGAGGGTGGGCCCCACAGGTGTTGATTTTGGAGCATGAGGCTGTGG
GAGCCTTTGTGACTCACTGTGGGTGGAACTCGACTCTTGAAGGGATCTCAGCTGGAGTGCCAATGGT
TACATGGCCTTTGTTCGCCGAGCAATTCTATAATGAGAAATTAGTGACTCACATTTTGAAAACTGGGGT
CTCCGTTGGAGTTAAGAAGTGGAGCAAAGAGCCTTCCCTAGAGAATGTGGTGAAGCGCGATGCCATA
GAGAAGGCACTGAGAGAGATAATGGCAGGAGAGGAGGCAGAAGAGAGGAGAAGCAGAGCCAAGA
AGTTGAAGGAGATGGCATGGAAGGCAGTTGAAGAAGGTGGTTCATCATACTGTGATTTAACTGCTTTG
ATAAATGGATTGCAGAATTATCGTGCTTAA

MGAEPQQLHVAFFPFMAHGHMIPILDIARLFVARGVKATIITTPLNVPVFTRAIDKGRKKPGSTGINIEVFT
LPAREAGLPEGCENLDQAIAMGIAPVLFKASEMLQDQLERFLEQTRPNCIVADMFFAWVTDSADKFNVPR
LVFHGISFFSLCAEEMVVLHEPHKNVSSDEEPLVLPYLPHEVKLTRLQLPDCVMNQESEFGNRFKKIRESE
LKSYGVLVNSFYELEPDYAELYRKELGRRAWHIGPVSLCNRSIEDKAWRGKQASLDKHEWLNWLNLKKP
NSVIYISFGSMANVIAPQLHEIALALEAAGQDFIWVVRNSNGQEEEWLPQGFEQRMEGKGLIIRGWAPQV
LILEHEAVGAFVTHCGWNSTLEGISAGVPMVTWPLFAEQFYNEKLVTHILKTGVSVGVKKWSKEPSLEN
VVKRDAIEKALREIMAGEEAEERRSRAKKLKEMAWKAVEEGGSSYCDLTALINGLQNYRA

>HU03G00266.1-R

ATGGCTTCAGAACAAACCCAGTTCCACATATTCTTCCTTCCTTACCTTGTCCCAGGCCATATGATCCCA
ACTCTGGACTTGGCCAGATTATTTGCTACTCGAGGCGTTAAATGCACCATCATCACCACTCCCCTTAAC
ATCCCTTTCTTTGCTGAAGCAACCAAAACTGACAGCCAAAAACTTGGTATTGAAATCAACCTTTTATC
AGTGAAATTCCCATATGAGGAACCAGGGTTCCCTAAAGAGTGTAAGAGTACACAAAATTTCTACAAA
GCCATTGCCGAGCTTCAGCACCCTTTTGGGGTACTAATGGAGGAGTTCCATCCCAACTGCCTTGTGGC
TGATTTTCAGTTTCCTTGGGCTGATTCTGTAGCTAAACACCTCGGAGTCCCATGTTTGTCATTCGATGG
ATGCAGTTACATTTATCCGTGTGTTTCTTCGAATCTGAATCGATACCAACCGCACAAGGCGGTTTCATC
TGATTCAGAGCATTTTGTGGTTCCAGGCCTTCCACATGAAGTAAAGCTAAGGAGGGCAGAATTGGCAC
CTTATGAAAAAGTAGAGTCTGAGAAGTGGATGATTGAGCTGGTTGATCGGATGTGGGCGGGGCAGAG
GGAGTGTTACGGGGTGATCAAGAACAGTTTCTATGAGTTAGAGCCTGATTATGCTGATTACTATGAAA
ATAACCTGGGATTCACACACTGGCACATTGGCCCGCTTTTTCTCTACTTCGAGGGCAAAGGAAGTGAC
TGTTATCAGATAGGAAAGAAATCCTCCGTTGATGTAGAGGAATGCTTAAGATGGCTTGATGAGAAACC
GGACAATTCAGTCATTTATGTCTGCTTCGGTAGCATGTCAAATGTGGCATATGCCCAGCTAGATGAGAT
TGCCAGAGCACTTGAATCTCTAGCCCAAGATTTCATCCTAGTAGTAAGGAACAAGGAGGACACCAAT
GGGTGGGTCCTGGATGGGTTCGAGGATAGGGTTAAAGGGCAAGGTCTGGCTATAAGAGGTTGGGCCC
CCCAACGGCTCATCCTCGAGCATCAAGCTGTTGGTGGGTTCGTGACTCATTGTGGGTGGAACTCGATT

CTCGAAGGAGTGGTGGCTGGCCTGCCCATGGTGACATGGCCGCGCCATGCTGAGCAGTTTTACAATG
AGAAATTGGTCACCAATGTTCTCAGAATCGGGGTTCCTGTTGGAGTGAAGGGATGGGCTGGGACTCT
AGATGAAAATGGGGATGTGATTGGGAAGGATGAAATTGTGACGGCCATAAGTAAGATAGTGGTTGGG
AAAGAAGCTGAGGATATGAGGAAGAGGGCTCAAGAACTCAAGAAAATGGCAAGGAGGGCTATTCAA
GAAGAGGGATCATCATACTCAGATTTGACTGCTCTGATTGAAGAACTGAAGGCGTGCACTATGTCACA
AAGAATTTGA

MASEQTQFHIFFLPYLVPGHMIPTLDLARLFATRGVKCTIITTPLNIPFFAEATKTDSQKLGIEINLLSVKFPY
EEPGFPKECKSTQNFYKAIAELQHPFGVLMEEFHPNCLVADFQFPWADSVAKHLGVPCLSFDGCSYIYPCV
SSNLNRYQPHKAVSSDSEHFVVPGLPHEVKLRRAELAPYEKVESEKWMIELVDRMWAGQRECYGVIKNS
FYELEPDYADYYENNLGFTHWHIGPLFLYFEGKGSDCYQIGKKSSVDVEECLRWLDEKPDNSVIYVCFGS
MSNVAYAQLDEIARALESLAQDFILVVRNKEDTNGWVLDGFEDRVKGQGLAIRGWAPQRLILEHQAVGG
FVTHCGWNSILEGVVAGLPMVTWPRHAEQFYNEKLVTNVLRIGVPVGVKGWAGTLDENGDVIGKDEIV
TAISKIVVGKEAEDMRKRAQELKKMARRAIQEEGSSYSDLTALIEELKACTMSQRI

>HU03G00266.1-W

ATGGCTTCAGAACAAACCCAGTTCCACATATTCTTCCTTCCTTACCTTGTCCCAGGCCATATGATCCCA
ACTCTGGACTTGGCCAGATTATTTGCTACTCGAGGCGTTAAATGCACCATCATCACCACTCCCCTTAAC
ATCCCTTTCTTTGCTGAAGCAACCAAAACTGACAGCCAAAAACTTGGTATTGAAATCAACCTTTTATC
AGTGAAATTCCCATATGAGGAACCAGGGTTCCCAAAAGAGTGTAAGAGTACACAAAATTTCTACAAA
GCCATTGCCGAGCTTCAGCACCCTTTTGGGGTACTAATGGAGGAGTTCCATCCCAACTGCCTTGTGGC
CGATTTTCAGTTTCCTTGGGCTGATTCTGTAGCTAAACACCTCGGAGTCCCATGTTTGTCATTCGATGT
ATGCAGTTACATTTATCCGTGTGTTTCTTCGAATCTGAATCGATACCAACCACACAAGGCGGTTTCATC
TGATTCAGAGCATTTTGTGGTTCCAGGCCTTCCACATGAAGTAAAGCTAAGGAGGGCAGAATTGGCAC
CTTATGAAAAAGTAGAGTCTGAGAAGTGGATGATTGAGCTGGTTGATCGGATGTGGGCGGGGCAGAG
GGAGTGTTACGGGGTGATCAAGAACAGTTTCTATGAGTTAGAGCCTGATTATGCTGATTACTATGAAA
ATAACCTGGGATTCACACACTGGCACATTGGCCCGCTTTTTCTCTACTTCGAGGGCAAAGGAAGTGAC
TGTTATCAGATAGGAAAGAAATCCTCCGTTGATGTAGAGGAATGCTTAAGATGGCTTGATGAGAAACC
GGACAATTCAGTCATTTATGTCTGCTTCGGTAGCATGTCAAATGTGGCATATGCCCAGCTAGATGAGAT
TGCCAGAGCACTTGAATCTCTAGCCCAAGATTTCATCCTGGTAGTAAGGAACAAGGAGGACACCAAT
GGGTGGGTCCTGGATGGGTTCGAGGATAGGGTTAAAGGGCAAGGTCTGGTTATAAGAGGTTGGGCCC
CCCAACGGCTCATCCTCGAGCATCAAGCTGTTGGTGGGTTCGTGACTCATTGTGGGTGGAACTCGATT
CTCGAAGGAGTGGTGGCTGGCCTGCCCATGGTGACATGGCCGCGCCATGCTGAGCAGTTTTACAATG
AGAAATTGGTCACCAATGTTCTCAGAATCGGGGTTCCTGTTGGAGTGAAGGGATGGGCTGGGACTCT
AGATGAAAATGGGGATGTGATTGGGAAGGATGAAATTGTGACGGCCATAAGTAAGATAGTGGTTGGG
AAAGAAGCTGAGGATATGAGGAAGAGGGCTCAAGAACTCAAGAAAATGGCAAGGAGGGCTATTCAA
GAAGAGGGATCATCATACTCAGATTTGACTGCTCTGATTGAAGAACTGAAGGCGTGCACTATGTCACA
AAGAATTTGA

MASEQTQFHIFFLPYLVPGHMIPTLDLARLFATRGVKCTIITTPLNIPFFAEATKTDSQKLGIEINLLSVKFPY
EEPGFPKECKSTQNFYKAIAELQHPFGVLMEEFHPNCLVADFQFPWADSVAKHLGVPCLSFDVCSYIYPCV
SSNLNRYQPHKAVSSDSEHFVVPGLPHEVKLRRAELAPYEKVESEKWMIELVDRMWAGQRECYGVIKNS
FYELEPDYADYYENNLGFTHWHIGPLFLYFEGKGSDCYQIGKKSSVDVEECLRWLDEKPDNSVIYVCFGS
MSNVAYAQLDEIARALESLAQDFILVVRNKEDTNGWVLDGFEDRVKGQGLVIRGWAPQRLILEHQAVGG
FVTHCGWNSILEGVVAGLPMVTWPRHAEQFYNEKLVTNVLRIGVPVGVKGWAGTLDENGDVIGKDEIV
TAISKIVVGKEAEDMRKRAQELKKMARRAIQEEGSSYSDLTALIEELKACTMSQRI

>HU03G00267.1-R

ATGGCTTCAGAAGAGGACCAACTTCACATATTTTTCCTACCCTTCATGGCTCAAGGCCATGTGATCCCA
ATGATTGACACGGCCAGATTATTCGCCACTCGTGGCGTCAAATGCACCATAGTCACCACTTCTTCGTTT
GCTGAGACCATCAACCTGGATAACCAAAATCTTGGGATTCAAATTAACCTCCAAACCATGAAATTCCC
CGCTAAGGAAGCAGGGCTCCCAGAAGGGTGTGAAAGTACAGATTCCATCTCCGGCCCTGAAATGTTC
CACAATTTCTTCAAAGGCCTTTCTCTTCTTCAACACCCATTAGAGAACTTAGTCGAAACTTTGCGACCT
GATTGTCTGGTAGCTGATGCCTTCTTCCCTTGGGCTAATTCCACTGCTGAGAAGTTCGGAATCCCTCGT
TTATGCTTCCATGGATGTGGGTACCTCTCTGAGTGCATCAGTTACAACTTAATTATGCACCAACCACTC
AAGAACATTTCATCATCAGGTTCAGACACTTTAGTTGTTCCTGGTAATCTGCCTCATGAGATCAAGCTG
ATGATATCTGAACTGTCTCCCTTCGACAAGGGGGCATGTTCAAAGGCTTTTCTTGACTTAAGGGATGA
AATTATAGAGGCAAAAAGGTCATGTCATGGGTGGATCATGAATAGCTTTTATGAGCTCGAAACATCTTA
TGTTGATTACTTTGAGAAGGAATTGGGGTTCAAACACTGGCATATCGGCCCCCTTGCGCTCTATTTTAA
GGATCAGATCAGAGGGAAGAAGTCCTCCATTGATTTGGAGGGATGCTTGAAATGGCTAGACCAAAAA
GAGGCCAATTCAGTTATTTACATTTGCTTTGGGAGCTTCTTAGATGTGCCAGATGCTCAGCTTCATGAG
ATAGCCTTAGCTCTTGAAGCTTTGGGTCAAAATTTTATCTTAGTGGTGAGGAAAGACTCTGACAATTG
GATGCCACAAGGATTTAAGGACAGGGTGAAAGAGCAAGGCTTGATAATCCGAGGGTGGGCCCCTCAA
GTGCTAATTTTGAACCACCGAGCTGTGGGAGGGTTTGTGACTCATTGTGGGTGGAACTCGGTGTTAGA
AGGAGTCACTGCGGGTGTGCCAATGGTGACATGGCCTCTTCATGCTGAGCAGTTTTACAATGAGAAA
CTGGTGACTCAAGTTCTCAGGATTGGCATTCAAGTCGGGGCCAAAAAATGGGTTGCGAGATTTGAGG
ATCAAAATATTCTCAGCAGCGAAACGATATTGATGGCTATGAGAGGGGTAATGGCTGGAGAGGAAGCT
CAGGACATGAGAAACAGGGCAGTGGAGCTCAAAGAAATGGCCAAGAGGGCTGTTTCTGAAGGAGGT
TCATCCTCCTCTGCAATAAATAACTTGATTGCAGAAATTAAATCTTACAAGATTTGA

MASEEDQLHIFFLPFMAQGHVIPMIDTARLFATRGVKCTIVTTSSFAETINLDNQNLGIQINLQTMKFPAKE
AGLPEGCESTDSISGPEMFHNFFKGLSLLQHPLENLVETLRPDCLVADAFFPWANSTAEKFGIPRLCFHGCG
YLSECISYNLIMHQPLKNISSSGSDTLVVPGNLPHEIKLMISELSPFDKGACSKAFLDLRDEIIEAKRSCHGW
IMNSFYELETSYVDYFEKELGFKHWHIGPLALYFKDQIRGKKSSIDLEGCLKWLDQKEANSVIYICFGSFL
DVPDAQLHEIALALEALGQNFILVVRKDSDNWMPQGFKDRVKEQGLIIRGWAPQVLILNHRAVGGFVTH
CGWNSVLEGVTAGVPMVTWPLHAEQFYNEKLVTQVLRIGIQVGAKKWVARFEDQNILSSETILMAMRG
VMAGEEAQDMRNRAVELKEMAKRAVSEGGSSSSAINNLIAEIKSYKI

>HU03G00267.1-W

ATGGCTTCAGAAGAGGACCAACTTCACATATTTTTCCTACCCTTCATGGCTCAAGGCCATGTGATCCCA
ATGATTGACACGGCCAGATTATTCGCCACTCGTGGCGTCAAATGCACCATAGTCACCACTTCTTCGTTT
GCTGAGACCATCAACCTGGATAACCAAAATCTTGGGATTCAAATTAACCTCCAAACCATGAAATTCCC
CGCTAAGGAAGCAGGGCTCCCAGAAGGGTGTGAAAGTACAGATTCCATCTCCGGCCCTGAAATGTTC
CACAATTTCTTCAAAGGCCTTTCTCTTCTTCAACACCCATTAGAGAACTTAGTCGAAACTTTGCGACCT
GATTGTCTGGTAGCTGATGCCTTCTTCCCTTGGGCTAATTCCACTGCTGAGAAGTTCGGAATCCCTCGT
TTATGCTTCCATGGATGTGGGTACCTCTCTGAGTGCATCAGTTACAACTTAATTATGCACCAACCACTC
AAGAACATTTCATCATCAGGTTCAGACACTTTAGTTGTTCCTGGTAATCTGCCTCATGAGATCAAGCTG
ATGATATCTGAACTGTCTCCCTTCGACAAGGGGGCATGTTCAAAGGCTTTTCTTGACTTAAGGGATGA
AATTATAGAGGCAAAAAGGTCATGTCATGGGTGGATCATGAATAGCTTTTATGAGCTCGAAACATCTTA
TGTTGATTACTTTGAGAAGGAATTGGGGTTCAAACACTGGCATATCGGCCCCCTTGCGCTCTATTTTAA
GGATCAGATCAGAGGGAAGAAGTCCTCCATTGATTTGGAGGGATGCTTGAAATGGCTAGACCAAAAA
GAGGCCAATTCAGTTATTTACATTTGCTTTGGGAGCTTCTTAGATGTGCCAGATGCTCAGCTTCATGAG
ATAGCCTTAGCTCTTGAAGCTTTGGGTCAAAATTTTATCTTAGTGGTGAGGAAAGACTCTGACAATTG
GATGCCACAAGGATTTAAGGACAGGGTGAAAGAGCAAGGCTTGATAATCCGAGGGTGGGCCCCTCAA
GTGCTAATTTTGAACCACCGAGCTGTGGGAGGGTTTGTGACTCATTGTGGGTGGAACTCGGTGTTAGA
AGGAGTCACTGCGGGTGTGCCAATGGTGACATGGCCTCTTCATGCTGAGCAGTTTTACAATGAGAAA
CTGGTGACTCAAGTTCTCAGGATTGGCATTCAAGTCGGGGCCAAAAAATGGGTTGCGAGATTTGAGG
ATCAAAATATTCTCAGCAGCGAAACGATATTGATGGCTATGAGAGGGGTAATGGCTGGAGAGGAAGCT
CAGGACATGAGAAACAGGGCAGTGGAGCTCAAAGAAATGGCCAAGAGGGCTGTTTCTGAAGGAGGT
TCATCCTCCTCTGCAATAAATAACTTGATTGCAGAAATTAAATCTTACAAGATTTGA

MASEEDQLHIFFLPFMAQGHVIPMIDTARLFATRGVKCTIVTTSSFAETINLDNQNLGIQINLQTMKFPAKE
AGLPEGCESTDSISGPEMFHNFFKGLSLLQHPLENLVETLRPDCLVADAFFPWANSTAEKFGIPRLCFHGCG
YLSECISYNLIMHQPLKNISSSGSDTLVVPGNLPHEIKLMISELSPFDKGACSKAFLDLRDEIIEAKRSCHGW
IMNSFYELETSYVDYFEKELGFKHWHIGPLALYFKDQIRGKKSSIDLEGCLKWLDQKEANSVIYICFGSFL
DVPDAQLHEIALALEALGQNFILVVRKDSDNWMPQGFKDRVKEQGLIIRGWAPQVLILNHRAVGGFVTH
CGWNSVLEGVTAGVPMVTWPLHAEQFYNEKLVTQVLRIGIQVGAKKWVARFEDQNILSSETILMAMRG
VMAGEEAQDMRNRAVELKEMAKRAVSEGGSSSSAINNLIAEIKSYKI

>HU10G00514.1-R

ATGGGTGCTGAACGTAGTCAACTACATGTAGCTTTCTTCCCTTGGATGGCCCCTGGCCACACCATCCC
AATGATCGACATGGTTCGCCTATTTGCAGCGCGCCATGTGAAAACAACCGTCATAACCACCCCTTTGA
ATGCCCCAAACTTCGCCAAGTCTCTCAACGGCACTGCTGCCACCCTTGAAATATTCACATTTCCCTCC
AACAAAGTCGGCCTCCCTCAAGGCATTGAGAACGTGGAGGATTGCACTTCTCCTGAAATGACCATGA
ATTTCATGAGAGGGACTCAACTGCTTCGAGGTCCCCTCGAGGAGTTCTTGGAGAAGACCCGGCCTGA
TTGCCTCATAGCTGATGCTTTCTTTCCCTTCGCAAGTGAATCTGCCGCTGGATTTGGTGTCACTATGCTA

GTCTTTCATGGGACTAGCTTCTTTGCCCTTTGTGCTAATGTGATTTTGTTTAAACATAAGCCTTTTATGT
CAGTTTCATCCAATGATGACTACTTTGTCATCCCAAATCTGCCTCATTTAATTAGGTTCACGCGGTTGCA
AATACCTCCTGAGATGCGTGAAAATAAGGAGAGTGACGTGTTAAGGATTGTGGAAGCAGGTTTTCTCT
CTCTCCATAGAAGTTATGGTACTATTGTCAATAGCTTCTATGAGCTTGAGCCAGAATATGCTGATTATTAT
AGGAAGGTGTTGGGGAGAAGGGCATGGCATATAGGCCCTCTTTCCCTATGTAACAAGGATTTTGAGGG
TAAGTCTCAAAGAGGGCATAAAGCTTCAATCAACGGGCACGAGTGCCTAAAATGGCTTGATTCTAAG
GAACCCAACTCAGTTATTTATGTTTGTTTTGGGAGCCGGACTCGATTTTCATCCTCTCAACTTCGTGAG
ATTGCGATGGCTTTTGAGGCTTCAGGGCAACAATTCATTTGGGTTGTAAAGAAGCCTATGAATGAAGA
GGAAGACAAGGGTAATGAACCATGGTTGCCAGAAGGGTTCGAGAAGAGGACCAGAGATCAGGGACT
CATCATAAGAGGATGGGCACCCCAAGTGTTAATTTGGAGCATGAAGCCATAGGAGGGTTCGTGACTC
ATTGTGGATGGAACTCGACATTGGAAGGCATAACGGCTGGTGTGCCCATGGTCACTTGGCCAGTGTTT
GCTGAACAGTTTTTTAACGAAAAGTTGGTGACTGAGATTTTGAAGGTTGGGATTCCCATTGGGGTTAA
AGAGTGGAGTGTGCAAGTTGTTGAAAGTAATATTATTAAGCAGGAACAAATAGAGGCAGCATTCAGG
CAACTCATGGTGGATGAAGAGGCTCTCGAGATGCGAAGCAGAGCAAAGAAACTTAAGGAGATGGCA
TGCAAGGCTATGGAAGAAGGTGGCTCGTCTCACTCTGATCTCTGTGCCTTGTTGCAAGACTTGAGTTC
TTCTAGCCATAACCATCATTCTTGA

MGAERSQLHVAFFPWMAPGHTIPMIDMVRLFAARHVKTTVITTPLNAPNFAKSLNGTAATLEIFTFPSNKV
GLPQGIENVEDCTSPEMTMNFMRGTQLLRGPLEEFLEKTRPDCLIADAFFPFASESAAGFGVTMLVFHGTS
FFALCANVILFKHKPFMSVSSNDDYFVIPNLPHLIRFTRLQIPPEMRENKESDVLRIVEAGFLSLHRSYGTIV
NSFYELEPEYADYYRKVLGRRAWHIGPLSLCNKDFEGKSQRGHKASINGHECLKWLDSKEPNSVIYVCF
GSRTRFSSSQLREIAMAFEASGQQFIWVVKKPMNEEEDKGNEPWLPEGFEKRTRDQGLIIRGWAPQVLILE
HEAIGGFVTHCGWNSTLEGITAGVPMVTWPVFAEQFFNEKLVTEILKVGIPIGVKEWSVQVVESNIIKQEQ
IEAAFRQLMVDEEALEMRSRAKKLKEMACKAMEEGGSSHSDLCALLQDLSSSSHNHHS

>HU10G00514.1-W

ATGGGTGCTGAACGTAGTCAACTACATGTAGCTTTCTTCCCTTGGATGGCCCCTGGCCACACCATCCC
AATGATCGACATGGTTCGCCTATTTGCAGCGCGCCATGTGAAAACAACCGTCATAACCACCCCTTTGA
ATGCCCCAAACTTCACCAAGTCTCTCAACGGCACTGATGCCACCCTTGAAATATTCACATTTCCCTCCA
ACGAAGTCGGCCTCCCTCAAGGCATTGAGAACGTGGAGGATTGCACTTCTCCTGAAATGACCATGAA
TTTCATGAGAGGGACTCAGCTGCTTCGAGGTCCCCTCGAGGAGTTCTTGGAGAAGACCCGGCCTGAT
TGCCTCATAGCTGATGCTTTCTTTCCCTTCGCAAGTGAATCTGCCGCTAGATTTGGTGTCACTATGCTA
GTCTTTCATGGGACTAGCTTCTTTGCCCTTTGTGCTAATGTGATTTTGTTTAAACATAAGCCTTTTATGT
CAGTTTCATCCAATGATGACTACTTTGTCATCCCAAATCTGCCTCATTTAATTAGGTTCACGCGGTTGCA
AATACCTCCTGAGATGCGTGAAAATAAGGAGAGTGACGTGTTAAGGATTGTGGAAGCAGGTTTTCTCT
CTCTCCATAGAAGTTATGGTGCTATTGTCAATAGCTTCTATGAGCTTGAGCCAGAATATGCTGATTATTA
TAGGAAGGTGTTGGGGAGAAGGGCATGGCATATAGGCCCTCTTTCCCTATGTAATAAGGATTTTGAAG
GTAAGTCTCAAAGAGGGAATAAAGCTTCAATCAACGGGCACGAGTGCCTAAAATGGCTTGATTCTAA
GGAACCCAACTCAGTTATTTATGTTTGTTTTGGGAGCTGGACTCGATTTTCATCCTCACAACTTCGTGA

GATTGCGATGGCTTTTGAGGCTTCAGGGCAACAATTCATTTGGGTTGTAAAGAAACCTATGAATGAAG
AGGAAGACAAGGGTAATGAACCATGGTTGCCAGAAGGGTTCGAGAAGAGGACCAGAGATCAGGGAC
TCATCATAAGAGGATGGGCACCCCAAGTGTTAATTTTGGAGCATGAAGCCATAGGAGGGTTCGTGACT
CATTGTGGGTGGAACTCGACATTGGAAGGCATAACGGCTGGTGTGCCCATGGTCACTTGGCCAGTGTT
TGCTGAACAGTTTTTTAACGAAAAGTTGGTGACTGAGATTTTGAAGGTTGGGATTCCCATTGGGGTTA
AAGAGTGGAGTATGCAAGTTGTTGAAAGTAATATTATTAAGCAGGAACAAATAGAGGCAGCATTCAGG
CAACTCATGGTGGATGAAGAGGCTCTCGAGATGCGAAGCAGAGCAAAGAAACTTAAGGAGATGGCA
TGCAAGGCTATGGAAGAAGGTGGCTCGTCTCACTCTGATCTCTGTGCCTTGTTGCAAGACTTGAGTTC
TTCTAGCCATAACCATCATTCTTGA

MGAERSQLHVAFFPWMAPGHTIPMIDMVRLFAARHVKTTVITTPLNAPNFTKSLNGTDATLEIFTFPSNEV
GLPQGIENVEDCTSPEMTMNFMRGTQLLRGPLEEFLEKTRPDCLIADAFFPFASESAARFGVTMLVFHGTS
FFALCANVILFKHKPFMSVSSNDDYFVIPNLPHLIRFTRLQIPPEMRENKESDVLRIVEAGFLSLHRSYGAIV
NSFYELEPEYADYYRKVLGRRAWHIGPLSLCNKDFEGKSQRGNKASINGHECLKWLDSKEPNSVIYVCF
GSWTRFSSSQLREIAMAFEASGQQFIWVVKKPMNEEEDKGNEPWLPEGFEKRTRDQGLIIRGWAPQVLIL
EHEAIGGFVTHCGWNSTLEGITAGVPMVTWPVFAEQFFNEKLVTEILKVGIPIGVKEWSMQVVESNIIKQE
QIEAAFRQLMVDEEALEMRSRAKKLKEMACKAMEEGGSSHSDLCALLQDLSSSSHNHHS

B6GT

>HU06G00241.1-R

ATGACAAAAATGGAGTTGGTGTTTGTCCCAATCCCAGGGATTGGGCACTTGCCGTCCACCGTCCAGTT
GGCCAAGCTCATCCTCCAGCGCGACCACCGTATTTCGGTGGTTGTCCTAATGATCAACGTCCCTCATG
ATGATAACGCCATAGTCAACGCTTACATTGATTCCCAGACACGTGATGCCCATCCTACCACCCGACTCA
CCTTCATCTCCCTACCCGCGCCCGCCAGCCCGCCCGACGCGTCCTCCCCTAAATTCTTCGCCAACATAA
TAGAGCTCCATAAGCCCTTCGTAAAGGAGGCTGTCCTCGACCGGATCCAATCCGGGTTGAGCAAGCC
CGTCGGGTTCATAGTCGACATGTTTTGCACCACCATGATTGACGTCGCCAATGAGCTCCACGTGGCCT
CTTACATCTTCTTCACCTCCGGAGCCATACTTCTCAATTTCATGTTCCGGATCGAATCATTGGTTGATGC
GGACAGGGTCAACTTTGCGGATGAGTTCGGTGCGGACCCGGATAAAGAAATGGATGTTCCCGGGTTC
AGGAACCGGGTACCGGGAAAGGTCATACCGTCGCCGTTTCTCGACAAGGAAGTTGTGTCCCAATGGA
ATCTCAACCCCATGAGGAGGTTTAGAGAATCCAAGGGCATTCTTGTAAATACGTACGCGGAGTTGGAA
TCCTACGCCACCCAAGCGTTGCTAGAGCAGGCAGAGGACAATAAGGTCCCTGCCGTTTATCCCGTGG
GCCCCATCTTGGTGCTCGACCGTGGGTCCCAGAAGGAGGAGGATGACTACTCCAATTCCATCATCATG
GAGTGGCTGGACCAGCAGCCCCCTTCCTCGGTGGTGTTCCTGTGCTTTGGGAGCATGGGAAGCTGGG
AGGAGGTCGAGGTCAAGGAGATAGCCAATGGCTTAGAACGAGCCGGCCACCGGTTCTTGTGGTCCCT
ACGCAAACCTCCTCCAGAGGGGAAGCTCGAGATGCCAAGTCAGCATGAGACCTTCGTGGATGCGCTC
CCTGAGGGGTTTCTGGAGAGGACGGCCCATCGAGGGAAGATCATCGCATGGGCCCCTCAAGTGGCCA
TCCTCGCCCACCAGGCCGTGGGTGGCTTCGTGTCGCATTGTGGGTGGAACTCGACATTGGAGAGCCT

GTGGTTCGGGGTTCCGATGGCTGTATGGCCACTGTACTCAGAGCAACAGTTGAATGCTTTCCTGCTGG
TGAAGGAGCTGGGAATTGCAGTCGAGATTAGGATGGATTATCGAACGGATTGGAAGACGAAAAAAGC
GAATTTTGTTGTGACAGCGGATGAAATAGAGAATGGGGTGAAGAAGCTGATGAGTATGGATGAGGAG
ATCAGAGGTAAAGTGAAGAAGATGAGTGAAAAAGGTAAAAAGGCTTTACAAGATGGAGGCTCCTCT
CATCATTGGTTAGGGCGTTTTATTGCAGATGTCTTGGATAACGTTGCCTAG

MTKMELVFVPIPGIGHLPSTVQLAKLILQRDHRISVVVLMINVPHDDNAIVNAYIDSQTRDAHPTTRLTFIS
LPAPASPPDASSPKFFANIIELHKPFVKEAVLDRIQSGLSKPVGFIVDMFCTTMIDVANELHVASYIFFTSGAI
LLNFMFRIESLVDADRVNFADEFGADPDKEMDVPGFRNRVPGKVIPSPFLDKEVVSQWNLNPMRRFRESK
GILVNTYAELESYATQALLEQAEDNKVPAVYPVGPILVLDRGSQKEEDDYSNSIIMEWLDQQPPSSVVFLC
FGSMGSWEEVEVKEIANGLERAGHRFLWSLRKPPPEGKLEMPSQHETFVDALPEGFLERTAHRGKIIAWA
PQVAILAHQAVGGFVSHCGWNSTLESLWFGVPMAVWPLYSEQQLNAFLLVKELGIAVEIRMDYRTDWKT
KKANFVVTADEIENGVKKLMSMDEEIRGKVKKMSEKGKKALQDGGSSHHWLGRFIADVLDNVA

>HU06G00241.1-W

ATGACAAAAATGGAGTTGGTGTTTGTCCCAATCCCAGGGATTGGGCACTTGCCGTCCACCGTCCAGTT
GGCCAAGCTCATCCTCCAGCGCGACCACCGTATTTCGGTGGTTGTCCTAATGATCAACGCCCCTCATG
ATGATAACGCCATAGTCAACGCTTACATTGATTCCCAGACACGTGATGCCCATCCTACCACCCGACTCA
CCTTCATCTCCCTACCCGCGCCCGCCAGCCCGCCCGACGCGTCCTCCCCTAAATTCTTCGCCAACATAA
TAGAGCTCCATAAGCCCTTCGTAAAGGAGGCTGTCCTCGACCGGATCCAATCCGGGTTGAGCAAGCC
CGTCGGGTTCATAGTCGACATGTTTTGCACCACCATGATTGACGTCGCCAATGAGCTCCACGTGGCCT
CTTACATCTTCTTCACCTCCGGAGCCATACTTCTCAATTTCATGTTCCGGATCGAATCATTGGTTGATGC
GGACAGGGTCAACTTTGCGGATGAGTTCGGTGCGGACCCGGATAAAGAAATGGATGTTCCCGGGTTC
AGGAACCGGGTACCGGGAAAGGTCATACCGTCGCCGTTTCTCGACAAGGAAGTTGTGTCCCAATGGA
ATCTCAACCCCATGAGGAGGTTTAGAGAATCCAAGGGCATTCTTGTAAATACGTACGCGGAGTTGGAA
TCCTACGCCACCCAAGCGTTGCTAGAGCAGGCAGAGGACAATAAGGTCCCTGCCGTTTATCCCGTGG
GCCCCATCTTGGTGCTCGACCGTGGGTCCCAGAAGGAGGAGGATGACTACTCCAATTCCATCATCATG
GAGTGGCTGGACCAGCAGCCCCCTTCCTCGGTGGTGTTCCTGTGCTTTGGGAGCATGGGAAGCTGGG
AGGAGGTCGAGGTCAAGGAGATAGCCAATGGCTTAGAACGAGCCGGCCACCGGTTCTTGTGGTCCCT
ACGCAAACCTCCTCCAGAGGGGAAGCTCGAGATGCCAAGTCAGCATGAGACCTTCGTGGATGCGCTC
CCTGAGGGGTTTCTGGAGAGGACGGCCCATCGAGGGAAGATCATCGCATGGGCCCCTCAAGTGGCCA
TCCTCGCCCACCAGGCCGTGGGTGGCTTCGTGTCGCATTGTGGGTGGAACTCGACATTGGAGAGCCT
GTGGTTCGGGGTTCCGATGGCTGCATGGCCACTGTACGCAGAGCAACAGTTGAATGCTTTCCTGCTGG
TGAAGGAGCTGGGAATTGCAGTCGAGATTAGGATGGATTATCGAACGGATTGGAAGACGAAAAAAGC
GAATTTTGTTGTGACAGCGGATGAAATAGAGAATGGGGTGAAGAAGCTGATGAGTATGGATGAGGAG
ATCAGAGGTAAAGTGAAGAAGATGAGTGAAAAAGGTAAAAAGGCTTTACAAGATGGAGGCTCCTCT
CATCATTGGTTAGGGCGTTTTATTGCAGATGTCTTGGATAACGTTGCCTAG

MTKMELVFVPIPGIGHLPSTVQLAKLILQRDHRISVVVLMINAPHDDNAIVNAYIDSQTRDAHPTTRLTFIS

LPAPASPPDASSPKFFANIIELHKPFVKEAVLDRIQSGLSKPVGFIVDMFCTTMIDVANELHVASYIFFTSGAI
LLNFMFRIESLVDADRVNFADEFGADPDKEMDVPGFRNRVPGKVIPSPFLDKEVVSQWNLNPMRRFRESK
GILVNTYAELESYATQALLEQAEDNKVPAVYPVGPILVLDRGSQKEEDDYSNSIIMEWLDQQPPSSVVFLC
FGSMGSWEEVEVKEIANGLERAGHRFLWSLRKPPPEGKLEMPSQHETFVDALPEGFLERTAHRGKIIAWA
PQVAILAHQAVGGFVSHCGWNSTLESLWFGVPMAAWPLYAEQQLNAFLLVKELGIAVEIRMDYRTDWKT
KKANFVVTADEIENGVKKLMSMDEEIRGKVKKMSEKGKKALQDGGSSHHWLGRFIADVLDNVA

>>HU06G00243.1-R

ATGGGGAAAACAGAGCTAGTGTTTGTCCCAACCCCAGGGATGGGGCATTTGCTGTCAACAGTGGAGG
TGGCCAAGCTCATCCTCCAGCGCGACCACCGCATTTCAGTCGTCATCCTAATCCTCAACTTCCCCGTC
GACAACTCAGTAGTCAACGCTTACATTGACTCCCAGTCACGACAGGCCGACCCCACTCGCCTCACCT
TCATCGCCCTGCCCCGGCTCGCCAACCCGATCGACCCGTCCTCTAAGAACTTCTTCGCCGACTTCATC
GAGGACCACAAGCCCCTTGTCAAGCAGGCCGTCCTTGATCGGATCGAGTCCGGGTCACCCAAACCTG
CCGGGTTGATCCTCGACATGTTCTGCATCACCATGGTCGACGTCGCCAATGAACTCCACGTCGACTCT
TACATGTTCTTCACCTCCGGAGCCTCAATGCTCAATCTCACGTACCGTGTCCAATCATTGGCCGATGAG
GAGGGAGAGAATGTTGTTGTGGATAGGTTAAGCGACCCGGAAAAAGAAATGGATGTTCCCGGGTTAA
GGAACCGGATACCCGGAAAATTCATACCCTCGGCGTTTCTCGATAGGGAAGGGGGGTTCGAGATGATT
CTCAATCTTGCTAGGAAGTTTCGAGAGTCAAAGGGCATTCTTATAAATACCTACATGGAGTTGGAATCC
TATCCTCTCCAAGCGTTGCTAGATCAGGCAAAGGATAACAAAATCCCAGCCGTCTATCCCGTGGGGCC
CATCTTGGAGCTCGACAGCAAAAGCCGCGGTGGATCCCAAAAGGAGGAACACGACTCCATCATGGAT
TGGCTCGACCAGCAGCCTCCATCGTCGGTGGTGTTCCTCTGCTTCGGGAGCATGGGAAGCTTCGATGA
GGTCCAGGTCAAGGAGACAGCCAACGGCCTCGACCGAGCTGGCCACCGGTTCCTGTGGTCCCTACGC
AGACCACCTCCGCAGGGGAGGTTCGGGATGCCAAGTCCTCACGAGACCTTCGTGGACGCTCTCCCTG
AGGGGTTCCTAGAGAGGACAGCCCACCGAGGGAAGATCATAGGGTGGGCCCCGCAAGTGGACGTCC
TGGCCCATCAGGCCATTGGAGGGTTCGTGTCTCATTGTGGGTGGAACTCGACTCTAGAGAGCCTGTGG
TTCGGGGTGCCAATGGCCACGTGGCCTTTATACGCAGAACAGCAACTGAATGCATTCACTCTGGTGGA
GGAGTTGGGGTTGGCAGTGGAGATTCGGATGGATTATCGGAGGGATTGGAGGACAAAACAGGCGAAT
TTTGTAGTGACGGCGGAGGAGATAGAGAATGGGGTGAAGAAGCTGATGAGTATGGATGAGGAGAAG
AGGGAGAAAGTTAGGGAGATGAGTGAAAATGGTAGGAAGGCTCTTCAAAATGGTGGCTCTTCTCACC
ATTGGCTGGGTCGCTTCGTTGCAAGTGTCTCTGATAACGTTGTTTAG

MGKTELVFVPTPGMGHLLSTVEVAKLILQRDHRISVVILILNFPVDNSVVNAYIDSQSRQADPTRLTFIALP
RLANPIDPSSKNFFADFIEDHKPLVKQAVLDRIESGSPKPAGLILDMFCITMVDVANELHVDSYMFFTSGAS
MLNLTYRVQSLADEEGENVVVDRLSDPEKEMDVPGLRNRIPGKFIPSAFLDREGGFEMILNLARKFRESK
GILINTYMELESYPLQALLDQAKDNKIPAVYPVGPILELDSKSRGGSQKEEHDSIMDWLDQQPPSSVVFLC
FGSMGSFDEVQVKETANGLDRAGHRFLWSLRRPPPQGRFGMPSPHETFVDALPEGFLERTAHRGKIIGWA
PQVDVLAHQAIGGFVSHCGWNSTLESLWFGVPMATWPLYAEQQLNAFTLVEELGLAVEIRMDYRRDWR
TKQANFVVTAEEIENGVKKLMSMDEEKREKVREMSENGRKALQNGGSSHHWLGRFVASVSDNVV

>HU06G00243.1-W

ATGGGGAAAACAGAGCTAGTGTTTGTCCCAACCCCAGGGATGGGGCATTTGCTGTCAACAGTGGAGG
TGGCCAAGCTCATCCTCCAGCGCGACCACCGCATTTCAGTCGTCATCCTAATCCTCAACTTCCCCGTC
GACAACTCAGTAGTCAACGCTTACATTGACTCCCAGTCACGACAGGCCGACCCCACTCGCCTCACCT
TCATCGCCCTGCCCCGGCTCGCCAACCCGATCGACCCGTCCTCCAAGAACTTCTTCGCCAACTTCATC
GAGGACCACAAGCCCCTTGTCAAGCAGGCCGTCCTTGATCGGATCGAGTCCGGGTCACCCAAACCTG
CCGGGTTGATCCTCGACATGTTCTGCATCACCATGGTCGACGTCGCCAATGAACTCCACGTCGACTCT
TACATGTTCTTCACCTCCGGAGCCTCAATGCTCAATCTCATGTACCGTGTCCAATCATTGACGGATGAG
GAGGGAGAGAATGTTGTTGTGGATAGGTTAAGCGACCCGGATAAAGAAATGGATGTTCCAGGGTTCA
GGAACCAGGTACCCGGAAAATTCATACCCGCGGTGTTTGTCGATAGGGAAGGGGGGTCCGAGATGAT
TCTCAATGTTGCTAGGAAGTCTCGAGAGTCTAAGGGCATTCTTATAAATACCTACATGGAGTTGGAATC
CTATCCTCTCCAAGCGTTGCTAGATCAGGCAAAGGATAACAAAATCCCAGCCGTCTATCCCGTGGGGC
CCATCTTGGAGCTCGACAGCAAAAGCCGCGGTGGATCCCAAAAGGAGGAACACGACTCCATCATGGA
TTGGCTCGACCAGCAGCCTCCATCGTCGGTGGCGTTCCTCTGCTTCGGGAGCATGGGAAGCTTCGATG
AGGTCCAGGTCAAGGAGACAGCCAACGGCCTCGACCGAGCTGGCCACCGGTTCCTGTGGTCCCTAC
GCAGACCACCTCCGCAGGGGAGGTTCGGGATGCCAAGTCCTCACGAGACCTTCGTGGACGCTCTCCC
TGAGGGGTTCCTAGAGAGGACAGCCCACCGAGGGAAGATCATAGGGTGGGCCCCGCAAGTGGACGT
CCTGGCCCATCAGGCCATTGGAGGGTTCGTGTCTCATTGTGGGTGGAACTCGACTCTAGAGAGCCTGT
GGTTCGGGGTGCCAATGGCCACGTGGCCTTTATACGCAGAACAGCAACTGAATGCATTCACTCTGGTG
GAGGAGTTGGGGTTGGCAGTGGAGATTCGGATGGATTATCGGAGGGATTGGAGGACAAAACAGGCG
AATTTTGTAGTGACGGCGGAGGAGATAGAGAATGGGGTGAAGAAGCTGATGAGTATGGATGAGGAGA
AGAGGGAGAAAGTTAGGGAGATGAGTGAAAATGGTAGGAAGGCTCTTCAAAACGGTGGCTCTTCTC
ACAATTGGCTGGGTCGCTTCGTTAGTGTCTTAGATAACGTTGTTTAG

MGKTELVFVPTPGMGHLLSTVEVAKLILQRDHRISVVILILNFPVDNSVVNAYIDSQSRQADPTRLTFIALP
RLANPIDPSSKNFFANFIEDHKPLVKQAVLDRIESGSPKPAGLILDMFCITMVDVANELHVDSYMFFTSGAS
MLNLMYRVQSLTDEEGENVVVDRLSDPDKEMDVPGFRNQVPGKFIPAVFVDREGGSEMILNVARKSRES
KGILINTYMELESYPLQALLDQAKDNKIPAVYPVGPILELDSKSRGGSQKEEHDSIMDWLDQQPPSSVAFL
CFGSMGSFDEVQVKETANGLDRAGHRFLWSLRRPPPQGRFGMPSPHETFVDALPEGFLERTAHRGKIIGW
APQVDVLAHQAIGGFVSHCGWNSTLESLWFGVPMATWPLYAEQQLNAFTLVEELGLAVEIRMDYRRDW
RTKQANFVVTAEEIENGVKKLMSMDEEKREKVREMSENGRKALQNGGSSHNWLGRFVSVLDNVV

>HU07G00076.1-R

ATGGAAGAATCACAAACTCACATACTGATCGTCCCAACGCCGGGTATGGGCCACCTTATTCCCCTGGT
CGAGCTCGCAAAGCGGCTCATAAACCAACGAACCCATAGGTTCACCTTCTCCTTCATCATCCCGACCC
AAGGGCCCGCCACCGAGGCCCAACGGGCCGCGGTCGGGTCTCTCCCATCGGGCCGGGCCGAGTGCT
CGTTCCTTCCTCCGGTCAGCATGGACGACCTGCCCCCCGACGCCAAGATAGAGACCAGGATCGCCCT
CACCCTGGCCCGGTCCCTCCCGGCTCTCCGGGCCGCGATTCGGGCAGTGGCTCGAGCCCGGATCCTC

GTCGTCGACCTCTTTGGAACTGATGCGTTTGAGCTCTGCCCGGAGTTCGGGCTCCGGCCCTACATCTT
CTACCCTACAACGGCTATGTGTCTCTCCTTCTTTTTCACCTCCCACAGTTAGACTCTCAAGTATCATGT
GAGTTTAGGGAGTTGGGTGAACCGGTGGAACTTCCCGGGTGTGTCCCGGTTCATGGAAAGGACCTCC
TGGACCCGGTTCAGGAGAGAGCAAATGATGCCTATAAATGGGTGCTTCATCACGCTAAGAGGTATAGG
TTGGCTGAGGGTATTTTGGTAAATAGCTTTATGGAGTTAGAGAAGGAGGCTATAGAGGCTTTAAAGAA
GCCTGAACCGGGAAAACCACCGGTTTACCCGGTTGGACCGCTGGTTCAGACCGGGTTGAGTGAGGG
TGTGGAGCGGTGTGAGTCTTTGGAGTGGTTGGATGAACAGCCTAGTGGGTCAGTCTTATATGTGTCTT
TTGGGAGTGGTGGGACCCTCTCCCATGATCAGTTGATTGAACTAGCTCATGGTTTGGAGATGAGTGGG
CTAAGATTTTTGTGGGTTGTGAGGAGCCCAAATGACGAAGCTGCCGATGCCACCTACTTCAACACCA
GCCAGGGTGGGATGGACCCCCTAGACTACCTACCTAAAGGGTTCCTAGAAAGGACCAAGAAGAGGG
GCCTAGTCGTCCCCACGTGGGCCCCACAGATCAAGGTCCTTAGCCACGAGTCCACCGGTGGGTTTTA
ACCCACTGTGGTTGGAACTCGATTTTGGAGGCTGTAGTTCATGGGGTACCCTTAATTGCCTGGCCGCT
CTACGCCGAGCAGAAAATGAACGCAGTAATGCTGACCGAGGGTCTAAAGGTTGCAATCAAGCCGGGT
GCCAATGAGACCGGCCTGGTTGGCCGAGGCGATATTGCAACTGTGGTAAGAGGCCTAATGGAAGGAG
AAGAAGGGAAAGAAATTCGAACCAGGATGAAGGGTTTGAAAGATGCAGCCTCTAGGGTGCTCAGCG
AAGAGGGCTCCTCCACCAAGGCACTCTGTGAAGTGGCTCACGGGTGGAGATCTCACTAA

MEESQTHILIVPTPGMGHLIPLVELAKRLINQRTHRFTFSFIIPTQGPATEAQRAAVGSLPSGRAECSFLPPVS
MDDLPPDAKIETRIALTLARSLPALRAAIRAVARARILVVDLFGTDAFELCPEFGLRPYIFYPTTAMCLSFFF
HLPQLDSQVSCEFRELGEPVELPGCVPVHGKDLLDPVQERANDAYKWVLHHAKRYRLAEGILVNSFMEL
EKEAIEALKKPEPGKPPVYPVGPLVQTGLSEGVERCESLEWLDEQPSGSVLYVSFGSGGTLSHDQLIELAH
GLEMSGLRFLWVVRSPNDEAADATYFNTSQGGMDPLDYLPKGFLERTKKRGLVVPTWAPQIKVLSHEST
GGFLTHCGWNSILEAVVHGVPLIAWPLYAEQKMNAVMLTEGLKVAIKPGANETGLVGRGDIATVVRGLM
EGEEGKEIRTRMKGLKDAASRVLSEEGSSTKALCEVAHGWRSH

>HU07G00076.1-W

ATGGAAGAATCACAAACTCACATACTGATCGTCCCAACGCCGGGTATGGGCCACCTTATTCCCCTGGT
CGAGCTCGCAAAGCGGCTCATAAACCAACGAACCCATAGGTTCACCTTCTCCTTCATCATCCCGACCC
AAGGGCCCGCCACCGAGGCCCAACGGGCCGCGGTCGGGTCTCTCCCATCGGGCCGGGCCGAGTGCT
CGTTCCTTCCTCCGGTCAGCATGGACGACCTGCCCCCCGACGCCAAGATAGAGACCAGGATCGCCCT
CACCCTGGCCCGGTCCCTCCCGGCTCTCCGGGCCGCGATTCGGGCAGTGGCTCGAGCCCGGATCCTC
GTCGTCGACCTCTTTGGAACTGATGCGTTTGAGCTCTGCCCGGAGTTCGGGCTCCGGCCCTACATCTT
CTACCCTACAACGGCTATGTGTCTCTCCTTCTTTTTCACCTCCCACAGTTAGACTCTCAAGTATCATGT
GAGTTTAGGGAGTTGGGTGAACCGGTGGAACTTCCCGGGTGTGTCCCGGTTCATGGAAAGGACCTCC
TGGACCCGGTTCAGGAGAGAGCAAATGATGCCTATAAATGGGTGCTTCATCACGCTAAGAGGTATAGG
TTGGCTGAGGGTATTTTGGTAAATAGCTTTATGGAGTTAGAGAAGGAGGCTATAGAGGCTTTAAAGAA
GCCTGAACCGGGAAAACCACCGGTTTACCCGGTTGGACCGCTGGTTCAGACCGGGTTGAGTGAGGG
TGTGGAGCGGTGTGAGTCTTTGGAGTGGTTGGATGAACAGCCTAGTGGGTCAGTCTTATATGTGTCTT
TTGGGAGTGGTGGGACCCTCTCCCATGATCAGTTGATTGAACTAGCTCATGGTTTGGAGATGAGTGGG

CTAAGATTTTTGTGGGTTGTGAGGAGCCCAAATGACAAAGCTGCCGATGCCACCTACTTCAACACCA
GCCAGGGTGGGATGGACCCCCTAGACTACCTACCTAAAGGGTTCCTAGAAAGGACCAAGAAGAGGG
GCCTAGTCGTCCCCACGTGGGCCCCACAGATCAAGGTCCTTAGCCACGAGTCCACCGGTGGGTTTTTA
ACCCACTGTGGTTGGAACTCGATTTTGGAGGCTGTGGTTCATGGGGTACCCTTAATTGCCTGGCCGCT
CTACGCCGAGCAGAAAATGAACGCAGTAATGCTGACCGAGGGTCTGAAGGTTGCAATCAAGCCGGGT
GCCAATGAGACCGGCCTGGTTGGCCGAGGCGATATTGCAACTGTGGTAAGAGGCCTAATGGAAGGAG
AAGAAGGGAAAGAAATTCGAACCAGGATGAAGGGTTTGAAAGATGCAGCCTCTAGGGTGCTCAGCG
AAGAGGGCTCCTCCACCAAGGCACTCTGTGAAGTGGCTCATGGGTGGAGATCTCACTAA

MEESQTHILIVPTPGMGHLIPLVELAKRLINQRTHRFTFSFIIPTQGPATEAQRAAVGSLPSGRAECSFLPPVS
MDDLPPDAKIETRIALTLARSLPALRAAIRAVARARILVVDLFGTDAFELCPEFGLRPYIFYPTTAMCLSFFF
HLPQLDSQVSCEFRELGEPVELPGCVPVHGKDLLDPVQERANDAYKWVLHHAKRYRLAEGILVNSFMEL
EKEAIEALKKPEPGKPPVYPVGPLVQTGLSEGVERCESLEWLDEQPSGSVLYVSFGSGGTLSHDQLIELAH
GLEMSGLRFLWVVRSPNDKAADATYFNTSQGGMDPLDYLPKGFLERTKKRGLVVPTWAPQIKVLSHEST
GGFLTHCGWNSILEAVVHGVPLIAWPLYAEQKMNAVMLTEGLKVAIKPGANETGLVGRGDIATVVRGLM
EGEEGKEIRTRMKGLKDAASRVLSEEGSSTKALCEVAHGWRSH

>HU11G01695.1-R

ATGGCAAAGCAGGAATGTCGTCCTCACATAGTGATCGTCCCCACACCAGGAATGGGGCACCTCATCCC
ACTAGTCCAGTTGGCCAACCGCATCCTTGACTCCTTCCCCTCTTTCACCCTCTCCTTCCTGGTCCCCAC
CATGGACCCACCATCCAAGGCCCAGGTTGCCACGCTGGCAGCCCTCCCCTTGAACCGGGCCTCCTCC
TCCTTCCTCCCTCCGGTCAGCACTCATGACCTCCCTCCCGACACCAAGATCGAGACCCGGATCTGTCT
GACCCTGGTCCGGTCCCTCCCGACTCTCCGCTCCCGCCTTGTGGACCTTGCCCGGGACCCCACCCGTC
GCCTCTCCGCCCTCGTCGTTGATCTCTTCGGGACCGACGCCTTCGATCTCTGCGCCGAGTTCGGCATG
AAGCCCTACATCTTCTTTCCCTCCACCGCCATGTGCCTCTCACTCTTCCTCCACCTCCCCGAGTTGGAT
GCCAAGGTTTCATGCGAGTTTAAGGACGTGGGTGAACCGGTCAGACTTCCCGGTTGTGTTCCCGTCC
GAGGAGAAGACCTCCTCGACCCGGTTCAGGACCGGAAGAACGAGACCTACAAATGGATACTTCATCA
TGCTAAGAGGTATCGGTTGGCTGAGGGTATTTTGGTAAATACATTTTTTGAGTTGGAGGAGGGTGCTG
TTAAAGCCCTGCAGCGGTCTGAACCGGGAAAGCCCCCAGTTCACCCGGTCGGGCCGCTGGTTCGGCC
GGGATCGGGTAAAGGAAAGGATGATGGGTCGGAGTGTCTGAGGTGGCTGGATGAGCAGCCACGTGG
GTCCGTGTTATTTGTCTCGTTTGGGAGTGGTGGGACCCTCACCTGTGATCAGTTAGCTGAGTTGGCCC
ATGGTTTGGAAATGAGTGAGCAGAGGTTTTTGTGGGTGGTGAGGGCCCCAAATGACACTACCAATGC
CACCTTCTTTAACAGTGGCGAAGCTGATAGGGACCCATTAGGCTTTTTACCTCAAGGGTTCCTAGAAA
GGACTAAAGAAAGGGGCCTAGTCATCCCCTCGTGGGCCCCACAAATAAAGGTCCTTAGCCACGAGTC
CACTGGTGGGTTCCTGACACATTGTGGGTGGAACTCCATTTTGGAGAGCGTGGATCATGGGGTACCGT
TCATTGCCTGGCCGCTCTACGCTGAGCAGCGGATGAACGCAGTAATGCTAACTGAGGATCTCAAGGTC
GCGTTACAGCCTAAGGCCGGACCCAATGGGCTCATCAGCCGAGGGGAGATCGCGGTTACAGTAAGAG
GTCTAATGGAGGGTGAAGAAGGGAAAAGAATTCGGAACAGGATGAAAGACTTGAAAGATGCAGCTG
CTAGAGCACTTAGTGACAATGGATCCTCCACAAAGGCACTGTCCGAAGCGGCCCTTACGTGGAAATC

TCAATAA

MAKQECRPHIVIVPTPGMGHLIPLVQLANRILDSFPSFTLSFLVPTMDPPSKAQVATLAALPLNRASSSFLPP
VSTHDLPPDTKIETRICLTLVRSLPTLRSRLVDLARDPTRRLSALVVDLFGTDAFDLCAEFGMKPYIFFPSTA
MCLSLFLHLPELDAKVSCEFKDVGEPVRLPGCVPVRGEDLLDPVQDRKNETYKWILHHAKRYRLAEGIL
VNTFFELEEGAVKALQRSEPGKPPVHPVGPLVRPGSGKGKDDGSECLRWLDEQPRGSVLFVSFGSGGTLT
CDQLAELAHGLEMSEQRFLWVVRAPNDTTNATFFNSGEADRDPLGFLPQGFLERTKERGLVIPSWAPQIK
VLSHESTGGFLTHCGWNSILESVDHGVPFIAWPLYAEQRMNAVMLTEDLKVALQPKAGPNGLISRGEIAVT
VRGLMEGEEGKRIRNRMKDLKDAAARALSDNGSSTKALSEAALTWKSQ

>HU11G01695.1-W

ATGGCAAACCAGGAATGTCGTCATCACATAGTGATCATCCCCACACCAGGAATGGGGCACCTCATCCC
ACTAGTCCAGTTGGCCAACCGCATCCTTGACTCCTTCCCCTCTTTCACCCTCTCCTTCCTGGTCCCCAC
CATGGACCCACCATCCAAGGCCCAGGTTGCCACGCTGGCAGCCCTCCCCTTGGACCGGGCCTCCTCC
TCCTTCCTCCCTCCGGTCAGCACTCATGACCTCCCTCCCGACACCAAGATCGAGACCCGGATCTGTCT
GACCCTGGTCCGGTCCCTCCCGACTCTCCGCTCCCGCCTTGTGGACCTTGCCCGGGACCCTACCCGTC
GCCTCTCCGCCCTCGTAGTTGATCTCTTCGGGACCGACGCCTTCGATCTCTGCGCCGAGTTCGGCATG
AAGCCCTACATCTTCTTTCCCTCCACCGCCATGTGCCTCTCACTCTTCCTCCACCTCCCCGAGTTGGAT
GCCAAGGTTTCATGCGAGTTTAAGGACGTGGGTGAACCGGTCAGACTTCCCGGTTGTGTTCCCGTCC
GAGGAGAAGACCTCCTCGACCCGGTTCAGGACCGGAAGAACGAGGCCTACAAATGGATACTTCATCA
TGCTAAGAGGTATCGGTTGGCTGAGGGTATTTTGGTAAATACATTTTTTGAGTTGGAGGAGGGTGCTG
TTAAAGCCCTGCAGCGGTCTGAACCGGGAAAGCCCCCAGTTCACCCGGTCGGGCCGCTGGTTCGGCC
GGGATCGGGTAAAGGAAAGGATGATGGGTCGGAGTGTCTGAGGTGGCTGGATGAGCAGCCACGTGG
GTCCGTGTTATTTGTCTCGTTTGGGAGTGGTGGGACCCTCACCTGTGATCAGTTAGCTGAGTTGGCCC
ATGGTTTGGAAATGAGTGAGCAGAGGTTTTTGTGGGTGGTGAGGGCCCCAAATGACACTACCAATGC
CACCTTCTTTAACAGTGGCGAAGCTGATAGGGACCCATTAGGCTTTTTACCTCAAGGGTTCCTAGAAA
GGACTAAAGAAAGGGGCCTAGTCATCCCCTCGTGGGCCCCACAAATAAAGGTCCTTAGCCACGAGTC
CACTGGTGGGTTCCTGACACATTGTGGGTGGAACTCCATTTTGGAGAGCGTGGATCATGGGGTACCGT
TCATTGCCTGGCCGCTCTACGCTGAGCAGCGGATGAACGCAGTAATGCTAACTGAGGATCTCAAGGTC
GCGTTACAGCCTAAGGCCGGACCCAATGGGCTCATCAGCCGAGGGGAGATCGCGGTTACAGTAAGAG
GTCTAATGGAGGGTGAAGAAGGGAAAAGAATTCGGAACAGGATGAAAGACTTGAAAGATGCAGCTG
CTAGAGCACTTAGTGACAATGGATCCTCCACAAAGGCACTGTCCGAAGCGGCCCTTACGTGGAAATC
TCAATAA

MANQECRHHIVIIPTPGMGHLIPLVQLANRILDSFPSFTLSFLVPTMDPPSKAQVATLAALPLDRASSSFLPP
VSTHDLPPDTKIETRICLTLVRSLPTLRSRLVDLARDPTRRLSALVVDLFGTDAFDLCAEFGMKPYIFFPSTA
MCLSLFLHLPELDAKVSCEFKDVGEPVRLPGCVPVRGEDLLDPVQDRKNEAYKWILHHAKRYRLAEGIL
VNTFFELEEGAVKALQRSEPGKPPVHPVGPLVRPGSGKGKDDGSECLRWLDEQPRGSVLFVSFGSGGTLT
CDQLAELAHGLEMSEQRFLWVVRAPNDTTNATFFNSGEADRDPLGFLPQGFLERTKERGLVIPSWAPQIK

VLSHESTGGFLTHCGWNSILESVDHGVPFIAWPLYAEQRMNAVMLTEDLKVALQPKAGPNGLISRGEIAVT
VRGLMEGEEGKRIRNRMKDLKDAAARALSDNGSSTKALSEAALTWKSQ

>HU11G01696.1-R

ATGTACTTCACCACCAACGCCATGTGCCTGCTCTTCTTGCTCAACGTTCCACGGTTGGATGAAACGGT
ATCATGTGAGTACCCGGATATGCCCGACCCGCTTGTTTACCCGGGTGCGTGCCACTCTA..CGGTAAG..
GACTTTGTGGACCCTTGCATGGACCGCAAGGACGAGTCCTACAGAGTGCTGCTCTATCACGTCAAGA
GGTATGCCCTGACTGAGGGTATTTTTGTCAATACATTTTTCGATCTGGAGCCCGGTGCTATCCGGGCTT
TGCAAAGTGAGGATCCGAATTGGCCCCCGGTCTACCCAGTTGGCCCGATTATTCAATCGGCTCAAAGT
GCGGAAACCGATGGAACTGAATGTTTGAGTTGGTTAGACCGACAGCCAACCGGGTCGGTTCTATTTGT
GTCGTTTGGGAGCGGAGGGACTTTGTCAAAAGGGCAACTAAATGAATTGGCAATCGGGCTCGAGAAA
AGTGGCCAGAGATTTGTGTGGGTAGTCCGAAGCCCAAACGAACACTCGTCATTTGGGTCATTTTTCAG
TGCCCAGAATCAAGATGATCCGTTTGGGTTCCTACCAGATGGATATCTCCATCGGATTAAAGACCGTGG
GCTGTTGGTTCCATCTTGGGCCCCACAGATAAGGGTTCTGAGCCATGGCTCCACAGGAGGGTTCTTGA
CTCACTGTGGGTGGAACTCGGTCTTAGAAAGTATAATGAATGGAGTGCCCTTGATTGTGTGGCCGTTG
TATGCAGAACAAAGGATGAATGCAGTAATGTTTAACCAAGGGCTTAAGGTCGCATTAAGGCCTAGTGC
TAATGAGCGTGGGCTTGTTGAGGCGGATGAGATTGCTAGAGTCGTCAAGGAGCTGATGGAGGGCAAT
GAAGGGAAAACAGCTCGCCATGGAATGAAGGCGTTAAGCGATTTGGCTAAGAAGACGATAAGTGAA
GATGGAGATTCATCAAAATTGCTAGCTGAGGTCACTCAGAAATGGAGTCACGGCAAAATTCTCCAGA
AAGATTACCCAGTTGATCTTCCATGA

MYFTTNAMCLLFLLNVPRLDETVSCEYPDMPDPLVLPGCVPLYGKDFVDPCMDRKDESYRVLLYHVKRY
ALTEGIFVNTFFDLEPGAIRALQSEDPNWPPVYPVGPIIQSAQSAETDGTECLSWLDRQPTGSVLFVSFGSG
GTLSKGQLNELAIGLEKSGQRFVWVVRSPNEHSSFGSFFSAQNQDDPFGFLPDGYLHRIKDRGLLVPSWA
PQIRVLSHGSTGGFLTHCGWNSVLESIMNGVPLIVWPLYAEQRMNAVMFNQGLKVALRPSANERGLVEA
DEIARVVKELMEGNEGKTARHGMKALSDLAKKTISEDGDSSKLLAEVTQKWSHGKILQKDYPVDLP

>HU11G01696.1-W

ATGTACTTCACCACCAACGCCATGTGCCTGCTCTTCTTGCTCAACGTTCCACGGTTGGATGAAACGGT
ATCATGTGAGTACCCGGATATGCCCGACCCGCTTGTTTACCCGGGTGCGTGCCACTCTA..CGGTAAG..
GACTTTGTGGACCCTTGCATGGACCGCAAGGACGAGTCCTACAGAGTGCTGCTCTATCACGTCAAGA
GGTATGCCCTGACTGAGGGTATTTTTGTCAATACATTTTTCGATCTGGAGCCCGGTGCTATCCGGGCTT
TGCAAAGTGAGGATCCGAATTGGCCCCCGGTCTACCCAGTTGGCCCGATTATTCAATCGGCTCAAAGT
GCGGAAACCGATGGAACTGAATGTTTGAGTTGGTTAGACCGACAGCCAACCGGGTCGGTTCTATTTGT
GTCGTTTGGGAGCGGAGGGACTTTGTCAAAAGGGCAACTAAATGAATTGGCAATCGGGCTCGAGAAA
AGTGGCCAGAGATTTGTGTGGGTAGTCCGAAGCCCAAACGAACACTCGTCATTTGGGTCATTTTTCAG
TGCCCAGAATCAAGATGATCCGTTTGGGTTCCTACCAGATGGATATCTCCATCGGATTACAGACCGTGG
GCTGTTGGTTCCATCTTGGGCCCCACAGATAAGGGCTCTGAGCCATGGCTCCACAGGAGGGTTCTTGA
CTCACTGTGGGTGGAACTCGGTCTTAGAAAGTATAATGAATGGAGTGCCCTTGATTGTGTGGCCGTTG

TATGCAGAACAAAGGATGAATGCAGTAATGTTTAACCAAGGGCTTAAGGTCGCATTAAGGCCTAGTGC
TAATGAGCGTGGGCTTGTTGAGGCGGATGAGATTGCTAGAGTCGTCAAGGAGCTGATGGAGGGCAAT
GAAGGGAAAACAGCTCGCCATGGAATGAAGGCGTTAAGCGATTTGGCTAAGAAGACGATAAGTGAA
GATGGAGATTCATCAAAATTGCTAGCTGAGGTCACTCAGAAATGGAGTCACAGCAAAATTCTCCAGA
AAGATTACCCAGTTGATCTTCCATGA

MYFTTNAMCLLFLLNVPRLDETVSCEYPDMPDPLVLPGCVPLYGKDFVDPCMDRKDESYRVLLYHVKRY
ALTEGIFVNTFFDLEPGAIRALQSEDPNWPPVYPVGPIIQSAQSAETDGTECLSWLDRQPTGSVLFVSFGSG
GTLSKGQLNELAIGLEKSGQRFVWVVRSPNEHSSFGSFFSAQNQDDPFGFLPDGYLHRITDRGLLVPSWA
PQIRALSHGSTGGFLTHCGWNSVLESIMNGVPLIVWPLYAEQRMNAVMFNQGLKVALRPSANERGLVEA
DEIARVVKELMEGNEGKTARHGMKALSDLAKKTISEDGDSSKLLAEVTQKWSHSKILQKDYPVDLP

>HU11G01697.1-R

ATGCATGTACGTGCCAATTTAGCCATGGAAGCTCTGGTTCACCAAATAGCCATAATCCCAAGCCCTGG
AATGGGCCATTTAATCCCCTTCGTCGAGTTCGCCAAGCTCCTCGTCTCCCGCTTCAACCTCTCAGTCTC
TCTCCTCATCCCCACTTTGGGGCAGCCCACCCGGGCCCAAGTCTCCTTCCTCGACTCCCTCCCTTCAG
CCATTTCCCACACCTTCCTTCCTCCTATTGACCCGGTCCAACTGCCCCAAGGCGCAGGCCATGAGGTC
ACCATCTCCCTTGTCCTGACCCATTCTCTCTCCTCCATCCGGACCTCCCTGGTCCGCATGGCCCACCTC
GTTGTTGCCCTTGTCACCGACCTGTTCGGTACCGACATCTTCGATGTTGCGAGGGAGCTGGGGATTCC
TCCCTACATGTATTTTACATGCTCAGCTATGAACTTGATGTTCTCCTTCCATCTCCCTAGGTTGGATGAA
ACGGTGTCGTGTGACTACAAGGATATGCCCGGCCCGCTTCTCCTGCCCGGGTGCGTGCCCATTCACGG
CCAAGATTTTGACGACCCGTGTCAGGACCGTAAGGGTGAGTGTTACAGGGTGTTGCTTCATCATTCAA
AGAGGTACGTATTGGCTGAGGGTATTTTGTCAATACGTTTTTGATCTCGAGCCGGGTGCTCTCCGGA
CTTTGCAAACTGAGAATCCGAATAGGCCTCCGGCTTACCCGATTGGCCCCATACTTCAGTCGGGTCAA
GCGGGGGACATCGATGGGTCGGAGTGCTTGAGTTGGTTGGAGCGGCAACCAACCGGGTCGGTCCTAT
ATGTGTCGTTTGGGAGTGGGGGGACTTTATCCAGAGAGCAGATCATTGAATTGGCAATTGGGCTTGAG
GGAAGTGAGCAGAGGTTTTTGTGGGTGGTTAGAGGCGCAAGTGATCAACTCTCTCATGGGTCATTTTT
CGGTGCCCAAAGTCTAGATGACCCATTTGGGTTCTTACCAAATGGATATCTCAATCGGATCAAAGATCG
CGGGCTATTAGTCCCTTCATGGGCCCCCCAGATAAAGGTCCTTAGCCACAAGTCCACAGGCGGGTTCT
TAACACATTGTGGATGGAACTCGATTCTGGAGAGCATAATTAACGGGGTACCCTTGATTGCATGGCCAT
TGTATGCGGAACAAAGGATGAATGCAGTAATGCTTACCCAAGGGCTTAAGGTCGCATTAAGGCCTATT
GCTAATGCGAATGGCCTTTTTAGGGCAGATGAGATTGCTAGAGTCATCAAAGTTTTAATGAAAGGCGA
AGAAGGGGAAATAATTCGCCATCGGATGAAAGAGCTAAGTGACTTGGCTAAGAAAGCTATGCGTGAA
GATGGAGATTCCTCAAAGTCACTTGCCCAGGTCGCTCACAAGTGGAATCAAAACAAACTTCTCCAGA
AAGATTAA

MHVRANLAMEALVHQIAIIPSPGMGHLIPFVEFAKLLVSRFNLSVSLLIPTLGQPTRAQVSFLDSLPSAISHT
FLPPIDPVQLPQGAGHEVTISLVLTHSLSSIRTSLVRMAHLVVALVTDLFGTDIFDVARELGIPPYMYFTCSA
MNLMFSFHLPRLDETVSCDYKDMPGPLLLPGCVPIHGQDFDDPCQDRKGECYRVLLHHSKRYVLAEGIF

VNTFFDLEPGALRTLQTENPNRPPAYPIGPILQSGQAGDIDGSECLSWLERQPTGSVLYVSFGSGGTLSREQ
IIELAIGLEGSEQRFLWVVRGASDQLSHGSFFGAQSLDDPFGFLPNGYLNRIKDRGLLVPSWAPQIKVLSHK
STGGFLTHCGWNSILESIINGVPLIAWPLYAEQRMNAVMLTQGLKVALRPIANANGLFRADEIARVIKVLM
KGEEGEIIRHRMKELSDLAKKAMREDGDSSKSLAQVAHKWNQNKLLQKD

>HU11G01697.1-W

ATGCATGTACGTGCCAATTTAGCCATGGAAGCTCTGGTTCACCAAATAGCCATAATCCCAAGCCCTGG
AATGGGCCATTTAATCCCCTTCGTCGAGTTCGCCAAGCTCCTCGTCTCCCGCTTCAACCTCTCAGTCTC
TCTCCTCATCCCCACTTTGGGGCAGCCCACCCGGGCCCAAGTCTCCTTCCTCGACTCCCTCCCTTCAG
CCATTTCCCACACCTTCCTTCCTCCTATTGACCCGGTCCAACTGCCCCAAGGCGCAGGCCATGAGGTC
ACCATCTCCCTTGTCCTGACCCATTCTCTCTCCTCCATCCGGACCTCCCTGGTCCGCATGGCCCACCTC
GTTGTTGCCCTTGTCACCGACCTGTTCGGTACCGACATCTTCGATGTTGCGAGGGAGCTGGGGATTCC
TCCCTACATGTATTTTACATGCTCAGCTATGAACTTGATGTTCTCCTTCCATCTCCCTAGGTTGGATGAA
ACGGTGTCGTGTGACTACAAGGATATGCCCGGCCCGCTTCTCCTGCCCGGGTGCGTGCCCATTCACGG
CCAAGATTTTGACGACCCGTGTCAGGACCGTAAGGGTGAGTGTTACAGGGTGTTGCTTCATCATTCAA
AGAGGTACGTATTGGCTGAGGGTATTTTTGTCAATACGTTTTTGATCTCGAGCCGGGTGCTCTCCGGA
CTTTGCAAACTGAGAATCCGAATAGGCCTCCGGCTTACCCGATTGGCCCCATACTTCAGTCGGGTCAA
GCGGGGGACATCGATGGGTCGGAGTGCTTGAGTTGGTTGGAGCGGCAACCAACCGGGTCGGTCCTAT
ATGTGTCGTTTGGGAGTGGGGGGACTTTATCCAGAGAGCAGATCATTGAATTGGCAATTGGGCTTGAG
GGAAGTGAGCAGAGGTTTTTGTGGGTGGTTAGAGGCGCAAGTGATCAACTCTCTCATGGGTCATTTTT
CGGTGCCCAAAGTCTAGATGACCCATTTGGGTTCTTACCAAATGGATATCTCAATCGGATCAAAGATCG
CGGGCTATTAGTCCCTTCATGGGCCCCCCAGATAAAGGTCCTTAGCCACAAGTCCACAGGCGGGTTCT
TAACACATTGTGGATGGAACTCGATTCTGGAGAGCATAATTAACGGGGTACCCTTGATTGCATGGCCAT
TGTACGCGGAACAAAGGATGAATGCAGTAATGCTTACCCAAGGGCTTAAGGTCGCATTAAGGCCTATT
GCTAATGCGAATGGCCTTTTTAGGGCAGATGAGATTGCTAGAGTCATCAAAGTTTTAATGAAAGGCGA
AGAAGGGGAAATAATTCGCCATCGGATGAAAGAGCTAAGTGACTTGGCTAAGAAAGCTATGCGTGAA
GATGGAGATTCCTCAAAGTCACTTGCCCAGGTCGCTCACAAGTGGAATCAAAACAAACTTCTCCAGA
AAGATTAA

MHVRANLAMEALVHQIAIIPSPGMGHLIPFVEFAKLLVSRFNLSVSLLIPTLGQPTRAQVSFLDSLPSAISHT
FLPPIDPVQLPQGAGHEVTISLVLTHSLSSIRTSLVRMAHLVVALVTDLFGTDIFDVARELGIPPYMYFTCSA
MNLMFSFHLPRLDETVSCDYKDMPGPLLLPGCVPIHGQDFDDPCQDRKGECYRVLLHHSKRYVLAEGIF
VNTFFDLEPGALRTLQTENPNRPPAYPIGPILQSGQAGDIDGSECLSWLERQPTGSVLYVSFGSGGTLSREQ
IIELAIGLEGSEQRFLWVVRGASDQLSHGSFFGAQSLDDPFGFLPNGYLNRIKDRGLLVPSWAPQIKVLSHK
STGGFLTHCGWNSILESIINGVPLIAWPLYAEQRMNAVMLTQGLKVALRPIANANGLFRADEIARVIKVLM
KGEEGEIIRHRMKELSDLAKKAMREDGDSSKSLAQVAHKWNQNKLLQKD

>HU11G01698.1-R

ATGGAAGAACCACACATTGTCATAGTCCCAAGCCCAGGAATGGGTCACATGATCCCCCTAGTTGAGTT

CGCAAAGCTCCTAGTCTCCCGCTTCAACTTCTCAGTCTCTCTCATCCTCCCTACCTCCACCCAACCCAC
CAAGGCCCAAACCTCCTTCCTCAATGCCCTCCCTCCTTCCATTTCTCACACCTTCCTTCCCCCGGTCGA
CCCGGCCCTTTTACCCGATGGTGTGGCCCATGAGGTAACCATCCACCACACCCTTGTCCACTCTCTCC
CATCTCTACGGGCCGCCCTCTCCGACTTGGCCCAACGGATCCCGGTCGCCGGTCTCGTCGCCGACCTC
TTTGGGACTGACTGCTTCGACGTGGCCCGGGAGCTTGGAATTCTTCCTTACATGTACTTCACATCCAA
CGCCATGTGCCTGCTGTTCCTCCTCAACCTTCCCCCGTTGGACAAAACGGTGTCGTGTGAGTATCCGG
ATATGCCTAACCCGCTTGTCTTACCCGGGTGCGTGCCGCTTTACGGTAAGGACTTTCCGGACCGTTGTA
CGGACCGCAATGACGAGTCCTATAGGGTGGTGCTTTATCACGTGAAGAGGTATGTTTTGTCTGAGGGT
ATTTTTGTCAACACGTTTTTTGATCTCGAGCGCGGTGCTATCCGGGCTTTGCAAACCGAGGGTCCGAA
CTGGCCCCCGGTTTATCCAGTCGGGCCGGTTATTCAGTCGGGTCAAGGTGTGGAGACCGATGGATCAT
CTGAGTGTTTGAGCTGGTTAGACCCACAACCAACCGGGTCGGTTTTATTTGTGTCGTTTGGGAGTGGA
GGCACTTTATCAAAAAGGCAACTTAATGAATTGGCAATTGGGTTGGAGAAAAGTGGGCAGAGGTTCT
TGTGGGTGGTTAGAAGCCCAAACGATCACTCCTCTAATGCGTCGTTCTTCACTGCCCAAAGTCAGGAC
GACCCGCTCGGGTTCCTACCCCATGGATATCTTGATCGGATCAAAGACCGTGGGCTGTTGGTTCCGTC
ATGGGGCCCACAGATAAAGATTCTAAGCCATGCGTCCACTGGCGGGTTCTTGACTCACTGTGGGTGGA
ATTCAGTTCTCGAGAGCATAATCAATGGGGTGCCCTTAATTGCATGGCCATTATATGCAGAACAAAGAA
TGAATGCAGTAATGCTTAACCAAGGGCTTAAGGTTGCAATAAGGCCTAATGCTAATGGACGCGGGCTA
TATGAGGCCGATGAAATTGCCAGAGTCGTCAAAGAGTTGATGGAGGGTGAGGAAGGGAAGGGAGCT
CGTCATCGGATGAAGGAAGTGAGTGATTTGGCTAAGAAGGCGATAAGCAAAGATGGAGATTCTTCAA
AGTTACTTGCTGAGGTTGCTCATCAATGGAGTCATAGCAAGATTGTCCACTAA

MEEPHIVIVPSPGMGHMIPLVEFAKLLVSRFNFSVSLILPTSTQPTKAQTSFLNALPPSISHTFLPPVDPALLP
DGVAHEVTIHHTLVHSLPSLRAALSDLAQRIPVAGLVADLFGTDCFDVARELGILPYMYFTSNAMCLLFLL
NLPPLDKTVSCEYPDMPNPLVLPGCVPLYGKDFPDRCTDRNDESYRVVLYHVKRYVLSEGIFVNTFFDLE
RGAIRALQTEGPNWPPVYPVGPVIQSGQGVETDGSSECLSWLDPQPTGSVLFVSFGSGGTLSKRQLNELAI
GLEKSGQRFLWVVRSPNDHSSNASFFTAQSQDDPLGFLPHGYLDRIKDRGLLVPSWGPQIKILSHASTGGF
LTHCGWNSVLESIINGVPLIAWPLYAEQRMNAVMLNQGLKVAIRPNANGRGLYEADEIARVVKELMEGEE
GKGARHRMKEVSDLAKKAISKDGDSSKLLAEVAHQWSHSKIVH

>HU11G01698.1-W

ATGGAAGAACCACACATTGTCATAGTCCCAAGCCCAGGAATGGGTCACCTGATCCCCCTAGTTGAGTT
CGCAAAGCTCCTAGTCTCCCACTTCAACTTCTCAGTCTCTCTCATCCTCCCCACCTCCACCCAGCCCA
CCAAGGCCCAAACCTCCTTCCTCAATGCCCTCCCTCCTTCCATTTCTCACACCTTCCTTCCCCCGGTCG
ACCCGGCCCTTTTACCCGATGGTGTGGCCCATGAGGTAACCATCCACCACACCCTTGTCCACTCTCTC
CCATCTCTACGGACCGCCCTCTCCGACTTGGCCCAATGGACCCCGGTCGCCGGTCTCGTCGCCGACCT
CTTTGGGACTGACTGCTTCGACGTGGCCCGGGAGCTTGGAATTCTTCCTTACATGTACTTCACATCCA
ACGCCATGTGCCTGCTGTTCCTCCTCAACCTTCCCCCGTTGGACAAAACGGTGTCGTGTGAGTATCCG
GATATGGCTAACCCGCTTGTCTTACCCGGGTGCGTGCCGCTTTACGGTAAGGACTTTCCGGACCGTTG
TATGGACCGCAATGACGAGTCCTATAGGGTGGTGCTTTATCATGTGAAGAGGTATGTTTTGTCTGAGGG

TATTTTTGTCAACACGTTTTTTGATCTCGAGCGCGGTGCTATCCGGGCTTTGCAAACCGAGGGTCCGA
ACTGGCCCCCGGTTTATCCAGTCGGGCCGGTTATTCAGTCGGGTCAAGGTGTGGAGACCGATGGATCA
TCTGAGTGTTTGAGCTGGTTAGACCGACAACCAACCGGGTCGGTTTTATTTGTGTCGTTTGGGAGTGG
AGGCACTTTATCAAAAAGGCAACTTAATGAATTGGCAATTGGGTTGGAGAAAAGTGGGCAGAGGTTC
TTGTGGGTGGTTAGAAGCCCAAACGATCACTCCTCTAACGCGTCGTTCTTCACTGCCCAAAGTCAGG
ACGACCCGCTCGGGTTCCTACCCCATGGATATCTTGATCGGATCAAAGACCGTGGGCTGTTGGTTCCG
TCATGGGGCCCACAGATAAAGATTCTAAGCCATGCGTCCACTGGCGGGTTCTTGACTCACTGTGGGTG
GAATTCAGTTCTCGAGAGCATAATCAATGGGGTGCCCTTAATTGCATGGCCATTATATGCAGAACAAAG
AATGAATGCAGTAATGCTTAACCAAGGGCTTAAGGTTGCAATAAGGCCTAATGCTAATGGACGCGGGC
TATATGAGGCCGATGAAATTGCCAGAGTCGTCAAAGAGTTGATGGAGGGTGAGGAAGGGAAGGGAG
CTCGTCATCGGATGAAGGAAGTGAGTGATTTGGCTAAGAAGGCGATAAGCAAAGATGGAGATTCTTC
AAAGTTACTTGCTGAGGTTGCTCATCAATGGAGTCATAGCAAGATTGTCCACTAA

MEEPHIVIVPSPGMGHLIPLVEFAKLLVSHFNFSVSLILPTSTQPTKAQTSFLNALPPSISHTFLPPVDPALLP
DGVAHEVTIHHTLVHSLPSLRTALSDLAQWTPVAGLVADLFGTDCFDVARELGILPYMYFTSNAMCLLFLL
NLPPLDKTVSCEYPDMANPLVLPGCVPLYGKDFPDRCMDRNDESYRVVLYHVKRYVLSEGIFVNTFFDLE
RGAIRALQTEGPNWPPVYPVGPVIQSGQGVETDGSSECLSWLDRQPTGSVLFVSFGSGGTLSKRQLNELA
IGLEKSGQRFLWVVRSPNDHSSNASFFTAQSQDDPLGFLPHGYLDRIKDRGLLVPSWGPQIKILSHASTGG
FLTHCGWNSVLESIINGVPLIAWPLYAEQRMNAVMLNQGLKVAIRPNANGRGLYEADEIARVVKELMEGE
EGKGARHRMKEVSDLAKKAISKDGDSSKLLAEVAHQWSHSKIVH

cDOPA5GT

>HU05G01532.1-R

ATGGAGAGGGTTGAGCAGGACAGCAAGCTAAGGAAGAGATTGCTCCTCTTCCCTCTACCTTTCCAAC
GCCATATAACACCAATGCTCCATTTAGCCATCCTTCTCTACTCCAAAGGCTTCTCCATCACCATAATCCA
AACCAACTACAAGTCTCTCAATCCCACCACTTTCCCTCACTTCACCTTTCATTTCATCAATGATGGCTT
GTCAATGTTCGAGGAAGCCGCAGCCTCCCAATCCCCTTTCGTCCTCCTTTCAGCCCTTAATGCGGACT
GCTTAGAGCCCTTCAGAGAATGCCTGTCTTGGGTTATGAATGGTGGTGATGCCAATGATCAGGAAGCA
GTTGCTTGCCCGATAGCAGACACTACATGGCAGTTTGCTGGGTCTGTGGCTGATAGCTTCAAACTGCC
TAGAGTCGCACTTAGGACTGGTTGTATATCTGCCTTTGTTGTCCATGATTCTCTTTCGCTTCTCCGAGA
AAAGGGCTATTTTCCTACACAAGAAACAAGATTGGATGAACTGGTGCCAGAGCTTTCACCACTAAAA
GTGAAAGACCTTCCCTTGGAGGCGCAGCATGACTTTCTCGCGGCTCTAATCAAAGAGACGATATCCTC
TAAGGGAACCATCTACAACTCGTTTGAGGAGCTGGAAAGTTCTGCATTTTCCCGGGTTCAACGAGACT
TGCCCATCCCTGTCTTCCCAATAGGTCCACTACACAGTCATTCACCAGCTTGGTCACCCAACACAAGT
GATCAAGACCAAACCACCATGTCCTGGCTAGACACACAAGCACCCAACACTGTTCCATACGTGAGCT
TTGGGAGCGTCATGACAATGAGCAAATCTGATCTTGTTGAATTAGCATGGGGTTTAGCCCACAGCATG
CAGCCATTCTTGTGGGTGGTTCGATCAGGGTTAGTTAAGGGGGTCCAAGAAAATGACCCACTGTCAG

AGGGCTTCTTTGACTACTTGGATGGAAGAGAGCACATTGTTAAATGGGCTCATCAAAATGAAGTTCTG
GCTCACCCCGCAGTTGGAGGCTTCTTGAGTCATTGTGGGTGGAACTCGACTCTGGAGAGCATCTATGC
GGGGGTGCATATGATTTGTTTGCCCCTGTTTGCTGATCAGGCAATCAATGCGAGGTAA

MERVEQDSKLRKRLLLFPLPFQRHITPMLHLAILLYSKGFSITIIQTNYKSLNPTTFPHFTFHFINDGLSMFEE
AAASQSPFVLLSALNADCLEPFRECLSWVMNGGDANDQEAVACPIADTTWQFAGSVADSFKLPRVALRT
GCISAFVVHDSLSLLREKGYFPTQETRLDELVPELSPLKVKDLPLEAQHDFLAALIKETISSKGTIYNSFEEL
ESSAFSRVQRDLPIPVFPIGPLHSHSPAWSPNTSDQDQTTMSWLDTQAPNTVPYVSFGSVMTMSKSDLVEL
AWGLAHSMQPFLWVVRSGLVKGVQENDPLSEGFFDYLDGREHIVKWAHQNEVLAHPAVGGFLSHCGW
NSTLESIYAGVHMICLPLFADQAINAR

>HU05G01532.1-W

ATGGAGAGGGTTGAGCAGGACAGCAAGCTAAGGAAGAGATTGCTCCTCTTCCCTCTACCTTTCCAAC
GCCATATAACACCAATGCTCCATTTAGCCATCCTTCTCTACTCCAAAGGCTTCTCCATCACCATAATCCA
AACCAACTACAAGTCTCTCAATCCCACCACTTTCCCTCACTTCACCTTTCATTTCATCAATGATGGCTT
GTCAATGTTCGAGGAAGCCGCAGCCTCCCAATCCCCTTTCGTCCTCCTTTCAGCCCTTAATGCGGACT
GCTTAGAGCCCTTCAGAGAATGCCTGTCTTGGGTTATGAATGGTGGTGATGCCAATGATCAGGAAGCA
GTTGCTTGCCCGATAGCAGACACTACATGGCAGTTTGCTGGGTCTGTGGCTGATAGCTTCAAACTGCC
TAGAGTCGCACTTAGGACTGGTTGTATATCTGCCTTTGTTGTCCATGATTCTCTTTCGCTTCTCCGAGA
AAAGGGCTATTTTCCTACACAAGAAACAAGATTGGATGAACTGGTGCCAGAGCTTTCACCACTAAAA
GTGAAAGACCTTCCCTTGGAGGCGCAGCATGACTTTCTCGCGGCTCTAATCAAAGAGACGATATCCTC
TAAGGGAACCATCTACAACTCGTTTGAGGAGCTGGAAAGTTCTGCATTTTCCCGGGTTCAACGAGACT
TGCCCATCCCTGTCTTCCCAATAGGTCCACTACACAGTCATTCACCAGCTTGGTCACCCAACACAAGT
GATCAAGACCAAACCACCATGTCCTGGCTAGACACACAAGCACCCAACACTGTTCCATACGTGAGCT
TTGGGAGCGTCATGACAATGAGCAAATCTGATCTTGTTGAATTAGCATGGGGTTTAGCCCACAGCATG
CAGCCATTCTTGTGGGTGGTTCGATCAGGGTTAGTTAAGGGGGTCCAAGAAAATGACCCACTGTCAG
AGGGCTTCTTTGACTACTTGGATGGAAGAGAGCACATTGTTAAATGGGCTCATCAAAATGAAGTTCTG
GCTCACCCCGCAGTTGGAGGCTTCTTGAGTCATTGTGGGTGGAACTCGACTCTGGAGAGCATCTATGC
GGGGGTGCATATGATTTGTTTGCCCCTGTTTGCTGATCAGGCAATCAATGCGAGGTAA

MERVEQDSKLRKRLLLFPLPFQRHITPMLHLAILLYSKGFSITIIQTNYKSLNPTTFPHFTFHFINDGLSMFEE
AAASQSPFVLLSALNADCLEPFRECLSWVMNGGDANDQEAVACPIADTTWQFAGSVADSFKLPRVALRT
GCISAFVVHDSLSLLREKGYFPTQETRLDELVPELSPLKVKDLPLEAQHDFLAALIKETISSKGTIYNSFEEL
ESSAFSRVQRDLPIPVFPIGPLHSHSPAWSPNTSDQDQTTMSWLDTQAPNTVPYVSFGSVMTMSKSDLVEL
AWGLAHSMQPFLWVVRSGLVKGVQENDPLSEGFFDYLDGREHIVKWAHQNEVLAHPAVGGFLSHCGW
NSTLESIYAGVHMICLPLFADQAINAR

>HU07G00240.1-R

ATGGACTCTGCAACAGAGCATATACTGATGCTCCCATACATGGCGCAGGGTCACATTCGCCCGTTCATC
GAGCTAGCCCACCGCCTCCAAGCCCGAACTGAAACCAGAACCCGAACCCACTTCACCATCACCATCC

TCACCACCCCTCTCAACGCTGAGCTCATCCGACGCCAATTCACCACCACCTCCACCATCCAAATTGCC
GAGCTCCCCTTCAACGCCGCCGACCATGGCCTCCCTGCTGGCGTCGAGAACACCGACAAGCTCCCGT
TCCCCCTCGTCGTCAACCTCTTCCACGCCTCCTCCTCGCTTGAACCCCACCTCCGCCACTTCATCGCC
ACCCGCTTCTCCCCCTGTAAACCCCCTCTCTGCATTGTCTTCGACGTCTTCCTGGGATGGGTTGACGCC
GTCGCTAGGAGCTTTGGATCCACCGGAGTCGCCTTCACCACCGGCGGCGCGTATGGGACGGCGGCGT
ATTTGTCACTGTGGCTTGATCTACCTCACCGAAACTTCTCAGACGATGAGGACTTCCCTTTGCCTGGAT
TCCCGGAGAACCACCGCTATCGCCGCTCCCAGCTCCACCGGTTCTTAAGATACGCTGATGGGTCGGAT
CAGTGGTCGCGGTTTTTCCAGCCCCAGCTGCGGTTCTCAATTGGTAGCCTCGGATGGCTCTGTAATTC
CGTCGAGGAGATTGAGCCTCTGGGGTTTGAAATTCTTCGGAATTACCTCAAATCACCGGTCTGGGCAG
TCGGGCCGTTGATTCCGGATCCTCCGCCGGTGGATAAATCATCATCTTCTTGTGACAAATGCATCCAGT
GGTTAAATTCCCACAAAGCCGATTCCGTTTTATACATCTCATTCGGGTCGCAGAATACGATTTCCCCGA
CCCAGATGATGGAGCTGGCTATGGGTTTAGAGCTAAGTGGGAAGCCATTTTTGTGGGTGATCCGGCCT
CCGTTCGGGTTCGACATCAACGGAGAATTCCGACTCGAATGGCTTCCGGATGGGTTCGAGGAGCGCG
TGAAGAACAACAACCAGGGGATGTTGGTGCATAAATGGGGGCCTCAAATGGAGATACTGGGTCATGA
ATCAACGGGTGGGTTTCTCAGCCATTGTGGGTGGAACTCGGTGTTGGAAGGACTGAGAGAGGGACTA
CCGATGATAGCGTGGCCATTGGCTGGGGAGCAGGCGTACAATTCGAAGATGCTGGTGGAGGAGATGG
CGGTGGCAGTGGAGCTGACGACGGGGTTGGAAGGGAAGGTGACTAAGGAGGCGGTGAAGAGGGTG
GTGGAGCTGGTGTTGGGGAGAGAGGAAGGGAGTGGTGGCGAGGAGATGAAGAAGAAGGCGGTTGA
AGCAGGGAAGAAGCTTAGAGATGCATTAAGAGAAGAGGAAGATGGGTTAGGGTTTAAGGGATCTTCG
ATTAAAGCAATGGATGAGTTTTGGATGCCATAGTTTCCGGCAGGGATCGACCAGCTGCTTGA

MDSATEHILMLPYMAQGHIRPFIELAHRLQARTETRTRTHFTITILTTPLNAELIRRQFTTTSTIQIAELPFNA
ADHGLPAGVENTDKLPFPLVVNLFHASSSLEPHLRHFIATRFSPCKPPLCIVFDVFLGWVDAVARSFGSTGV
AFTTGGAYGTAAYLSLWLDLPHRNFSDDEDFPLPGFPENHRYRRSQLHRFLRYADGSDQWSRFFQPQLRF
SIGSLGWLCNSVEEIEPLGFEILRNYLKSPVWAVGPLIPDPPPVDKSSSSCDKCIQWLNSHKADSVLYISFGS
QNTISPTQMMELAMGLELSGKPFLWVIRPPFGFDINGEFRLEWLPDGFEERVKNNNQGMLVHKWGPQME
ILGHESTGGFLSHCGWNSVLEGLREGLPMIAWPLAGEQAYNSKMLVEEMAVAVELTTGLEGKVTKEAVK
RVVELVLGREEGSGGEEMKKKAVEAGKKLRDALREEEDGLGFKGSSIKAMDEFLDAIVSGRDRPAA

>HU07G00240.1-W

ATGGACTCTGCAACAGAGCATATACTGATGCTCCCATACATGGCGCAGGGTCACATTCGCCCGTTCATC
GAGCTAGCCCACCGCCTCCAAGCCCGAACTGAAACCAGAACCCGAACCCACTTCACCATCACCATCC
TCACCACCCCTCTCAACGCTGAGCTCATCCGACGCCAATTCACCACCACCTCCACCATCCAAATTGCC
GAGCTCCCCTTCAACGCCGCCGACCATGGCCTCCCTGCTGGCGTCGAGAACACCGACAAGCTCCCGT
TCCCCCTCGTCGTCAACCTCTTCCACGCCTCCTCCTCGCTTGAACCCCACCTCCGCCACTTCATCGCC
ACCCGCTTCTCCCCCTGTAAACCCCCTCTCTGCATTGTCTTCGACGTCTTCCTGGGATGGGTTGACGCC
GTCGCTAGGAGCTTTGGATCCACCGGAGTCGCCTTCACCACCGGCGGCGCGTATGGGACGGCGGCGT
ATTTGTCACTGTGGCTTGATCTACCTCACCGAAACTTCTCAGACGATGAGGACTTCCCTTTGCCTGGAT
TCCCGGAGAACCACCGCTATCGCCGCTCCCAGCTCCACCGGTTCTTAAGATACGCTGATGGGTCGGAT

CAGTGGTCGCGGTTTTTCCAGCCCCAGCTGCGGTTCTCAATTGGTAGCCTCGGATGGCTCTGTAATTC
CGTCGAGGAGATTGAGCCTCTGGGGTTTGAAATTCTTCGGAATTACCTCAAATCACCGGTCTGGGCAG
TCGGGCCGTTGATTCCGGATCCTCCGCCGGTGGATAAATCATCATCTTCTTGTGACAAATGCATCCAGT
GGTTAAATTCCCACAAAGCCGATTCCGTTTTATACATCTCATTCGGGTCGCAGAATACGATTTCCCCGA
CCCAGATGATGGAGCTGGCTATGGGTTTAGAGCTAAGTGGGAAGCCATTTTTGTGGGTGATCCGGCCT
CCGTTCGGGTTCGACATCAACGGAGAATTCCGACTCGAATGGCTTCCGGATGGGTTCGAGGAGCGCG
TGAAGAACAACAACCAGGGGATGTTGGTGCATAAATGGGGGCCTCAAATGGAGATACTGGGTCATGA
ATCAACGGGTGGGTTTCTCAGCCATTGTGGGTGGAACTCGGTGTTGGAAGGACTGAGAGAGGGACTA
CCGATGATAGCGTGGCCATTGGCTGGGGAGCAGGCGTACAATTCGAAGATGCTGGTGGAGGAGATGG
CGGTGGCAGTGGAGCTGACGACGGGGTTGGAAGGGAAGGTGACTAAGGAGGCGGTGAAGAGGGTG
GTGGAGCTGGTGTTGGGGAGAGAGGAAGGGAGTGGTGGCGAGGAGATGAAGAAGAAGGCGGTTGA
AGCAGGGAAGAAGCTTAGAGATGCATTAAGAGAAGAGGAAGATGGGTTAGGGTTTAAGGGATCTTCG
ATTAAAGCAATGGATGAGTTTTTGGATGCCATAGTTTCCGGCAGGGATCGACCAGCTGCTTGA

MDSATEHILMLPYMAQGHIRPFIELAHRLQARTETRTRTHFTITILTTPLNAELIRRQFTTTSTIQIAELPFNA
ADHGLPAGVENTDKLPFPLVVNLFHASSSLEPHLRHFIATRFSPCKPPLCIVFDVFLGWVDAVARSFGSTGV
AFTTGGAYGTAAYLSLWLDLPHRNFSDDEDFPLPGFPENHRYRRSQLHRFLRYADGSDQWSRFFQPQLRF
SIGSLGWLCNSVEEIEPLGFEILRNYLKSPVWAVGPLIPDPPPVDKSSSSCDKCIQWLNSHKADSVLYISFGS
QNTISPTQMMELAMGLELSGKPFLWVIRPPFGFDINGEFRLEWLPDGFEERVKNNNQGMLVHKWGPQME
ILGHESTGGFLSHCGWNSVLEGLREGLPMIAWPLAGEQAYNSKMLVEEMAVAVELTTGLEGKVTKEAVK
RVVELVLGREEGSGGEEMKKKAVEAGKKLRDALREEEDGLGFKGSSIKAMDEFLDAIVSGRDRPAA

>HU08G02083.1-R

ATGGAGAACTCAAGCTATAATAATAATAAGCAGGTGAAAATCGAGAAGCCTCATGCAATAATCGTAGC
CCTTCCTTTGCAAGGCCATGTAATCCCAACCGTCCAACTGGCCCTGAAATTAGCATCCAAGGGGTTCT
CTATCACCTTCATCAACGCCGAGAACATCCACCACCGCATCACCGCCGCAGCTTCCGTTGCAGACGGC
AGGCTCTTCGGTGGTGATGAGGATATAGAATACGTCACCGTATCGGACGGGCTGCCGGTGGAGTACGA
TAGGTCGCTGAACCATGACCGGTGGGTGGGGGCTACTATCCACTGCTACCCGGCCCACGTTGAGATGG
CTGTGGAGAACATCCTCAAAGCTCGACCGTCGGATAGGTTTTGCTTGATTGCTGACGTGTACTCTGTT
TGGGCGTCTGACATGGCTAAGAAGTTTGGGTTGGTACACGTGGCCTTTTGGACTGAGGCTGCTTTGGT
TTTTGATATCTACCTTCATCGCCATTTGCTCCAACAGAATGGTCATGTTGGTCCCGGTGCCCGCAAGGA
CACAATAGACTATATACCAGGAGTAAAATCAATAAAACCAACGGACCTGATGTCATATCTCCAAGGAG
AGGACCTGACAACTATCTGCCATCAGTTCATTTTCCAGTGCATGGACGATGCTAAACGGGCCGATTTT
GTATTAGGCAATACGGTCCAAGAGCTTGAAGATCGCCCAATTTCTGCTATTCAGGCCCAAGTCCCAAT
CTTTGCAATTGGGCCTTTGTTTTCACCTGGATTTCCTAAGAGCCCAGTAGCCACTAGCTTATGGGCCGA
GTCAGATTGCACTCAATGGCTAAACTCCAAGCCTCCTAGCTCGGTTTTGTACGTCTCATTTGGTAGCTA
TGCGCATATTAGAAAGGATGAGCTCGTAGAGATTGCTAATGGAATCAAGCTTAGTAGGGTCAACTTCC
TTTGGGCCCTCCGGCCAGATATGGTGAGCTCCGATGACTCAGACCCACTTCCAAATGGGTTTAAGGAT
GAGGTCCAGGGCCGAGGATGGTTGTCACTTGGACCAGTCAGACTGAGGTGTTGTATAATAAAGCGA

TTGGAGGGTTCTTGACACACTGTGGGTGGAACTCGATACTTGAGAGTATATGGTGTGAAGTTCCCCTA
CTGTGCTTTCCTCTACTGACGGATCAGTTTACTAATCGAAAGCTGATTGTGGAAGAGTGGAAGATTGG
ATGCAATCTTTGTGACGAGAATAATTCACCAACGACCGAGGAGGTGTCGAGGAAAATCAAGGATTTG
ATGCATGGAAAAAGAGGGCACGAGCTCAAGGAGAAGATTAAGAAGATCAAGCAAATTGTAGAGAAT
GCAAGGCTGCCTCAAGGATCATCAGAGAGGAATGTTGACGAGTTCATGAAGGAATTGGGTGTGGCTT
TTGACAAGAAATTGGCAACAAATGCCTAA

MENSSYNNNKQVKIEKPHAIIVALPLQGHVIPTVQLALKLASKGFSITFINAENIHHRITAAASVADGRLFG
GDEDIEYVTVSDGLPVEYDRSLNHDRWVGATIHCYPAHVEMAVENILKARPSDRFCLIADVYSVWASDM
AKKFGLVHVAFWTEAALVFDIYLHRHLLQQNGHVGPGARKDTIDYIPGVKSIKPTDLMSYLQGEDLTTIC
HQFIFQCMDDAKRADFVLGNTVQELEDRPISAIQAQVPIFAIGPLFSPGFPKSPVATSLWAESDCTQWLNSK
PPSSVLYVSFGSYAHIRKDELVEIANGIKLSRVNFLWALRPDMVSSDDSDPLPNGFKDEVQGRGMVVTWT
SQTEVLYNKAIGGFLTHCGWNSILESIWCEVPLLCFPLLTDQFTNRKLIVEEWKIGCNLCDENNSPTTEEVS
RKIKDLMHGKRGHELKEKIKKIKQIVENARLPQGSSERNVDEFMKELGVAFDKKLATNA

>HU08G02083.1-W

ATGGAGAACTCAAGCTATAATAATAATAAGCAGGTGAAAATCGAGAAGCCTCATGCAATAATCGTAGC
CCTTCCTTTGCAAGGCCATGTAATCCCAACCGTCCAACTGGCCCTGAAATTAGCATCCAAGGGGTTCT
CTATCACCTTCATCAACGCCGAGAACATCCACCACCGCATCACCGCCGCAGCTTCCGTTGCAGACGGC
AGGCTCTTCGGTGGTGATGAGGATATAGAATACGTCACCGTATCGGACGGGCTGCCGGTGGAGTACGA
TAGGTCGCTGAACCATGACCGGTGGGTGGGGGCTACTATCCACTGCTACCCGGCCCACGTTGAGATGG
CTGTGGAGAACATCCTCAAAGCTCGACCGTCGGATAGGTTTTGCTTGATTGCTGACGTGTACTCTGTT
TGGGCGTCTGACATGGCTAAGAAGTTTGGGTTGGTACACGTGGCCTTTTGGACTGAGGCTGCTTTGGT
TTTTGATATCTACCTTCATCGCCATTTGCTCCAACAGAATGGTCATGTTGGTCCCGGTGCCCGCAAGGA
CACAATAGACTATATACCAGGAGTAAAATCAATAAAACCAACGGACCTGATGTCATATCTCCAAGGAG
AGGACCTGACAACTATCTGCCATCAGTTCATTTTCCAGTGCATGGACGATGCTAAACGGGCCGATTTT
GTATTAGGCAATACGGTCCAAGAGCTTGAAGATCGCCCAATTTCTGCTATTCAGGCCCAAGTCCCAAT
CTTTGCAATTGGGCCTTTGTTTTCACCTGGATTTCCTAAGAGCCCAGTAGCCACTAGCTTATGGGCCGA
GTCAGATTGCACTCAATGGCTAAACTCCAAGCCTCCTAGCTCGGTTTTGTACGTCTCATTTGGTAGCTA
TGCGCATATTAGAAAGGATGAGCTCGTAGAGATTGCTAATGGAATCAAGCTTAGTAGGGTCAACTTCC
TTTGGGCCCTCCGGCCAGATATGGTGAGCTCCGATGACTCAGACCCACTTCCAAATGGGTTTAAGGAT
GAGGTCCAGGGCCGAGGGATGGTTGTCACTTGGACCAGTCAGACTGAGGTGTTGTATAATAAAGCGA
TTGGAGGGTTCTTGACACACTGTGGGTGGAACTCGATACTTGAGAGTATATGGTGTGAAGTTCCCCTA
CTGTGCTTTCCTCTACTGACGGATCAGTTTACTAATCGAAAGCTGATTGTGGAAGAGTGGAAGATTGG
ATGCAATCTTTGTGACGAGAATAATTCACCAACGACGGAGGAGGTGTCGAGGAAAATCAAGGATTTG
ATGCATGGAAAAAGAGGGCACGAGCTCAAGGAGAAGATTAAGAAGATCAAGCAAATTGTAGAGAAT
GCAAGGCTGCCTCAAGGATCATCAGAGAGGAATGTTGACGAGTTCATGAAGGAATTGGGTGTGGCTT
TTGACAAGAAATTGGCAACAAATGCCTAA

MENSSYNNNKQVKIEKPHAIIVALPLQGHVIPTVQLALKLASKGFSITFINAENIHHRITAAASVADGRLFG
GDEDIEYVTVSDGLPVEYDRSLNHDRWVGATIHCYPAHVEMAVENILKARPSDRFCLIADVYSVWASDM
AKKFGLVHVAFWTEAALVFDIYLHRHLLQQNGHVGPGARKDTIDYIPGVKSIKPTDLMSYLQGEDLTTIC
HQFIFQCMDDAKRADFVLGNTVQELEDRPISAIQAQVPIFAIGPLFSPGFPKSPVATSLWAESDCTQWLNSK
PPSSVLYVSFGSYAHIRKDELVEIANGIKLSRVNFLWALRPDMVSSDDSDPLPNGFKDEVQGRGMVVTWT
SQTEVLYNKAIGGFLTHCGWNSILESIWCEVPLLCFPLLTDQFTNRKLIVEEWKIGCNLCDENNSPTTEEVS
RKIKDLMHGKRGHELKEKIKKIKQIVENARLPQGSSERNVDEFMKELGVAFDKKLATNA

>HU09G01874.1-R

ATGGACATCTGGGAAATTCTCACTGAAAAGCCCTTTCAGCTTGATCCTGATATCCCAACAAACATTCCC
TCCAGTGAAGGCTGCTTCACCTCAGAGTTCTTAGACTTCTTATATAAACAAATAGAGCTCCTGGACTTC
CAATCTGGAAAACTGTTCAATACGTGCAGGGTTATAGAAGCTAGATATTTGGAGCTACTGGAAAGGCT
ACCAATTTATGGAAACATGAAAACCTTCGCCATTGGACCTCTTAATCCAGTGCAAATCAGAAGGACAA
GTGAGAAGCAACGACATGAATGTCTAGAATGGCTAGATAAACAAGAGGAGAATTCTGTGATATATGTC
TCCTTCGGATCGACAACAGCGATGACTGATGAGCAGATCACAGAGTTGGCGAAAGGGTTGGAACAAA
GCGGGCAGAAGTTTATATGGGTGCTTAGAAAAGCTGATAAAGGCGACATTTTTATGGGAGATGAAGAG
GGAAGGCCTCAGCAGCCAGAAGGGTATGAAGAGAGGGTGAAGGACAGGGGAATGGTGGTGAGAGA
CTGGGCACCACAGCTGGAAATTCTCGGTCACCCATCAACAGGTGGGTTCATGAGTCACTGTGGGTGG
AATTCATGCATAGAGAGTATCAGCATGGGTGTACCAGTTGTGGCATGGCCGATGCACTCTGACCAGCC
TCGGAATGCCACTTTGTTGACAGAAGTGCTAAGAATTGGATTATTGATCAAGGATTGCGTGCATCTAGC
TGAGCTGGTGACATCAGATACCATAGAGGATGCTGTGAGACGACTGATGACATCACAGGAAGGAGAG
GAGATGAAGAAAAAGGCAGAAAAACTAGGCGAGGCTGTTCGAGCTTCAGTCGCTGAAGGTGGTGCT
TCTCGCTTGGAAATGGATTCTTTCATTGCTCATATTACCAGATAA

MDIWEILTEKPFQLDPDIPTNIPSSEGCFTSEFLDFLYKQIELLDFQSGKLFNTCRVIEARYLELLERLPIYGN
MKTFAIGPLNPVQIRRTSEKQRHECLEWLDKQEENSVIYVSFGSTTAMTDEQITELAKGLEQSGQKFIWVL
RKADKGDIFMGDEEGRPQQPEGYEERVKDRGMVVRDWAPQLEILGHPSTGGFMSHCGWNSCIESISMGV
PVVAWPMHSDQPRNATLLTEVLRIGLLIKDCVHLAELVTSDTIEDAVRRLMTSQEGEEMKKKAEKLGEAV
RASVAEGGASRLEMDSFIAHITR

>HU09G01874.1-W

ATGGACATCTGGGAAATTCTCACTGAAAAGCCCTTTCAGCTTGATCCTGATATCCCAACAAACATTCCC
TCCAGTGAAGGCTGCTTCACCTCAGAGTTCTTAGACTTCTTATATAAACAAATAGAGCTCCTGGACTTC
CAATCTGGAAAACTGTTCAATACGTGCAGGGTTATAGAAGCTAGATATTTGGAGCTACTGGAAAGGCT
ACCAATTTATGGAAACATGAAAACCTTCGCCATTGGACCTCTTAATCCAGTGCAAATCAGAAGGACAA
GTGAGAAGCAGCAACATGAATGTCTAGAATGGCTAGATAAACAAGAGGAGAATTCTGTGATATATGTC
TCCTTCGGATCGACAACAGCGATGACTGATGAGCAGATCACAGAGTTGGCGAAAGGGTTGGAACAAA
GCGGGCAGAAGTTTATATGGGTGCTTAGAAAAGCTGATAAAGGCGACATTTTTATGGGAGATGAAGAG
GGAAGGCCTCAGCAGCCAGAGGGGTATGAAGAGAGGGTAAAGGACAGGGGAATGGTGGTGAGAGA

CTGGGCACCACAGCTGGAAATTCTCGGTCACCCATCAACAGGTGGGTTCATGAGTCACTGTGGGTGG
AATTCATGCATAGAGAGTATCAGCATGGGTGTACCAGTTGTGGCATGGCCGATGCACTCTGACCAGCC
TCGGAATGCCACTTTGTTGACAGAAGTGCTAAGAATTGGATTATTGATCAAGGATTGCGTGCATCTAGC
TGAGCTGGTGACATCAGATACCATAGAGGATGCTGTGAGAAGACTGATGACATCACAGGAAGGAGAG
GAGATGAAGAAAAGGGCAGAAAAACTAGGCGAGGCTGTTCGAGCTTCAGTCGCTGAAGGTGGTGCT
TCTCGCTTGGAAATGGATTCTTTCGTTGCTCATATTACCAGATAA

MDIWEILTEKPFQLDPDIPTNIPSSEGCFTSEFLDFLYKQIELLDFQSGKLFNTCRVIEARYLELLERLPIYGN
MKTFAIGPLNPVQIRRTSEKQQHECLEWLDKQEENSVIYVSFGSTTAMTDEQITELAKGLEQSGQKFIWVL
RKADKGDIFMGDEEGRPQQPEGYEERVKDRGMVVRDWAPQLEILGHPSTGGFMSHCGWNSCIESISMGV
PVVAWPMHSDQPRNATLLTEVLRIGLLIKDCVHLAELVTSDTIEDAVRRLMTSQEGEEMKKRAEKLGEAV
RASVAEGGASRLEMDSFVAHITR

>HU09G01875.1-R

ATGGAAAGCCAGAGTTGCTGCCAAAATGGTATCCAGAAGGGCCAAAAATCTGCTGAAGTAGCTGTG
GTCATGGTGCCCTTCCTAGCCCAAGGCCATCTCAACCAGTTCCTCCATCTCTCTCATCATCTCGTAGC
CTCCTATGGCATACCAGTTCACTACGCTGGGTCTGCCATCCACAACCGCCAAGCCAAGCTACGGTTC
CATGGTTGGGACCCGGAAACTTCCAACAGAATTGAGTTCCACGACCTACAGCTCCCTCCTTACAGCT
CACCCTCTCCAAACCCAAATGCAGCTACACATTTCCCTGCGCACCTCCAGCCTCTTTTCGATGCTTCT
ATGCAACTCCGCGAACCCCTTTTCCAAGTCTGCCGAGACCTCTCGACCAAGTTCAGGAGAATTGTCA
TAATTCATGATACCATAGTAGGTTCAATTGTTCAAGATGTAAAACTCATCCCAAATGCCGAGGCTTA
CGTGCTTGTTCCAACCTCGGCTTTTCAAACATCTATGGGCATCACGAAAAATCTTACTGAAAAGCCC
TTTCAGCTTGACCCTGATATCCCAACAAACATTCCCTCCAATGAAGGCTGCTTCACCTCAGAGTTCTT
AGACTTCGCATATAAACAAATAGAGCTCCTGGACTTCCAATCTGGAAAACTGTTCAATACATGCAGG
GTTATAGAAGATAGATATTTGGAGCTACTGGAAAGGCTACCAATTTATGGAAACATGAAAACCTTCG
CCATTGGACCTCTTAATCCAGTGCAAATCAGAAGGACAAGTGAGAAGCAACGACATGAATGTCTAG
AATGGCTAGATAAACAAGAGGAGAATTCTGTGATATATGTCTCCTTCGGATCGACAACAGCGATGA
CTGATGAGCAGATCACAGAGTTGGCGAAAGGGTTGGAACAAAGCGGGCAGAAGTTTATATGGGTGC
TTAGAAAAGCTGATAAAGGCGACATTTTTATGGGAGATGAAGAGGGAAGGCCTCAGCAGCCAGAAG
GGTATGAAGAGAGGGTAAAGGACTGGGGAATGGTGGTGAGATACTGGGCACCACAGCTGGAAATTC
TCGGTCACCCATCAACAGGTGGGTTCATGAGTCACTGTGGGTGGAATTCATGCATAGAGAGTATCAG
CATGGGTGTACCAGTTGTGGCATGGCCGATGCACTCTGACCAGCCTAGGAATGCCACTTTGTTGACA
GAAGTGCTAAGAATTGGATTATTGATCAAGGATTGCGTGCATCTAGCTGAGCTGGTGACATCAGATA
CCATAGAGGATGCTGTGAGACGACTGATGACATCACAGGAAGGAGAGGAGATGAAGAAAAAGGCA
GAAAAACTAGGCGAGGCTGTTCGAGCTTCAGTCGCTGAAGGTGGTGCTTCTCGCTTGGAAATGGATT
CTTTCATTGCTCATATTACCAGATAA

MESQSCCQNGIQKGQKSAEVAVVMVPFLAQGHLNQFLHLSHHLVASYGIPVHYAGSAIHNRQAKLRFH
GWDPETSNRIEFHDLQLPPYSSPSPNPNAATHFPAHLQPLFDASMQLREPLFQVCRDLSTKFRRIVIIHDTI

VGSIVQDVKLIPNAEAYVLVPTSAFQTSMGITKNLTEKPFQLDPDIPTNIPSNEGCFTSEFLDFAYKQIELLD
FQSGKLFNTCRVIEDRYLELLERLPIYGNMKTFAIGPLNPVQIRRTSEKQRHECLEWLDKQEENSVIYVSF
GSTTAMTDEQITELAKGLEQSGQKFIWVLRKADKGDIFMGDEEGRPQQPEGYEERVKDWGMVVRYWA
PQLEILGHPSTGGFMSHCGWNSCIESISMGVPVVAWPMHSDQPRNATLLTEVLRIGLLIKDCVHLAELVT
SDTIEDAVRRLMTSQEGEEMKKKAEKLGEAVRASVAEGGASRLEMDSFIAHITR

>HU09G01875.1-W

ATGGAAAGCCAGAGTTGCTGCCAAAATGGTATCCAGAAGGGCCAAAAATCTGCTGAAGTAGCTGTGG
TCATGGTGCCCTTCCTAGCCCAAGGCCATCTCAACCAGTTCCTCCATCTCTCTCATCATCTCGTAGCCT
CCTATGGCATACCAGTTCACTACGCTGGGTCTGCCATCCACAACCGCCAAGCCAAGCTACGGTTCCAT
GGTTGGGACCCGGAAACTTCCAACAGAATTGAGTTCCACGACCTACAGCTCCCTCCTTACAGCTCAC
CCTCTCCAAACCCAAATGCAGCTACACATTTCCCTGCGCACCTCCAGCCTCTTTTCGATGCTTCTATGC
AACTCCGCGAACCCCTTTTCCAAGTCTGCCGAGACCTCTCGACCAAGTTCAGGAGAATTGTCATAATT
CATGATACCATAGTAGGTTCAATTGTTCAAGATGTAAAACTCATCCCAAATGCCGAGGCTTACGTGCTT
GTTCCAACCTCGGCTTTTCAAACATCTATGGGCATCACGAAAAATCTTACTGAAAAGCCCTTTCAGCT
TGACCCTGATATCCCAACAAACATTCCCTCCAATGAAGGCTGCTTCACCTCAGAGTTCTTAGACTTCG
CATATAAACAAATAGAGCTCCTGGACTTCCAATCTGGAAAACTGTTCAATACATGCAGGGTTATAGAA
GATAGATATTTGGAGCTACTGGAAAGGCTACCAATTTATGGAAACATGAAAACCTTCGCCATTGGACC
TCTTAATCCAGTGCAAATCAGAAGGACAAGTGAGAAGCAACGACATGAATGTCTAGAATGGCTAGAT
AAACAAGAGGAGAATTCTGTGATATATGTCTCCTTCGGATCGACAACAGCGATGACTGATGAGCAGAT
CACAGAGTTGGCGAAAGGGTTGGAACAAAGCGGGCAGAAGTTTATATGGGTGCTTAGAAAAGCTGAT
AAAGGCGACATTTTTATGGGAGATGAAGAGGGAAGGCCTCAGCAGCCAGAAGGGTATGAAGAGAGG
GTAAAGGACTGGGGAATGGTGGTGAGATACTGGGCACCACAGCTGGAAATTCTCGGTCACCCATCAA
CAGGTGGGTTCATGAGTCACTGTGGGTGGAATTCATGCATAGAGAGTATCAGCATGGGTGTACCAGTT
GTGGCATGGCCGATGCACTCTGACCAGCCTAGGAATGCCACTTTGTTGACAGAAGTGCTAAGAATTGG
ATTATTGATCAAGGATTGCGTGCATCTAGCTGAGCTGGTGACATCAGATACCATAGAGGATGCTGTGAG
ACGACTGATGACATCACAGGAAGGAGAGGAGATGAAGAAAAAGGCAGAAAAACTAGGCGAGGCTG
TTCGAGCTTCAGTCGCTGAAGGTGGTGCTTCTCGCTTGGAAATGGATTCTTTCATTGCTCATATTACCA
GATAA

MESQSCCQNGIQKGQKSAEVAVVMVPFLAQGHLNQFLHLSHHLVASYGIPVHYAGSAIHNRQAKLRFHG
WDPETSNRIEFHDLQLPPYSSPSPNPNAATHFPAHLQPLFDASMQLREPLFQVCRDLSTKFRRIVIIHDTIVG
SIVQDVKLIPNAEAYVLVPTSAFQTSMGITKNLTEKPFQLDPDIPTNIPSNEGCFTSEFLDFAYKQIELLDFQS
GKLFNTCRVIEDRYLELLERLPIYGNMKTFAIGPLNPVQIRRTSEKQRHECLEWLDKQEENSVIYVSFGSTT
AMTDEQITELAKGLEQSGQKFIWVLRKADKGDIFMGDEEGRPQQPEGYEERVKDWGMVVRYWAPQLEI
LGHPSTGGFMSHCGWNSCIESISMGVPVVAWPMHSDQPRNATLLTEVLRIGLLIKDCVHLAELVTSDTIED
AVRRLMTSQEGEEMKKKAEKLGEAVRASVAEGGASRLEMDSFIAHITR

UGT79B6

>HU03G02206.1-R

ATGGACCTGACCCACCCTGAAGTTGAGCTGATTATAATGAGCCTGGAGTCCAAGCCAAACCTCCTCTT
CTACGACGTGGCTCATTGGATCCCCGAGATAGCCTCCAAGCTGTCTGGGGTCCAAACCGTCTGCTACA
AAGTCGTCTGTGCTGCCTCCCTCGCCATCGCCATCGTCCCAGCCCGGGGTCTGAGCCGGGACAAAAC
CGTCACCGACTCGGACCTGGCCGAACTCCCCCGAGGTTACCCCTCAAACAACGTTGTTTTACGGGGA
GCAGAGTGGCAGAACCTCCTCTTTATTGGGCTGGAGTTTGGCTCCGGCCTCACCTTCTATGAGCGGAT
TACAAGGGCAATACGCTCCTGCGATGCGATAGCATTCCGAACGTGCAGAGAAATCGAAGGGAGCTTC
TGTGACTACATGGCAAGCCAATACAACAAGCCGGTCTTTCTTACCGGTCCTGTTTTAGCAGAAGTGGA
TAGTACTACAACAACGCCACTGGACCAGAAATGGGCCAATTGGCTAAACCAGTTTGGGTCCCGTTCG
GTGGTGTTTTGCGCATTCGGGAGCCAGTTTGCACTCGAGAAGGAGCAGCTGAAAGAGCTACTGCTTG
GGTTTGAGATGGCAGGGAAGCCATTCTTCATGGCTGTGAAGCCCCCTTTAGAGTGCGCCTCAATCGGG
GAGGCACTGCCAAAGGGGTTCGAGGAGCGGGTCCGAGCGAGAGGAGTGGTCTGCGGGTCATGGGTA
CAGCAACCACAGATTCTTGCGCACCCGTCGGGCGGTTGCTTCGTGAACCACTGTGGGTTCGGTTCGAT
GTGGGAGTCGTTAATGAGTGAGAGTCAAATAGTGTTGGTGCCGCAGCTTGGGGACCAGATTTTGAAC
GCTAGGATAATTGCTGGGGAGATGAAGGTGGCTGTGGAGGTGGAGAGGGGAGAGAATGGATGGGTCT
CTAAGGAGAATCTATGCAAAGCTATAAATTCAGTGATGGATGAAGGGAGTGAGGTGGGGGAGATGGT
TAAGAAGAATCATGCAAAGTGGAGAGAGGTTTTTGTTAGGAATGACTTTATGGGTGGTTACATTGATA
ATTTTGTTAAGGATTTGGAAATGCTTGTTGGTGGGCATTAG

MDLTHPEVELIIMSLESKPNLLFYDVAHWIPEIASKLSGVQTVCYKVVCAASLAIAIVPARGLSRDKTVTD
SDLAELPRGYPSNNVVLRGAEWQNLLFIGLEFGSGLTFYERITRAIRSCDAIAFRTCREIEGSFCDYMASQY
NKPVFLTGPVLAEVDSTTTTPLDQKWANWLNQFGSRSVVFCAFGSQFALEKEQLKELLLGFEMAGKPFF
MAVKPPLECASIGEALPKGFEERVRARGVVCGSWVQQPQILAHPSGGCFVNHCGFGSMWESLMSESQIV
LVPQLGDQILNARIIAGEMKVAVEVERGENGWVSKENLCKAINSVMDEGSEVGEMVKKNHAKWREVFV
RNDFMGGYIDNFVKDLEMLVGGH

>HU03G02206.1-W

ATGGACCTGACCCGCCCTGAAGTTGAGCTGATTATAATGAGCCTGGAGTCCAAGCCAAACCTCCTCTT
CTACGACATGGCCCATTGGATCCCCCAGATAGCCTCTAAGCTGTCCAGGGTCCAAACCGTTTGCTACA
AAGTCGTCTGTGCTGCCTCCCTCGCCATCGCCGTCGTCCCAGCTCGGGGTCTGACACGGGACAAACC
CGTCACTGAGTCGGACCTTGCCCAGCTCCCCCAAGGCTATCCCTCAAACAACGTCGTTTTACGGGGA
GCAGAGTGGCAGAACCTCCTCTTCATTGGGCTGGAGTTTGGATCCGGGCTCACCTTCTGTGAGCGGAT
TACAAGGGCTATACGCTCCTGCGATGCGATAGCATTACGAACGTGCAGAGAAATCGAAGGGGGCTTCT
GTGACTACATGGCAAGCCAATACAACAAGCCGGTCTTTCTTACCGGTCCTGTTTTAGCAGAAGTGGAT
AGTACTACAACAACGCCACTGGACCAGAAATGGGCGAATTGGCTAAACCAGTTTGGGTCTCGTTCGG
TGGTGTTTTGCGCGTTCGGGAGCCAGTTTGTACTCGAGAAGGAGCAGCTGAAAGAGCTACTGCTTGG
GTTTGAAATGGCAGGGAAACCATTCTTCGTGGCTGTGAAGCCCCCTTTAGAGTGCGCCTCAATCGGG

GAGGCACTGCCAAAAGGGTTCGAGGAGCGGGTCCGAGCGAGAGGAGTGGTCTGCGGGTCATGGGTA
CAGCAACCACAGATTCTTGCGCACCCGTCGGTCGGGTGCTTCGTGAACCACTGTGGGTTCGGTTCGAT
GTGGGAGTCGTTAATGAGTGAGAGTCAGATAGTGTTGGTGCCGCAGCTTGGGGACCAGATTTTGAAC
GCTAGGATAATTGCTGGGGAGATGAAGGTGGCTGTGGAGGTGGAGAGGGGAGAGAATGGATGGGTCT
CCAAGGAGAATCTGTGCAAAGCTATAAATTCAGTGATGGATGAAGGGAGTGAGGTGGGGGAGATGGT
TAAGAAGAATCATGCAAAGTGGAGAGAGGTTTTTGTTAGGAATGACTTTATGGGTGGTTACATTGATA
ATTTTGTTAAGGATTTGGAAATGCTTGTTGGAGGGCATTAG

MDLTRPEVELIIMSLESKPNLLFYDMAHWIPQIASKLSRVQTVCYKVVCAASLAIAVVPARGLTRDKPVTE
SDLAQLPQGYPSNNVVLRGAEWQNLLFIGLEFGSGLTFCERITRAIRSCDAIALRTCREIEGGFCDYMASQ
YNKPVFLTGPVLAEVDSTTTTPLDQKWANWLNQFGSRSVVFCAFGSQFVLEKEQLKELLLGFEMAGKPF
FVAVKPPLECASIGEALPKGFEERVRARGVVCGSWVQQPQILAHPSVGCFVNHCGFGSMWESLMSESQIV
LVPQLGDQILNARIIAGEMKVAVEVERGENGWVSKENLCKAINSVMDEGSEVGEMVKKNHAKWREVFV
RNDFMGGYIDNFVKDLEMLVGGH

>HU07G01550.1-R

ATGGGTAAACAGCTCCACATAGTGATGTTCCCATGGTTTGCGATGGGACACATGATCCCATTCCTACAA
CTTGCGAACAAGCTTGCAGAAAGAGGTCACAAAACAACCTTCCTACTACCCAAGAAAGCCATCCTAC
ATTTAGCCTCCTTAAACCTCCATACATCCCTCATCAGCCTCCACCCAATCACTGTCCCTCACGTGGACC
CTCTCCCTCCTGGCATTGAGACTGCCTCCGACATTCCCATCCACCTCAACAACCACCTCGCCACAGCC
ATGGACCTGACCCGCCCAGAAGTTGAGCTGATTATAATGAGCCTGGAGTCCAAGCCAAACCTCCTCTT
CTACGATGTGGCTCATTGGATCCCCGAGATAGCCTCCAAGCTGTCTGGGGTCCAAACCGTCTGCTACA
AAGTCGTCTGTGCTGCCTCCCTCGCCATCGCCATCGTCCCAGCCCGGGGTCTGAGCCGGGACAAAAC
CGTCACCGACTCGGACCTGGCCGAACTCCCCCGAGGTTACCCCTCAAACAACGTTGTTTACGGGGA
GCAGAGTGGCAGAACCTCCTCTTTATTGGGCTGGAGTTTGGCTCCGGCCTCACCTTCTATGAGCGGAT
TACAAGGGCAATACGCTCCTGCGATGCGATAGCATTCCGAACGTGCAGAGAAATCGAAGGGAGCTTC
TGTGACTACATGGCAAGCCAATACAACAAGCCGGTTTTTCTTACCGGTCCTGTTTTGCCTGAACTGGA
TAGTACTACAACAACGCCACTTGACCAGAAATGGGCCGATTGGCTGGATCAGTTCACGTCCCGTTCGG
TGGTGTTCTGCGCGTTCGGGAGCCAGTTTGTACTGGAGAAGGAGCAGCTGAAAGAGCTACTGCTTGG
GTTTGAGATGGCAGGGAAGCCATTCTTCGTGGCTGTGAAACCCCCTTCAGGGTGCGCCTCAATCGGG
GAGGCACTGCCAGAAGGGTTCGAGGAGCAGGTCCGAGAGAGAGGAGTGGTCTGCGGGTCATGGGTA
CAGCAACCACAGATTCTTGCGCACCCGTCGGTCGGGTGCTTCGTGAGCCACTGTGGGTTCGGTTCGAT
GTGGGAGTCGTTGATGAGTGAGAGTCAGATAGTGTTGGTGCCGCAGCTTGGGGACCAGATTTTGAAT
GCCAGGATAATTGCTGGGGAGATGAAGGTGGCTGTGGAGGTGGAGAGGGAAGAGAACGGATGGGTC
TCCAAGGAGAATCTGTGCAAAGCTATAAAGTCAGTGATGGATGAAGGGAGTGAGGTGGGGGAGATGG
TTAAGAAGAATCATGCAAAGTGGAGAGGGGTTTTTGTTAGGAATGACTTTATGGGTGGTTACATTGAT
AATTTTGTTAAGGATTTGGAAATGCTTGTTGGTGGGCATTAG

MGKQLHIVMFPWFAMGHMIPFLQLANKLAERGHKTTFLLPKKAILHLASLNLHTSLISLHPITVPHVDPLP

PGIETASDIPIHLNNHLATAMDLTRPEVELIIMSLESKPNLLFYDVAHWIPEIASKLSGVQTVCYKVVCAAS
LAIAIVPARGLSRDKTVTDSDLAELPRGYPSNNVVLRGAEWQNLLFIGLEFGSGLTFYERITRAIRSCDAIA
FRTCREIEGSFCDYMASQYNKPVFLTGPVLPELDSTTTTPLDQKWADWLDQFTSRSVVFCAFGSQFVLEK
EQLKELLLGFEMAGKPFFVAVKPPSGCASIGEALPEGFEEQVRERGVVCGSWVQQPQILAHPSVGCFVSH
CGFGSMWESLMSESQIVLVPQLGDQILNARIIAGEMKVAVEVEREENGWVSKENLCKAIKSVMDEGSEV
GEMVKKNHAKWRGVFVRNDFMGGYIDNFVKDLEMLVGGH

>HU07G01550.1-W

ATGGGTAAACAGCTCCACATAGTGATGTTCCCATGGTTTGCGATGGGACACATGATCCCATTCCTACAA
CTTGCGAACAAGCTTGCAGAAAGAGGTCACAAAACAACCTTCCTACTACCCAAGAAAGCCATCCTAC
ATTTAGCCTCCTTAAACCTCCATACATCCCTCATCAGCCTCCACCCAATCACTGTCCCTCACGTGGACC
CTCTCCCTCCTGGCATTGAGACTGCCTCCGACATTCCCATCCACCTCAACAACCACCTCGCCACAGCC
ATGGACCTGACCCGCCCAGAAGTTGAGCTGATTATAATGAGCCTGGAGTCCAAGCCAAACCTCCTCTT
CTACGATGTGGCTCATTGGATCCCCGAGATAGCCTCCAAGCTGTCTGGGGTCCAAACCGTCTGCTACA
AAGTCGTCTGTGCTGCCTCCCTCGCCATCGCCATCGTCCCAGCCCGGGGTCTGAGCCGGGACAAAAC
CGTCACCGACTCGGACCTGGCCGAACTCCCCCGAGGTTACCCCTCAAACAACGTTGTTTACGGGGA
GCAGAGTGGCAGAACCTCCTCTTTATTGGGCTGGAGTTTGGCTCCGGCCTCACCTTCTATGAGCGGAT
TACAAGGGCAATACGCTCCTGCGATGCGATAGCATTCCGAACGTGCAGAGAAATCGAAGGGAGCTTC
TGTGACTACATGGCAAGCCAATACAACAAGCCGGTTTTTCTTACCGGTCCTGTTTTGCCTGAACTGGA
TAGTACTACAACAACGCCACTTGACCAGAAATGGGCCGATTGGCTGGATCAGTTCACGTCCCGTTCGG
TGGTGTTCTGCGCGTTCGGGAGCCAGTTTGTACTGGAGAAGGAGCAGCTGAAAGAGCTACTGCTTGG
GTTTGAGATGGCAGGGAAGCCATTCTTCGTGGCTGTGAAACCCCCTTCAGGGTGCGCCTCAATCGGG
GAGGCACTGCCAGAAGGGTTCGAGGAGCAGGTCCGAGAGAGAGGAGTGGTCTGCGGGTCATGGGTA
CAGCAACCACAGATTCTTGCGCACCCGTCGGTCGGGTGCTTCGTGAGCCACTGTGGGTTCGGTTCGAT
GTGGGAGTCGTTGATGAGTGAGAGTCAGATAGTGTTGGTGCCGCAGCTTGGGGACCAGATTTTGAAT
GCCAGGATAATTGCTGGGGAGATGAAGGTGGCTGTGGAGGTGGAGAGGGAAGAGAACGGATGGGTC
TCCAAGGAGAATCTGTGCAAAGCTATAAAGTCAGTGATGGATGAAGGGAGTGAGGTGGGGGAGATGG
TTAAGAAGAATCATGCAAAGTGGAGAGGGGTTTTTGTTAGGAATGACTTTATGGGTGGTTACATTGAT
AATTTTGTTAAGGATTTGGAAATGCTTGTTGGTGGGCATTAG

MGKQLHIVMFPWFAMGHMIPFLQLANKLAERGHKTTFLLPKKAILHLASLNLHTSLISLHPITVPHVDPLP
PGIETASDIPIHLNNHLATAMDLTRPEVELIIMSLESKPNLLFYDVAHWIPEIASKLSGVQTVCYKVVCAAS
LAIAIVPARGLSRDKTVTDSDLAELPRGYPSNNVVLRGAEWQNLLFIGLEFGSGLTFYERITRAIRSCDAIA
FRTCREIEGSFCDYMASQYNKPVFLTGPVLPELDSTTTTPLDQKWADWLDQFTSRSVVFCAFGSQFVLEK
EQLKELLLGFEMAGKPFFVAVKPPSGCASIGEALPEGFEEQVRERGVVCGSWVQQPQILAHPSVGCFVSH
CGFGSMWESLMSESQIVLVPQLGDQILNARIIAGEMKVAVEVEREENGWVSKENLCKAIKSVMDEGSEV
GEMVKKNHAKWRGVFVRNDFMGGYIDNFVKDLEMLVGGH

>HU09G01034.1-R

ATGGGGGAACAACTCCACATCGTGATGTACCCGTGGCTGCATTTGGGTCACATAATCCCATATCTTCAC
TTCGCAAACAAGCTTGCTGAGAGATCAGGCCACAAAATTAGGACCACCCTCTTACTCCCCAAGAAGG
CGGTCCCCCAACTCAACCACATGAATCTCCACCCTTCTCTGATCTCCCTCCACCCAATCACCATCCCCC
ATGTGGACCCCCTCCCTCTCGGCTCCGAGACCGCCTCCGATGTTCCCCCCCACCTGGCCCCTCACCTC
GCCACCGCCATGGACCTGACTAGACCCGAAGTCGAGTCCACGATATTGGGCCTCCAGCCCAGGGCCA
CCCTCATCTTTTACGATCTAGCCTATTGGGTCAACGAAATAGCATCCGAGCTCGGAATCAAGACCGTCT
GCTACGTCCCCGTTTGCGCGGCTTGTCAAGCCATGGGTATTGCTATGCGACCAATCCAAGACGAGGCC
GTACCAGAGCTCGCCCCGGAGTACCCCTCAACCGACGTCGTCTTAAGGGGGCCGGCCGAGTGGCGGA
AGATATCATTGAGAACGTCACAGTTCGGCAGCGGGCTCAAGTTTAGTGAGCGGACCGGACGGTCCAA
GACATTGTGTGACGCCATTGGCCTCCGAACTTGCAGAGAGATTGAAGGGGCTTTCTGCGACTACGTAA
GTAGCCACTTTGGCAAGCCCCTTCTCCTCTCGGGCCCAGTCCTGCCCGAGCCTAATACGGCCACGCCG
CTCGACCCCGAATGGGCCTCGTGGCTGGGCCGATTCGGGCTTGGCTCGGTGGTGTTCTGCACCTTAGG
GAGTCAGATGACCCTGGAGAGGGAGCAGTTCCAGGAACTGCTCCTGGGTTTTGAAATGACCAAAATG
CCCTTTCTCGTCGTCCTGAAGCCTCCAACTGGGTGCTCCTCCATCGATGAGGCCCTCCCGGAGGGGTT
CCAGGAGAGGGTGGGAGACAGAGGGGTCGTTCATGGCGGGTGGGTGCAACAACCACAGATACTGTC
GCACCCATCGGTGGGTTGCTTCGTGAGCCATTGTGGGCTCGGGGCGCTGTGGGAGTCGCTCATGTCTG
AGTGTCAGATAGTGCTCCTACCGCAGGCTTGGGACCATATTGTGAATGCTAGGATTATGGCAGCCCAG
ATGAAGGTGGCTGTGGAGGTAGAGAAGAGGGAAGAGGATGGGTGGGTTTCAAAGGAGAGTTTGTGT
AAAGCCATCAAAGCCGTCATGGATGACGAGAGTGAGGTCGGGTGCTTGGTCAAGAAGAACCACGCA
AAATGGAGAGAGGTTATCACTCGGCAAGGGTTTATGGATGGCTATGTTGATAATTTCATCAAGGATTTA
GAAAGACTTATTTCTACTGCTTGA

MGEQLHIVMYPWLHLGHIIPYLHFANKLAERSGHKIRTTLLLPKKAVPQLNHMNLHPSLISLHPITIPHVDP
LPLGSETASDVPPHLAPHLATAMDLTRPEVESTILGLQPRATLIFYDLAYWVNEIASELGIKTVCYVPVCAA
CQAMGIAMRPIQDEAVPELAPEYPSTDVVLRGPAEWRKISLRTSQFGSGLKFSERTGRSKTLCDAIGLRTC
REIEGAFCDYVSSHFGKPLLLSGPVLPEPNTATPLDPEWASWLGRFGLGSVVFCTLGSQMTLEREQFQELL
LGFEMTKMPFLVVLKPPTGCSSIDEALPEGFQERVGDRGVVHGGWVQQPQILSHPSVGCFVSHCGLGAL
WESLMSECQIVLLPQAWDHIVNARIMAAQMKVAVEVEKREEDGWVSKESLCKAIKAVMDDESEVGCLV
KKNHAKWREVITRQGFMDGYVDNFIKDLERLISTA

>HU09G01034.1-W

ATGGGGGAACAACTCCACATCGTGATGTACCCGTGGCTGCATTTGGGTCACATAATCCCATATCTTCAC
TTCGCAAACAAGCTTGCCGAGAGATCAGGCCACAAAATTAGGACCACCCTCTTACTCCCCAAGAAGG
CGGTCCCCCAACTCAACCACATGAATCTCCACCCTTCTCTGATCTCCCTCCACCCAATCACCATCCCCC
ATGTGGACCCCCTCCCTCTCGGCTCCGAGACCGCCTCCGATGTTCCCCCCCACCTGGCCCCTCACCTC
GCCACCGCCATGGACCTGACTCGGCCTGAAGTCGAGTCCACGATATTGGGCCTCCAGCCCAGGGCCA
CCCTCATCTTTTACGACATGGCCTATTGGGTCAACGAAATAGCGTCCGAGCTCGGAATCAAGACCGTC
TGCTACATGCCCGTTTGCGCGGCTTGTCAAGCCATGGCTATTGCTATGCGACCAACCCAAGACGAGGC
CGTACCAGAGCTCGCCCCGGAGTACCCTTCAACCGACGTCGTCTTACGGGGGCCGGCCGAGTGGCGG

AAGATATCATTGAGAAAGTCACAGTTCGGCAGCGGGCTCAAGTTTAGTGAGCGGACCAGACGGTCCA
AGACATTGTGTGACGCCATTGGCCTCCGAACGTGTAGAGAGATTGAAGGGGCTTTCTGCGACTACGTA
AGTAGCCACTTTGGCAAGCCCCTTCTCCTCTCGGGCCCAGTCCTGCCCGAGCCTAATACGGCCACGCC
GCTCGACCCCGAATGGGCCGCGTGGCTGGGCCGATTCGGGCCTGGCTCGGTGGTGTTCTGCACCTTA
GGGAGTCAGATGACCCTGGAGAGAGAGCAGTTCCAGGAACTGCTCCTGGGTTTTGAAATGACCAAA
ATACCCTTTCTCGTCGTCCTGAAGCCTCCAACTGGGTGCTCCTCCATCGATGAGGCCCTCCCGGAGGG
GTTCCAGGAGAGGGTGGGAGACAGAGGGGTCGTTCATGGCGGGTGGGTGCAACAACCACAGATACT
GTCGCACCCATCGGTGGGTTGCTTCGTGAGCCATTGTGGGCTCGGGGCGCTGTGGGAGTCGCTCATGT
CTGAGTGTCAGATAGTGCTCCTACCGCAGGCTTGGGACCATATTATGAATGCTAGGATTATGGCAGCCC
AGATGAAGGTGGCTGTGGAGGTAGAGAAGAGGGAAGAGGATGGGTGGGTTTCAAAGGAGAGTTTTT
GTAAAGCCATCAAAGCCGTCATGGATGAAGAGAGTGAGGTGGGGTGCTTGGTCAAGAAGAACCACG
CAAAATGGAGAGAGGTTATCACTCGGCAAGGGTTTATGGATGGCTATGTTGATAATTTCATCAAGGATT
TAGAAAGACTTGTTACTGATGCTTGA

MGEQLHIVMYPWLHLGHIIPYLHFANKLAERSGHKIRTTLLLPKKAVPQLNHMNLHPSLISLHPITIPHVDP
LPLGSETASDVPPHLAPHLATAMDLTRPEVESTILGLQPRATLIFYDMAYWVNEIASELGIKTVCYMPVCA
ACQAMAIAMRPTQDEAVPELAPEYPSTDVVLRGPAEWRKISLRKSQFGSGLKFSERTRRSKTLCDAIGLRT
CREIEGAFCDYVSSHFGKPLLLSGPVLPEPNTATPLDPEWAAWLGRFGPGSVVFCTLGSQMTLEREQFQEL
LLGFEMTKIPFLVVLKPPTGCSSIDEALPEGFQERVGDRGVVHGGWVQQPQILSHPSVGCFVSHCGLGAL
WESLMSECQIVLLPQAWDHIMNARIMAAQMKVAVEVEKREEDGWVSKESFCKAIKAVMDEESEVGCLV
KKNHAKWREVITRQGFMDGYVDNFIKDLERLVTDA

UGT79B30

>HU04G00198.1-R

ATGGCCCAGGCTAAAGAAAAGCTTAATTTGCACATAGCCATGCACCCATGGTTCGCCATGAGCCATATT
ACTAATTTTCTAGGGCTAGCCAACAAGTTCGCTGAGAAGGGTCACAAAATAAGCTTCTTCCTACCACC
CAAAACCCAGCCCAAGCTAGCCTCCTTCAACCACCATCCCCACCTCATCACCTTCATCCCGGTCCCAG
TTCCCCGGGTCGACGGGCTGCCCGACGGGGCCGAGACCACCCACGACGTGCCAGCCCTGGCCCGGC
CCCTCCTCATGGCCGCCATGGACCTGACCCGGGACGTCATCGACTCCCACCTGGCCCGACTCAGGCC
CGACGTAGTCTTCTTCGACTTCGCCGAGTGGCTACCCGGGCTGGCCAGAAAACACGGGGCCAAGTCC
GTTCGCTTTGCCACCACGTTCCTGTTTACGGCTGCTTACCTCCTCGCCGGGCCGAGGAATTTGCCCGA
TGGCTGCCCCCCTAAGGAGGAGGACCTCATGCACCCACCACCGGGCTTCCCAGCCCAAGGTATCGGG
CTGGCGGCCCATGAGGCCCGGGTCATAGTCAAGGCAATAAGAATGGAATTTGGTGGAGGGATGAATTT
GTTCGAGAGGTCAAAAATAGCTTTTGAGGAATCTGATGCTATGGGGTTCAAGACTTGTAGGGAGATGG
AAGGTCCCTTTGTTGAGTTTGTTGCAAAGCATTCGGACAAGCGTTTGCTCCTGGCTGGGCCAATGCTC
CCGAGCCCCCCCGATTCGAGGCTCGACGAGGACATCGATGCGTGGCTCAAGGGGTTCGGTCATGGTG
GCGTGGTCTACTGCGCCCTTGGGAGTGAATCGGATCTTCAGCAGGACCAGTTTCAAGAGCTTGCTCTT

GGGCTTGAGCTAACAGGTCGGCCATTCTTGGCAGCCCTCAAGCCCCCAAGAAATCACCAAACCATAG
AGTCAGCATTGCCAAAGGGGTTCTTAGAGAGAACAAAAGATCGAGGCATGATTCATGGGGGATGGGT
GCAACAACAACTGATCCTGCACCATCCTTCAGTAGGGTGTTTCGTGACACATTGTGGAGCCTCGTCCT
TGTTCGAAGGCATGTCGAGTGAGTGCCAACTAGTCATGTTGCCCCAACAAATTGACCAGTTCACCAAT
GCAAGGGTGATGAGCTCATACTTTAAAGTTGGAGTTGAGGTTGAGAAGAGAGACCATGATGGCTTCT
TCACTAGAGAGGCAATTTGCAAAGCAGTTAGTTTGGTGATGGAGGAAGAAAGCGAGGTGGGTAAAG
AAGTGAGAGCCAACCATGCTAAATGGAGGGAGATCTTGTCCAAGGAGGGCCTTGAAGATTCTTATATT
ACCGAACTTGTTCGGGATTTACAAGGTTTACTTGCA

MAQAKEKLNLHIAMHPWFAMSHITNFLGLANKFAEKGHKISFFLPPKTQPKLASFNHHPHLITFIPVPVPR
VDGLPDGAETTHDVPALARPLLMAAMDLTRDVIDSHLARLRPDVVFFDFAEWLPGLARKHGAKSVRFAT
TFLFTAAYLLAGPRNLPDGCPPKEEDLMHPPPGFPAQGIGLAAHEARVIVKAIRMEFGGGMNLFERSKIAF
EESDAMGFKTCREMEGPFVEFVAKHSDKRLLLAGPMLPSPPDSRLDEDIDAWLKGFGHGGVVYCALGSE
SDLQQDQFQELALGLELTGRPFLAALKPPRNHQTIESALPKGFLERTKDRGMIHGGWVQQQLILHHPSVG
CFVTHCGASSLFEGMSSECQLVMLPQQIDQFTNARVMSSYFKVGVEVEKRDHDGFFTREAICKAVSLVME
EESEVGKEVRANHAKWREILSKEGLEDSYITELVRDLQGLLA

>HU04G00198.1-W

ATGGCCCAGGCTAAAGAAAAGCTTAATTTGCACATAGCCATGCACCCATGGTTCGCCATGAGCCATATT
ACTAGTTTTCTAGGGCTAGCCAACAAGTTCGCTGAGAAGGGTCACAAAATAAGCTTCTTCCTACCACC
CAAAACCCAGCCCAAGCTAGCCTCCTTCAACCACCATCCCCACCTCATCACCTTCATCCCGGTCCCAG
TTCCCCGGGTCGACGGGCTGCCCGACGGGGCCGAGACCACCCACGACGTGCCAGCCCTGGCCCGGC
CCCTCCTCATGGCCGCCATGGACCTGACCCGGGACGTCATCGACTCCCACCTGGCCCGACTCAAGCC
CGACGTAGTCTTCTTCGACTTCGCCGAGTGGCTACCCGGGCTGGCCAGAAAACACGGGGCCAAGTCC
GTTCGCTTTGCCACCACGTTCCTGTTTACGGCTGCTTACCTCCTCGCCGGGCCGAGGAATTTGCCCGA
TGGCTGCCCCCCTAAGGAGGAGGACCTCATGCACCCACCACCGGGCTTCCCAGCCCAAGGTATCGGG
CTGGCGGCCCATGAGGCCCGGGTCATAGTCAAGGCAATAAGCATGGAATTTGGTGGAGGGATGAATTT
GTTCGAGAGGTCAAAAATAGCTTTTGAGGAATCTGATGCTATGGGGTTCAAGACTTGTAGGGAGATGG
AAGGTCCCTTTGTTGAGTTTGTTGCAAAGCATTCGGACAAGCGTTTGCTCCTGGCTGGGCCAATGCTC
CCGAGCCCCCCCGATTCGAGGCTCAACGAGGACATCGATGCGTGGCTCAAGGGGTTCGGTCATGGTG
GCGTGGTCTACTGCGCCCTTGGGAGTGAATCGGATCTTCAGCAGGACCAGTTTCAAGAGCTTGCTCTT
GGGCTTGAGCTAACAGGTCGGCCATTCTTGGCAGCCCTCAAGCCCCCAAGAAATCACCAAACAATAG
AGTCAGCATTGCCAAAGGGGTTCTTAGAGAGAACAAAAGATCGAGGCATGATTCATGGGGGATGGGT
GCAACAACAACTGATCCTGCACCATCCTTCAGTAGGGTGTTTCGTGACACATTGTGGAGCCTCGTCCT
TGTTCGAAGGCATGTCGAGTGAGTGCCAACTAGTCATGTTGCCCCAACAAATTGACCAGTTCACCAAT
GCAAGGGTGATGAGCTCATACTTTAAAGTTGGAGTTGAGGTTGAGAAGAGAGACCATGATGGCTTCT
TCACTAGAGAGGCAATTTCCAAAGCAGTTAGTTTGGTGATGGAGGAAGAAAGCGAGGTGGGTAAAG
AAGTGAGAGCCAACCATGCTAAATGGAGGGAGATCTTGTCCAAGGAGGGCCTTGAAGATTCTTATATT
ACCGAACTTGTTCGGGATTTACAAGCCTCCCTGAACCACCACCCCCACCGCATCACCTTTATCCCGGT

CCCGGTCCCCCGGGTCGACGGCCTACCGGAATGGGCCGAGACCACGAATGATGTGCCCCCTGAAGCC
CGACCACTCATCATGATCGCCATGGACTTGACCCGGGACACCATTGACTCTCATCTGGCCCAACTCAG
GCCCGAACTCATCATCTTTGACTTCGCGCCTTGGGTACCTGGGCTGGCCCGAAAGTACCGGGCCAAGT
CCGTTTTCTATTGCGCGTCGTTCTTGATGCCTGCCGTTTACTTCTTCCCCCAGGCCCGGAACAAGCCCA
TCAACTATGCCCTCACGGAGGAGGATCTAGTCCACCCGCCTCCGGGTTTTCCTGACGGAATCCGGCTT
GCGGCCCACGAGGCCCGGGCCATGGCCTATGCATTGAAGTTGGATTTCGGAGGAGGGATGACATGGG
CCCAAAGGTCCATGATGGCCTATAAAGAATGTGATGCTATTGCCATCAAGACTTGTAGGGAGATGGAA
GGTCCCTTGTGTGAGTTTGCCGCAATGCTCGTTAACAAGCCAGTGTTCCTAGCCGGGCCATTGCTTCC
GGGCCATCCGACTTCCCGCCTCGATGAGGACATAGATGGGTGGTTGAAGGGGTTCGGTCGTGGCACC
GTGATCTACTGTGCCCTTGGGAGTGAATGCACTCTTGAAAAGGACCAGTTTCAGGAGCTTGTTCTTGG
ACTTGAGCTAACAGGTAAGCCATTTTTGGCAGCCCTGAAGCCCCCAATAAATTGCAAAACAATCGAGT
CAGCATTGCCAGAAGGGTTCCTAGAAAGAACAAGAGACAGAGGTATGATTCACGACGGATGGGTGCA
ACAACAGTTAATCTTGAACCACCCCTCAGTAGGGTGTTTCGTGACACATAGTGGATCTGGGTCTTTTT
CGGAGGCCATAATAAGCAAGTGTCAAATGGTCTTGCTTCCTCAAGCAGTCGACCAGTTCCTCGTCGCG
AGGTTGATGAGTTGCTACATGAAAATTGGAGTTGAGGTCGAGAAGAGAGACTATGATGGCTTCTTCAC
TAGAGAGGCCGTTCGCAAAGCGATCAATTTAGTGATGGAGGAAGAGAGCGAGGTGGGTCAAGAAGT
GAGAGCCAACCATGCTAAATGGAGGGAGCTCTTGTCTAAGGAAGGCCTTGAAGAATACTATATTTCCC
AACTTGTTCAAGGTTTACATGGTTTACTTGGACCTAAGTAA

MAQAKEKLNLHIAMHPWFAMSHITSFLGLANKFAEKGHKISFFLPPKTQPKLASFNHHPHLITFIPVPVPR
VDGLPDGAETTHDVPALARPLLMAAMDLTRDVIDSHLARLKPDVVFFDFAEWLPGLARKHGAKSVRFAT
TFLFTAAYLLAGPRNLPDGCPPKEEDLMHPPPGFPAQGIGLAAHEARVIVKAISMEFGGGMNLFERSKIAF
EESDAMGFKTCREMEGPFVEFVAKHSDKRLLLAGPMLPSPPDSRLNEDIDAWLKGFGHGGVVYCALGSE
SDLQQDQFQELALGLELTGRPFLAALKPPRNHQTIESALPKGFLERTKDRGMIHGGWVQQQLILHHPSVG
CFVTHCGASSLFEGMSSECQLVMLPQQIDQFTNARVMSSYFKVGVEVEKRDHDGFFTREAISKAVSLVME
EESEVGKEVRANHAKWREILSKEGLEDSYITELVRDLQASLNHHPHRITFIPVPVPRVDGLPEWAETTNDV
PPEARPLIMIAMDLTRDTIDSHLAQLRPELIIFDFAPWVPGLARKYRAKSVFYCASFLMPAVYFFPQARNKP
INYALTEEDLVHPPPGFPDGIRLAAHEARAMAYALKLDFGGGMTWAQRSMMAYKECDAIAIKTCREMEG
PLCEFAAMLVNKPVFLAGPLLPGHPTSRLDEDIDGWLKGFGRGTVIYCALGSECTLEKDQFQELVLGLELT
GKPFLAALKPPINCKTIESALPEGFLERTRDRGMIHDGWVQQQLILNHPSVGCFVTHSGSGSFSEAIISKCQ
MVLLPQAVDQFLVARLMSCYMKIGVEVEKRDYDGFFTREAVRKAINLVMEEESEVGQEVRANHAKWRE
LLSKEGLEEYYISQLVQGLHGLLGPK

>HU10G01599.1-R

ATGTCAGAAAACAATCACAAACCTTTGCACATAGCCATGTTCCCATGGTTTGCTTTCGGCCACATTACA
TCTTTTCTCCACTTAGCCAACAAACTAGCTGAACAAGGCCACAGAATCTCCTTCATCCTCCCTCCCAA
AACTCAAGCCCAAATGCTCTCTCATAACCACCACCCTCATCTCATTTCGTTCATCCCCATCACTCTCCC
TCCTGTTGATGGGCTCCCCACTTGGGCCGAGACTACGGCCGATGTACCCGCCTCATCCCGGCCCCTTAT
CATGACCGCCATGGACCGGACCCAAGACATGATCGAGTCCCTGCTCTCGAGCCTTAATCTTGATTTCAT

TTTCTTCGACTTCACTGAGTGGGTACCGGGCCTGGCCAGAAAATACCGAGTCAATTCCGTGTTCTATG
CCACCGTGTACGTGGTAACAGTTGCTTACTTGACCCCTCAGGCAAGAAACCTCCCTGTCAATGGCCTT
ATGGAGGAGAATTTGGCGGGTCCGCCACCTGGTTTTCCATGCCCATCTATAGGGCTCAGGGCCCATGA
GGCCCAATACATGGCGAATGCATCAGGGGTGAAATTTGGAGGGGAAATATCACTGCTCGAAAGGGCT
GCAATCGCTTTCCGAGAGTGTGATGCCATGGGGATCAAGAGTTGTAGGGAAATGGAAGGGCCATATTG
TGATTATGTTATTGAGCACTATGGCAAGCCTTTGCTTTTGGCTGGGCCAGTGGTGCCCAAACTGCCGG
ATTCTAAGCTAGATGAGCAGCTTGAGGCCTGGTTGAAGAGTTTTAGTCAAGGATGTATTGTGTATTGTG
CACTTGGGAGTGAATGCTTTCTCAAAAAGGACCAGTTCCAAGAGCTAGTTTTGGGACTTGAGCTCAC
AGGAATGCCCTTCCTGGCAGCTTTAAAGCTACCTATAGATTGTCCTACAATGGAGTCAGCATTACCCTA
TGGCTTCACGGAGCGAACCAAAGGAAGGGCGATCATCCACGAGGGGTGGGTGCAGCAGCAGCTGAT
CTTGAACCACCCTTCAGTTGGGTGCTTCGTGACTCACTGTGGAGCTGGATCCTTGTCAGAAGGTCTAG
TAAGCCACTGCCAACTGGTGATGCTTCCTCAGGCAGTTGATCAGTTTCTGAACGCCAGGATGATGAGC
CAGGACCTCAAGGTTGGCGTGGAGGTCGAGGAGAGGGAAGGTGGTTTGATTACTCGGGAGGCAGTT
CGCGATGCGATACTGTTGGTGATGCAGGAGGGAAGTGCGATGGGGAGGGAGGTGCGGGAAAACCAT
GCTAAGATGAGAGAGTTCTTGGTCAAAGGAGGGGTTGAGGACTCTTATATTAGTAGCTTTGTTCAGAG
TCTGTATGACCTGAGGGGTTGA

MSENNHKPLHIAMFPWFAFGHITSFLHLANKLAEQGHRISFILPPKTQAQMLSHNHHPHLISFIPITLPPVD
GLPTWAETTADVPASSRPLIMTAMDRTQDMIESLLSSLNLDFIFFDFTEWVPGLARKYRVNSVFYATVYVV
TVAYLTPQARNLPVNGLMEENLAGPPPGFPCPSIGLRAHEAQYMANASGVKFGGEISLLERAAIAFRECD
AMGIKSCREMEGPYCDYVIEHYGKPLLLAGPVVPKLPDSKLDEQLEAWLKSFSQGCIVYCALGSECFLKK
DQFQELVLGLELTGMPFLAALKLPIDCPTMESALPYGFTERTKGRAIIHEGWVQQQLILNHPSVGCFVTHC
GAGSLSEGLVSHCQLVMLPQAVDQFLNARMMSQDLKVGVEVEEREGGLITREAVRDAILLVMQEGSAM
GREVRENHAKMREFLVKGGVEDSYISSFVQSLYDLRG

>HU10G01599.1-W

ATGTCAGAAAACAATCACAAACCTTTGCACATAGCCATGTTCCCATGGTTTGCTTTCGGCCACATTACA
TCTTTTCTCCACTTAGCCAACAAACTAGCTGAACAAGGCCACAGAATCTCCTTCATCCTCCCTCCCAA
AACTCAAGCCCAAATGCTCTCTCATAACCACCACCCTCATCTCATTTCGTTCATCCCCATCACTCTCCC
TCCTGTTGATGGGCTCCCCACTTGGGCCGAGACTACGGCCGATGTACCCGCCTCATCCCGGCCCCTTAT
CATGACCGCCATGGACCGGACCCAAGACATGATCGAGTCCCTGCTCTCGAGCCTTAATCTTGATTTCAT
TTTCTTCGACTTCACTGAGTGGGTACCGGGCCTGGCCAGAAAATACCGAGTCAATTCCGTGTTCTATG
CCACCGTGTACGTGGTAACAGTTGCTTACTTGACCCCTCAGGCAAGAAACCTCCCTGTCAATGGCCTT
ATGGAGGAGAATTTGGCGGGTCCGCCACCTGGTTTTCCATGCCCATCTATAGGGCTCAGGGCCCATGA
GGCCCAATACATGGCGAATGCATCAGGGGTGAAATTTGGAGGGGAAATATCACTGCTCGAAAGGGCT
GCAATCGCTTTCCGAGAGTGTGATGCCATGGGGATCAAGAGTTGTAGGGAAATGGAAGGGCCATATTG
TGATTATGTTATTGAGCACTATGGCAAGCCTTTGCTTTTGGCTGGGCCAGTGGTGCCCAAACTGCCGG
ATTCTAAGCTAGATGAGCAGCTTGAGGCCTGGTTGAAGAGTTTTAGTCAAGGATGTATTGTGTATTGTG
CACTTGGGAGTGAATGCTTTCTCAAAAAGGACCAGTTCCAAGAGCTAGTTTTGGGACTTGAGCTCAC

AGGAATGCCCTTCCTGGCAGCTTTAAAGCTACCTATAGATTGTCCTACAATGGAGTCAGCATTACCCTA
TGGCTTCACGGAGCGAACCAAAGGAAGGGCGATCATCCACGAGGGGTGGGTGCAGCAGCAGCTGAT
CTTGAACCACCCTTCAGTTGGGTGCTTCGTGACTCACTGTGGAGCTGGATCCTTGTCAGAAGGTCTAG
TAAGCCACTGCCAACTGGTGATGCTTCCTCAGGCAGTTGATCAGTTTCTGAACGCCAGGATGATGAGC
CAGGACCTCAAGGTTGGCGTGGAGGTCGAGGAGAGGGAAGGTGGTTTGATTACTCGGGAGGCAGTT
CGCGATGCGATACTGTTGGTGATGCAGGAGGGAAGTGCGATGGGGAGGGAGGTGCGGGAAAACCAT
GCTAAGATGAGAGAGTTCTTGGTCAAAGGAGGGGTTGAGGACTCTTATATTAGTAGCTTTGTTCAGAG
TCTGTATGACCTGAGGGGTTGA

MSENNHKPLHIAMFPWFAFGHITSFLHLANKLAEQGHRISFILPPKTQAQMLSHNHHPHLISFIPITLPPVD
GLPTWAETTADVPASSRPLIMTAMDRTQDMIESLLSSLNLDFIFFDFTEWVPGLARKYRVNSVFYATVYVV
TVAYLTPQARNLPVNGLMEENLAGPPPGFPCPSIGLRAHEAQYMANASGVKFGGEISLLERAAIAFRECD
AMGIKSCREMEGPYCDYVIEHYGKPLLLAGPVVPKLPDSKLDEQLEAWLKSFSQGCIVYCALGSECFLKK
DQFQELVLGLELTGMPFLAALKLPIDCPTMESALPYGFTERTKGRAIIHEGWVQQQLILNHPSVGCFVTHC
GAGSLSEGLVSHCQLVMLPQAVDQFLNARMMSQDLKVGVEVEEREGGLITREAVRDAILLVMQEGSAM
GREVRENHAKMREFLVKGGVEDSYISSFVQSLYDLRG

>HU07G01678.1-R

ATGGAAGTTACAAGAAATGGTGAAAATGCAACCAAAATCAAGCTCCATGTAGTTATGTTTCCTTGGTT
TGCATTTGGTCATATAAACCCATTTATCCAGCTATCCAACAAGCTCACCTCCCATGGCATTCAAATCTCT
TTCTTGTCAATACCTGGCAACATTTCTCGGATCCAGTCATCCCTCGATCTGTCTCCTCCCAACCAAATC
ATCCCTCTTACTATCCCTCCTATCGAAGGTCTGCCTCCTGATGTTGACAGCAGCTCTTTCTTACCACCA
CAGGTGTACGAGCTTCTCAAGCTGGCACTTGACCAAATGCAACCCCAAGTGAAGTCCCTTTTAACCC
AACTACATCCCGACATTGTTTTGTTTGACTTTGCACACCATTGGCTCCCTCCACTGGCATCCGAATTGG
GTATCAAGTCTGCCAACTTCTGTGTCTTCTCTGCTGTTGCCACTGCATACCTTACTGTGCCTTCAAGAA
TGCCTGACCCAAATCAACCCCCCACCTTTGAAGACATGAAGAAGCCTCCCCCAGGCTTCCCTGAGAC
CTCGCTTACCGGGATCAAAACATTCCAAGCTCAAGACTTTATGTATCTCTTCAAAAGCTTCTATGAAGG
TCCCCCTGTTTTTGAGAGGATCATGGAGTACACAAAGACTTGTGATGCTGTCATATATAAGTCATGTGT
TGAGTTTGAAGGTCCTTACATAGATTACTTGAAGACCCAGTTGGCGAAACCTCTTCTTTTCGCTGGTC
CTGCTGTTCCCATGCCGCCCTCCGGCATCCTAGAAGCAAAATGGGCAAAATGGTTAGATAATTTCCCTA
AGAATTCTGTCATATTTTGCAATTTTGGGAGTGAGACATACTTAGATGACGTCCAAATAGAGGAGCTG
GCAATTGGGTTGGAGCTAACTGGGTTGCCCTTTTTCCTGGTTCTGAATTTCGGGACCAGTGGTGATGG
TGCCGAGGCTAAGTTAGAAGCATCATTGCCAAAAGGGTTTAGAGAAAGAGTGAAAGAAAGAGGAGT
TGTGCACACAGGGTGGGTGCAGCAACAGCATATACTGGCACACCAAAGTGTAGGGGGTTATGTGAAC
CACTCAGGATTCAGCTCTGTGATTGAGGCCCTTGTGAATGATTGTCAGTTGATAATGATTCCCCAAAAG
GGGGATCAGCACATAAATTCGAAGTTGCTGAGTAGGGTTCTCAAGGTAGGAGTGGAGGTGAATAGGA
GAGATGAAGACGGGTATTTTGCGAGGGAGGATATTCTGAATGCAGTTCGAACAGTTATGCTAGAAGTT
GGTAAGGAACCAGGGAGGTCTATTAGGGCTCATCAGAAGAAATGGAAAGAGTTTCTCCTGAATAAAG
AGATTGAAGACAAATACATTGCCAATTTGGTCGAGGAACTCAAGGCTTTGGCTCATGAAGCTTAG

MEVTRNGENATKIKLHVVMFPWFAFGHINPFIQLSNKLTSHGIQISFLSIPGNISRIQSSLDLSPPNQIIPLTIPP
IEGLPPDVDSSSFLPPQVYELLKLALDQMQPQVKSLLTQLHPDIVLFDFAHHWLPPLASELGIKSANFCVFS
AVATAYLTVPSRMPDPNQPPTFEDMKKPPPGFPETSLTGIKTFQAQDFMYLFKSFYEGPPVFERIMEYTKTC
DAVIYKSCVEFEGPYIDYLKTQLAKPLLFAGPAVPMPPSGILEAKWAKWLDNFPKNSVIFCNFGSETYLDD
VQIEELAIGLELTGLPFFLVLNFGTSGDGAEAKLEASLPKGFRERVKERGVVHTGWVQQQHILAHQSVGG
YVNHSGFSSVIEALVNDCQLIMIPQKGDQHINSKLLSRVLKVGVEVNRRDEDGYFAREDILNAVRTVMLE
VGKEPGRSIRAHQKKWKEFLLNKEIEDKYIANLVEELKALAHEA

>HU07G01678.1-W

ATGGAAGTTACAAGAAATGGTGAAAATGCAACCAAAATCAAGCTCCATGTAGTTATGTTTCCTTGGTT
TGCATTTGGTCATATAAACCCATTTATCCAGCTATCCAACAAGCTCACCTCCCATGGCATTCAAATCTCT
TTCTTGTCAATACCTGGCAACATTTATCGGATCCAGTCATCCCTCGATCTGTCTCCTCCCAACCAAATC
ATCCCTCTTACTATCCCTCCTATCGAAGGTCTGCCTCCTGATGTTGACAGCAGCTCTTTCTTACCACCA
CAGGTGTACGAGCTTCTCAAGCTGGCACTTGACCAAATGCAACCCCAAGTGAAGTCCCTTTTAACCC
AACTACATCCCGATATTGTCTTGTTTGACTTTGCACACCATTGGCTCCCTCCACTGGCATCCGAATTGG
GTATCAAGTCTGCCAACTTCTGTGTCTTCTCTGCTGTTGCCAATGCATACCTTATTGTGCCTTCAAGAAT
GCCTGACCCAAATCAACCCCCCACCTTTGAAGACATGAAGAAGCCTCCCCCAGGCTTCCCTGAGACC
TCGCTTACCGGGATCAAAACATTCCAAGCTCAAGACTTTATGTATCTCTTCAAAAGCTTCTATGAAGGT
CCCCCTGTTTTTGAGAGGATCATGGAGTACACAAAGACTTGTGATGCTGTCATATATAAGTCATGTGTT
GAGTTTGAAGGTCCTTACATAGATTACTTGAAGACCCAGTTGGGGAAACCTCTTCTTTTCGCTGGTCC
TGCTGTTCCCATGCCGCCCTCCGGCATCCTAGAAGCAAAATGGGCAAAATGGTTAGATAATTTCCCTA
AGAATTCTGTCATATTTTGCAATTTTGGGAGTGAGACATACTTAGATGACGTCCAAATAGAGGAGCTG
GCAATTGGGTTGGAGCTAACTGGGTTGCCCTTTTTCCTGGTTCTGAATTTCGGGACCAGTGGTGATGG
TGCCGAGGCTAAGTTAGAAGCATCATTGCCAAAAGGGTTTAGAGAAAGAGTGAAAGAAAGAGGAGT
TGTGCACACAGGGTGGGTGCAGCAACAGCATATACTGGCACACCAAAGTGTAGGGGGTTATCTGAAC
CACTCAGGATTCAGCTCTGTGATTGAGGCCCTTGTGAATGACTGTCAGTTGATAATGATTCCCCAAAA
GGGGGATCAGCACATAAATTCGAAGTTGCTGAGTAGGGTTCTCAAGGTAGGAGTGGAGGTGAATAGG
AGAGATGAAGACGGGTATTTTGCGAGGGAGGATATTCTGAATGCAGTTCGAACAGTTATGCTAGAAGT
TGATAAGGAACCAGGGAGGTCTATTAGGGCTCATCAGAAGAAATGGAAAGAGTTTCTCCTGAATAAA
GAGATTGAAGACAAATACATTGCCAATTTGGTCGAGGAACTCAAGGCTTTGGCTCATGAAGCTTAG

MEVTRNGENATKIKLHVVMFPWFAFGHINPFIQLSNKLTSHGIQISFLSIPGNIYRIQSSLDLSPPNQIIPLTIP
PIEGLPPDVDSSSFLPPQVYELLKLALDQMQPQVKSLLTQLHPDIVLFDFAHHWLPPLASELGIKSANFCVF
SAVANAYLIVPSRMPDPNQPPTFEDMKKPPPGFPETSLTGIKTFQAQDFMYLFKSFYEGPPVFERIMEYTKT
CDAVIYKSCVEFEGPYIDYLKTQLGKPLLFAGPAVPMPPSGILEAKWAKWLDNFPKNSVIFCNFGSETYLD
DVQIEELAIGLELTGLPFFLVLNFGTSGDGAEAKLEASLPKGFRERVKERGVVHTGWVQQQHILAHQSVG
GYLNHSGFSSVIEALVNDCQLIMIPQKGDQHINSKLLSRVLKVGVEVNRRDEDGYFAREDILNAVRTVML
EVDKEPGRSIRAHQKKWKEFLLNKEIEDKYIANLVEELKALAHEA
