# Supplementary material for: A Genome-Wide Identification Study Reveals That HmoCYP76AD1, HmoDODAα1 and HmocDOPA5GT Involved in Betalain Biosynthesis in Hylocereus
Source: Genes (Basel). 2021 Nov 23;12(12):1858. doi: 10.3390/genes12121858 (PMC8702118; doi:10.3390/genes12121858)
Supplement: Supplementary file 1 [file genes-12-01858-s001.zip › Supplementary Text S2. cDNA sequences of genes for VIGS in red pitaya.pdf]

Text S2. cDNA sequences of genes for VIGS in red pitaya.

CYP76AD1

> HU03G00480.1-R

ATGGATAGCCCAACCCTCTCGCTTTTCATCTTTGCCTCAATCTTTTACTTCATAACTTTTCA
AATTGTGAAGCTAGGGTTTAATGTGGTCATGACCTCTAAAAAAACCAAAAGAAGAAGAC
CCCCCTTACCTCCGGGCCCCAAGCCATTGCCCATCATAGGCAATGTGCTCGAGCTCGGGC
CGAAGCCACACCGCTCGTTCGCCGACCTAGCCAAGGTCCATGGTCCACTCATGTCCCTC
CGGCTAGGTAGTGTGACCACGATTATCGTGTCATCCTCTGATGTTGCCAAAGAAATGTTC
CTTAAAAATGACCAACCCTTGAGCTCCAGCCGAACCATACCCAACTCAGTCACGGCTGG
GGATCACCACATGCTGACCATGTCTTGGCTCCCAGTCTCCCCCAAATGGCGGAGTTTAG
GAAGATCACCACCTTCCACCTTCTCTCCCCCCAGCGCCTCGATGCTTGCTCTAGCCTTAG
GCAAGCCAAGGTGCAGCAGCTATTCGAGTACGTTCTGGAATGTTCTAGAACCGGCCAGG
CCGTCGATATAGGCAAGGCTGCTTTCACGACGTCCCTTAACTTGTTGTCCAAGCTGTTTT
TTTCTTTAGAGTTGGCTCACCATAGATCTAGCAAGTCTCAAGAGTTTAAGGACTTAATTT
GGGATATTATGGAGGATATTGGGAAGCCTAATTACGCGGATTATTTCCCATGCTTAAAGTA
CTTTGACCCATGTGGAATACGACGTCGTTTGGCAAATAGTTTTGAGAAATTAATTGAGGT
CTTTCAAGGTATTATTCGTCAAAGGCTATCCCTGTCATCTGGCTCTCATACTCATAATGAT
GTGTTAGATGTTCTTCTTCAATTGTACAACCAAGAGGAACTCACCATGGACGAGATAAA
CCATCTGCTCGTGGATATATTTGATGCCGGAACAGACACCACTTCCAGTACATTTGAATG
GGCCATGGCTGAGTTAATTAAAAATCCGACGATGATGGAGAAAGCTCAAGCTGAAATCA
AAGTGGTTCTTGGGAAACAGTCGCATATTCAAGAGTCCGATATCCCAAAATTGCCTTATT
TGCGGGCAATTATCAAAGAAACATTGCGTCTACACCCTCCTACTGTATTCCTCCTACCTC
GTAAGGCTGAGACCGATGTGGAACTCTATGGCTACACCGTACCAAAAAATGCACAAATA
CTGGTGAACTTGTGGGCCTTAGGTCGAGACCCCAAAGTTTGGGAAAACCCAGAGGTGT
TCTTACCTGAAAGGTTCCTGACTTGCGACATCGATGTTAAAGGAAGAGATTTTGGACTAC
TGCCTTTTGGGGCAGGAAGGCGAATATGTCCTGGGATGAATTTGGCGTACAGAATGCTG
ACCTTAATGCTCGCTACGCTTCTACAATCGTTTGATTGGAAACTCCCAAATGAGATGAAC
TCCAAGAATTTGGACATGGATGAAAAGTTTGGAATAGCATTGCAAAAGACTAAACCCCT
TGAAATTATTCCCGTTTGCAAGGATTGA

DODAα

> HU03G01342.1-R

ATGGGTGTTGGCAAAGAAGTGTCGTTCAAGGAGACTTTCTATGTGTCTCATGGGAATCC
GGCCATGTTGGCCGATGTGTCGTTCATAGCACGGAACTTCCTGCTGGGGTGGAAGACGA
ATGTCTTCCCCATCAAACCCAAGTCGATCCTGGTTGTCTCTGCTCACTGGGAGACTGATG
TGCCTTCTGTATCTGCCGGTGAACATCCTGATGTCATTTACGATTTCAGCGATGTTCCTGA
CTGTATGTTCCAGATGAAGTACCCAGCTCTAGGGTCACCAAAACTGGCCAAAAGGGTGC
AGGAGCTACTGATAGCAGGAGGGTTCAAGACAGCGAGCCTAGACGAGAGTCGTGGGTT
CGACCACAGCTCATGGGTGCCCCTGAGCCTCATGTACCCTGAGGCTGACATCCCGGTGT
GCCAGCTCTCAGTCCAGCCTCACCTAAGCGCGAGCCACCACTTCGACATAGGGAGGGCT
TTGGCTCCTCTCAAGGAGGAAGGGGTCCTGTTCATTGGGTCTGGGGGTGCAGTGCACCC
TTCTGATGACACCCCACACTGGTCTGATGGGGTTGCCCCTTGGGCTGCTGAGTTTGATCA
ATGGCTTGAGGATGCTCTCATTAATGGAAGGTACGATGATGTGAATAATTATCAAACAAA
AGCACCTTCTGGGTGGAAAATAGCACATCCAATTCCAGAACACTTTTTACCGTTGCATGT
AGCCATGGGTGCAGCTGGTGAAAAATCAAAGGCAGAGCTCATTTATCGTACGTGGGATC
ATGGTACTCTTGGCTATGCCTCCTACAAGTTCACTTCCATCTGA

> HU03G01342.1-W

ATGGGTGTTGGCAAAGAAGTGTCGTTCAAGGAGACTTTCTATGTGTCTCATGGGAATCC
GGCCATGTTGGCCGATGTGTCGTTCATAGCGCGGAACTTCCTGCTGGGGTGGAAGAAGA
ATGTCTTCCCCATCAAACCCAAGTCGATCCTGGTTGTCTCTGCTCACTGGGAGACTGATG
TGCCTTCTGTATCTGCCGGTGAACATCCTGATGTCATTTACGATTTCAGCGATGTTCCTGA
CTGCATGTTCCAGATGAAGTACCCAGCTCCAGGGTCACCAAAACTGGCCAAAAGAGTG
CAGGAGCTACTGATAGCAGGAGGGTTCAAGACAGCGAGCCTAGACGAGAGTCGTGGGT
TCGACCACAGCTCATGGGTGCCCCTGAGCCTCATGTACCCTGAGGCTGACATCCCGGTG
TGCCAGCTCTCAGTCCAGCCTCACCTAAGCGCGAGCCACCACTTCGACGTAGGGAGGG

CTTTGGCTCCTCTCAAGGAGGAAGGGGTCCTGTTCATTGGGTCTGGGGGTGCAGTGCAC

CCTTCTGATGACACCCCACACTGGTTTGATGGGGTCGCCCCTTGGGCTGCTGAGTTTGAT

CAATGGCTTGAGGATGCTCTCATTAATGGAAGGTACGATGATGTGAATAATTATCAAACA

AAAGCACCTTCTGGGTGGAAAATAGCACATCCAATTCCAGAACACTTTTTACCGTTGCAT

GTAGCCATGGGTGCAGCTGGTGAAAAATCAAAGGCAGAGCTCATTTATCGTACGTGGGA

TCATGGTACTCTTGGCTATGCCTCCTACAAGTTCACTTCCATCTGA

>HU08G01572.1-R

ATGCTTCAGCAACTAATTAGTGGATGGCAGAGATTGGGCATTGGCTGTCCTTATCTTGATT

ATAAGTATCTCGTGACAGGCAGAGAAGTGGGTACACTTCTAATAGCTTCTAAGATCAAA

ACAAGAGAAAAACTAAATTTCATTTGCACCAGCAAAATGGGTGAGCAGGAAGCAATTA

GGGAGACATTCTACATATCTCATGGGACACCAAAGATGTCGATCGATGATTCTATAGCAG

CAAAGAAGTTCTTTCAGGAATGGGAGAAAGTCTACTCCAAGAGGCCAAAGTCTATGCTT

GTGATCTCTGCTCACTGGGAGACTGATGTGCCTGCTGTTAATGCTGTTACTCACTCTGAT

CTCATCTATGATTTCAGGGGCTTTCCTGCCATCATGTACCAGCTCAAGTACCCAGCTCCTG

GTGCACCAGATTTGGCAAGGAGGGTAGAGGAACTTCTGACAGCATCAGGGTTCAGCTG

TGTTGTAGACAAGAAGCGCGGTCTTGATCATGGCTCATGGGTGCCTCTCATGCTCATGTA

CCCGGAGGCTGATATCCCGGTTTGCCAGCTCTCAGTGCAATCACACCTAGATGGAACGC

ACCACTATAACTTGGGAAAGGCTTTGGCTCCTCTTAAGGAGGAAGGTGTCCTTATCATTG

GTTCTGGGAGTGCTGTTCACCCTTCAAATAGTACTCCTATTTCTCTTGATGGAGTTGCTCC

TTGGGCTGCAGCGTTCGATTCCTGGCTAGAAGAAGCTCTCAAAAGTGGAAGGTATGAAG

ATGTGAACAATTATCAAGCAAAGGCACCAAACTGGGAACTGGCACACCCATGGCCAGA

GCACTTTTACCCATTACATGTAGCCATGGGTGCAGCTGGTGAAAACTCGAAGGCCGAGC

TTATTCATCGCAGtTGGGACCATGGCACCCTTGGCTATGCCTCCTACATATTCACTTCCTGC

TGA

B5GT

> HU01G00156.1-R

ATGAACATGGCTGCTGAAGATCAACAGCTTCATGTAGTGTTCTTCCCCTTCTTGGCCCAT

GGCCATATGATTCCGACCCTCGATGTTGTAAGGCTCTTTGCATGTCATGGGGTCAGGGTC

ACCATCATCACCACCCCTCTGAACGCCCACATCTTCAATAAGGCTGTTGAGACGGCTAC
GAAAATGGGTGCCCGGGTCGTTGATGTTGAGCTATTCAGGTTCCCTGCCGAAGAAGCAG
GCCTGCCAGAAGGATTTGAGAACTTAGAGCTAGTTAAGGAGGATGACATGATTGTTAAG
TTCATCGCTGCCAGTGAGCTGCTCAGAGAGCAGCTTGAGCCATACTTGGAGAAGACACG
ACCGAACTGCCTCGTTGCTGACATGTTTTTCCATGGGCAACGGAGTGTGCAGCCAAGT
TCGACATCCCTAGGCTAGTCTTCCATGGCACTAACTTCTTTTACTTGTGTGCTTCGGAGAT
TATCAGGCTATATGAGCCTTATAAGAATGTCTCTTCGGATGAGGAGCCATTCGCTCTTCCG
TCCCTTCCACATGAGATACAGATGACAAGACTGCAGCTACCAGAGGATCTATGGAAGTA
CGAGGAATCCGATTTCAAAAGGAGGATAGCACTAATCCACGAATCGGAGGTTGCAAGCT
ATGGGGTGATCGTCAACAGCTTTTATGAGTTGGAACCGGATTATGCAGATTTCTTCGGGA
AGGAATTAGGAAGGAGGGCATGGCATATAGGCCCAGCTTTACTCTGCAATAGGAGTACC
AAGAACAAAGCTCAGAGGTTGAAACAACCCGATCTAGATGAACACGAGTGCCTGAAGT
GGCTTAACTCAAGGAAGCCCAACTCAGTCATTTACATCTGTTTCGGAAGCACTGCACAT
GTGATTGGCCCTCAGCTACATGAGATAGCAGTGGCTTTGGAAGCGTCAGAACAAGCGTT
CATTTGGGTAGTGAAGAATGAGGATTACGAAAAGAGTGCAGAGTGGTTGCCCACAGTG
TTTGAGGAGCGGTTGGAACAACGAGGTCTAATTATAAAAGGGTGGGCTCCTCAGGTGCT
TATTTTAGAGCATGAAGCTATAGGAGCATTTGTGACCCACTGTGGATGGAACTCGATCTT
AGAAGGGATCTCGGCTGGGGTGCCAGTGGTTACATGGCCTGTTTTTGCTGAGCAGTTCT
ATAACGAAAGGTTCGTGACTCAGATTTTGAAGATTGGGGTGCCCATTGGATCTAAGAAG
TGGGGTATTGTGACTACTACAGAGTACATGGTGAAACGGGACTCTATAGAGAAGGCATT
GAGAGAAATCATGGTAGGAGAGGAAGCTGAGGAGAGGAGAAACAGAGCACAGAAGTT
GAAGGCGATGGCATGGAAGGCTGTCGAAGAAGGGGGTTCATCTTATTCTGATCTAAGTG
CACTAATAAATGAGCTTAGGGTTTATCGCACTTAA

cDOPA5GT

>HU07G00240.1-R

ATGGACTCTGCAACAGAGCATATACTGATGCTCCCATACATGGCGCAGGGTCACATTCGC
CCGTTCATCGAGCTAGCCCACCGCCTCCAAGCCCGAACTGAAACCAGAACCCGAACCC
ACTTCACCATCACCATCCTCACCACCCCTCTCAACGCTGAGCTCATCCGACGCCAATTCA

CCACCACCTCCACCATCCAAATTGCCGAGCTCCCCTTCAACGCCGCCGACCATGGCCTC
CCTGCTGGCGTCGAGAACACCGACAAGCTCCCGTTCCCCCTCGTCGTCAACCTCTTCCA
CGCCTCCTCCTCGCTTGAACCCCACCTCCGCCACTTCATCGCCACCCGCTTCTCCCCCTG
TAAACCCCCTCTCTGCATTGTCTTCGACGTCTTCCTGGGATGGGTTGACGCCGTCGCTAG
GAGCTTTGGATCCACCGGAGTCGCCTTCACCACCGGCGGCGCGTATGGGACGGCGGCGT
ATTTGTCACTGTGGCTTGATCTACCTCACCGAAACTTCTCAGACGATGAGGACTTCCCTT
TGCCTGGATTCCCGGAGAACCACCGCTATCGCCGCTCCCAGCTCCACCGGTTCTTAAGAT
ACGCTGATGGGTCGGATCAGTGGTCGCGGTTTTTCCAGCCCCAGCTGCGGTTCTCAATT
GGTAGCCTCGGATGGCTCTGTAATTCCGTCGAGGAGATTGAGCCTCTGGGGTTTGAAATT
CTTCGGAATTACCTCAAATCACCGGTCTGGGCAGTCGGGCCGTTGATTCCGGATCCTCCG
CCGGTGGATAAATCATCATCTTCTTGTGACAAATGCATCCAGTGGTTAAATTCCCACAAA
GCCGATTCCGTTTTATACATCTCATTCGGGTCGCAGAATACGATTTCCCCGACCCAGATGA
TGGAGCTGGCTATGGGTTTAGAGCTAAGTGGGAAGCCATTTTTGTGGGTGATCCGGCCT
CCGTTCGGGTTCGACATCAACGGAGAATTCCGACTCGAATGGCTTCCGGATGGGTTCGA
GGAGCGCGTGAAGAACAACAACCAGGGGATGTTGGTGCATAAATGGGGGCCTCAAATG
GAGATACTGGGTCATGAATCAACGGGTGGGTTTCTCAGCCATTGTGGGTGGAACTCGGT
GTTGGAAGGACTGAGAGAGGGACTACCGATGATAGCGTGGCCATTGGCTGGGGAGCAG
GCGTACAATTCGAAGATGCTGGTGGAGGAGATGGCGGTGGCAGTGGAGCTGACGACGG
GGTTGGAAGGGAAGGTGACTAAGGAGGCGGTGAAGAGGGTGGTGGAGCTGGTGTTGG
GGAGAGAGGAAGGGAGTGGTGGCGAGGAGATGAAGAAGAAGGCGGTTGAAGCAGGG
AAGAAGCTTAGAGATGCATTAAGAGAAGAGGAAGATGGGTTAGGGTTTAAGGGATCTTC
GATTAAAGCAATGGATGAGTTTTTGGATGCCATAGTTTCCGGCAGGGATCGACCAGCTGC
TTGA
