# Supplementary material for: A Genome-Wide Identification Study Reveals That HmoCYP76AD1, HmoDODAα1 and HmocDOPA5GT Involved in Betalain Biosynthesis in Hylocereus
Source: Genes (Basel). 2021 Nov 23;12(12):1858. doi: 10.3390/genes12121858 (PMC8702118; doi:10.3390/genes12121858)
Supplement: Supplementary file 1 [file genes-12-01858-s001.zip › Supplementary Text S3. Vectors used in this study.pdf]

Text S3. The information of all vectors used for functional analyses

1) Diagrams of the constructed vectors.

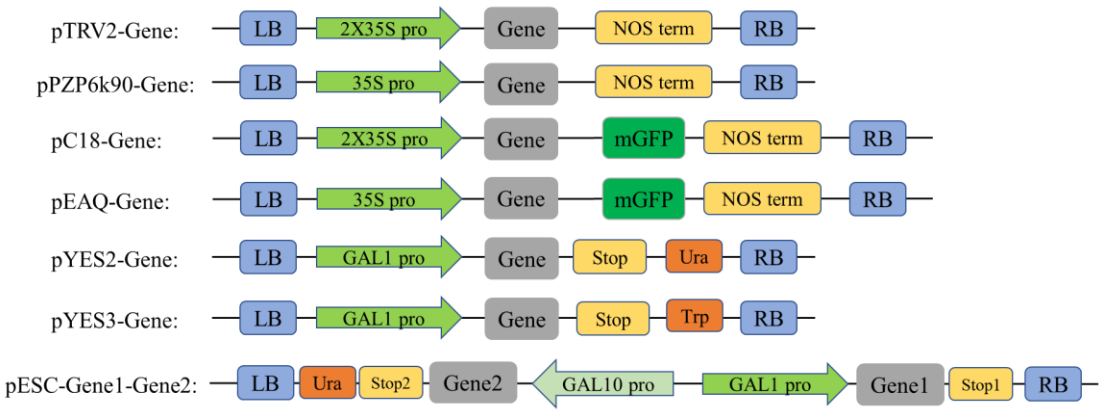

2) The detail information of all vectors

pTRV1 and pTRV2:

Liu Y, Schiff M, Marathe R, Dinesh-Kumar SP. (2002b). Tobacco Rar1, EDS1 and NPR1/NIM1 like genes are required for N-mediated resistance to tobacco mosaic virus. Plant J 30: 415–429.

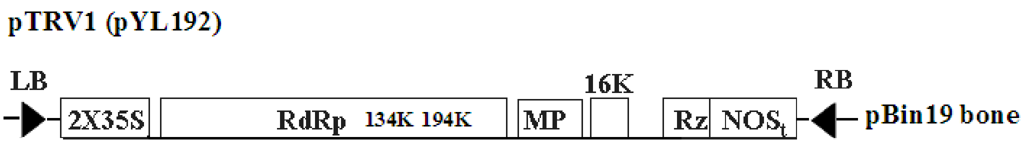

The entire sequence of cDNA corresponding to TRV Ppk20 strain RNA1 is deposited in GenBank (AF406990).

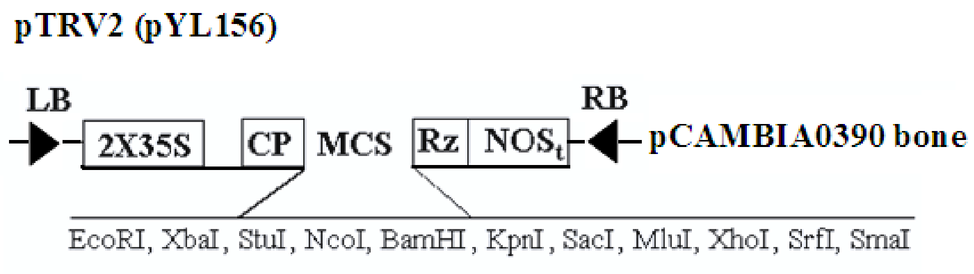

Plasmid pTRV2 was generated by subcloning the *Hind* III-*Eco*R I restricted fragment

of pYL36 into *Hind* III-*Hpa* I restricted pCAMBIA0390 T-DNA vector. The complete sequence of pTRV2 is deposited in GenBank (AF406991).

pC18: Transient expression of recombinant proteins in plants.

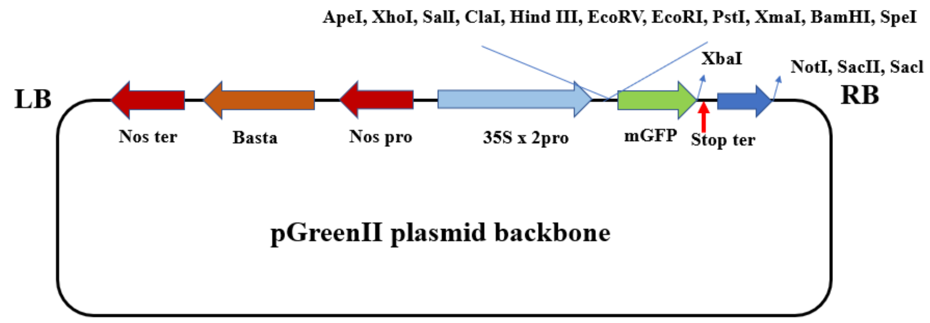

pEAQ-GFP: Transient expression of recombinant proteins in plants. The plasmid was produced from pEAQ-HT by insertion of GFP.

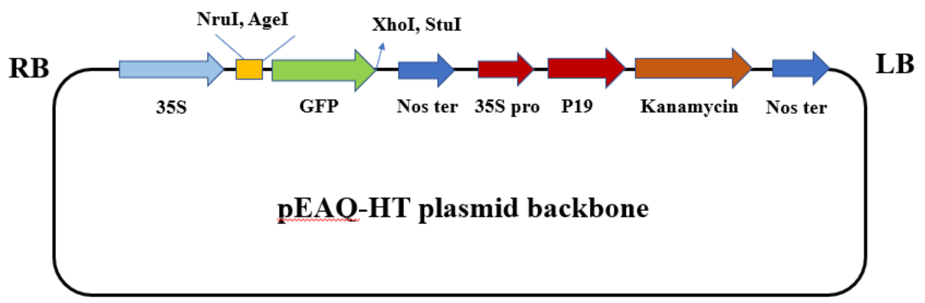

pPZP6k90: Genetic expression of recombinant proteins in plants. The plasmid was construct by our lab.

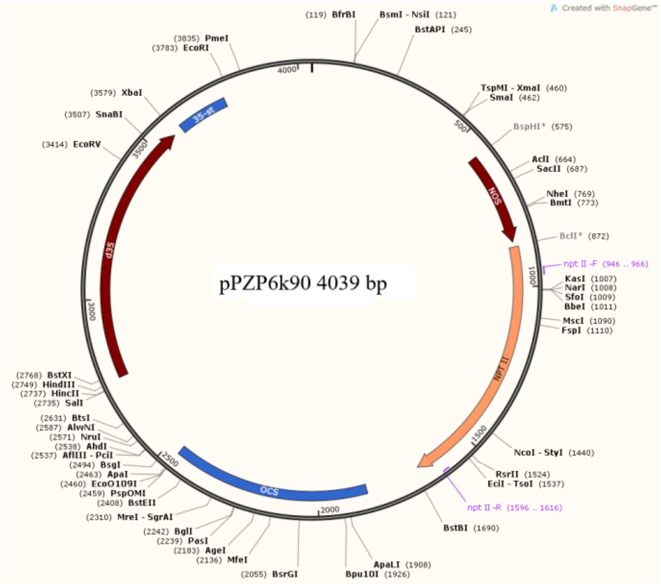

Plasmid circular map

pYES2/CT and pYES3/CT (Invitrogen, USA): pYES2/CT and pYES3/CT are 6.0 kb and 5.9 kb vectors, respectively, designed for inducible expression of recombinant proteins in *Saccharomyces cerevisiae*.

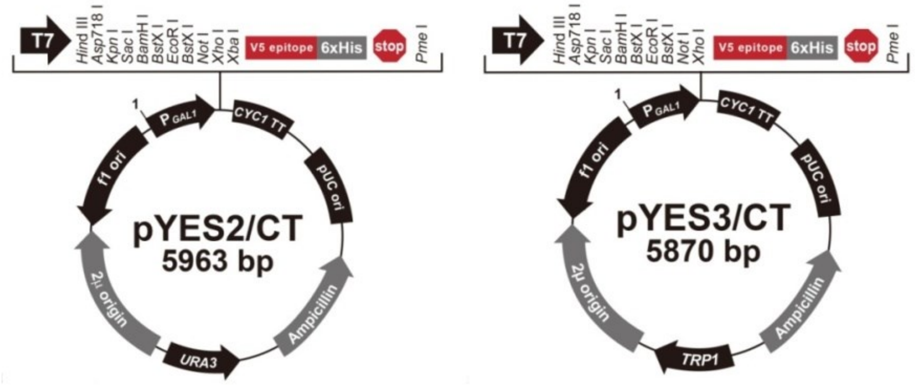

Plasmid circular map

pESC-Ura (https://www.addgene.org/vector-database/7711/): The pESC vectors are a series of epitope-tagging vectors for expression and functional analyses of eukaryotic genes in the yeast *S. cerevisiae*. These vectors contained the GAL1 and GAL10 yeast promoters in opposing orientation. Vectors with one or two genes were introduced into the yeast host strains under the control of a repressible promoter. When two genes are

co-expressed, protein-protein interactions can be confirmed by immunoprecipitation analysis.

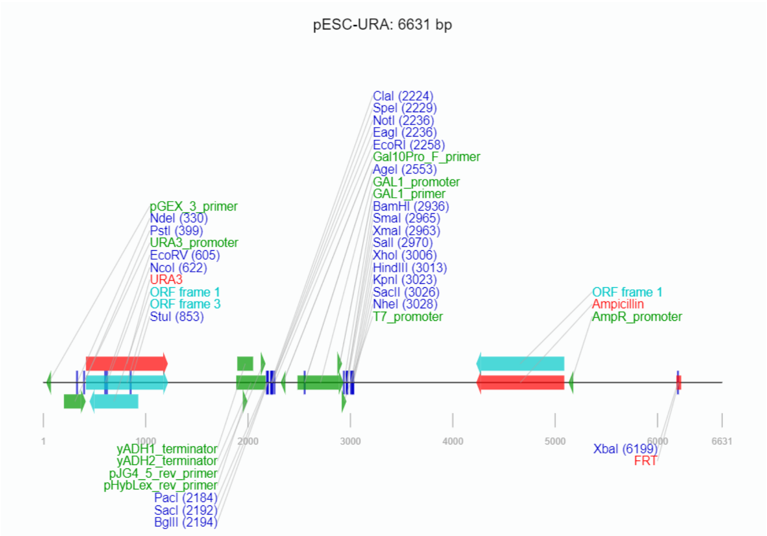

Plasmid linear map
