# Supplementary figures and images for: A Genome-Wide Identification Study Reveals That HmoCYP76AD1, HmoDODAα1 and HmocDOPA5GT Involved in Betalain Biosynthesis in Hylocereus
Source: Genes (Basel). 2021 Nov 23;12(12):1858. doi: 10.3390/genes12121858 (PMC8702118; doi:10.3390/genes12121858)

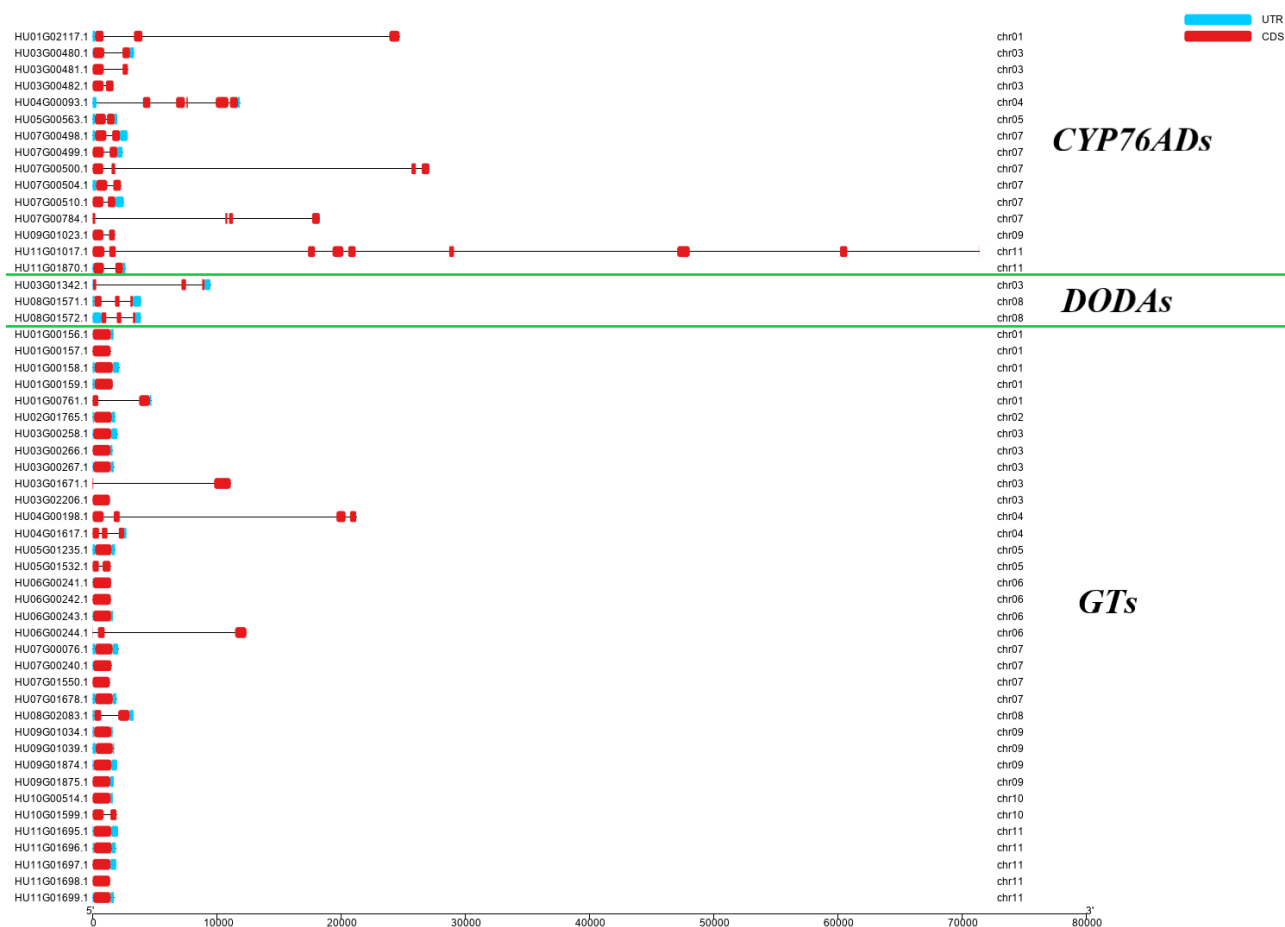

**Figure S1. Gene structures of 53 genes.**

Supplement: Supplementary file 1 [file genes-12-01858-s001.zip › Supplementary Figure S1. Gene structures of 53 genes.pdf]
